# Stereospecific Coupling of Alcohols and Carbanion Nucleophiles through a Circular P(V) Activation Manifold

**DOI:** 10.1021/acs.orglett.5c04289

**Published:** 2025-12-04

**Authors:** Isabel L. Wood, Stephen P. Argent, Ross M. Denton

**Affiliations:** † The GlaxoSmithKline Carbon Neutral Laboratories for Sustainable Chemistry, 6123 University of Nottingham, Jubilee Campus, Triumph Road, Nottingham NG7 2TU, U.K.; ‡ Chemistry, 6123 University of Nottingham, University Park, Nottingham NG7 2RD, U.K.

## Abstract

We report a method for the stereospecific construction of sp^3^–sp^3^ carbon–carbon bonds using alcohols and carbanion nucleophiles. The process is based on a circular phosphorus­(V) manifold which starts and ends with triphenylphosphine oxide. The redox neutral approach eliminates the need for hazardous diazodicarboxylate oxidants and allows for recovery and recycling of triphenylphosphine oxide. Alcohol activation is achieved *in situ* through formation of alkoxyphosphonium triflates, which are kinetically stable, and undergo coupling with exogenous carbanion nucleophiles. In this manner, a range of alcohols undergo deoxyalkylation with inversion of configuration in moderate to excellent yield.

## Graphical abstract

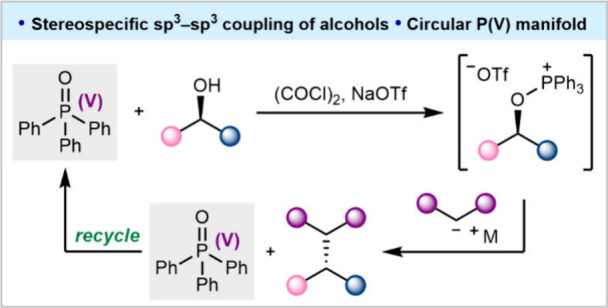

Strategies to construct sp^3^–sp^3^ carbon–carbon bonds are integral to the field of synthetic organic chemistry, and a wide range of cross-coupling reactions have been developed to facilitate this process.^1–4^ Of the many possible coupling partners available, alcohols are considered as attractive since they are abundant and easy to handle.^5^ For these reasons, alcohol coupling processes are highly sought after, and a variety of methods have been reported using primary and secondary alcohols.^6^ Coupling reactions of secondary alcohols are of particular interest since they provide an opportunity to form of sp^3^–sp^3^ linkages with control of stereochemistry. While stereospecific alkylations of preformed secondary mesylates have been reported,^7^ there are limited methods for direct, stereospecific nucleophilic substitution of alcohols with carbon nucleophiles. For example, Tsunoda and others have demonstrated Mitsunobu coupling reactions using suitably Brønsted acidic carbon pronucleophiles, such as triethylmethane tricarboxylate and Meldrum’s acid (Scheme 1A).^8–33^ While these processes enable stereocontrolled coupling in some cases,^21–33^ they take place in the conventional P­(III)/P­(V) Mitsunobu manifold in which alcohol activation is achieved at the expense of a phosphine and an oxidant (Scheme 1A).^34^ Some oxidants, such as diazodicarboxylates, present practical problems as a result of toxicity and difficulty in removal of the resultant hydrazine byproduct which generates additional downstream waste.^35–37^ As a result of these limitations, elegant catalytic Mitsunobu reactions have been developed by the groups of Toy,^38^ Taniguchi^39–41^ and Aldrich,^42^ which allow for the cycling of a substoichiometric quantity of the diazocarboxylate in the presence of terminal oxidants. However, no examples of carbon–carbon bond forming reactions have yet been demonstrated within these or any other catalytic manifolds.^38–43^ Here we describe a simple, redox-neutral phosphorus­(V)-mediated process for the coupling of alcohols with carbanion nucleophiles. Our reaction design is depicted in Scheme 1B and is based upon a circular activation manifold that starts and ends with triphenylphosphine oxide. This is desirable since it eliminates hazardous oxidants and allows for the phosphine oxide to be recycled.

**Scheme 1:**
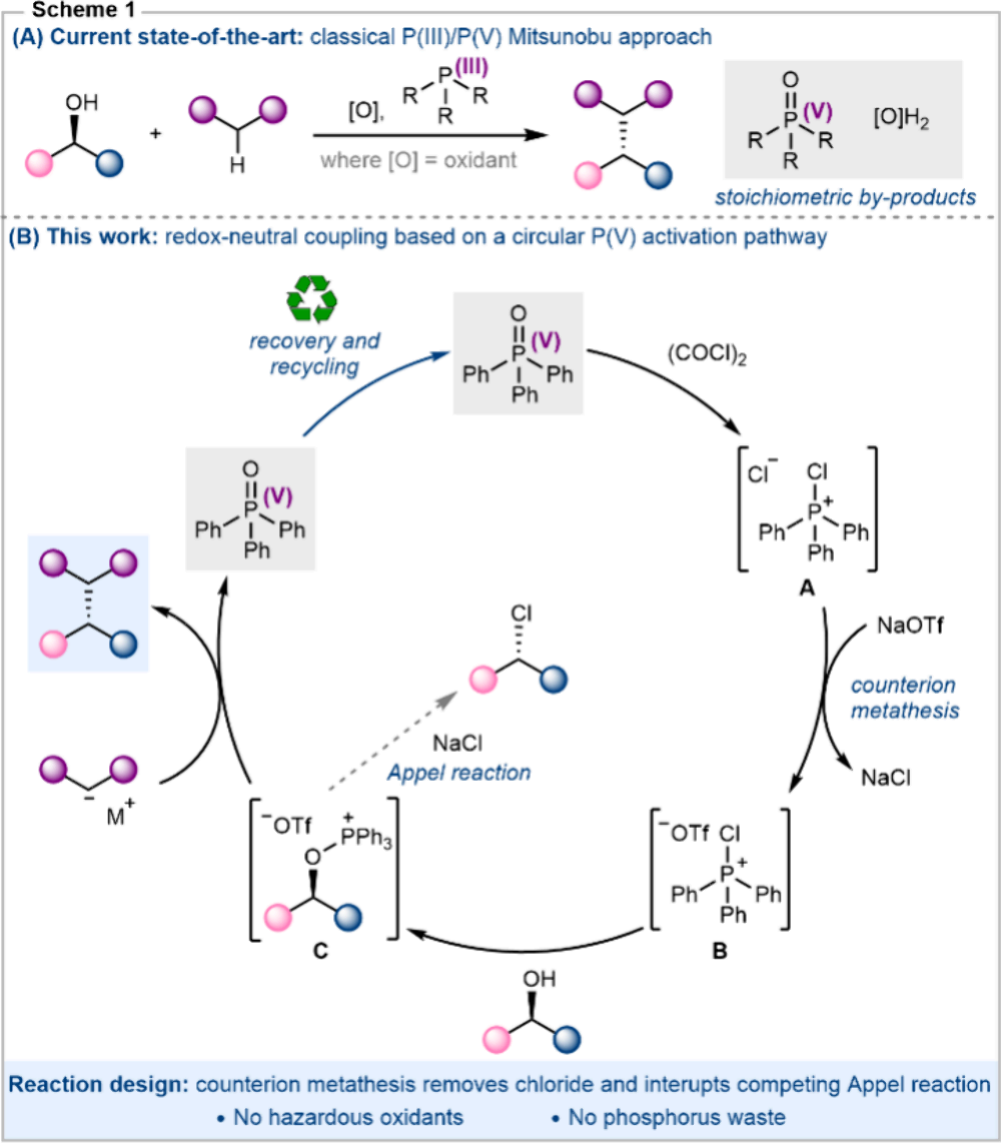
(A) Mitsunobu-Based P­(III)/P­(V) Coupling; (B) Redox-Neutral P­(V) Coupling Based on a Circular Activation Manifold

The key aspect of our reaction design is phosphine oxide activation using oxalyl chloride^44–46^ in the presence of sodium triflate. We reasoned that this additive would promote counterion metathesis generating phosphonium triflate **B** from the initially formed chlorophosphonium salt **A** (Scheme 1B). Critically, this should allow alcohol activation to occur through phosphonium triflate **B** giving rise to alkoxyphosphonium salt **C**, which has a non-nucleophilic triflate counteranion. In this manner, the competing deoxychlorination process (Appel reaction)^47–58^ should be suppressed, and alkoxyphosphonium salt **C** should be accessible to exogenous nucleophiles as shown. While this approach seemed attractive, we were aware at the outset that the solution speciation of the phosphonium intermediates, and their associated counteranions would likely be more complex than depicted in Scheme 1B. Furthermore, no literature precedent was available for the generation of alkoxyphosphonium triflates through the proposed interrupted Appel reaction. For these reasons, we began by investigating the feasibility of the metathesis and alcohol activation processes.

First, we examined the competing Appel deoxychlorination reaction using alcohol **A1** as a model compound (Scheme 2A). To this end, alcohol **A1** was added to an *in situ* generated solution of chlorotriphenylphosphonium chloride in THF, obtained by treatment of triphenylphosphine oxide **1** with oxalyl chloride. As expected, deoxychlorination of the alcohol occurred readily, and 50% of alkyl chloride **2** was isolated after 5 h at room temperature. We next carried out an analogous reaction with the addition of 3.00 equiv of sodium diethylmalonate **3** as a representative external carbanion nucleophile (Scheme 2B). In this instance, a mixture of alkylated product **4a** (29%) and alkyl chloride **2** (36%) was observed (Scheme 2B). This result demonstrates that, while the addition of external nucleophile and subsequent coupling is feasible, it will not be efficient in the absence of an additive to control phosphonium salt speciation and access the desired alkoxyphosphonium triflate intermediate. Therefore, we next examined the use of additives to effect counterion metathesis.

**Scheme 2:**
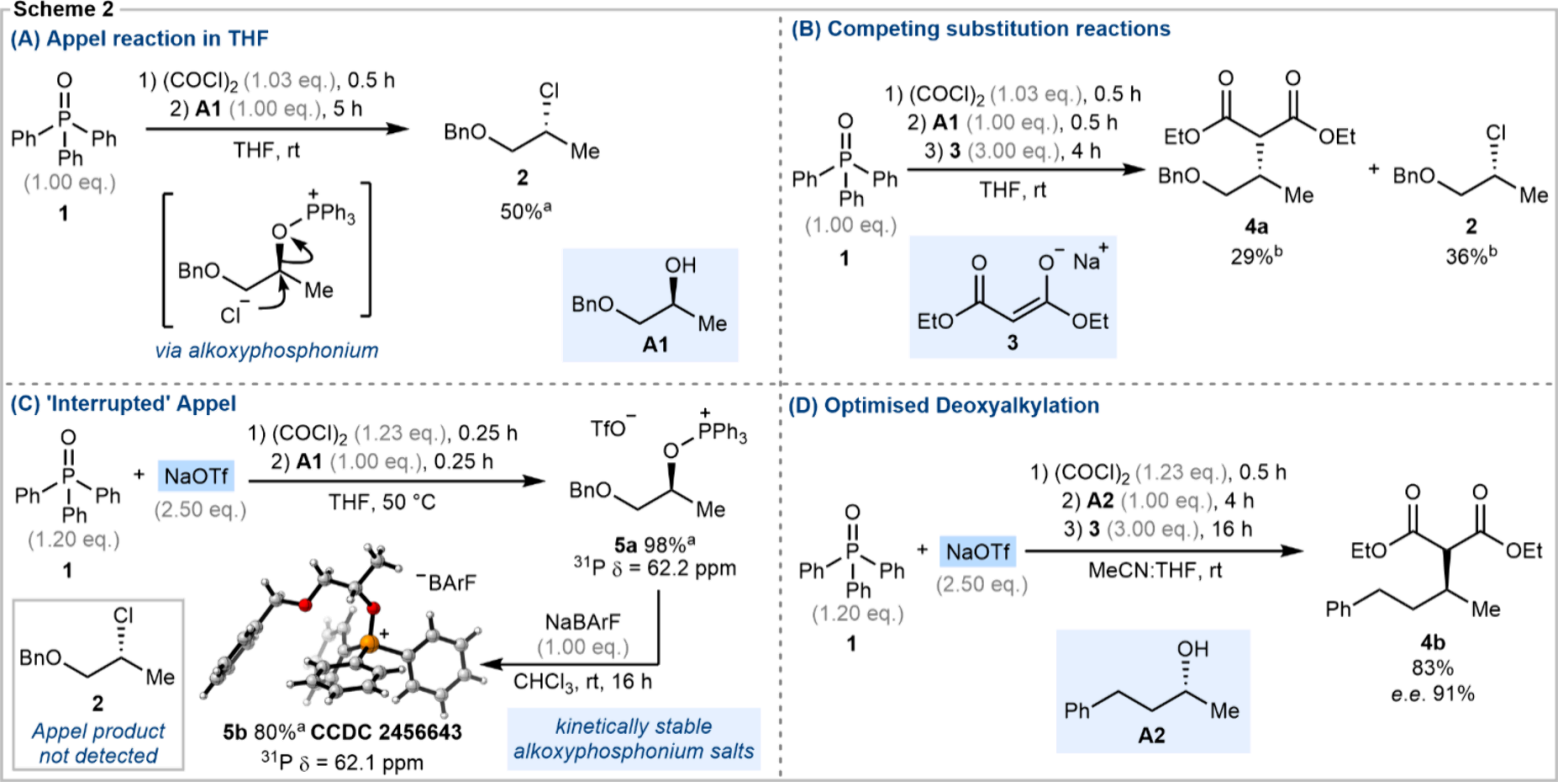
(A) Appel Reaction in THF; (B) Competing Alkylation and Appel Reactions; (C) Interrupted Appel Procedure, Yielding Kinetically Stable Alkoxyphosphonium Salts **5a** and **5b**; (D) Optimized Conditions for Deoxyalkylation

a Isolated yield shown.
b Yields determined by quantitative ^1^H NMR spectroscopy using a trimethoxybenzene standard.

A range of salts were screened including sodium triflate, silver triflate, sodium triflimide, and sodium tetraphenylborate, with varying degrees of success.^59^ Through these experiments we established that triphenylphosphine oxide activation using oxalyl chloride in the presence of 2.50 equiv of sodium triflate was remarkably effective, and we were able to prepare, isolate and characterize alkoxyphosphonium triflate **5a** from model alcohol **A1** (Scheme 2C). A further counterion metathesis reaction converted phosphonium triflate **5a** into the corresponding phosphonium borate **5b**, which was characterized by X-ray analysis (Scheme 2C). Due to the rapid coupling between the phosphonium cation and associated counteranion, most alkoxyphosphonium salts are challenging to observe and, to date, very few have been isolated and characterized.^48,60^ Our counterion metathesis protocol provides a simple method to access kinetically stable salts for reactions with nucleophiles. With an efficient metathesis and alcohol activation protocol in hand, we next conducted a series of optimization reactions^61^ using model alcohols **A1** and **A2** in combination with sodium diethylmalonate **3** under an anhydrous argon atmosphere. The optimized conditions are depicted in Scheme 2D for alcohol **A2**. Under these conditions, the competing Appel deoxychlorination is completely suppressed, and product **4b** was obtained in 83% yield and 91% *e*.*e*. in favor of the inverted stereoisomer.

We next explored the scope of the reaction (Scheme 3). In all cases, yields were determined after chromatographic purification and key findings were as follows. The transformation was shown to be compatible with a wide range of secondary alcohols, containing benzyl (**4a**), sulfone (**4d**), alkynyl (**4g**), furyl (**4h**) and silyl (**4i**) functionalities, while maintaining excellent enantiomeric excess in all cases. Both unfunctionalized acyclic (**4e**) and cyclic (**4f**) substrates performed effectively. Notably, homobenzylic substrates (**4c**, **4j**, **4o**) were also well tolerated, with no evidence of alkene formation. Primary alcohol substrates performed well, and amide (**4p**), ester (**4q**) and pyridyl (**4r**) containing alcohols were compatible in the coupling process. We next examined halogen-containing alcohols, and 5-chloro-1-pentanol underwent activation and coupling to give product **4m** without any competing nucleophilic substitution or elimination of chloride. The analogous reaction with 5-bromo-1-pentanol gave product **4n**, but in this instance, the dialkylated product was also observed because of competing nucleophilic substitution. In terms of unsuccessful substrates, sterically congested alcohols such as L-menthol as well as benzylic alcohols, including 1-indanol and 1-phenolethanol, underwent elimination in favor of the desired nucleophilic coupling.^59^

**Scheme 3:**
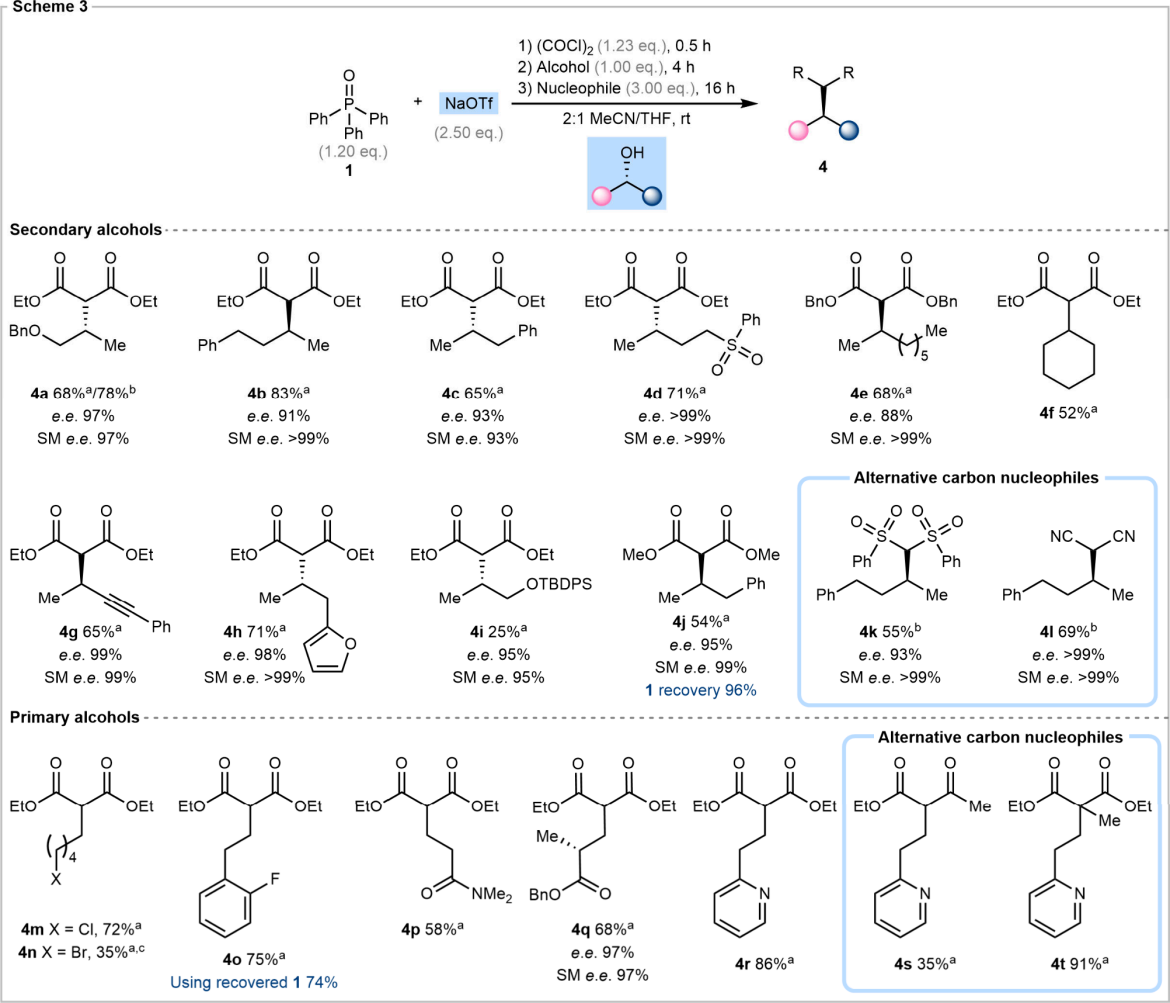
Stereospecific Deoxyalkylation Scope

a Standard reaction conditions: (i) triphenylphosphine oxide (1.20 mmol), sodium triflate (2.50 mmol), oxalyl chloride (1.23 mmol), THF (1 mL), MeCN (2 mL), rt, 0.5 h; (ii) alcohol (1.00 mmol), rt, 4 h; (iii) sodium diethylmalonate solution (0.95 M in THF, 3.00 mmol), rt, 16 h.
b Full experimental conditions including those used for alternative nucleophiles are provided in the Supporting Information.
c 14% of the dialkylated product was isolated.

We next examined the nucleophile scope and found that the reaction was not limited to unsubstituted malonates but is sensitive to the degree of stabilization of the carbanion. For example, potassium bis­(phenylsulfonyl)-methanide and sodium malononitrile performed well, giving rise to products **4k** and **4l** respectively. Sodium diethylmethylmalonate gave product **4t**, with a quaternary carbon, in excellent 91% yield. Less effective was sodium ethyl acetoacetate, which gave product **4s** in moderate yield. Unfortunately, attempts to use Grignard reagents as nucleophiles furnished unwanted Appel products, likely due to magnesium halides forming *in situ* as a result of the Schlenk equilibrium. Simple enolates, such as the lithium enolate of ethyl acetate, were also found to be unsuccessful.^59^

Finally, to establish the circular nature of the coupling protocol, product **4j** was prepared and isolated in 54% yield after chromatography along with 96% of analytically pure triphenylphosphine oxide. The isolated phosphine oxide was then used to prepare product **4o** which was obtained with no loss in isolated yield.

In summary, a stereospecific method for the deoxyalkylation of alcohols has been described. The reaction proceeds *via* an interrupted Appel reaction, allowing the formation of kinetically stable alkoxyphosphonium triflate salts *in situ*, which can undergo nucleophilic substitution with carbon nucleophiles to generate alkylated products in a stereocontrolled manner. Our methodology avoids a discrete activation step, *i*.*e*. the synthesis and isolation of alkyl halides or pseudohalides,^7^ as well as avoiding high energy diazodicarboxylate oxidants which are necessary for coupling in the conventional P­(III)/P­(V) Mitsunobu manifold.^16^ Structurally diverse primary and secondary alcohols are tolerated, including those containing amide, ester, and halogen functionalities, allowing for downstream functionalization.

## Supplementary Material



## Data Availability

The data underlying this study are available in the published article and its Supporting Information.

## Notes

Experimental procedures, compound characterization data, NMR spectra, HPLC and SFC chromatograms, and reaction optimization data (PDF)
