# Supplementary material for: Stereospecific Coupling of Alcohols and Carbanion Nucleophiles through a Circular P(V) Activation Manifold
Source: Org Lett. 2025 Dec 4;28(4):1115–20. doi: 10.1021/acs.orglett.5c04289 (PMC12865800; doi:10.1021/acs.orglett.5c04289)
Supplement: Supplementary file 1 [file ol5c04289_si_001.pdf]

Supplementary Information

Stereospecific Coupling of Alcohols and Carbanion Nucleophiles Through a Circular P(V) Activation Manifold

Isabel L. Wood, Stephen P. Argent and Ross M. Denton*
School of Chemistry, GlaxoSmithKline Carbon Neutral Laboratories for Sustainable Chemistry, University of Nottingham, 6 Triumph Road, Nottingham, NG7 2GA, UK. E-mail: ross.denton@nottingham.ac.uk.

Contents

General Information S3
Reagents and Solvents S3
Analysis and Characterisation S3
General Procedures S3
Preparation of sodium enolate solutions S3
Deoxyalkylation General Procedure A S3
Deoxyalkylation General Procedure B S3
Optimisation Studies S4
Counterion metathesis screening S4
Optimisation of chlorophosphonium salt formation S4
Optimisation of alkoxyphosphonium salt formation S5
Optimisation of the substitution reaction S6
Optimisation with alcohol A2 ..... S6
Preparation of Enantioenriched Alcohol Starting Materials S7
Malonate ester products (made from secondary alcohols) S12
Malonate ester products (made from primary alcohols) S17
Alternative nucleophile products S21
Assignment of Absolute Stereochemistry S23
Isolation of kinetically stable alkoxyphosphonium salts S25
Unsuccessful alcohols and nucleophiles S27
Preparation of Chiral HPLC Standards S27
Secondary alcohols S27
Primary Alcohols S29
Esters S30
Malonate ester products S31
Alternative nucleophile products S34
X-ray crystallography S35
HPLC & SFC Chromatograms S36
Alcohols S36
Esters S44
Alkyl chlorides S45
Products S46
Absolute Stereochemistry Standards S57
NMR spectra S59

Alcohol Starting Materials S59
Products (from secondary alcohols) S64
Products (from primary alcohols) S77
Other S87
References S93

General Information

Reagents and Solvents

All reactions were performed in conventional glassware. Unless otherwise indicated all reagents and solvents were purchased from commercial suppliers. Alcohol reagents were dried over calcium hydride and distilled or prepared by drying over molecular sieves (3 or 4 Å). Anhydrous solvents were obtained from in-house solvent purification systems (Inert® ProSolv; dried by passage through activated alumina columns under pressure of argon) or prepared by drying over molecular sieves (3 or 4 Å). Degassing of solvents was achieved by sparging with argon for 15 minutes. Reactions requiring inert conditions were conducted in dried glassware (dried by flame or heat gun) under an atmosphere of anhydrous argon or nitrogen using standard Schlenk techniques. Heated reactions were conducted using heating blocks, including Radleys Heat-On blocks or Asynt DrySyn heating blocks. When reactions were heated in microwave tubes, a Radleys Heat-On insert for 3×28mm Biotage Microwave Tubes (polymer coated) was used.

Analysis and Characterisation

Column chromatography was carried out using commercial silica gel 60A (40–63 mesh). Analytical TLC was performed on Merck aluminium-backed silica gel 60 F254 plates. Methods used to visualise compounds are specified in the experimental details below. NMR spectra were recorded at 298 K on Bruker Avance-III spectrometers, operating at nominal ^{1}H frequencies of 400 and 500 MHz, and internally referenced to residual solvent signals: $CDCl_3$ δ 7.26 (^{1}H) and 77.16 (^{13}C). Chemical shifts (δ) are reported in ppm, coupling constants (J) are reported in Hertz (Hz), heteronuclear coupling is indicated where relevant. External frequency standards are used for ^{31}P (85% H_3PO_4(aq); 0.00 ppm) and ^{19}F (CCl_3F; 0.00 ppm) spectroscopy. Signals are reported as singlet (s), doublet (d), triplet (t), quartet (q), pentet (p), heptet (h), multiplet (m), broad (br.), apparent (app.) or combinations thereof. IR spectra were obtained neat on a Bruker ALPHA FTIR spectrometer. HR-MS spectra were acquired on a Bruker MicrOTOF II ESI-TOF instrument. Melting points were determined using a Gallenkamp melting point apparatus. Chiral HPLC was carried out on an Agilent 1260 Infinity HPLC instrument. Chiral SFC analyses were carried out by Reach Separations (BioCity Nottingham, Pennyfoot Street, Nottingham, NG1 1GF, http://www.reachseparations.com).

General Procedures

Preparation of sodium enolate solutions

To a flame-dried vessel was added NaH (160 mg, 4 mmol of a 60 wt.% suspension in mineral oil), which was filled and evacuated with argon three times. Anhydrous THF (4 mL) was added, and the suspension was cooled to 0 °C. Anhydrous malonate ester or β-keto ester (4 mmol) was added dropwise (Caution: evolution of H_2 gas). After gas evolution ceased, the solution was warmed to room temperature to give a solution of sodium enolate in THF. Solutions were prepared and used within 2 hours.

This method was used to prepare sodium enolate solutions from diethylmalonate (to produce NaDEM **3**), dibenzylmalonate, dimethylmalonate, diethyl 2-methylmalonate and ethyl acetoacetate.

Deoxyalkylation General Procedure A

To a microwave tube was added anhydrous NaOTf (430 mg, 2.50 mmol, 2.50 equiv.) and anhydrous triphenylphosphine oxide **1** (334 mg, 1.20 mmol, 1.20 equiv.). The tube was then heated (heat gun) under high vacuum for 2.5 minutes, before filling with argon. The tube was evacuated and backfilled with argon a further two times. Anhydrous THF (1 mL) and MeCN (2 mL) were added, before oxalyl chloride (105 µL, 1.23 mmol, 1.23 equiv.) was added dropwise (Caution: slow addition due to evolution of CO_2 and CO gas). After stirring for 30 minutes at room temperature, anhydrous alcohol (1.00 mmol, 1.00 equiv.) was added, and the reaction was stirred for 4 hours at room temperature. After which time NaDEM **3** (3.16 mL, 3.00 mmol, 3.00 equiv. of a 0.950 M solution in THF) was added, and the reaction was stirred for 16 hours at room temperature. Upon completion, solvent was removed *in vacuo*, and crude mixture purified by column chromatography.

Deoxyalkylation General Procedure B

To a microwave tube was added anhydrous NaOTf (430 mg, 2.50 mmol, 2.50 equiv.) and anhydrous triphenylphosphine oxide **1** (334 mg, 1.20 mmol, 1.20 equiv.). The tube was then heated (heat gun) under high

vacuum for 2.5 minutes, before filling with argon. The tube was evacuated and backfilled with argon a further two times. Anhydrous THF (1 mL) and MeCN (2 mL) were added, before oxalyl chloride (105 µL, 1.23 mmol, 1.23 equiv.) was added dropwise (Caution: slow addition due to evolution of CO_2 and CO gas). After stirring for 30 minutes at room temperature, anhydrous alcohol (1.00 mmol, 1.00 equiv.) was added as a solution in anhydrous MeCN (0.2 mL), and the reaction was stirred for 4 hours at room temperature. After which time NaDEM **3** (3.16 mL, 3.00 mmol, 3.00 equiv. of a 0.950 M solution in THF) was added, and the reaction was stirred for 16 hours at room temperature. Upon completion, solvent was removed *in vacuo*, and crude mixture purified by column chromatography.

Optimisation Studies

Counterion metathesis screening

To a microwave tube was added the appropriate anhydrous salt (0.00 – 2.50 equiv.) and anhydrous triphenylphosphine oxide **1** (1.00 equiv.). The tube was then heated (heat gun) under high vacuum for 2.5 minutes, before filling with argon. The tube was evacuated and backfilled with argon a further two times. Anhydrous THF (3 mL) was added, and oxalyl chloride (1.03 equiv.) was added dropwise (Caution: slow addition due to evolution of CO_2 and CO gas). Once gas evolution ceased, anhydrous alcohol **A1** (1.00 equiv.) was added and the reaction was stirred for 3 hours at room temperature, after which time trimethoxybenzene (0.100 equiv.) was added. After a further 5 minutes an aliquot was taken and analysed by ^{1}H NMR spectroscopy.

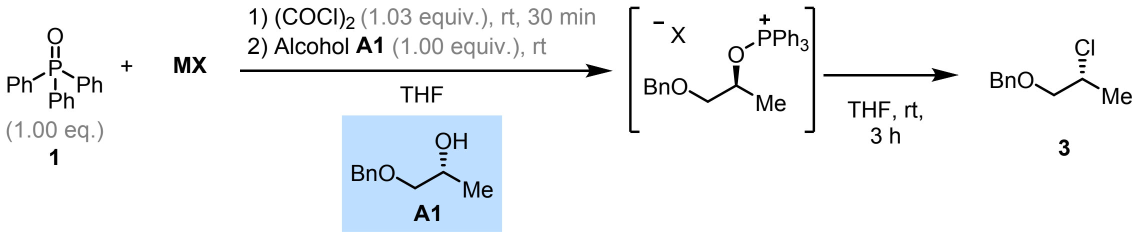

Entry	Salt (Equiv.)	Yield of 3 (%)*
1	-	54§
2	NaOTf (1.10)	11
**3**	**NaOTf (2.10)**	**0**
4	AgOTf (2.10)	0
5	$NaNTf_2$ (2.10)	0
6	$NaBPh_4$ (2.10)	14
7	$NaBF_4$ (2.10)	0†

*Table S1: Salt screening. *Yields determined by quantitative ^{1}H NMR spectroscopy using trimethoxybenzene standard. §After 5 hours. †Formed TPPO·BF_3 adduct by ^{31}P NMR spectroscopy.*

Optimisation of chlorophosphonium salt formation

To a microwave tube was added anhydrous NaOTf (2.10 – 2.50 equiv.) and anhydrous triphenylphosphine oxide **1** (1.00–1.20 equiv.). The tube was then heated (heat gun) under high vacuum for 2.5 minutes, before filling and evacuating the tube with argon three times. Anhydrous THF (3 mL) was added, and oxalyl chloride (1.03–1.23 equiv.) was added dropwise and the mixture was stirred (Caution: slow addition due to evolution of CO_2 and CO gas). If the reaction was heated, a heating block was used. An aliquot was taken and analysed by ^{31}P NMR spectroscopy.

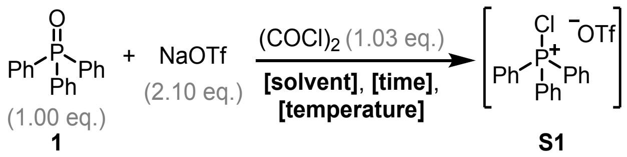

Entry	Deviation from standard conditions	Temp. (°C)	Time (min.)	S1 (%)*	1 (%)*
1	-	rt	5	78	22
2	-	rt	35	91	9
3	-	rt	1440	95	5
**4**	**2.30 equiv. NaOTf 1.10 equiv. $(COCl)_2$**	**rt**	**30**	**99**	**1**
5	-	50	10	91	9
6	-	50	45	91	9
7	1.20 equiv. TPPO, 2.50 equiv. NaOTf, 1.23 equiv. $(COCl)_2$	50	15	94	6

*Table S2: Optimisation of the formation of chlorotriphenylphosphonium triflate **S1**. *Based on ratio by ^{31}P spectroscopy.*

Optimisation of alkoxyphosphonium salt formation

To a microwave tube was added anhydrous NaOTf (2.10 – 2.50 equiv.) and anhydrous triphenylphosphine oxide **1** (1.00 – 1.20 equiv.). The tube was then heated (heat gun) under high vacuum for 2.5 minutes, before filling with argon. The tube was evacuated and backfilled with argon a further two times. Anhydrous THF (3 mL) was added, and oxalyl chloride (1.03 - 1.23 equiv.) was added dropwise (Caution: slow addition due to evolution of CO_2 and CO gas). Once gas evolution ceased, anhydrous alcohol **A1** (1.00 equiv.) was added, and the reaction was stirred. If the reaction was heated, a heating block was used. An aliquot was taken and analysed by ^{31}P NMR spectroscopy.

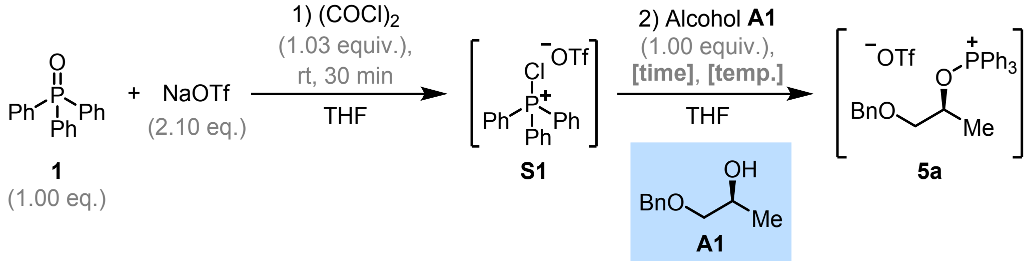

Entry	Deviation from standard conditions (Step 1)	Temp. (°C)	Time (min.)	5a (%)*	S1*	1*
1	-	rt	15	61 (61)	9	30
2	35 min.	rt	70	83 (83)	5	12
3	35 min.	rt	150	83 (84)	3	13
4	2.30 equiv. NaOTf 1.10 equiv. $(COCl)_2$, 30 min.	rt	70	79 (79)	11	10
5	2.30 equiv. NaOTf 1.10 equiv. $(COCl)_2$, 30 min.	rt	150	81 (81)	8	11

**6**	**1.20 equiv. TPPO, 2.50 equiv. NaOTf, 1.23 equiv. $(COCl)_2$, 15 min., 50 °C**	**50**	**15**	**81 (97)**	**8**	**11**
7	1.20 equiv. TPPO, 2.50 equiv. NaOTf, 1.23 equiv. $(COCl)_2$, 15 min., 50 °C	50	30	81 (97)	4	15

*Table S3: Optimisation of formation of alkoxytriphenylphosphonium triflate **5a**. *Yields (inside parentheses) and ratio of products (outside parentheses) determined by quantitative ^{31}P NMR spectroscopy.*

Optimisation of the substitution reaction

To a microwave tube was added anhydrous NaOTf (2.50 equiv.) and anhydrous triphenylphosphine oxide **1** (1.20 equiv.). The tube was then heated (heat gun) under high vacuum for 2.5 minutes, before filling with argon. The tube was evacuated and backfilled with argon a further two times. Anhydrous THF (3 mL) was added, and oxalyl chloride (1.23 equiv.) was added dropwise (Caution: slow addition due to evolution of CO_2 and CO gas). After stirring for 30 minutes at room temperature, anhydrous alcohol **A1** (1.00 equiv.) was added, and the reaction was stirred for 15 minutes at 50 °C using a heating block. The reaction was cooled to room temperature, NaDEM **3** (2.00 – 4.00 equiv. of a 0.950 M solution in THF) was added, and the reaction was stirred. Upon completion, solvent was removed *in vacuo*, and trimethoxybenzene (0.10 equiv.) and chloroform (20 mL) were added. After stirring for a further 5 minutes an aliquot was taken and analysed by ^{1}H NMR spectroscopy.

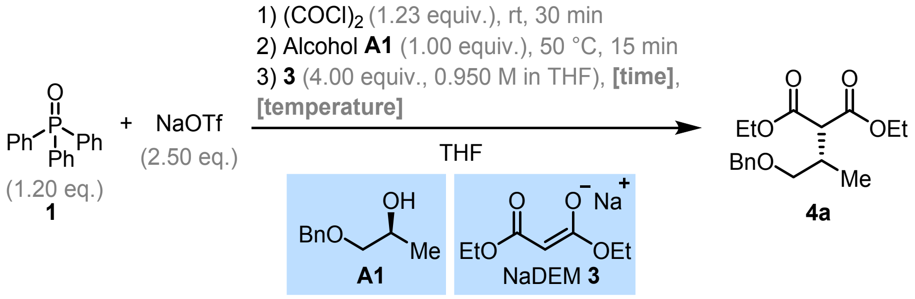

Entry	Deviation from standard conditions (Step 3)	Temp. (°C)	Time (h)	Yield of 4a (%)*	e.e. (%)
1	-	reflux	16	85 (78)	97
2	**3** (3.00 equiv.)	reflux	16	82	-
3	**3** (2.00 equiv.)	reflux	16	63	-
4	**3** (3.00 equiv.)	reflux	5	85	-
**5**	**3 (3.00 equiv.)**	**rt**	**16**	**84**	**-**
6	**3** (3.00 equiv.)	rt	5	68	-

*Table S4: Optimisation of the substitution step (step 3). *^{1}H NMR yield determined using trimethoxybenzene as an internal standard. Isolated yield shown in brackets.*

Optimisation with alcohol A2

To a microwave tube was added anhydrous NaOTf (2.50 equiv.) and anhydrous triphenylphosphine oxide **1** (1.20 equiv.). The tube was then heated (heat gun) under high vacuum for 2.5 minutes, before filling with argon. The tube was evacuated and backfilled with argon a further two times. Anhydrous THF (3 mL) was added, and oxalyl chloride (1.23 equiv.) was added dropwise (Caution: slow addition due to evolution of CO_2 and CO gas). After stirring for 30 minutes at room temperature, anhydrous alcohol **A2** (156 μL, 1.00 mmol) was added, and the reaction was stirred. After which time NaDEM **3** (3.00 equiv. of a 0.950 M solution in THF) was added, and the reaction was stirred at room temperature for 16 h. Upon completion, solvent was removed *in vacuo*, and the product was purified by column chromatography to afford **4b**.

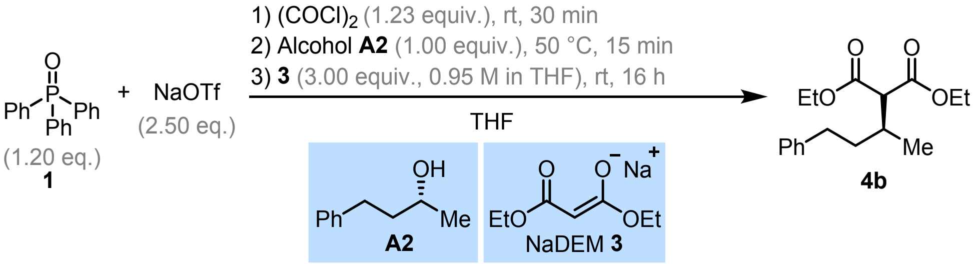

Entry	Deviation from standard conditions	Yield of 4b (%)*	e.e. of 4b (%)
1	-	25	92
2	MeCN; Step 2: rt, 4 h.	77	83
**3**	**2:1 MeCN:THF; Step 2: rt, 4 h**	**83**	**91**

*Table S5: Optimisation with alcohol **A2**. *Yields inside parentheses are yields determined by quantitative ^{1}H NMR spectropscopy using trimethoxybenzene as an internal standard.*

Preparation of Enantioenriched Alcohol Starting Materials

(*S*)-2-octanol (S2)

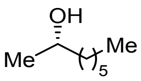

HPLC: The enantiomeric excess (>99%) was determined by chiral HPLC analysis of 4-nitrobenzyl esters **S24** and **S25**.

(*R*)-(−)-1-Phenyl-2-propanol (S3)

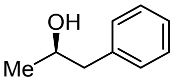

HPLC: The enantiomeric excess (93%) was determined by chiral HPLC analysis. (Daicel Chiralcel OD-H, IPA/isohexane = 5/95, flow rate = 1.0 mL/min, l = 210 nm) t_R = 7.46 min (minor), 8.25 min (major).

(*S*)-(−)-1-Phenyl-2-propanol (S4)

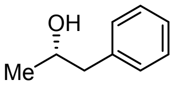

HPLC: The enantiomeric excess (99%) was determined by chiral HPLC analysis. (Daicel Chiralcel OD-H, IPA/isohexane = 5/95, flow rate = 1.0 mL/min, l = 210 nm) t_R = 7.47 min (major), 8.30 min (minor).

(*R*)-4-Phenylbutan-2-ol (A2)

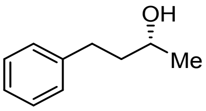

HPLC: The enantiomeric excess (>99%) was determined by chiral HPLC analysis. (Daicel Chiralcel OD-H, IPA/isohexane = 10/90, flow rate = 1.0 mL/min, l = 210 nm) t_R = 6.72 min (major), minor not observed.

(*S*)-1-(benzyloxy)propan-2-ol (A1)

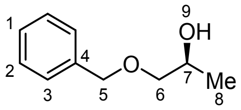

To a solution of (*S*)-benzyl glycidyl ether (10.0 mL, 59.8 mmol) in THF (300 mL) was added $LiAlH_4$ (33.0 mL, 66.0 mmol of a 2.00 M solution in THF) in 10 portions at 0 °C, under an argon atmosphere and the reaction mixture was stirred at 0 °C for 1 hour and 15 minutes. The reaction was then diluted with Et_2O (300 mL) and cooled to 0 °C before H_2O (2.50 mL) was added dropwise, followed by NaOH (2.50 mL of a 15% w/v aqueous solution), then H_2O (7.50 mL) (Caution: formation of H_2 gas). After gas evolution was complete, the mixture was warmed to room temperature and stirred for 15 minutes, after which $MgSO_4$ (15 g) was added and the reaction mixture stirred for a further 15 minutes, then filtered, eluting with Et_2O. The solvent was removed *in vacuo* to afford the title compound as a colourless liquid (9.17 g, 92%, 97% *e.e.*).

^{1}H NMR (500 MHz, $CDCl_3$) δ 7.39 – 7.28 (m, 5H, H-1, H-2, H-3) 4.56 (s, 2H, H-5), 4.05 – 3.96 (m, 1H, H-7), 3.48 (dd, J = 9.4, 3.1 Hz, 1H, H-6a), 3.29 (dd, J = 9.4, 8.1 Hz, 1H, H-6b), 2.37 (d, J = 3.0 Hz, 1H, H-9), 1.15 (d, J = 6.4 Hz, 3H, H-8).

^{13}C{^{1}H} NMR (126 MHz, $CDCl_3$) δ 138.1 (C-4), 128.6 (C-2), 127.90 (C-1), 127.86 (C-3), 75.9 (C-6), 73.4 (C-5), 66.6 (C-7), 18.8 (C-8).

IR (ATR, neat) (ν_{max} /cm^{-1}): 3422, 3030, 2970, 2929.

HRMS: (ESI) m/z: $[M + Na]^+$ Calcd for $NaC_{10}H_{14}O_2^+$ 189.0886; Found 189.0889.

HPLC: The enantiomeric excess (97%) was determined by chiral HPLC analysis. (Daicel Chiralcel AS-H, IPA/isohexane = 5/95, flow rate = 1.0 mL/min, l = 210 nm) t_R = 9.42 min (major), 11.60 min (minor).

TLC: R_f 0.27 (6:4 pentane:Et_2O).

OR: $[\alpha]_D^{25}$: +12.0 (c. 1.0, $CHCl_3$)

Data in agreement with literature.[1, 2]

(*R*)-(−)-4-(phenylsulfonyl)-2-butanol (S6)

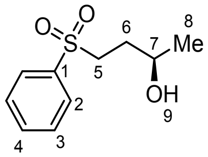

To a solution of methyl phenyl sulfone (781 mg, 5.00 mmol), in THF (6 mL) was added *n*-BuLi (3.13 mL, 5.00 mmol of a 1.60 M solution in hexanes) dropwise at −78 °C. The reaction mixture was stirred at −78 °C for 30 minutes. (*R*)-(+)-propylene oxide (524 µL, 7.50 mmol) was added dropwise. The reaction mixture was then warmed to room temperature and stirred for 14 hours and 30 minutes, after which the reaction was quenched with NH_4Cl (10 mL of a saturated aqueous solution) at 0 °C and extracted with CH_2Cl_2 (3 × 20 mL). The combined organic fractions were washed with NH_4Cl (40 mL of a saturated aqueous solution), then brine (40 mL of a saturated aqueous solution), dried over $MgSO_4$ and concentrated. The crude residue was purified by flash column chromatography (SiO_2, 1:9 CH_2Cl_2:Et_2O) to afford the title compound as a colourless oil, which solidified on standing. (857 mg, 80%, *e.e.* >99%).

^{1}H NMR (400 MHz, $CDCl_3$) δ 7.96 – 7.89 (m, 2H, H-2 or H-3), 7.70 – 7.62 (m, 1H, H-4), 7.62 – 7.54 (m, 2H, H-2 or H-3), 3.99 – 3.85 (m, 1H, H-7), 3.35 – 3.26 (m, 1H, H-5a), 3.26 – 3.14 (m, 1H, H-5b), 2.03 – 1.88 (m, 1H, H-6a), 1.86 – 1.70 (m, 1H, H-6b), 1.50 (d, J = 5.1 Hz, 1H, H-9), 1.22 (d, J = 6.2 Hz, 3H, H-8).

$^{13}C\{^{1}H\}$ NMR (126 MHz, $CDCl_3$) δ 139.3 (C-1), 133.9 (C-4), 129.5 (C-2 or C-3), 128.2 (C-2 or C-3), 66.3 (C-7), 53.3 (C-5), 31.7 (C-6), 23.8 (C-8).

IR (ATR, neat) (ν_{max} /cm^{-1}): 3482, 2971, 2927.

HRMS: (ESI) m/z: $[M + H]^+$ Calcd for $C_{10}H_{15}O_3S^+$ 215.0736; Found 215.0734.

HPLC: The enantiomeric excess (>99%) was determined by chiral HPLC analysis. (Daicel Chiralcel OD-H, IPA/isohexane = 10/90, flow rate = 1.0 mL/min, l = 210 nm) t_R = 20.89 min (minor), 23.63 min (major).

TLC: R_f 0.25 (9:1 Et_2O:CH_2Cl_2)

OR: $[\alpha]_D^{25}$: –24.0 (c. 1.0, $CHCl_3$)

Data in agreement with literature.[2]

(*R*)-1-((tert-butyldiphenylsilyl)oxy)propan-2-ol (S7)

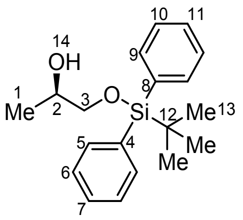

To a solution of (*R*)-1,2-propane diol (367 µL, 5.00 mmol) and imidazole (781 mg, 6.50 mmol) in CH_2Cl_2 (25.0 mL) at 0 °C was added *tert*-butyldiphenylchlorosilane (1.30 mL, 5.00 mmol) dropwise. The reaction mixture was slowly warmed to room temperature and stirred at room temperature for 16 hours. $NaHCO_3$ was added (10 mL of a saturated aqueous solution) and the mixture was vigorously stirred for 1 minute, before extracting with CH_2Cl_2 (3 × 15 mL). The combined organic fractions were washed with brine (20 mL of a saturated aqueous solution), dried over $MgSO_4$ and concentrated. The crude residue was purified by flash column chromatography (SiO_2, 1:9 Et_2O:pentane) to afford the title compound as a colourless oil that solidified on standing. (1.29 g, 82%, *e.e.* 95%).

^{1}H NMR (400 MHz, $CDCl_3$): δ 7.71 – 7.62 (m, 4H, H-5, H-9), 7.48 – 7.35 (m, 6H, H-6, H-10, H-7, H-11), 3.98 – 3.85 (m, 1H, H-2), 3.63 (dd, J = 10.1, 3.4 Hz, 1H, H-3a), 3.45 (dd, J = 10.1, 7.8 Hz, 1H, H-3b), 2.56 – 2.53 (m, 1H, H-14), 1.10 (d, J = 6.3 Hz, 3H, H-1), 1.07 (s, 9H, H-13).

$^{13}C\{^{1}H\}$ NMR (101 MHz, $CDCl_3$) : δ 135.69 (C-5 or C-9), 135.67 (C-5 or C-9), 133.4 (C-4 or C-8), 133.3 (C-4 or C-8), 123.0 (C-7, C-11), 127.9 (C-6, C-10), 69.5 (C-3), 68.2 (C-2), 27.0 (C-13), 19.4 (C-12), 18.4 (C-1).

IR (ATR, neat) (ν_{max} /cm^{-1}): 3582, 3411, 3071, 3049.

HRMS: (ESI) m/z: $[M + Na]^+$ Calcd for $NaC_{19}H_{26}O_2Si^+$ 337.1595; Found 337.1598.

HPLC: The enantiomeric excess (95%) was determined by chiral HPLC analysis. (Daicel Chiralcel OD-H, IPA/isohexane = 10/90, flow rate = 1.0 mL/min, l = 210 nm) t_R = 4.42 min (major), 4.91 min (minor).

TLC: R_f 0.28 (9:1 pentane:Et_2O)

OR: $[\alpha]_D^{25}$: –12.0 (c. 1.0, $CHCl_3$)

m.p.: 46 – 48 °C.

Data in agreement with literature.[3]

(*R*)-Benzyl 3-hydroxy-2-methylpropanoate (S8)

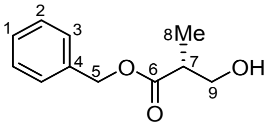

To a solution of methyl (*R*)-(−)-3-hydroxy-2-methylpropionate (1.10 mL, 10.0 mmol) in MeOH (10.0 mL), was added NaOH (3.40 mL, 10.2 mmol, 3.00 M solution in H_2O) and the reaction was stirred at room temperature for 19 hours. Volatiles were removed *in vacuo* to result a crude residue. The vessel was evacuated and backfilled with argon three times, anhydrous DMF (10 mL) was added, and benzyl bromide (1.21 mL, 10.2 mmol) was added in 5 portions. The resulting mixture was heated to 80 °C using a heating block and stirred for 24 hours. The reaction mixture was diluted with EtOAc (20 mL) and extracted with H_2O (10 mL × 3) and brine (10 mL of a saturated aqueous solution). The organics were dried over $MgSO_4$ and concentrated. The crude residue was purified by flash column chromatography (SiO_2, 1:1 EtOAc:cyclohexane) to afford the title compound as a colourless oil. (904 mg, 47%, *e.e.* 97%).

^{1}H NMR (400 MHz, $CDCl_3$) δ 7.51 – 7.30 (m, 5H, H-1, H-2, H-3), 5.16 (s, 2H, H-5), 3.85 – 3.62 (m, 2H, H-9), 2.84 – 2.64 (m, 1H, H-7), 1.20 (d, J = 7.2 Hz, 3H, H-8).

^{13}C{^{1}H} NMR (101 MHz, $CDCl_3$) δ 175.6 (C-6), 135.9 (C-4), 128.8 (C-2), 128.5 (C-1), 128.2 (C-3), 66.6 (C-5), 64.7 (C-9), 41.9 (C-7), 13.6 (C-8).

IR (ATR, neat) (v_{max} /cm^{-1}): 2976, 2940, 2881, 1734, 1709.

HRMS: (ESI) m/z: $[M + Na]^+$ Calcd for $NaC_{11}H_{14}O_3^+$ 217.0836; Found 217.0835.

HPLC: The enantiomeric excess (97%) was determined by chiral HPLC analysis. (Daicel Chiralcel OD-H, IPA/isohexane = 10/90, flow rate = 1.0 mL/min, l = 210 nm) t_R = 8.26 min (major), 8.98 min (minor).

TLC: R_f 0.24 (1:1 EtOAc:cyclohexane)

OR: $[\alpha]_D^{25}$: –20.0 (c. 1.0, $CHCl_3$)

Data in agreement with literature.[4]

(*S*)-Benzyl 3-hydroxy-2-methylpropanoate (S9)

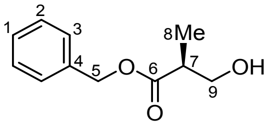

To a solution of methyl (*S*)-(−)-3-hydroxy-2-methylpropionate (552 µL, 5.00 mmol) in MeOH (5 mL), was added NaOH (1.70 mL, 5.10 mmol, 3.00 M solution in H_2O) and the reaction was stirred at room temperature for 19 hours. Volatiles were removed *in vacuo* to result a crude residue. The vessel was evacuated and backfilled with argon three times, after which anhydrous DMF (5 mL) was added followed by benzyl bromide (606 µL, 5.10 mmol) in 5 portions. The resulting mixture was heated to 80 °C using a heating block and stirred for 24 hours. The reaction mixture was diluted with EtOAc (10 mL) and extracted with H_2O (3 × 5 mL) and brine (5 mL of a saturated aqueous solution). The organics were dried over $MgSO_4$ and concentrated. The crude residue was purified by flash column chromatography (SiO_2, 1:1 EtOAc:cyclohexane) to afford the title compound as a colourless oil. (588 mg, 61%, *e.e.* 90%).

^{1}H NMR, ^{13}C{1H} NMR, IR, HRMS and R_f match compound S8.

HPLC: The enantiomeric excess (90%) was determined by chiral HPLC analysis. (Daicel Chiralcel OD-H, IPA/isohexane = 10/90, flow rate = 1.0 mL/min, l = 210 nm) t_R = 8.07 min (major), 8.96 min (major).

OR: $[\alpha]_D^{25}$: +20.0 (c. 1.0, $CHCl_3$)

Data in agreement with literature.[5]

(*S*)-2-(2-hydroxypropyl)furan (S10)

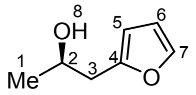

Prepared following literature method.[6]

To a solution of furan (727 µL, 10.0 mmol) in anhydrous THF (25 mL) at −78 °C under an atmosphere of argon was added *n*-BuLi (6.25 mL, 10.0 mmol of a 1.60 M solution in hexanes) dropwise. After stirring for 30 minutes, (*R*)-propylene oxide (840 µL, 12.0 mmol) was added. The reaction was warmed to room temperature and left to stir for 16 hours. The reaction was then quenched with NH_4Cl (16 mL of a saturated aqueous solution) and the aqueous phase extracted with ethyl acetate (2 × 15 mL). The organics were washed with brine (10 mL of a saturated aqueous solution), dried over $MgSO_4$, filtered and solvents were removed *in vacuo*. The crude product was purified by flash column chromatography (SiO_2, 1:3 Et_2O:pentane) to afford the title compound as a yellow oil. (612 mg, 49%, e.e. >99%).

^{1}H NMR (400 MHz, $CDCl_3$) δ 7.38 – 7.30 (m, 1H, H-7), 6.34 – 6.29 (m, 1H, H-6), 6.14 – 6.07 (m, 1H, H-5), 4.16 – 4.03 (m, 1H, H-2), 2.85 – 2.70 (m, 2H, H-3), 1.81 – 1.74 (m, 1H, H-8), 1.24 (d, J = 6.2 Hz, 3H, H-1).

^{13}C{^{1}H} NMR (101 MHz, $CDCl_3$) δ 152.6 (C-4), 141.7 (C-7), 110.3 (C-6), 107.0 (C-5), 66.8 (C-2), 37.8 (C-3), 22.7 (C-1).

IR (ATR, neat) (v_{max} /cm^{-1}): 2970, 1597, 1507.

HRMS: (ESI) m/z: $[M + Na]^+$ Calcd for $NaC_7H_{10}O_2^+$ 149.0573; Found 149.0560.

HPLC: The enantiomeric excess (>99%) was determined by chiral HPLC analysis. (Lux A2, MeCN/water (0.1% v/v TFA) = 5/95, flow rate = 1.0 mL/min, l = 210 nm) t_R = 12.93 min (major), minor not observed.

TLC: R_f 0.087 (3:1 pentane:Et_2O).

OR: $[\alpha]_D^{25}$: –36.0 (c. 1.0, $CHCl_3$)

Data in agreement with literature.[7]

(*S*)-5-Phenylpent-4-yn-2-ol (S11)

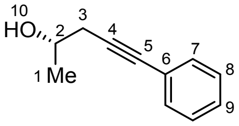

Prepared following literature method.[8]

To a stirred solution of phenylacetylene (1.10 mL, 10.0 mmol) in anhydrous THF (25 mL) at −78 °C was added dropwise *n*-BuLi (6.88 mL, 11.0 mmol of a 1.60 M solution in hexanes). After stirring for 1 hour at −78 °C, $BF_3·OEt_2$ (1.48 mL, 12.0 mmol) was added dropwise and the stirring continued for another 15 minutes before (*S*)-propylene oxide (1.05 mL, 15.0 mmol) was added dropwise at −78 °C. Stirring was continued for 3 hours at −78 °C, then the reaction was quenched with NH_4Cl (10 mL of a saturated aqueous solution). The mixture was extracted with ether (3 × 15 mL), then the combined organic layers were washed with saturated brine (15 mL of a saturated aqueous solution), dried over sodium sulfate, then the solvent removed *in vacuo*. The residues were purified by flash chromatography (SiO_2, 1:4 Et_2O:pentane) to afford the title compound as a yellow oil. (612 mg, 49%, *e.e.* 99%).

^{1}H NMR (400 MHz, $CDCl_3$) δ 7.45 – 7.38 (m, 2H, H-7 or H-8), 7.33 – 7.27 (m, 3H, H-7 or H-8, H-9), 4.12 – 3.99 (m, 1H, H-2), 2.67 – 2.52 (m, 2H, H-3), 1.89 (br s, 1H, H-10), 1.33 (d, J = 6.2 Hz, 3H, H-1).

^{13}C{^{1}H} NMR (101 MHz, $CDCl_3$) δ 131.7 (C-7 or C-8), 128.3 (C-7 or C-8), 128.0 (C-9), 123.4 (C-6), 86.1 (C-4 or C-5), 83.1 (C-4 or C-5), 66.6 (C-2), 30.1 (C-3), 22.4 (C-1).

IR (ATR, neat) (v_{max} /cm^{-1}): 3404, 3053, 2976, 2933.

HRMS: (ESI) m/z: [M + Na]$^+$ Calcd for $NaC_{11}H_{12}O^+$ 183.0781; Found 183.0777.

HPLC: The enantiomeric excess (99%) was determined by chiral HPLC analysis. (Daicel Chiralcel OD-H, IPA/isohexane = 10/90, flow rate = 1.0 mL/min, l = 210 nm) t_R = 9.90 min (major), 15.41 min (minor).

TLC: R_f 0.12 (4:1 pentane:Et_2O)

OR: $[\alpha]_D^{25}$: –20.0 (c. 1.0, $CHCl_3$)

Data in agreement with literature.[9]

Malonate ester products (made from secondary alcohols)

(*S*)-Diethyl 2-(1-(benzyloxy)propan-2-yl)malonate (4a)

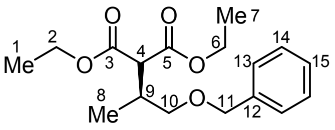

Following general procedure A. Purification by flash chromatography (SiO_2, 95:5 pentane:Et_2O) gave a mixture of diethylmalonate and the title compound. Residual diethylmalonate was removed by heating to 50 °C under high vacuum, using a heating block, resulting the title compound as a light-yellow liquid (210 mg, 0.680 mmol, 68%, 97% e.e.).

Alternative method: To a microwave tube was added anhydrous NaOTf (430 mg, 2.50 mmol) and anhydrous triphenylphosphine oxide **1** (334 mg, 1.20 mmol), then heated (heat gun) under high vacuum for 2.5 minutes, before filling with argon. The tube was evacuated and backfilled with argon a further two times. Oxalyl chloride (105 µL, 1.23 mmol) was added dropwise (Caution: evolution of CO_2 and CO gas) and then the mixture was stirred at room temperature for 30 minutes. Anhydrous (*S*)-1-(benzyloxy)propan-2-ol (154 µL, 1.00 mmol) was added, and the mixture was stirred at 50 °C for 15 minutes using a heating block. NaDEM **3** (3.16 mL, 3.00 mmol of a 0.950 M solution in THF) was added, and the reaction was stirred at room temperature for 16 hours. The solvent was removed *in vacuo*. Purification by flash chromatography (SiO_2, 95:5 pentane:Et_2O) gave a mixture of diethylmalonate and the title compound. Residual diethylmalonate was removed by heating to 50 °C under high vacuum, using a heating block, resulting the title compound as a light-yellow liquid (241 mg, 0.780 mmol, 78%, 97% *e.e.*).

^{1}H NMR (400 MHz, $CDCl_3$): δ 7.37 – 7.26 (m, 5H, H-13, H-14, H-15), 4.48 (s, 2H, H-11), 4.23 – 4.08 (m, 4H, H-2, H-6), 3.51 (d, J = 7.6 Hz, 1H, H-4), 3.48 – 3.40 (m, 2H, H-10), 2.64 – 2.52 (m, 1H, H-9), 1.25 (t, J = 7.1 Hz, 3H, H-1 or H-7), 1.23 (t, J = 7.1 Hz, 3H, H-1 or H-7), 1.05 (d, J = 6.9 Hz, 3H, H-8).

^{13}C{^{1}H} NMR (126 MHz, $CDCl_3$): δ 169.1 (C-3 or C-5), 168.9 (C-3 or C-5), 138.5 (C-12), 128.5 (C-14), 127.74 (C-13), 127.67 (C-15), 73.2 (C-11), 73.0 (C-10), 61.3 (C-2 or C-6), 61.3 (C-2 or C-6), 54.4 (C-4), 34.1 (C-9), 14.9 (C-8), 14.3 (C-1 or C-7), 14.2 (C-1 or C-7).

IR (ATR, neat) (v_{max} /cm^{-1}): 2980, 2937,1748, 1728, 1454.

HRMS: (ESI) m/z: [M + Na]$^+$ Calcd for $NaC_{15}H_{28}O_4^+$ 331.1516; Found 331.1514.

HPLC: The enantiomeric excess (97%) was determined by chiral HPLC analysis. (Daicel Chiralcel IC, IPA/isohexane = 1/99, flow rate = 1.0 mL/min, l = 210 nm) t_R = 22.66 min (minor), 24.37 min (major).

TLC: R_f 0.15 (pentane:Et_2O 95:5)

OR: $[\alpha]_D^{25}$: –20.0 (c. 1.0, $CHCl_3$)

^{1}H, ^{13}C, IR and HR-MS match racemic compound.[10]

(*S*)-Diethyl 2-(4-phenylbutan-2-yl)malonate (4b)

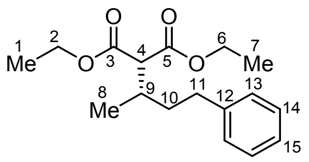

Following general procedure A. Purification by flash chromatography (SiO_2, 95:5 pentane:Et_2O) afforded title compound as a colourless liquid (241.3 mg, 0.830 mmol, 83%, 91% *e.e.*).

^{1}H NMR (400 MHz, $CDCl_3$): δ 7.31 – 7.24 (m, 2H, H-13 or H-14), 7.20 – 7.13 (m, 3H, H-15, H-13 or H-14), 4.26 – 4.12 (m, 4H, H-2, H-6), 3.29 (d, *J* = 7.9 Hz, 1H, H-4), 2.78 – 2.66 (m, 1H, H-11a), 2.64 – 2.52 (m, 1H, H-11b), 2.39 – 2.24 (m, 1H, H-9), 1.84 – 1.71 (m, 1H, H-10a), 1.61 – 1.47 (m, 1H, H-10b), 1.27 (t, *J* = 7.2 Hz, 3H, H-1 or H-7), 1.25 (t, *J* = 7.1 Hz, 3H, H-1 or H-7), 1.07 (d, *J* = 6.8 Hz, 3H, H-8).

^{13}C{^{1}H} NMR (101 MHz, $CDCl_3$): δ 168.9 (C-3 or C-5), 168.9 (C-3 or C-5), 142.2 (C-12), 128.5 (C-13 or C-14), 128.3 (C-13 or C-14), 126.0 (C-15), 61.34 (C-2 or C-6), 61.29 (C-2 or C-6), 57.8 (C-4), 36.4 (C-10), 33.4 (C-11), 33.3 (C-9), 17.1 (C-8), 14.28 (C-1 or C-7), 14.25 (C-1 or C-7).

IR (ATR, neat) (ν_{max} /cm^{-1}): 3028, 2981, 1754, 1725.

HRMS: (ESI) m/z: $[M + Na]^+$ Calcd for $NaC_{17}H_{24}O_4^+$ 315.1567; Found 315.1569.

HPLC: The enantiomeric excess (91%) was determined by chiral HPLC analysis. (Daicel Chiralcel IC, IPA/isohexane = 5/95, flow rate = 1.0 mL/min, l = 210 nm) t_R = 6.61 min (minor), 7.06 min (major).

TLC: R_f 0.29 (pentane:Et_2O 95:5).

OR: $[\alpha]_D^{25}$: –4.0 (c. 1.0, $CHCl_3$)

Data in agreement with literature.[11]

(*S*)-Diethyl 2-(1-phenylpropan-2-yl)malonate (4c)

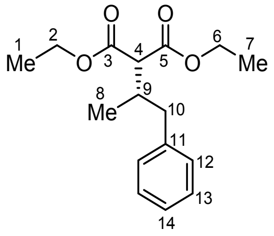

Following general procedure A. Purification by flash chromatography (SiO_2, 95:5 pentane:Et_2O) afforded title compound as a colourless liquid (182 mg, 0.650 mmol, 65%, 93% *e.e.*).

^{1}H NMR (500 MHz, $CDCl_3$): δ 7.32 – 7.26 (m, 2H, H-13), δ 7.23 – 7.16 (m, 3H, H-12, H-14), 4.26 – 4.16 (m, 4H, H-2, H-6), 3.28 (d, *J* = 7.4 Hz, 1H, H-4), 2.83 (dd, *J* = 13.2, 5.0 Hz, 1H, H-10a), 2.62 – 2.48 (m, 1H, H-9), 2.43 (dd, *J* = 13.2, 9.1 Hz, 1H, H-10b), 1.32 – 1.25 (m, 6H, H-1, H-7), 0.95 (d, *J* = 6.7 Hz, 3H, H-8).

^{13}C{^{1}H} NMR (126 MHz, $CDCl_3$): δ 169.1 (C-3 or C-5), 168.8 (C-3 or C-5), 140.0 (C-11), 129.4 (C-12), 128.5 (C-13), 126.3 (C-14), 61.4 (C-2 or C-6), 61.3 (C-2 or C-6), 57.1 (C-4), 40.8 (C-10), 35.6 (C-9), 16.9 (C-8), 14.30 (C-1 or C-7), 14.27 (C-1 or C-7).

IR (ATR, neat) (ν_{max} /cm^{-1}): 3063, 3028, 1752, 1724, 1496.

HRMS: (ESI) m/z: $[M + Na]^+$ Calcd for $NaC_{16}H_{22}O_4^+$ 301.1411; Found 301.1414.

HPLC: The enantiomeric excess (93%) was determined by chiral HPLC analysis. (Daicel Chiralcel IC, IPA/isohexane = 2/98, flow rate = 1.0 mL/min, l = 210 nm) t_R = 8.27 min (minor), 8.96 min (major).

TLC: R_f 0.11 (95:5 pentane:Et_2O).

OR: $[\alpha]_D^{25}$: +12.0 (c. 1.0, $CHCl_3$)

^1H and ^{13}C NMR data are consistent with the literature.[12]

(*R*)-Dibenzyl 2-(octan-2-yl)malonate (4e)

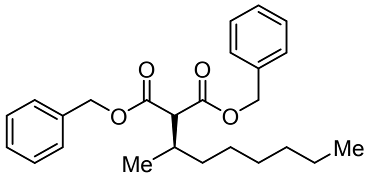

Following general procedure A, using a solution of sodium dibenzylmalonate in THF as the nucleophile. Purification by flash chromatography (SiO_2, 95:5 pentane:Et_2O) afforded title compound as a colourless liquid (270 mg, 0.680 mmol, 68%, 88% e.e.).

^1H NMR (500 MHz, $CDCl_3$): δ 7.36 – 7.27 (m, 10H, ArH × 10), 5.14 (s, 4H, CH_2 × 2), 3.35 (d, *J* = 8.0 Hz, 1H, CH), 2.34 – 2.17 (m, 1H, CH), 1.40 – 1.06 (m, 10H, CH_2 × 5), 0.95 (d, *J* = 6.8 Hz, 3H, CH_3), 0.86 (t, *J* = 7.1 Hz, 3H, CH_3).

$^{13}C\{^1H\}$ NMR (126 MHz, $CDCl_3$): δ 168.9 (C_q), 168.7 (C_q), 135.6 (C_q × 2), 128.7 (CH × 4), 128.43 (CH × 2), 128.38 (CH × 4), 67.03 (CH_2), 67.00 (CH_2), 57.9 (CH), 34.45 (CH_2), 33.8 (CH), 31.9 (CH_2), 29.4 (CH_2), 26.9 (CH_2), 22.8 (CH_2), 17.1 (CH_3), 14.2 (CH_3).

IR (ATR, neat) (ν_{max} /cm^{-1}): 3067, 3034, 2958, 1756, 1729, 1498.

HRMS: (ESI) m/z: $[M + Na]^+$ Calcd for $NaC_{25}H_{32}O_4^+$ 419.2193; Found 419.2190.

SFC: The enantiomeric excess (88%) was determined by chiral SFC analysis. (Chiralpak IG, IPA/CO_2 = 10/90, flow rate = 3.0 mL/min, l = 211 nm) t_R = 4.47 min (minor), 4.95 min (major).

TLC: R_f 0.25 (95:5 pentane:Et_2O).

OR: $[\alpha]_D^{25}$: +4.0 (c. 1.0, $CHCl_3$)

(*R*)-Diethyl 2-(1-((tert-butyldiphenylsilyl)oxy)propan-2-yl)malonate (4i)

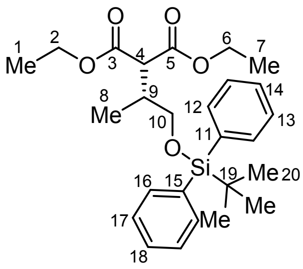

Following general procedure B. Purification by flash chromatography (SiO_2, 95:5 pentane:Et_2O) afforded title compound as a colourless liquid (113.5 mg, 0.250 mmol, 25%, 95% *e.e.*).

^1H NMR (400 MHz, $CDCl_3$):) δ 7.70 – 7.59 (m, 4H, H-12, H-16), 7.46 – 7.33 (m, 6H, H-13, H-14, H-17, H-18), 4.24 – 4.06 (m, 4H, H-2, H-6), 3.67 – 3.55 (m, 3H, H-4, H-10), 2.56 – 2.42 (m, 1H, H-9), 1.29 – 1.20 (m, 6H, H-1, H-7), 1.07 – 1.01 (m, 12H, H-8, H-20).

$^{13}C\{^1H\}$ NMR (101 MHz, $CDCl_3$): δ 169.2 (C-3 or C-5), 169.0 (C-3 or C-5), 135.74 (C-12 or C-16), 135.70 (C-12 or C-16), 133.7 (C-11 or C-15), 133.6 (C-11 or C-15), 129.8 (C-14, C-18), 127.79 (C-13, C-17), 66.3 (C-4), 61.4 (C-2 or C-6), 61.3 (C-2 or C-6), 53.8 (C-10), 36.0 (C-9), 27.0 (C-20), 19.5 (C-19), 14.5 (C-8), 14.3 (C-1 or C-7), 14.2 (C-1 or C-7).

IR (ATR, neat) (ν_{max} /cm^{-1}): 3049, 2960, 1752, 1730,1589.

HRMS: (ESI) m/z: $[M + Na]^+$ Calcd for $NaC_{26}H_{36}O_5Si^+$ 479.2225; Found 479.2229.

HPLC: The enantiomeric excess (95%) was determined by chiral HPLC analysis. (Daicel Chiralcel AD-H, IPA/isohexane = 5/95, flow rate = 1.0 mL/min, l = 210 nm) t_R = 3.76 min (major), 4.16 min (minor).

TLC: R_f 0.08 (95:5 pentane:Et_2O).

OR: $[\alpha]_D^{25}$: +8.0 (c. 1.0, $CHCl_3$)

(*S*)-Diethyl 2-(4-(phenylsulfonyl)butan-2-yl)malonate (4d)

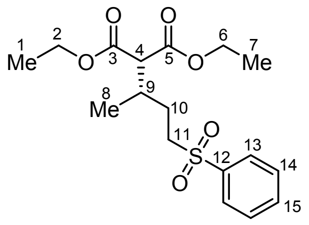

Following general procedure B. Purification by flash chromatography (SiO_2, 70:30 cyclohexane:EtOAc) afforded title compound as a yellow liquid (255 mg, 0.710 mmol, 71%, >99% *e.e.*).

^{1}H NMR (400 MHz, $CDCl_3$): δ 7.94 – 7.87 (m, 2H, H-13 or H-14), 7.70 – 7.63 (m, 1H, H-15), 7.61 – 7.55 (m, 2H, H-13 or H-14), 4.19 – 4.09 (m, 4H, H-2, H-6), 3.19 (d, J = 7.7 Hz, 1H, H-4), 3.20 – 3.04 (m, 2H, H-11), 2.39 – 2.24 (m, 1H, H-9), 1.92 – 1.79 (m, 1H, H-10a), 1.75 – 1.60 (m, 1H, H-10b), 1.26 – 1.18 (m, 6H, H-1, H-7), 0.98 (d, J = 6.8 Hz, 3H, H-8).

^{13}C{^{1}H} NMR (101 MHz, $CDCl_3$): δ 168.3 (C-3 or C-5), 168.2 (C-3 or C-5), 139.1 (C-12), 133.9 (C-15), 129.5 (C-13 or C-14), 128.2 (C-13 or C-14), 61.64 (C-2 or C-6), 61.58 (C-2 or C-6), 57.1 (C-4), 54.2 (C-11), 32.3 (C-9), 27.2 (C-10), 17.1 (C-8), 14.20 (C-1 or C-7), 14.19 (C-1 or C-7).

IR (ATR, neat) (ν_{max}/cm^{-1}): 2982, 2937, 1750, 1722, 1479.

HRMS: (ESI) m/z: $[M + Na]^+$ Calcd for $NaC_{17}H_{24}O_6S^+$ 379.1186; Found 379.1187.

HPLC: The enantiomeric excess (>99%) was determined by chiral HPLC analysis. (Daicel Chiralcel AS-H, IPA/isohexane = 10/90, flow rate = 1.0 mL/min, l = 210 nm) t_R = 48.95 min (major), minor not observed.

TLC: R_f 0.22 (70:30 cyclohexane:EtOAc).

OR: $[\alpha]_D^{25}$: –4.0 (c. 1.0, $CHCl_3$)

(*R*)-Dimethyl-2-(1-phenylpropan-2-yl)malonate (4j)

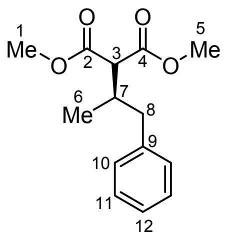

Following general procedure A, using a solution of sodium dimethylmalonate in THF as the nucleophile. Purification by flash chromatography (SiO_2, 93:7 pentane:Et_2O) afforded title compound as a colourless liquid (135 mg, 0.540 mmol, 54%, e.e. 95%).*

*Triphenylphosphine oxide **1** was recovered by eluting with 1:9 MeOH:CH_2Cl_2, solvent was removed *in vacuo* to give a colourless solid (322 mg, 1.16 mmol, 96% recovery).

^{1}H NMR (400 MHz, $CDCl_3$): δ 7.34 – 7.27 (m, 2H, H-11), 7.24 – 7.11 (m, 3H, H-10, H-12), 3.75 (s, 3H, H-1 or H-5), 3.73 (s, 3H, H-1 or H-5), 3.33 (d, *J* = 7.4 Hz, 1H, H-3), 2.81 (dd, *J* = 13.2, 5.2 Hz, 1H, H-8a), 2.62 – 2.50 (m, 1H, H-7), 2.43 (dd, *J* = 13.2, 8.9 Hz, 1H, H-8b), 0.95 (d, *J* = 6.7 Hz, 3H, H-6).

$^{13}C\{^1H\}$ NMR (101 MHz, $CDCl_3$): δ 169.5 (C-2 or C-4), 169.2 (C-2 or C-4), 139.9 (C-9), 129.4 (C-10), 128.5 (C-11), 126.4 (C-12), 56.7 (C-3), 52.5 (C-1 or C-5), 52.4 (C-1 or C-5), 40.8 (C-8), 35.7 (C-7), 17.0 (C-6).

IR (ATR, neat) ($ν_{max}$ /cm^{-1}): 3027, 2953, 1756, 1730, 1496.

HRMS: (ESI) m/z: $[M + Na]^+$ Calcd for $NaC_{14}H_{18}O_4^+$ 273.1098; Found 273.1099.

HPLC: The enantiomeric excess (95%) was determined by chiral HPLC analysis. (Daicel Chiralcel OD-H, IPA/isohexane = 5/95, flow rate = 1.0 mL/min, l = 210 nm) t_R = 6.75 min (minor), 9.69 min (major).

TLC: R_f 0.11 (93:7 pentane:Et_2O).

OR: $[α]_D^{25}$: –16.0 (c. 1.0, $CHCl_3$)

Data in agreement with literature.[13]

(*R*)-Diethyl 2-(5-phenylpent-4-yn-2-yl)malonate (4g)

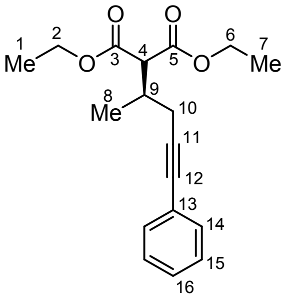

Following general procedure A. Purification by flash chromatography (SiO_2, 93:7 pentane:Et_2O) afforded title compound as a colourless liquid (195 mg, 0.650 mmol, 65%, 99% *e.e.*).

^{1}H NMR (400 MHz, $CDCl_3$): δ 7.43 – 7.36 (m, 2H, H-14 or H-15), 7.29 (m, 3H, H-14 or H-15, H-16), 4.29 – 4.13 (m, 4H, H-2, H-6), 3.58 – 3.42 (m, 1H, H-4), 2.63 – 2.49 (m, 3H, H-9, H-10), 1.30 – 1.25 (m, 6H, H-1, H-7), 1.17 (d, J = 6.7 Hz, 3H, H-8).

$^{13}C\{^1H\}$ NMR (101 MHz, $CDCl_3$): δ 168.8 (C-3 or C-5), 168.7 (C-3 or C-5), 131.7 (C-14 or C-15), 128.4 (C-14 or C-15), 127.9 (C-16), 123.8 (C-13), 87.2 (C-11), 82.8 (C-12), 61.52 (C-2 or C-6), 61.46 (C-2 or C-6), 56.4 (C-4), 32.9 (C-10), 24.8 (C-9), 17.4 (C-8), 14.3 (C-1 or C-7), 14.2 (C-1 or C-7).

IR (ATR, neat) ($ν_{max}$ /cm^{-1}): 2981, 2937, 1752, 1726, 1491.

HRMS: (ESI) m/z: $[M + Na]^+$ Calcd for $NaC_{18}H_{22}O_4^+$ 325.1411; Found 325.1410.

SFC: The enantiomeric excess (99%) was determined by chiral SFC analysis. (Chiralpak IG, methanol (0.1% v/v NH_3)/CO_2 = 5/95, flow rate = 3.0 mL/min, l = 210-400 nm) t_R = 3.23 min (minor), 3.66 min (major).

TLC: R_f 0.14 (93:7 pentane:Et_2O).

OR: $[α]_D^{25}$: –4.0 (c. 1.0, $CHCl_3$)

(*S*)-Diethyl 2-(1-(furan-2-yl)propan-2-yl)malonate (4h)

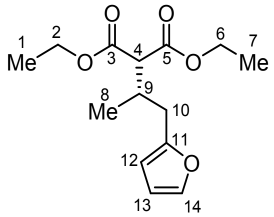

Following general procedure A. Purification by flash chromatography (SiO_2, 93:7 pentane:Et_2O) afforded title compound as a colourless liquid (191.3 mg, 0.710 mmol, 71%, 98% *e.e.*)

^{1}H NMR (400 MHz, $CDCl_3$): δ 7.34 – 7.29 (m, 1H, H-14), 6.31 – 6.25 (m, 1H, H-13), 6.04 (d, J = 3.1 Hz, 1H, H-12), 4.25 – 4.14 (m, 4H, H-2, H-6), 3.28 (d, J = 7.1 Hz, 1H, H-4), 2.85 – 2.74 (m, 1H, H-10a), 2.68 – 2.58 (m, 2H, H-9, H-10b), 1.31 – 1.23 (m, 6H, H-1, H-7), 1.00 (d, J = 6.4 Hz, 3H, H-8).

^{13}C{^{1}H} NMR (126 MHz, $CDCl_3$): δ 168.9 (C-3 or C-5), 168.7 (C-3 or C-5), 153.7 (C-11), 141.5 (C-14), 110.3 (C-13), 107.0 (C-12), 61.42 (C-2 or C-6), 61.36 (C-2 or C-6), 56.6 (C-4), 33.1 (C-9), 32.8 (C-10), 17.2 (C-8), 14.27 (C-1 or C-7), 14.25 (C-1 or C-7).

IR (ATR, neat) (ν_{max}/cm^{-1}): 2982, 2938, 2909, 1752, 1725, 1507.

HRMS: (ESI) m/z: $[M + Na]^+$ Calcd for $NaC_{14}H_{20}O_5^+$ 291.1203; Found 291.1206.

HPLC: The enantiomeric excess (98%) was determined by chiral HPLC analysis. (Daicel Chiralcel OD-H, IPA/isohexane = 5/95, flow rate = 1.0 mL/min, l = 210 nm) t_R = 5.25 min (major), 5.94 min (minor).

TLC: R_f 0.11 (93:7 pentane:Et_2O).

OR: $[\alpha]_D^{25}$: +16.0 (c. 1.0, $CHCl_3$)

Diethyl 2-cyclohexylmalonate (4f)

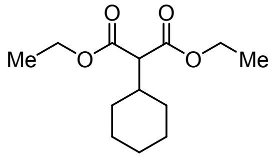

Following general procedure A. Purification by flash chromatography (SiO_2, 90:10 pentane:Et_2O) afforded title compound as a colourless liquid (127 mg, 0.520 mmol, 52%)

^{1}H NMR (400 MHz, $CDCl_3$): δ 4.18 (q, *J* = 7.1 Hz, 4H, CH_2), 3.13 (d, *J* = 9.1 Hz, 1H, CH), 2.16 – 2.02 (m, 1H, CH), 1.77 – 1.66 (m, 5H, CH_2), 1.36 – 0.99 (m, 11H, CH_2, CH_3).

^{13}C{^{1}H} NMR (101 MHz, $CDCl_3$): δ 169.0 (C_q), 61.3 (CH_2), 58.6 (CH), 38.1 (CH), 30.9 (CH_2), 26.2 (CH_2), 26.1 (CH_2), 14.3 (CH_3).

IR (ATR, neat) (ν_{max}/cm^{-1}): 2982, 2927, 2853, 1755, 1728, 1448.

HRMS: (ESI) m/z: $[M + Na]^+$ Calcd for $NaC_{13}H_{22}O_4^+$ 265.1411; Found 265.1411.

TLC: R_f 0.30 (90:10 pentane:Et_2O).

Data consistent with literature.[14]

Malonate ester products (made from primary alcohols)

Diethyl 2-(5-chloropentyl)malonate (4m)

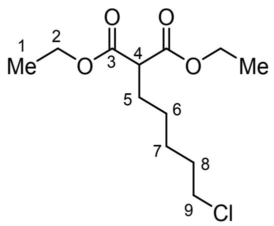

Following general procedure A. Purification by flash chromatography (SiO_2, 94:6 pentane:Et_2O) gave product with residual diethylmalonate. Residual diethylmalonate was removed under high vacuum at 50 °C using a heating block, which afforded title compound as a colourless liquid (190 mg, 0.720 mmol, 72%).

^{1}H NMR (400 MHz, $CDCl_3$): δ 4.23 – 4.15 (m, 4H, H-2), 3.52 (t, *J* = 6.6 Hz, 2H, H-9), 3.31 (t, *J* = 7.5 Hz, 1H, H-4), 1.95 – 1.85 (m, 2H, H-5), 1.83 – 1.72 (m, 2H, H-8), 1.52 – 1.42 (m, 2H, H-7), 1.41 – 1.30 (m, 2H, H-6), 1.26 (t, *J* = 7.1 Hz, 6H, H-1).

^{13}C{^{1}H} NMR (101 MHz, $CDCl_3$): δ 169.6 (C-3), 61.5 (C-2), 52.1 (C-4), 45.0 (C-9), 32.4 (C-8), 28.7 (C-5), 26.7 (C-6), 26.6 (C-7), 14.2 (C-1).

IR (ATR, neat) (ν_{max} /cm^{-1}): 2960, 2937, 2906, 1751, 1727, 1465.

HRMS: (ESI) m/z: $[M + Na]^+$ Calcd for $NaC_{12}H_{21}O_4Cl^+$ 287.1021; Found 287.1018.

TLC: R_f 0.11 (94:6 pentane:Et_2O).

Data consistent with literature.[15]

Diethyl 2-(2-fluorophenethyl)malonate (4o)

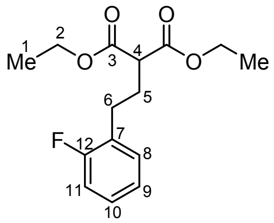

Following general procedure A. Purification by flash chromatography (SiO_2, 95:5 pentane:Et_2O) afforded title compound as a colourless liquid (214 mg, 0.750 mmol, 75%).

Using recovered triphenylphopshine oxide (1): To a microwave tube was added anhydrous NaOTf (215 mg, 1.25 mmol, 2.50 equiv.) and anhydrous recovered triphenylphosphine oxide **1** (167 mg, 0.60 mmol, 1.20 equiv.). The tube was then heated (heat gun) under high vacuum for 2.5 minutes, before filling with argon. The tube was evacuated and backfilled with argon a further two times. Anhydrous THF (0.5 mL) and MeCN (1 mL) were added, before oxalyl chloride (52.5 µL, 0.615 mmol, 1.23 equiv.) was added dropwise (Caution: slow addition due to evolution of CO_2 and CO gas). After stirring for 30 minutes at room temperature, anhydrous 2-(2-fluorophenyl)ethanol (67.0 µL, 0.50 mmol, 1.00 equiv.) was added, and the reaction was stirred for 4 hours at room temperature. After which time NaDEM **3** (1.60 mL, 1.50 mmol, 3.00 equiv. of a 0.950 M solution in THF) was added, and the reaction was stirred for 16 hours at room temperature. Upon completion, solvent was removed *in vacuo*, and crude mixture purified by flash chromatography (SiO_2, 95:5 pentane:Et_2O) to afford title compound as a colourless liquid (105 mg, 0.370 mmol, 74%).

^{1}H NMR (400 MHz, $CDCl_3$): δ 7.22 – 7.15 (m, 2H, H-9, H-11) 7.10 – 6.97 (m, 2H, H-8, H-10), 4.24 – 4.16 (m, 4H, H-2), 3.34 (t, J = 7.5 Hz, 1H, H-4), 2.75 – 2.66 (m, 2H, H-6), 2.26 – 2.17 (m, 2H, H-5), 1.27 (t, J = 7.1 Hz, 6H, H-1).

^{13}C{^{1}H} NMR (101 MHz, $CDCl_3$): δ 169.4 (C-3), 161.3 (d, J = 245.2 Hz, C-12), 130.9 (d, J = 4.8 Hz, C-8 or C-10), 128.1 (d, J = 8.0 Hz, C-8 or C-10), 127.6 (d, J = 15.8 Hz, C-7), 124.2 (d, J = 3.6 Hz, C-9), 115.5 (d, J = 22.1 Hz, C-11), 61.6 (C-2), 51.5 (C-4), 29.1 (C-5), 26.7 (d, J = 2.5 Hz, C-6), 14.2 (C-1).

^{19}F NMR (376 MHz, $CDCl_3$) δ -118.59.

IR (ATR, neat) (v_{max} /cm^{-1}): 2982, 2940, 1751, 1727, 1492.

HRMS: (ESI) m/z: $[M + Na]^+$ Calcd for $NaC_{15}H_{19}O_4F^+$ 305.1160; Found 305.1155.

TLC: R_f 0.62 (90:10 pentane:Et_2O).

Data consistent with literature.[16]

3-Benzyl 1,1-diethyl (*R*)-butane-1,1,3-tricarboxylate (4q)

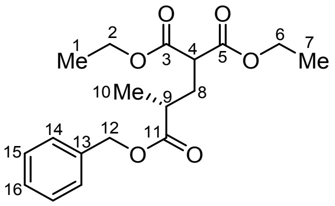

Following general procedure A. Purification by flash chromatography (SiO_2, 93:7 pentane:Et_2O) afforded title compound as a colourless liquid (227 mg, 0.68 mmol, 68%, 97% *e.e.*).

^{1}H NMR (400 MHz, CDCl$_3$): δ 7.40 – 7.30 (m, 5H, H-14, H-15, H-16), 5.12 (d, *J* = 3.1 Hz, 2H, H-12), 4.25 – 4.09 (m, 4H, H-2, H-6), 3.43 (dd, *J* = 8.8, 6.4 Hz, 1H, H-4), 2.63 – 2.49 (m, 1H, H-9), 2.33 – 2.21 (m, 1H, H-8a), 2.10 – 1.98 (m, 1H, H-8b), 1.28 – 1.20 (m, 9H, H-1, H-7, H-10).

^{13}C{^{1}H} NMR (101 MHz, CDCl$_3$): δ 175.5 (C-11), 169.3 (C-3 or C-5), 169.1 (C-3 or C-5), 136.1 (C-13), 128.7 (C-15), 128.3 (C-16), 128.2 (C-14), 66.5 (C-12), 61.7 (C-2 or C-6), 61.6 (C-2 or C-6), 50.1 (C-4), 37.5 (C-9), 32.3 (C-8), 17.6 (C-10), 14.2 (C-1, C-7).

IR (ATR, neat) (v_{max} /cm^{-1}): 2981, 2939, 2907, 1752, 1724, 1457.

HRMS: (ESI) m/z: $[M + Na]^+$ Calcd for $NaC_{18}H_{24}O_6^+$ 359.1466; Found 359.1466.

HPLC: The enantiomeric excess (97%) was determined by chiral HPLC analysis. (Daicel Chiralcel IC, IPA/isohexane = 5/95, flow rate = 1.0 mL/min, l = 210 nm) t_R = 23.16 min (minor), 24.57 min (major).

TLC: R_f 0.054 (93:7 pentane:Et_2O).

OR: $[\alpha]_D^{25}$: –24.0 (c. 1.0, CHCl$_3$)

Diethyl 2-(3-(dimethylamino)-3-oxopropyl)malonate (4p)

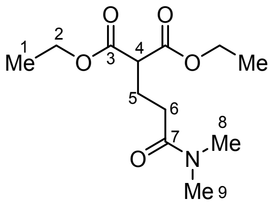

Following general procedure A. Purification by flash chromatography (SiO_2, 95:5 Et_2O:pentane) afforded title compound as a yellow liquid (151.3 mg, 0.580 mmol, 58%).

^{1}H NMR (500 MHz, CDCl$_3$): δ 4.24 – 4.14 (m, 4H, H-2), 3.50 (t, J = 7.2 Hz, 1H, H-4), 2.98 (s, 3H, H-8 or H-9), 2.93 (s, 3H, H-8 or H-9), 2.40 (t, J = 7.4 Hz, 2H, H-6), 2.28 – 2.17 (m, 2H, H-5), 1.26 (t, J = 7.1 Hz, 6H, H-1).

^{13}C{^{1}H} NMR (126 MHz, CDCl$_3$): δ 171.7 (C-7), 169.5 (C-3), 61.5 (C-2), 51.2 (C-4), 37.3 (C-8 or C-9), 35.6 (C-8 or C-9), 30.6 (C-6), 24.3 (C-5), 14.2 (C-1).

IR (ATR, neat) (v_{max} /cm^{-1}): 2983, 2937, 2875, 1749, 1724, 1648.

HRMS: (ESI) m/z: $[M + Na]^+$ Calcd for $NaC_{12}H_{21}O_5N^+$ 282.1312; Found 282.1317.

TLC: R_f 0.29 (Et_2O).

Data is consistent with literature.[17]

Diethyl 2-(5-bromopentyl)malonate (4n)

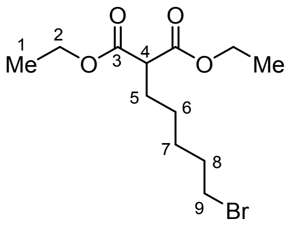

Following general procedure A. Purification by flash chromatography (SiO_2, 95:5 Et_2O:pentane) afforded title compound as a colourless liquid (106.7 mg, 0.350 mmol, 35%).

^{1}H NMR (400 MHz, $CDCl_3$): δ 4.24 – 4.13 (m, 4H, H-2), 3.39 (t, J = 6.8 Hz, 2H, H-9), 3.31 (t, J = 7.5 Hz, 1H, H-4), 1.95 – 1.80 (m, 4H, H-5, H-8), 1.54 – 1.42 (m, 2H, H-7), 1.42 – 1.32 (m, 2H, H-6), 1.27 (t, J = 7.1 Hz, 6H, H-1).

^{13}C{^{1}H} NMR (101 MHz, $CDCl_3$): δ 169.6 (C-3), 61.5 (C-2), 52.1 (C-4), 33.7 (C-9), 32.5 (C-8), 28.6 (C-5), 27.9 (C-7), 26.6 (C-6), 14.2 (C-1).

IR (ATR, neat) (v_{max} /cm^{-1}): 2982, 2937, 2863, 1751, 1727, 1464.

HRMS: (ESI) m/z: $[M + Na]^+$ Calcd for $NaC_{12}H_{21}O_4Br^+$ 311.0516, 333.0495; Found 311.0669, 333.0490.

TLC: R_f 0.06 (95:5 pentane:Et_2O).

Known compound. Analytical data not in the literature.[18]

Diethyl 2-(2-(pyridin-2-yl)ethyl)malonate (4r)

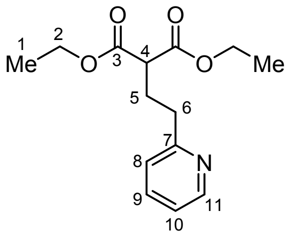

Following general procedure A. Purification by flash chromatography (SiO_2, 1:1 Et_2O:pentane) afforded title compound as a light-yellow liquid (229 mg, 0.860 mmol, 86%).

^{1}H NMR (400 MHz, $CDCl_3$): δ 8.56 – 8.50 (m, 1H, H-11), 7.63 – 7.55 (m, 1H, H-9), 7.19 – 7.07 (m, 2H, H-8, H-10), 4.24 – 4.15 (m, 4H, H-2), 3.40 (t, J = 7.5 Hz, 1H, H-4), 2.89 – 2.80 (m, 2H, H-6), 2.40 – 2.30 (m, 2H, H-5), 1.26 (t, J = 7.1 Hz, 6H, H-1).

^{13}C{^{1}H} NMR (101 MHz, $CDCl_3$): 169.4 (C-3), 160.6 (C-7), 149.6 (C-11), 136.5 (C-9), 123.1 (C-8), 121.5 (C-10), 61.5 (C-2), 51.5 (C-4), 35.8 (C-6), 28.6 (C-5), 14.2 (C-1).

IR (ATR, neat) (v_{max} /cm^{-1}): 2982, 2937, 2863, 1751, 1727, 1464.

HRMS: (ESI) m/z: $[M + Na]^+$ Calcd for $NaC_{14}H_{19}O_4N^+$ 288.1207; Found 288.2108.

TLC: R_f 0.15 (1:1 Et_2O:pentane)

Data consistent with literature.[19]

Alternative nucleophile products

(*S*)-2-(4-phenylbutan-2-yl)malononitrile (4l)

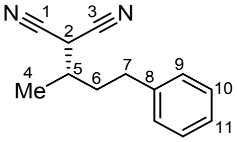

To a microwave tube was added anhydrous NaOTf (430 mg, 2.50 mmol) and anhydrous triphenylphosphine oxide **1** (334 mg, 1.20 mmol), then heated (heat gun) under high vacuum for 2.5 minutes, before filling with argon. The tube was evacuated and backfilled with argon a further two times. Anhydrous THF (1 mL) and MeCN (2 mL) were added. Oxalyl chloride (105 µL, 1.23 mmol) was added dropwise (Caution: evolution of CO_2 and CO gas) and then the mixture was stirred at room temperature for 30 minutes. Anhydrous (*R*)-(−)-4-Phenylbutan-2-ol (154 µL, 1.00 mmol, 1.00 equiv.) was added, and the mixture was stirred at room temperature for 4 hours. The solution was cooled to 0 °C, sodium hydride (120 mg, 3.00 mmol of a 60 wt.% suspension in mineral oil) was added, before malononitrile (198 mg, 3.00 mmol) was added in 5 portions (Caution: evolution of H_2 gas) before warming to room temperature and stirring for 16 hours. The solvent was removed *in vacuo*. Purification by flash chromatography (SiO_2, 93:7 pentane:Et_2O) resulted the title compound as a light-yellow liquid (136 mg, 0.690 mmol, 69%, >99% *e.e.*).

^{1}H NMR (400 MHz, $CDCl_3$): δ 7.35 – 7.28 (m, 2H, H-10), 7.26 – 7.14 (m, 3H, H-11, H-9), 3.64 (d, J = 5.0 Hz, 1H, H-2), 2.85 – 2.73 (m, 1H, H-7a), 2.71 – 2.59 (m, 1H, H-7b), 2.26 – 2.11 (m, 1H, H-5), 2.04 – 1.90 (m, 1H, H-6a), 1.89 – 1.74 (m, 1H, H-6b), 1.31 (d, J = 6.7 Hz, 3H, H-4).

^{13}C{^{1}H} NMR (126 MHz, $CDCl_3$): δ 140.1 (C-8), 128.9 (C-10), 128.4 (C-9), 126.7 (C-11), 112.2 (C-1 or C-3), 111.7 (C-1 or C-3), 35.3 (C-6), 35.1 (C-5), 32.9 (C-7), 29.4 (C-2), 16.9 (C-4).

IR (ATR, neat) (ν_{max} /cm^{-1}): 3028, 2921, 1496.

HRMS: (ESI) m/z: [M + Na]$^+$ Calcd for $NaC_{13}H_{14}N_2^+$ 221.1050; Found 221.1045.

HPLC: The enantiomeric excess (>99%) was determined by chiral HPLC analysis. (Daicel Chiralcel OD-H, IPA/isohexane = 5/95, flow rate = 1.0 mL/min, l = 210 nm) t_R = 31.41 min (major), 45.42 min (minor).

TLC: R_f 0.27 (8:2 pentane:Et_2O)

OR: $[\alpha]_D^{25}$: –8.0 (c. 1.0, $CHCl_3$)

^{1}H, ^{13}C, IR and HRMS matches literature for known racemic compound.[20]

(*S*)-(2-methyl-4-phenylbutane-1,1-diyldisulfonyl)dibenzene (4k)

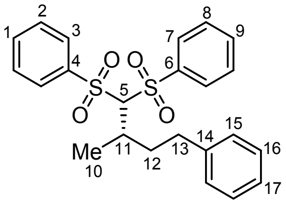

To a microwave tube was added anhydrous NaOTf (430 mg, 2.50 mmol, 2.50 equiv.) and anhydrous triphenylphosphine oxide **1** (334 mg, 1.20 mmol, 1.20 equiv.), then heated (heat gun) under high vacuum for 2.5 minutes, before filling with argon. The tube was evacuated and backfilled with argon a further two times. Anhydrous THF (1 mL) and MeCN (2 mL) were added. Oxalyl chloride (105 µL, 1.23 mmol, 1.23 equiv.) was added (Caution: evolution of CO_2 and CO gas) and then the mixture was stirred at room temperature for 30 minutes. Anhydrous (*R*)-(-)-4-Phenylbutan-2-ol (154 µL, 1.00 mmol, 1.00 equiv.) was added in one portion, and the mixture was stirred at room temperature for 4 hours. To the reaction was added potassium bis(phenylsulfonyl)methane (1.00 g, 3.00 mmol) and anhydrous 18-crown-6 (793 mg, 3.00 mmol) in 1:1 MeCN:THF (6 mL) and the reaction was stirred at

room temperature for 16 hours. The solvent was removed *in vacuo*. Purification by flash chromatography (SiO_2, 70:30 cyclohexane:EtOAc) gave the title compound as a colourless solid (236 mg, 0.550 mmol, 55%, 93% e.e.).

^{1}H NMR (500 MHz, $CDCl_3$): δ 7.91 – 7.86 (m, 2H, H-2 or H-3 or H-7 or H-8), 7.81 – 7.76 (m, 2H, H-2 or H-3 or H-7 or H-8), 7.69 – 7.61 (m, 2H, H-1, H-9), 7.58 – 7.45 (m, 4H, H-2 or H-3 or H-7 or H-8), 7.32 – 7.19 (m, 3H, H-15 or H-16, H-17), 7.14 – 7.07 (m, 2H, H-15 or H-16), 4.50 (d, J = 1.3 Hz, 1H, H-5), 2.77 – 2.66 (m, 1H, H-11), 2.59 – 2.53 (m, 1H, H-12a), 2.52 – 2.43 (m, 1H, H-12b), 2.18 – 2.09 (m, 2H, H-13), 1.28 (d, J = 7.1 Hz, 3H, H-10).

^{13}C{^{1}H} NMR (126 MHz, $CDCl_3$): δ 141.1 (C-14), 140.1 (C-4 or C-6), 138.9 (C-4 or C-6), 134.6 (C-1 or C-9), 134.3 (C-1 or C-9), 129.8 (C-2 or C-3 or C-7 or C-8), 129.3 (C-2 or C-3 or C-7 or C-8), 129.20 (C-2 or C-3 or C-7 or C-8), 129.15 (C-2 or C-3 or C-7 or C-8), 128.69 (C-15 or C-16), 128.67 (C-15 or C-16), 126.2 (C-17), 87.2 (C-5), 36.0 (C-13), 34.4 (C-12), 34.1 (C-11), 16.7 (C-10).

IR (ATR, neat) (v_{max} /cm^{-1}): 3023, 2921, 1739, 1583.

HRMS: (ESI) m/z: $[M + Na]^+$ Calcd for $NaC_{23}H_{24}O_4S_2^+$ 451.1009; Found 451.1013.

HPLC: The enantiomeric excess (93%) was determined by chiral HPLC analysis. (Daicel Chiralcel AD-H, IPA/isohexane = 3/97, flow rate = 1.0 mL/min, l = 254 nm) t_R = 21.23 min (minor), 25.30 min (major).

TLC: R_f 0.11 (6:4 pentane:Et_2O)

OR: $[\alpha]_D^{25}$: –36.0 (c. 1.0, $CHCl_3$)

m.p.: 157-159 °C.

(*R*)-((2-chloropropoxy)methyl)benzene (2)

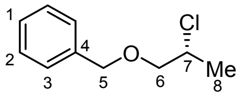

To a microwave tube was added triphenylphosphine oxide **1** (279 mg, 1.00 mmol) and THF (3 mL). To this mixture was added oxalyl chloride dropwise (88.0 µL, 1.03 mmol) (Caution: formation of CO and CO_2 gas) and the mixture was stirred for 30 minutes at room temperature. (*S*)-1-(benzyloxy)propan-2-ol (155.6 µL, 1.00 mmol) was added and the mixture was stirred at room temperature for 18 hours. Solvent was removed *in vacuo* and purification by flash chromatography (SiO_2, 98:2 pentane:Et_2O) afforded pure title compound as a colourless liquid (92.6 mg, 0.500 mmol, 50%, 95% e.e.).

^{1}H NMR (400 MHz, $CDCl_3$): δ 7.39 – 7.27 (m, 5H, H-1, H-2, H-3), 4.59 (d, J = 0.9 Hz, 2H, H-5), 4.21 – 4.08 (m, 1H, H-7), 3.61 (dd, J = 10.1, 6.2 Hz, 1H, H-6a), 3.54 (dd, J = 10.1, 6.1 Hz, 1H, H-6b), 1.52 (d, J = 6.6 Hz, 3H, H-8).

^{13}C{^{1}H} NMR (101 MHz, $CDCl_3$): δ 138.0 (C-4), 128.6 (C-2), 127.9 (C-1), 127.8 (C-3), 75.4 (C-6), 73.4 (C-5), 55.5 (C-7), 22.0 (C-8).

IR (ATR, neat) (v_{max} /cm^{-1}): 3088, 3065, 3031, 1496.

HRMS: (ESI) m/z: $[M + Na]^+$ Calcd for $NaC_{10}H_{23}OCl^+$ 207.0552; Found 207.0548.

HPLC: The enantiomeric excess (95%) was determined by chiral HPLC analysis. (Daicel Chiralcel OD-H, IPA/isohexane = 2/98, flow rate = 1.0 mL/min, l = 210 nm) t_R = 5.26 min (major), 5.71 min (minor).

TLC: R_f 0.23 (pentane:Et_2O 98:2)

OR: $[\alpha]_D^{25}$: +4.0 (c. 1.0, $CHCl_3$)

^{1}H, ^{13}C, and IR data match that of the racemic compound.[21]

Ethyl 2-acetyl-4-(pyridin-2-yl)butanoate (4s)

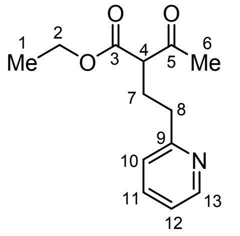

Following general procedure A, using a solution of sodium ethyl acetoacetate in THF as the nucleophile. Purification by flash chromatography (SiO_2, 60:40 Et_2O:pentane) afforded title compound as a light-yellow liquid (83.0 mg, 0.350 mmol, 35%).

^{1}H NMR (400 MHz, $CDCl_3$): δ 8.56 – 8.47 (m, 1H, H-13), 7.62 – 7.52 (m, 1H, H-11), 7.19 – 7.05 (m, 2H, H-10, H-12), 4.18 (q, *J* = 7.2 Hz, 2H, H-2), 3.49 (t, *J* = 7.3 Hz, 1H, H-4), 2.86 – 2.72 (m, 2H, H-8), 2.37 – 2.26 (m, 2H, H-7), 2.23 (s, 3H, H-6), 1.26 (t, *J* = 0.9 Hz, 3H, H-1).

^{13}C{^{1}H} NMR (126 MHz, $CDCl_3$): δ 203.2 (C-5), 169.7 (C-3), 160.6 (C-9), 149.5 (C-13), 136.6 (C-11), 123.1 (C-10 or C-12), 121.5 (C-10 or C-12), 61.5 (C-2), 59.1 (C-4), 35.7 (C-8), 29.2 (C-6), 27.8 (C-7), 14.2 (C-1).

IR (ATR, neat) (v_{max}/cm^{-1}): 3434, 2981, 2936, 1736, 1711, 1644.

HRMS: (ESI) m/z: [M + H]$^+$ Calcd for $C_{13}H_{18}NO_3^+$ 236.1282; Found 236.1283.

TLC: R_f 0.14 (EtOAc:pentane 60:40)

Diethyl 2-methyl-2-(2-(pyridin-2-yl)ethyl)malonate (4t)

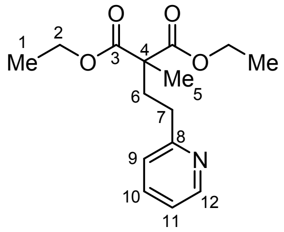

Following general procedure A, using a solution of sodium diethylmethylmalonate in THF as the nucleophile. Purification by flash chromatography (SiO_2, 1:1 Et_2O:pentane) afforded title compound as a colourless liquid (254 mg, 0.910 mmol, 91%).

^{1}H NMR (500 MHz, $CDCl_3$): δ 8.56 – 8.48 (m, 1H, H-12), 7.65 – 7.53 (m, 1H, H-10), 7.19 – 7.14 (m, 1H, H-9 or H-11), 7.12 – 7.03 (m, 1H, H-9 or H-11), 4.25 – 4.11 (m, 4H, H-2), 2.81 – 2.72 (m, 2H, H-7), 2.34 – 2.23 (m, 2H, H-6), 1.55 – 1.46 (m, 3H, H-5), 1.30 – 1.17 (m, 6H, H-1).

^{13}C{^{1}H} NMR (126 MHz, $CDCl_3$): δ 172.3 (C-3), 161.4 (C-8), 149.4 (C-12), 136.5 (C-10), 122.9 (C-9 or C-11), 121.3 (C-9 or C-11), 61.4 (C-2), 53.7 (C-4), 35.7 (C-7), 33.5 (C-6), 20.1 (C-5), 14.2 (C-1).

IR (ATR, neat) (v_{max}/cm^{-1}): 2982, 2940, 1725, 1590.

HRMS: (ESI) m/z: [M + H]$^+$ Calcd for $C_{15}H_{22}NO_4^+$ 280.1544; Found 280.1539.

TLC: R_f 0.19 (Et_2O:pentane 1:1)

Assignment of Absolute Stereochemistry

Compounds **S12**, **S13**, and **S14**, which have been previously prepared through asymmetric synthesis by Taguri and co-workers,[13] were prepared according to reference 13. Comparison of the HPLC retention time and specific rotation of product **4c** with **S12** and **S14** shows that product **4c** has the (*R*)-stereochemistry confirming that

inversion of configuration has taken place. The stereochemical outcome of other deoxyalkylation reactions were assigned by analogy.

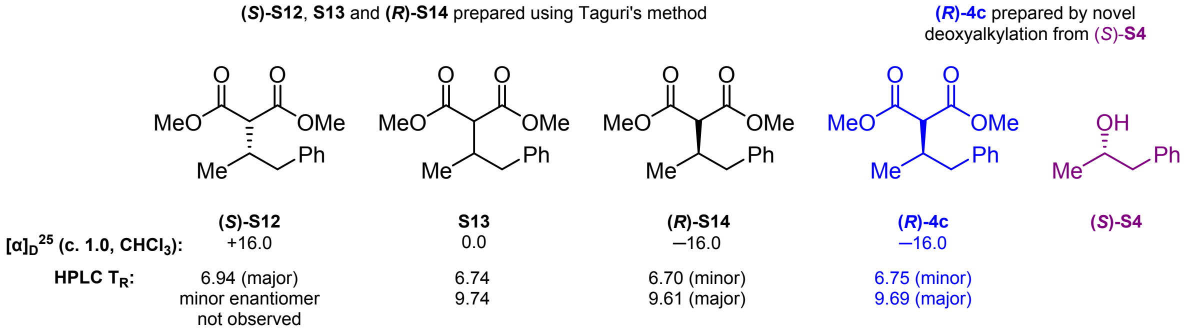

Figure S1: Absolute stereochemical assignment of product ***4c****. HPLC conditions: Column: Daicel Chiralcel OD-H (4.6 mm x 250 mm), mobile phase: isohexane:IPA = 95:5, flow rate: 1 mL/min, temperature: 25 °C, detector: 210 nm.*

(*S*)-Dimethyl-2-(1-phenylpropan-2-yl)malonate (S12)

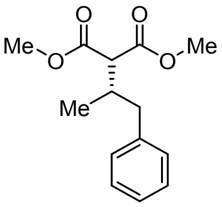

^{1}H NMR, ^{13}C{1H} NMR, IR and HRMS match compound 4j.

HPLC: The enantiomeric excess (>99%) was determined by chiral HPLC analysis. (Daicel Chiralcel OD-H, IPA/isohexane = 5/95, flow rate = 1.0 mL/min, l = 210 nm) t_R = 6.94 (major), minor not observed.

OR: $[\alpha]_D^{25}$: +16.0 (c. 1.0, $CHCl_3$)

Data consistent with literature.[13]

Dimethyl-2-(1-phenylpropan-2-yl)malonate (S13)

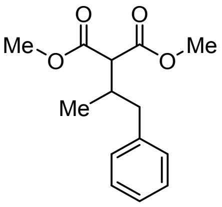

^{1}H NMR, ^{13}C{1H} NMR, IR and HRMS match compound 4j.

HPLC: The enantiomeric excess (racemic) was determined by chiral HPLC analysis. (Daicel Chiralcel OD-H, IPA/isohexane = 5/95, flow rate = 1.0 mL/min, l = 210 nm) t_R = 6.74, 9.74.

Data consistent with literature.[13]

(*R*)-Dimethyl-2-(1-phenylpropan-2-yl)malonate (S14)

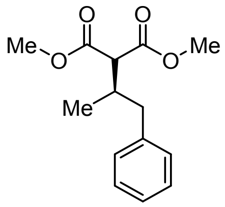

^{1}H NMR, ^{13}C{1H} NMR, IR and HRMS match compound 4j.

HPLC: The enantiomeric excess (92%) was determined by chiral HPLC analysis. (Daicel Chiralcel OD-H, IPA/isohexane = 5/95, flow rate = 1.0 mL/min, l = 210 nm) t_R = 6.70 (minor), 9.61 (major).

OR: $[\alpha]_D^{25}$: –16.0 (c. 1.0, $CHCl_3$)

Data consistent with literature.[13]

Isolation of kinetically stable alkoxyphosphonium salts

(*S*)-((1-(benzyloxy)propan-2-yl)oxy)triphenylphosphonium triflate (5a)

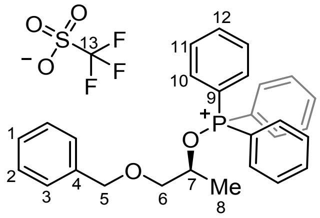

To a microwave tube was added anhydrous NaOTf (430 mg, 2.50 mmol, 2.50 equiv.) and anhydrous triphenylphosphine oxide **1** (334 mg, 1.20 mmol, 1.20 equiv.). The tube was then heated (heat gun) under high vacuum for 2.5 minutes, before filling with argon. The tube was evacuated and backfilled with argon a further two times. Anhydrous THF (3 mL) was added, before oxalyl chloride (105 µL, 1.23 mmol, 1.23 equiv.) was added dropwise (Caution: slow addition due to evolution of CO_2 and CO gas). After stirring for 15 minutes at 50 °C using a heating block, anhydrous (*S*)-1-(benzyloxy)propan-2-ol (156 µL, 1.00 mmol, 1.00 equiv.) was added, and the reaction was stirred for 30 minutes at 50 °C. The suspension was filtered and washed with $CHCl_3$ (2 × 10 mL). Solvent was removed *in vacuo*. To the crude liquid was added Et_2O (10 mL), before stirring for 5 minutes and decanting off the Et_2O. This process was repeated two further times, to result title compound as a brown liquid (566 mg, 0.980 mmol, 98%).

^{1}H NMR (500 MHz, $CDCl_3$): δ 7.86 – 7.81 (m, 3H, H-12), 7.77 – 7.69 (m, 6H, H-11), 7.69 – 7.62 (m, 6H, H-10), 7.34 – 7.27 (m, 3H, H-1, H-2), 7.09 – 7.03 (m, 2H, H-3), 4.81 – 4.72 (m, 1H, H-7), 4.35 – 4.24 (m, 2H, H-5), 3.85 – 3.75 (m, 1H, H-6a), 3.63 – 3.55 (m, 1H, H-6b), 1.44 (d, J = 6.3 Hz, 3H, H-8).

^{13}C{^{1}H} NMR (126 MHz, $CDCl_3$): δ 137.0 (C-4), 136.2 (d, *J* = 3.0 Hz, C-12), 133.8 (d, *J* = 11.7 Hz, C-11), 130.4 (d, *J* = 13.6 Hz, C-10), 128.6 (C-2), 128.4 (C-3), 128.3 (C-1), 120.2 (d, *J* = 108.0 Hz, C-9), 82.1 (d, *J* = 9.2 Hz, C-7), 73.40 (C-5 or C-6), 73.38 (C-5 or C-6), 18.3 (d, *J* = 4.6 Hz, C-8).

^{31}P NMR (202 MHz, $CDCl_3$): δ 61.19.

^{19}F NMR (376 MHz, $CDCl_3$): δ −78.12.

HRMS: (ESI) m/z: $[M]^+$ Calcd for $C_{28}H_{28}O_2P^+$ 427.1822; Found 427.1846.

(*S*)-((1-(benzyloxy)propan-2-yl)oxy)triphenylphosphonium tetrakis[3,5-bis(trifluoro-methyl)phenyl]borate (5b)

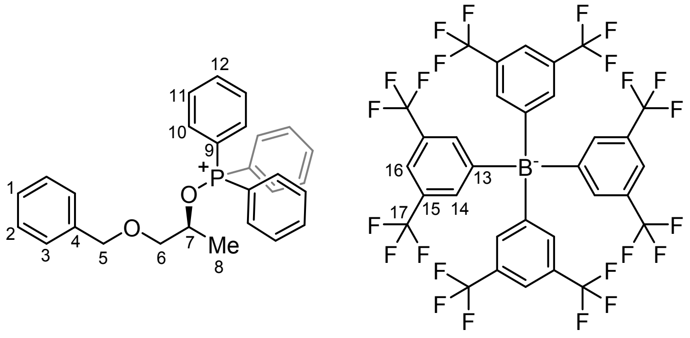

To a microwave tube was added anhydrous NaOTf (430 mg, 2.50 mmol, 2.50 equiv.) and anhydrous triphenylphosphine oxide **1** (334 mg, 1.20 mmol, 1.20 equiv.). The tube was then heated (heat gun) under high vacuum for 2.5 minutes, before filling with argon. The tube was evacuated and backfilled with argon a further two times. Anhydrous THF (3 mL) was added, before oxalyl chloride (105 µL, 1.23 mmol, 1.23 equiv.) was added dropwise (Caution: slow addition due to evolution of CO_2 and CO gas). After stirring for 15 minutes at 50 °C, using a heating block, anhydrous (*S*)-1-(benzyloxy)propan-2-ol (156 µL, 1.00 mmol, 1.00 equiv.) was added, and the reaction was stirred for 30 minutes at 50 °C. The suspension was filtered and washed with $CHCl_3$ (2 × 10 mL). Solvent was removed *in vacuo*. To the crude liquid was added Et_2O (10 mL), before stirring for 5 minutes and decanting off the Et_2O. This process was repeated two further times, to result (*S*)-((1-(benzyloxy)propan-2-yl)oxy)triphenylphosphonium triflate. To this was added $CHCl_3$ (10 mL) and sodium tetrakis[3,5-bis(trifluoromethyl)phenyl]borate (886 mg, 1.00 mmol, 1.00 equiv.) before stirring at room temperature for 16 hours. The suspension was filtered, and solvent was removed *in vacuo* to result the title compound as a colourless solid (1.03 g, 0.80 mmol, 80%).

^{1}H NMR (500 MHz, $CDCl_3$): δ 7.80 – 7.74 (m, 3H, H-12), 7.73 – 7.67 (m, 8H, H-14), 7.65 – 7.52 (m, 12H, H-10, H-11), 7.49 (s, 4H, H-16), 7.35 – 7.27 (m, 3H, H-1, H-2), 7.02 – 6.96 (m, 2H, H-3), 4.60 – 4.48 (m, 1H, H-7), 4.30 – 4.16 (m, 2H, H-5), 3.71 – 3.63 (m, 1H, H-6a), 3.54 – 3.46 (m, 1H, H-6b), 1.32 (d, J = 6.4 Hz, 3H, H-8).

^{13}C{^{1}H} NMR (126 MHz, $CDCl_3$): δ 161.8 (q, J = 49.8 Hz, C-13), 136.4 (d, J = 3.0 Hz, C-12), 136.2 (C-4), 134.9 (C-14), 133.4 (d, J = 11.6 Hz, C-11), 130.3 (d, J = 13.6 Hz, C-10), 129.1 (m, C-15), 128.72 (C-2), 128.65 (C-1), 128.4 (C-3), 124.7 (q, J = 272.5 Hz, C-17), 119.9 (d, J = 108.1 Hz, C-9), 117.6 (C-16), 81.7 (d, J = 9.1 Hz, C-7), 73.7 (C-5), 73.3 (d, J = 2.8 Hz, C-6), 18.0 (d, J = 4.9 Hz, C-8).

^{31}P NMR (162 MHz, $CDCl_3$): δ 62.13.

^{19}F NMR (471 MHz, $CDCl_3$): δ −62.41.

HRMS: (ESI) m/z: $[M]^+$ Calcd for $C_{28}H_{28}O_2P^+$ 427.1822; Found 427.1820.

Unsuccessful alcohols and nucleophiles

Unsuccessful alcohols

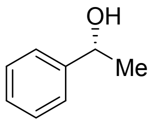

Forms styrene.

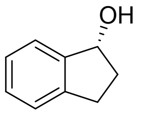

Forms indene.

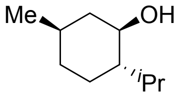

Forms a mixture of alkenes.

Unsuccessful nucleophiles

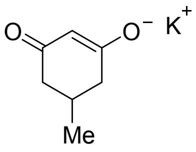

C-alkylation not observed. *O*-alkylation observed using a solution of the potassium enolate with 18-crown-6.

RMgX

Forms Appel product (Schlenk equilibrium).

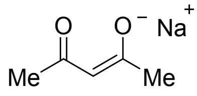

No reaction observed.

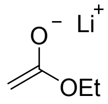

No productive reaction observed.

Preparation of Chiral HPLC Standards

Secondary alcohols

2-octanol (S15)

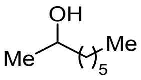

HPLC: Racemic, confirmed by synthesis of the 4-nitrobenzyl ester – see **S25**.

1-phenyl-2-propanol (S16)

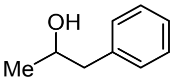

HPLC: The enantiomeric excess (racemic) was determined by chiral HPLC analysis. (Daicel Chiralcel OD-H, IPA/isohexane = 5/95, flow rate = 1.0 mL/min, l = 210 nm) t_R = 7.54, 8.40.

4-Phenylbutan-2-ol (S17)

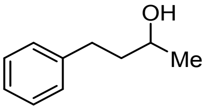

HPLC: The enantiomeric excess (racemic) was determined by chiral HPLC analysis. (Daicel Chiralcel OD-H, IPA/isohexane = 10/90, flow rate = 1.0 mL/min, l = 210 nm) t_R = 6.81, 9.34.

1-benzyloxy-2-propanol (S18)

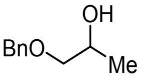

To a solution of benzyl glycidyl ether (2.0 mL, 12.00 mmol) in THF (10 mL) was added $LiAlH_4$ (713.4 mg, 18.80 mmol) in portions at 0 °C, under an argon atmosphere and the reaction mixture was stirred at room temperature for 2 hours. The reaction was then diluted with Et_2O (50 mL) and cooled to 0 °C before H_2O (0.7 mL) was added dropwise, followed by NaOH (0.7 mL of a 15% w/v aqueous solution) then H_2O (2.1 mL). The reaction was warmed to room temperature and stirred for 15 minutes, before $MgSO_4$ (5 g) was added and the reaction mixture stirred for a further 15 minutes then filtered, eluting with EtOAc. The solvent was removed in vacuo to afford the title compound as a colourless liquid (1.98 g, 99%).

^{1}H NMR, ^{13}C{1H} NMR, IR and HRMS match compound A1.

HPLC: The enantiomeric excess (racemic) was determined by chiral HPLC analysis. (Daicel Chiralcel AS-H, IPA/isohexane = 5/95, flow rate = 1.0 mL/min, l = 210 nm) t_R = 9.01, 10.91.

Known compound. Data consistent with literature.[22]

4-(phenylsulfonyl)-2-butanol (S19)

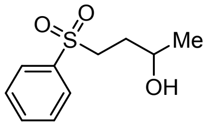

To a solution of methyl phenyl sulfone (781 mg, 5.00 mmol) in THF (25.0 mL) was added *n*-BuLi (3.125 mL, 5.00 mmol of a 1.60 M solution in hexanes) dropwise at −78 °C. The reaction mixture was stirred at −78 °C for 30 minutes. Propylene oxide (524 µL, 7.50 mmol) was added dropwise. The reaction mixture was then warmed to room temperature and stirred for 14 hours and 30 minutes, after which the reaction was quenched with NH_4Cl (10 mL of a saturated aqueous solution) at 0 °C and extracted with CH_2Cl_2 (3 × 20 mL). The combined organic fractions were washed with NH_4Cl (40 mL of a saturated aqueous solution), then brine (40 mL of a saturated aqueous solution), dried over $MgSO_4$ and concentrated. The crude residue was purified by flash column chromatography (SiO_2, 1:9 CH_2Cl_2:Et_2O) to afford the title compound as a colourless oil, which solidified on standing. (724 mg, 68%).

^{1}H NMR, ^{13}C{1H} NMR, IR, HRMS, m.p. and R_f match compound S6.

HPLC: The enantiomeric excess (racemic) was determined by chiral HPLC analysis. (Daicel Chiralcel OD-H, IPA/isohexane = 10/90, flow rate = 1.0 mL/min, l = 210 nm) t_R = 20.79, 24.16.

Data consistent with literature.[23]

1-((tert-butyldiphenylsilyl)oxy)propan-2-ol (S20)

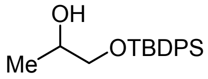

To a solution of 1,2-propane diol (367 µL, 5.00 mmol) and imidazole (781 mg, 6.50 mmol) in CH_2Cl_2 (25.0 mL) at 0 °C was added *tert*-Butyldiphenylchlorosilane (1.30 mL, 5.00 mmol) dropwise. The reaction mixture was slowly warmed to room temperature and stirred at room temperature for 16 hours. $NaHCO_3$ was added (10 mL of a saturated aqueous solution) and the mixture was vigorously stirred for 1 minute, before extracting with CH_2Cl_2 (3 x 15 mL). The combined organic fractions were washed with brine (20 mL of a saturated aqueous soltuion), dried over $MgSO_4$ and concentrated. The crude residue was purified by flash column chromatography (SiO_2, 1:9 Et_2O:pentane) to afford the title compound as a colourless oil that solidified on standing. (1.27 g, 81%).

^{1}H NMR, ^{13}C{1H} NMR, IR, HRMS, m.p. and R_f match compound S7.

HPLC: The enantiomeric excess (racemic) was determined by chiral HPLC analysis. (Daicel Chiralcel OD-H, IPA/isohexane = 10/90, flow rate = 1.0 mL/min, l = 210 nm) t_R = 4.39, 4.84.

Data consistent with literature.[24]

2-(2-hydroxypropyl)furan (S21)

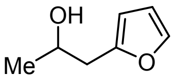

Prepared following literature method.[6]

To a vessel containing a solution of furan (727 μL, 10.0 mmol) in anhydrous THF (25 mL) at −78 °C under an atmosphere of argon was added dropwise *n*-BuLi (6.25 mL, 10.0 mmol of a 1.60 M solution in hexanes). After stirring for 30 minutes, propylene oxide (840 μL, 12.0 mmol) was added. The reaction was warmed to room temperature and left to stir for 16 hours. The reaction was quenched with NH_4Cl (16 mL of a saturated aqueous solution) and the aqueous phase extracted with ethyl acetate (2 × 15 mL). The organics were washed with brine (10 mL of a saturated aqueous solution), dried over $MgSO_4$, filtered and solvents were removed *in vacuo*. Purification by flash chromatography (SiO_2, 1:3 Et_2O:pentane) afforded title compound as a yellow liquid (587 mg, 4.70 mmol, 47%)

^{1}H NMR, ^{13}C{1H} NMR, IR, HRMS and R_f match compound S10.

HPLC: The enantiomeric excess (racemic) was determined by chiral HPLC analysis. (Lux A2, MeCN/water (0.1% v/v TFA) = 5/95, flow rate = 1.0 mL/min, l = 220 nm) t_R = 13.22, 13.86.

Data in accordance with literature.[25]

5-Phenylpent-4-yn-2-ol (S22)

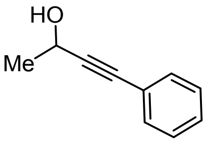

Prepared following literature method.[8]

To a stirred solution of phenylacetylene (1.10 mL, 10.0 mmol) in anhydrous THF (25 mL) at −78 °C was added dropwise *n*-BuLi (6.88 mL, 11.0 mmol of a 1.60 M solution in hexanes). After stirring 1 h at −78 °C, $BF_3{\cdot}OEt_2$ (1.48 mL, 12 mmol) was added dropwise and the stirring continued for another 15 min. Propylene oxide (1.05 mL, 15.0 mmol) was then added dropwise at −78 °C and stirring was continued for 3 hours at −78 °C after which the reaction was quenched with NH_4Cl (10 mL of a saturated aqueous solution). The mixture was extracted with ether (3 × 15 mL), then the combined organic layers were washed with saturated brine (15 mL of a saturated aqueous solution), dried over sodium sulfate, then the solvent removed in vacuo. Purification by flash chromatography (SiO_2, 80:20 pentane:Et_2O) afforded title compound as a yellow liquid (993 mg, 6.20 mmol, 62%)

^{1}H NMR, ^{13}C{1H} NMR, IR, HRMS and R_f match compound S11.

HPLC: The enantiomeric excess (racemic) was determined by chiral HPLC analysis. (Daicel Chiralcel OD-H, IPA/isohexane = 10/90, flow rate = 1.0 mL/min, l = 210 nm) t_R = 10.17, 15.62.

Data in accordance with literature.[26]

Primary Alcohols

Benzyl 3-hydroxy-2-methylpropanoate (S23)

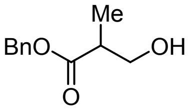

Prepared by mixing samples of (*R*)-Benzyl 3-hydroxy-2-methylpropanoate (**S8**) and (*S*)-Benzyl 3-hydroxy-2-methylpropanoate (**S9**).

HPLC: The enantiomeric excess (racemic) was determined by chiral HPLC analysis. (Daicel Chiralcel OD-H, IPA/isohexane = 10/90, flow rate = 1.0 mL/min, l = 210 nm) t_R = 8.30, 9.02.

Esters

(*S*)-octan-2-yl 4-nitrobenzoate (S24)

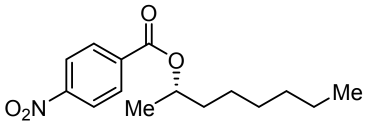

To a solution of (*S*)-2-octanol (795 µL, 5.00 mmol) in anhydrous THF (10 mL) was added NaH (204 mg, 5.10 mmol of a 60 wt.% suspension in mineral oil) in 5 portions at 0 °C and stirred (Caution: evolution of H_2 gas) until gas evolution ceased. 4-nitrobenzoylchloride (928 mg, 5.00 mmol) was added, and the mixture was warmed to room temperature, before stirring for 24 hours. H_2O (5 mL) was added at 0 °C, and the mixture was extracted with EtOAc (3 × 5 mL). The organics were washed with brine (5 mL of a saturated aqueous solution) and dried over $MgSO_4$. The solvent was removed *in vacuo* and the crude product purified by flash chromatography (SiO_2, 98:2 pentane:Et_2O) afforded pure title compound as a yellow liquid (271 mg, 1.00 mmol, 20%, *e.e.* >99%)

^{1}H NMR (400 MHz, $CDCl_3$) δ 8.32 – 8.25 (m, 2H, ArH), 8.24 – 8.17 (m, 2H, ArH), 5.24 – 5.13 (m, 1H, CH), 1.83 – 1.70 (m, 1H, CH_2), 1.68 – 1.57 (m, 1H, CH_2), 1.44 – 1.21 (m, 11H, CH_3, CH_2), 0.94 – 0.81 (m, 3H, CH_3).

^{13}C{^{1}H} NMR (101 MHz, $CDCl_3$) δ 164.5 (C_q), 150.6 (C_q), 136.5 (C_q), 130.8 (CH), 123.6 (CH), 73.3 (CH), 36.1 (CH_2), 31.9 (CH_2), 29.3 (CH_2), 25.5 (CH_2), 22.7 (CH_2), 20.1 (CH_3), 14.2 (CH_3).

IR (ATR, neat) (ν_{max} /cm^{-1}): 2958, 2930, 2857, 1719, 1607.

HRMS: (ESI) m/z: $[M + Na]^+$ Calcd for $NaC_{15}H_{21}O_4N^+$ 302.1363; Found 302.1365.

OR: $[\alpha]_D^{25}$: +28.0 (c. 1.0, $CHCl_3$)

HPLC: The enantiomeric excess (>99%) was determined by chiral HPLC analysis. (Daicel Chiralcel IC, IPA/isohexane = 2/98, flow rate = 1.0 mL/min, l = 210 nm) t_R = 8.89 (major), minor not observed.

R_f 0.22 (98:2 pentane:Et_2O)

Data in accordance with literature.[27]

Octan-2-yl 4-nitrobenzoate (S25)

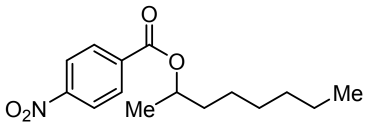

To a solution of 2-octanol (795 µL, 5.00 mmol) in anhydrous THF (10 mL) was added NaH (204 mg, 5.10 mmol of a 60 wt.% suspension in mineral oil) in 5 portions at 0 °C and stirred (Caution: evolution of H_2 gas) until gas evolution ceased. 4-nitrobenzoylchloride (928 mg, 5.00 mmol) was added, and the mixture was warmed to room temperature, before stirring for 24 hours. H_2O (5 mL) was added at 0 °C, and the mixture was extracted with EtOAc (3 × 5 mL). The organics were washed with brine (5 mL of a saturated aqueous solution) and dried over $MgSO_4$. Solvent was removed *in vacuo* and purification by flash chromatography (SiO_2, 98:2 pentane:Et_2O) afforded pure title compound as a yellow liquid (318 mg, 1.14 mmol, 23%).

^{1}H NMR, ^{13}C{1H} NMR, IR, HRMS and R_f match compound S24.

HPLC: The enantiomeric excess (racemic) was determined by chiral HPLC analysis. (Daicel Chiralcel IC, IPA/isohexane = 2/98, flow rate = 1.0 mL/min, l = 210 nm) t_R = 8.58, 9.27.

Data in accordance with literature.[28]

Malonate ester products

Diethyl 2-(1-(benzyloxy)propan-2-yl)malonate (S26)

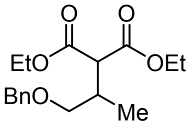

To a microwave tube was added anhydrous NaOTf (361 mg, 2.10 mmol) and anhydrous triphenylphosphine oxide **1** (279 mg, 1.00 mmol). The tube was then heated (heat gun) under high vacuum for 2.5 minutes, before filling and evacuating the tube with argon three times. Anhydrous THF (3 mL) was added, before oxalyl chloride (88.0 µL, 1.03 mmol) was added dropwise (Caution: slow addition due to evolution of CO_2 and CO gas). After stirring for 15 minutes at room temperature, anhydrous 1-(benzyloxy)propan-2-ol (156 µL, 1.00 mmol) was added, and the reaction was stirred for 4 hours at room temperature. After which NaDEM **3** (3.16 mL, 3.00 mmol, 3.00 equiv. of a 0.950 M solution in THF) was added, and the reaction was stirred for 16 hours at room temperature. The solvent was removed *in vacuo*, and purification by flash chromatography (SiO_2, 95:5 pentane:Et_2O) afforded title compound as a colourless liquid (25.2 mg, 0.08 mmol, 8%).

^{1}H NMR, ^{13}C{1H} NMR, IR, HRMS and R_f match compound 4a.

HPLC: The enantiomeric excess (racemic) was determined by chiral HPLC analysis. (Daicel Chiralcel IC, IPA/isohexane = 1/99, flow rate = 1.0 mL/min, l = 210 nm) t_R = 22.70, 24.80.

Data consistent with literature.[10]

Diethyl 2-(4-phenylbutan-2-yl)malonate (S27)

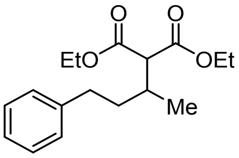

To a microwave tube was added anhydrous NaOTf (430 mg, 2.50 mmol) and anhydrous triphenylphosphine oxide **1** (334 mg, 1.20 mmol). The tube was then heated (heat gun) under high vacuum for 2.5 minutes, before filling with argon. The tube was evacuated and backfilled with argon a further two times. Anhydrous MeCN (3 mL) was added, before oxalyl chloride (105 µL, 1.23 mmol) was added dropwise (Caution: slow addition due to evolution of CO_2 and CO gas). After stirring for 30 minutes at room temperature, anhydrous 4-phenylbutan-2-ol (154 µL, 1.00 mmol) was added, and the reaction was stirred for 4 hours at room temperature. After which NaDEM **3** (3.16 mL, 3.00 mmol, 3.00 equiv. of a 0.950 M solution in THF) was added, and the reaction was stirred for 16 hours at room temperature. Upon completion, solvent was removed *in vacuo*, and purification by flash chromatography (SiO_2, 95:5 pentane:Et_2O) afforded title compound as a colourless liquid (223 mg, 0.76 mmol, 76%).

^{1}H NMR, ^{13}C{1H} NMR, IR, HRMS and R_f match compound 4b.

HPLC: The enantiomeric excess (racemic) was determined by chiral HPLC analysis. (Daicel Chiralcel IC, IPA/isohexane = 5/95, flow rate = 1.0 mL/min, l = 210 nm) t_R = 6.85, 7.31.

Data consistent with the literature.[29]

Diethyl 2-(1-phenylpropan-2-yl)malonate (S28)

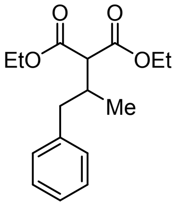

To a flame-dried microwave tube was added anhydrous NaOTf (365 mg, 2.12 mmol, 2.12 equiv.) and an anhydrous solution of tributylphosphine oxide (3.15 mL, 1.00 mmol of a 0.315 M solution in THF). Oxalyl chloride (88.0 µL, 1.03 mmol) was added dropwise (Caution: slow addition due to evolution of CO_2 and CO gas). After stirring for 10 minutes at room temperature, anhydrous 1-phenylpropan-2-ol (141 µL, 1.00 mmol) was added, and the reaction was stirred for a further 10 minutes at room temperature. After which NaDEM **3** (3.16 mL, 3.00 mmol, 3.00 equiv. of a 0.950 M solution in THF) was added, and the reaction was refluxed for 16 hours. Upon completion, solvent was removed *in vacuo*, and purification by flash chromatography (SiO_2, 90:10 pentane:Et_2O) afforded title compound as a colourless liquid (57.0 mg, 0.20 mmol, 20%).

^{1}H NMR, ^{13}C{1H} NMR, IR, HRMS and R_f match compound 4c.

HPLC: The enantiomeric excess (racemic) was determined by chiral HPLC analysis. (Daicel Chiralcel IC, IPA/isohexane = 2/98, flow rate = 1.0 mL/min, l = 210 nm) t_R = 8.13, 8.82.

Data consistent with literature.[12]

Dibenzyl 2-(octan-2-yl)malonate (S29)

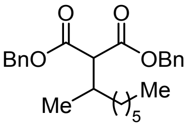

To a flame-dried microwave tube was added anhydrous NaOTf (145 mg, 2.12 mmol, 2.12 equiv.) and an anhydrous solution of tributylphosphine oxide (1.27 mL, 0.400 mmol of a 0.315 M solution in THF). Oxalyl chloride (35.0 µL, 0.408 mmol) was added dropwise (Caution: slow addition due to evolution of CO_2 and CO gas). After stirring for 10 minutes at room temperature, anhydrous 2-octanol (64.0 µL, 0.400 mmol) was added, and the reaction was stirred for a further 10 minutes at room temperature. After which sodium dibenzylmalonate (1.37 mL, 3.00 mmol of a 0.875 M solution in THF) was added, and the reaction was refluxed for 16 hours. Upon completion, solvent was removed *in vacuo*, and purification by flash chromatography (SiO_2, 90:10 pentane:Et_2O) afforded title compound as a colourless liquid (75.0 mg, 0.20 mmol, 20%).

^{1}H NMR, ^{13}C{1H} NMR, IR, HRMS and R_f match compound 4e.

SFC: The enantiomeric excess (racemic) was determined by chiral SFC analysis. (Chiralpak IG, IPA/CO_2 = 10/90, flow rate = 3.0 mL/min, l = 210 nm) t_R = 4.59, 5.17.

Diethyl 2-(1-((tert-butyldiphenylsilyl)oxy)propan-2-yl)malonate (S30)

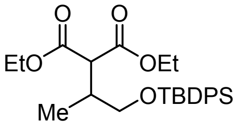

Following general procedure B. Purification by flash chromatography (SiO_2, 95:5 pentane:Et_2O) afforded title compound as a colourless liquid (64.0 mg, 0.140 mmol, 14%).

^{1}H NMR, ^{13}C{1H} NMR, IR, HRMS and R_f match compound 4i.

HPLC: The enantiomeric excess (racemic) was determined by chiral HPLC analysis. (Daicel Chiralcel AD-H, IPA/isohexane = 5/95, flow rate = 1.0 mL/min, l = 210 nm) t_R = 3.72, 4.14.

Diethyl 2-(4-(phenylsulfonyl)butan-2-yl)malonate (S31)

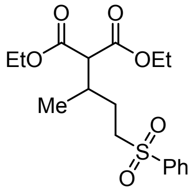

Following general procedure B. Purification by flash chromatography (SiO_2, 70:30 cyclohexane:EtOAc) afforded title compound as a yellow liquid (250 mg, 0.70 mmol, 70%)

^{1}H NMR, ^{13}C{1H} NMR, IR, HRMS and R_f match compound 4d.

HPLC: The enantiomeric excess (racemic) was determined by chiral HPLC analysis. (Daicel Chiralcel AS-H, IPA/isohexane = 10/90, flow rate = 1.0 mL/min, l = 210 nm) t_R = 38.09, 48.30.

3-benzyl 1,1-diethyl butane-1,1,3-tricarboxylate (S32)

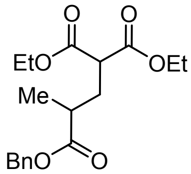

Following general procedure A. Purification by flash chromatography (SiO_2, 93:7 pentane:Et_2O) afforded title compound as a colourless liquid (225 mg, 0.67 mmol, 67%)

^{1}H NMR, ^{13}C{1H} NMR, IR, HRMS and R_f match compound 4q.

HPLC: The enantiomeric excess (racemic) was determined by chiral HPLC analysis. (Daicel Chiralcel IC, IPA/isohexane = 5/95, flow rate = 1.0 mL/min, l = 210 nm) t_R = 22.93, 24.40.

Diethyl 2-(5-phenylpent-4-yn-2-yl)malonate (S33)

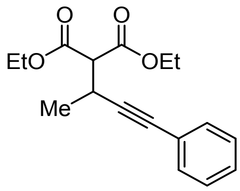

Following general procedure A. Purification by flash chromatography (SiO_2, 93:7 pentane:Et_2O) afforded title compound as a colourless liquid (168 mg, 0.560 mmol, 56%)

^{1}H NMR, ^{13}C{1H} NMR, IR, HRMS and R_f match compound 4g.

SFC: The enantiomeric excess (racemic) was determined by chiral SFC analysis. (Chiralpak IG, methanol (0.1% v/v NH_3)/CO_2 = 5/95, flow rate = 3.0 mL/min, l = 210-400 nm) t_R = 3.23, 3.70.

Diethyl 2-(1-(furan-2-yl)propan-2-yl)malonate (S34)

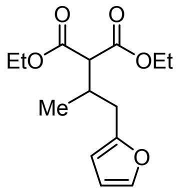

Following general procedure A. Purification by flash chromatography (SiO_2, 93:7 pentane:Et_2O) afforded title compound as a colourless liquid (176 mg, 0.660 mmol, 66%)

^{1}H NMR, ^{13}C{1H} NMR, IR, HRMS and R_f match compound 4h.

HPLC: The enantiomeric excess (racemic) was determined by chiral HPLC analysis. (Daicel Chiralcel OD-H, IPA/isohexane = 5/95, flow rate = 1.0 mL/min, l = 210 nm) t_R = 5.12, 5.68.

Alternative nucleophile products

2-(4-phenylbutan-2-yl)malononitrile (S35)

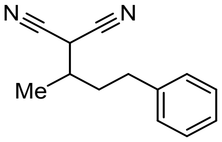

To a microwave tube was added anhydrous NaOTf (430 mg, 2.50 mmol) and anhydrous triphenylphosphine oxide **1** (334 mg, 1.20 mmol), then heated (heat gun) under high vacuum for 2.5 minutes, before filling with argon. The tube was evacuated and backfilled with argon a further two times. Anhydrous THF (1 mL) and MeCN (2 mL) were added. Oxalyl chloride (105 µL, 1.23 mmol) was added dropwise (Caution: evolution of CO_2 and CO gas) and then the mixture was stirred at room temperature for 30 minutes. Anhydrous (*R*)-(-)-4-Phenylbutan-2-ol (154 µL, 1.00 mmol) was added, and the mixture was stirred at room temperature for 4 hours. The solution was cooled to 0 °C, sodium hydride (120 mg, 3.00 mmol of a 60 wt.% suspension in mineral oil) was added, before malononitrile (198 mg, 3.00 mmol) was added in 5 portions (Caution: evolution of H_2 gas) before warming to room temperature and stirring for 16 hours. The solvent was removed *in vacuo*. Purification by flash chromatography (SiO_2, 93:7 pentane:Et_2O) resulted the title compound as a light yellow liquid (148 mg, 0.750 mmol, 75%).

^{1}H NMR, ^{13}C{1H} NMR, IR, HRMS and R_f match compound 4l.

HPLC: The enantiomeric excess (racemic) was determined by chiral HPLC analysis. (Daicel Chiralcel OD-H, IPA/isohexane = 5/95, flow rate = 1.0 mL/min, l = 210 nm) t_R = 32.32, 45.23.

Data consistent with literature.[20]

(2-methyl-4-phenylbutane-1,1-diyldisulfonyl)dibenzene (S36)

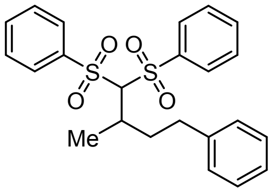

To a microwave tube was added anhydrous NaOTf (430 mg, 2.50 mmol) and anhydrous triphenylphosphine oxide **1** (334 mg, 1.20 mmol), then heated (heat gun) under high vacuum for 2.5 minutes, before filling with argon. The tube was evacuated and backfilled with argon a further two times. Anhydrous THF (1 mL) and MeCN (2 mL) were added. Oxalyl chloride (105 µL, 1.23 mmol) was added (Caution: evolution of CO_2 and CO gas) and then the mixture was stirred at room temperature for 15 minutes. Anhydrous phenylbutan-2-ol (154 µL, 1.00 mmol) was added in one portion, and the mixture was stirred at room temperature for 4 hours. To the reaction was added potassium bis(phenylsulfonyl)methane (1.00 g, 3.00 mmol) and anhydrous 18-crown-6 (793 mg, 3.00 mmol) in 1:1 MeCN:THF (6 mL)and the reaction was stirred at room temperature for 16 hours. The solvent was removed in

vacuo. Purification by flash chromatography (SiO_2, 70:30 cyclohexane:EtOAc) resulted the title compound as a colourless solid (197 mg, 0.460 mmol, 46%).

^{1}H NMR, ^{13}C{1H} NMR, IR, HRMS and R_f match compound 4k.

HPLC: The enantiomeric excess (racemic) was determined by chiral HPLC analysis. (Daicel Chiralcel AD-H, IPA/isohexane = 3/97, flow rate = 1.0 mL/min, l = 254 nm) t_R = 21.52, 25.66.

((2-chloropropoxy)methyl)benzene (S37)

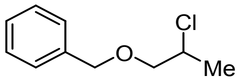

To a microwave tube was added triphenylphosphine oxide **1** (279 mg, 1.00 mmol) and THF (3 mL). To this mixture was added oxalyl chloride dropwise (88.0 µL, 1.03 mmol) (Caution: slow addition due to formation of CO and CO_2 gas) and the mixture was stirred for 30 minutes at room temperature. 1-(benzyloxy)propan-2-ol (155.6 µL, 1.00 mmol) was added and the mixture was stirred at room temperature for 5 hours. Solvent was removed *in vacuo* and purification by flash chromatography (SiO_2, 95:5 pentane:Et_2O) afforded pure title compound as a colourless liquid (158 mg, 0.860 mmol, 86%).

^{1}H NMR, ^{13}C{1H} NMR, IR, HRMS and R_f match compound 2.

HPLC: The enantiomeric excess (racemic) was determined by chiral HPLC analysis. (Daicel Chiralcel OD-H, IPA/isohexane = 2/98, flow rate = 1.0 mL/min, l = 210 nm) t_R = 5.24, 5.69.

Data consistent with literature.[21]

X-ray crystallography

Single Crystal X-ray diffraction experimental method

A single crystal of **5b** was selected and mounted using Fomblin® (YR-1800 perfluoropolyether oil) on a polymer-tipped MiTeGen MicroMountTM and cooled rapidly to 120 K in a stream of cold N_2 using an Oxford Cryosystems open flow cryostat.[30] Single crystal X-ray diffraction data were collected on an Oxford Diffraction GV1000 (TitanS2 CCD area detector, mirror-monochromated Cu-Kα radiation source; λ = 1.54184 Å, ω scans). Cell parameters were refined from the observed positions of all strong reflections and absorption corrections were applied using a Gaussian numerical method with beam profile correction (CrysAlisPro).[31] Structures were solved within Olex2[32] by dual space iterative methods (SHELXT)[33] and all non-hydrogen atoms refined by full-matrix least-squares on all unique F2 values with anisotropic displacement parameters (SHELXL).[34] Hydrogen atoms were refined with constrained riding geometries and thermal parameters linked to Uiso of their parent atoms. Hydrogen atoms were refined with constrained riding geometries and thermal parameters linked to Uiso their parent atoms). The structure was checked with checkCIF.[35] CCDC-2456643 contains the supplementary data for the compound. The data can be obtained free of charge from The Cambridge Crystallographic Data Centre via www.ccdc.cam.ac.uk/data_request/cif.

Experimental details for crystal structure 5b ($C_{138}H_{116}B_2F_{48}O_4P_2$; M =2833.86 g/mol): triclinic, space group $P1$ (no. 1), a = 12.9831(3) Å, b = 13.1109(3) Å, c = 20.4586(6) Å, α = 80.427(2)°, β = 78.106(2)°, γ = 78.993(2)°, V = 3315.67(15) Å^3, Z = 1, T = 120(2) K, μ(Cu Kα) = 1.371 mm^{-1}, $Dcalc$ = 1.419 g/cm^3, 48314 reflections measured (6.93° ≤ 2Θ ≤ 139.97°), 23016 unique (R_{int} = 0.0287, R_{sigma} = 0.0362) which were used in all calculations. The final R_1 was 0.0658 (I > 2σ(I)) and wR_2 was 0.1913 (all data).

Single crystal X-ray diffraction refinement experimental details

Several crystals of the compound were screened for diffraction; all of them were observed to exhibit a minor twin component whose diffraction pattern overlapped partially with that of the main component. To reduce the extent of pattern peak overlaps between the two patterns a collection strategy with an extended detector distance of 90 mm was selected for data collection. Although the peaks of the two patterns could be indexed, attempts to perform a

twin data reduction on overlapping patterns resulted in poorer intensity and internal consistency statistics than those obtained performing a data reduction on only the major twin component. After the event twinning handling of the data using the PLATON routine TwinRotMat indicated a twin law with a twin fraction of 0.19. Attempts to refine the data using a PLATON generated HKLF5 reflection file or with direct use of the twin law and a batch scale factor with the HKLF4 reflection file resulted in no improvements in R1 and nonsensical batch scale factors, hence, were not pursued.

Six of the trifluoromethane moieties are conformationally disordered with their fluorine atoms split over two sets of positions. In each case occupancies of the pairs of positions are refined and constrained to sum to unity giving values for the major components in the range 0.51-0.69. The geometries of all the trifluoromethane moieties in the structure were geometrically restrained have similar C-F and F···F distances (SADI and SAME). Rigid bond and similarity restraints were applied to all the fluorine atoms and carbon atoms attached to disordered fluorine atoms in the structure (RIGU, SIMU).

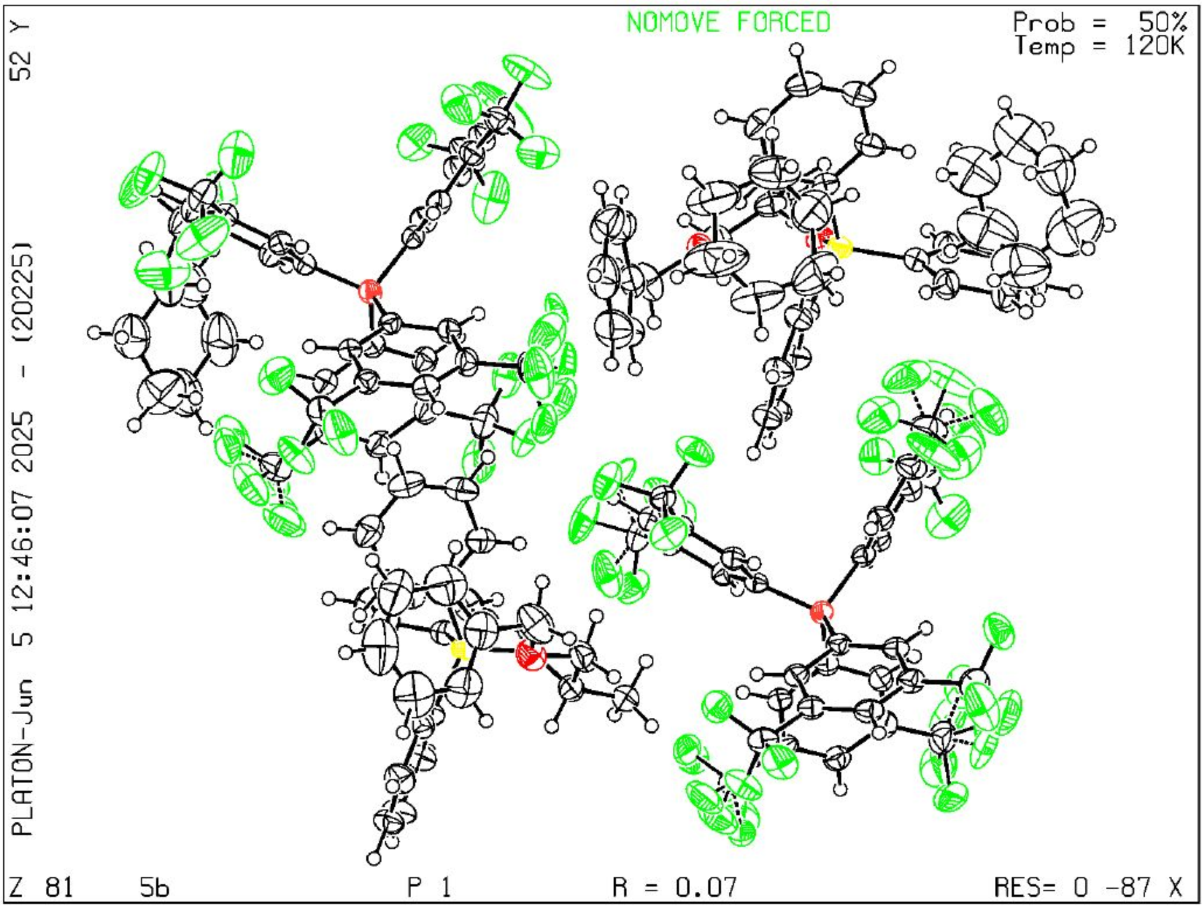

*Figure S2: Thermal ellipsoid plot for alkoxyphosphonium salt **5b**. Probability levels 50%.*

HPLC & SFC Chromatograms

Alcohols

1-benzyloxy-2-propanol (S18)

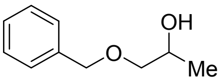

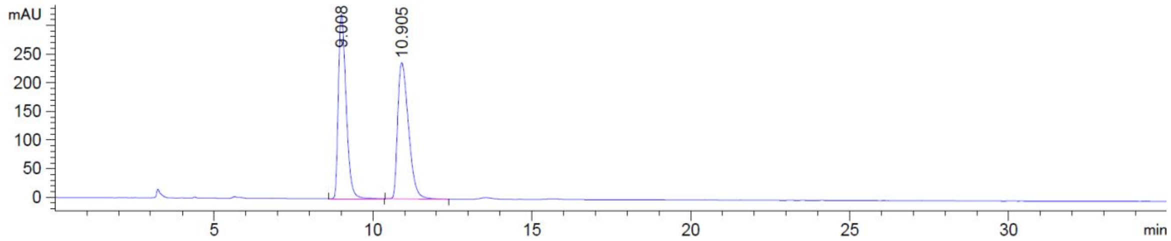

Signal 2: DAD1 B, Sig=210,4 Ref=360,100

Peak #	RetTime [min]	Type	Width [min]	Area [mAU*s]	Height [mAU]	Area %
1	9.008	BB	0.2636	5440.61182	321.20355	49.3029
2	10.905	BB	0.3709	5594.46875	237.44821	50.6971

Totals : 1.10351e4 558.65176

(*S*)-1-(benzyloxy)propan-2-ol (A1)

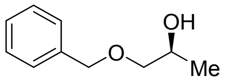

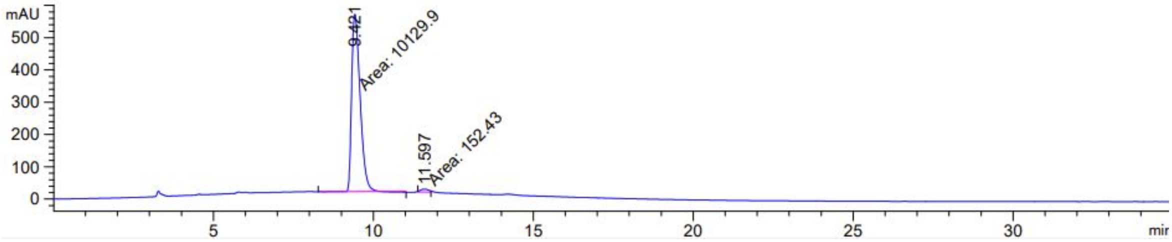

Signal 2: DAD1 B, Sig=210,4 Ref=360,100

Peak #	RetTime [min]	Type	Width [min]	Area [mAU*s]	Height [mAU]	Area %
1	9.421	MM	0.3072	1.01299e4	549.49915	98.5176
2	11.597	MM	0.2912	152.42996	8.72362	1.4824

Totals : 1.02823e4 558.22276

4-phenylbutan-2-ol (S17)

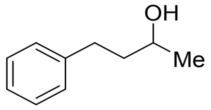

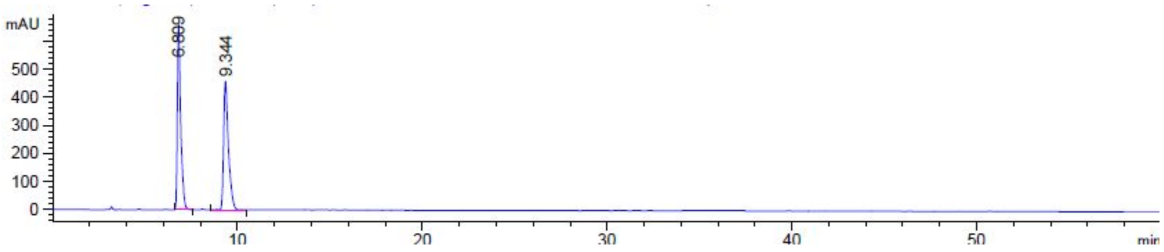

Signal 2: DAD1 B, Sig=210,4 Ref=360,100

Peak #	RetTime [min]	Type	Width [min]	Area [mAU*s]	Height [mAU]	Area %
1	6.809	BB	0.1852	8306.52637	658.29535	50.1886
2	9.344	BB	0.2646	8244.08203	456.59863	49.8114
Totals :				1.65506e4	1114.89398	

(*R*)-4-phenylbutan-2-ol (A2)

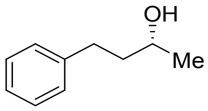

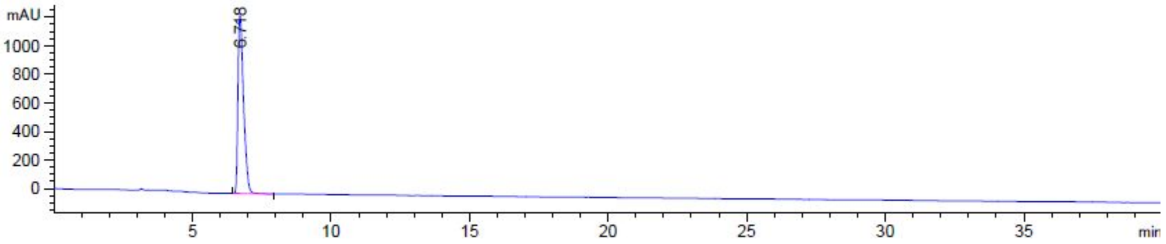

Signal 2: DAD1 B, Sig=210,4 Ref=360,100

Peak #	RetTime [min]	Type	Width [min]	Area [mAU*s]	Height [mAU]	Area %
1	6.718	BB	0.2101	1.75490e4	1247.36328	100.0000
Totals :				1.75490e4	1247.36328	

1-phenylpropan-2-ol (S16)

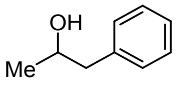

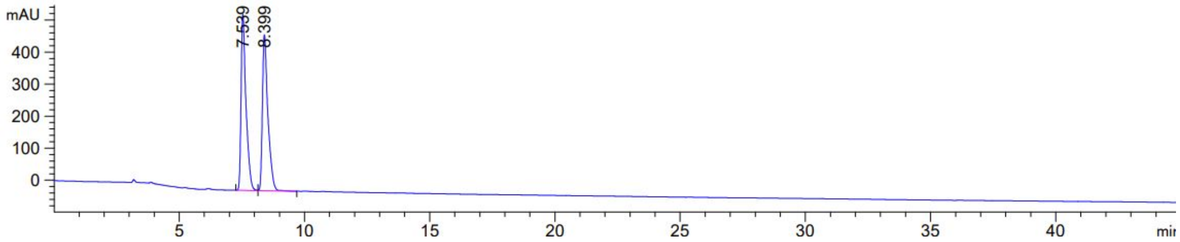

Signal 2: DAD1 B, Sig=210,4 Ref=360,100

Peak #	RetTime [min]	Type	Width [min]	Area [mAU*s]	Height [mAU]	Area %
1	7.539	BV	0.1992	7489.02295	548.97479	50.1900
2	8.399	VB	0.2220	7432.32666	487.06296	49.8100
Totals :				1.49213e4	1036.03775	

(*R*)-1-phenylpropan-2-ol (S3)

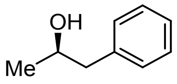

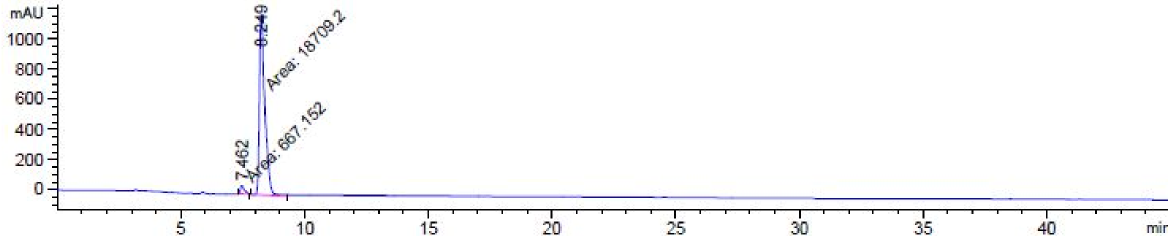

Signal 2: DAD1 B, Sig=210,4 Ref=360,100

Peak #	RetTime [min]	Type	Width [min]	Area [mAU*s]	Height [mAU]	Area %
1	7.462	MM	0.2014	667.15204	55.21366	3.4431
2	8.249	MM	0.2588	1.87092e4	1204.66565	96.5569

Totals : 1.93763e4 1259.87931

(*S*)-1-phenylpropan-2-ol (S4)

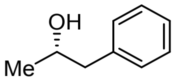

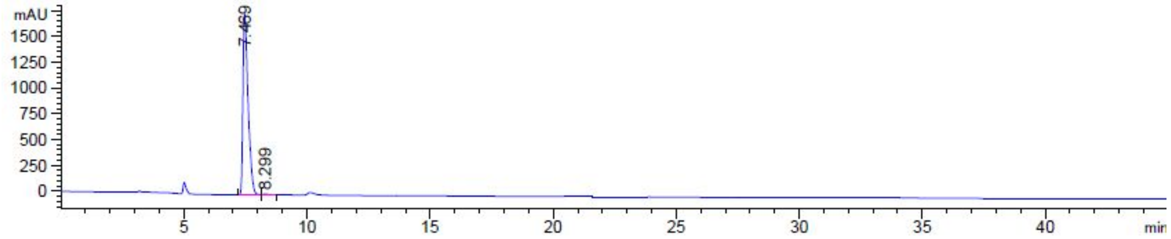

Signal 2: DAD1 B, Sig=210,4 Ref=360,100

Peak #	RetTime [min]	Type	Width [min]	Area [mAU*s]	Height [mAU]	Area %
1	7.469	BV	0.2159	2.54977e4	1751.14587	99.6189
2	8.299	VB	0.2167	97.53960	6.59009	0.3811

Totals : 2.55953e4 1757.73597

4-(phenylsulfonyl)-2-butanol (S19)

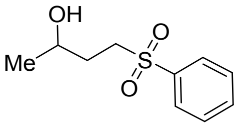

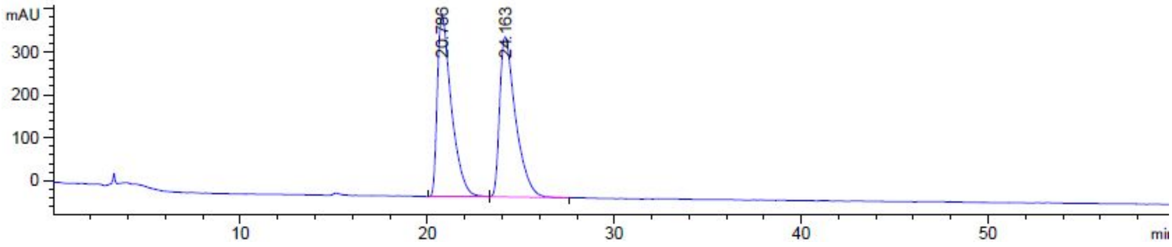

Signal 2: DAD1 B, Sig=210,4 Ref=360,100

Peak #	RetTime [min]	Type	Width [min]	Area [mAU*s]	Height [mAU]	Area %
1	20.786	BB	0.7829	2.20613e4	421.31433	50.0158
2	24.163	BB	0.8830	2.20474e4	372.21909	49.9842
Totals :				4.41087e4	793.53342	

(*R*)-(-)-4-(phenylsulfonyl)-2-butanol (S6)

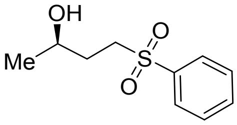

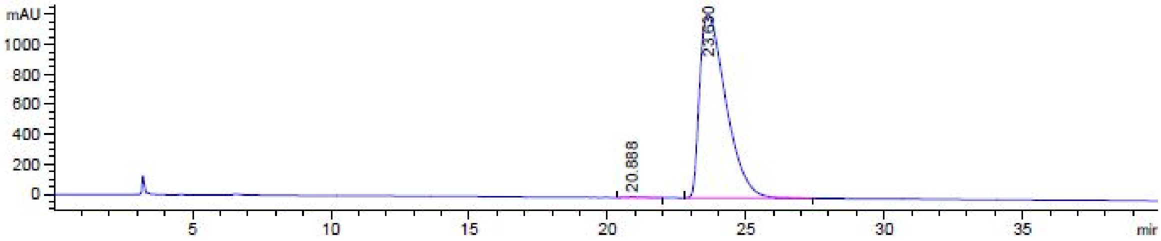

Signal 2: DAD1 B, Sig=210,4 Ref=360,100

Peak #	RetTime [min]	Type	Width [min]	Area [mAU*s]	Height [mAU]	Area %
1	20.888	BB	0.5580	232.73988	4.99163	0.2866
2	23.630	BB	0.9434	8.09832e4	1224.72009	99.7134
Totals :				8.12159e4	1229.71172	

1-((tert-butyldiphenylsilyl)oxy)propan-2-ol (S20)

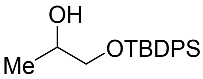

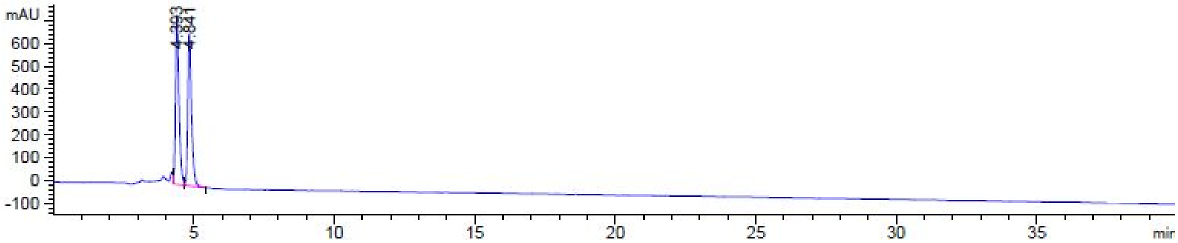

Signal 2: DAD1 B, Sig=210,4 Ref=360,100

Peak #	RetTime [min]	Type	Width [min]	Area [mAU*s]	Height [mAU]	Area %
1	4.393	VV	0.1265	6370.33984	739.46271	50.4976
2	4.841	VB	0.1396	6244.79639	664.89221	49.5024
Totals :				1.26151e4	1404.35492	

(*R*)-1-((tert-butyldiphenylsilyl)oxy)propan-2-ol (S7)

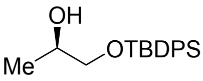

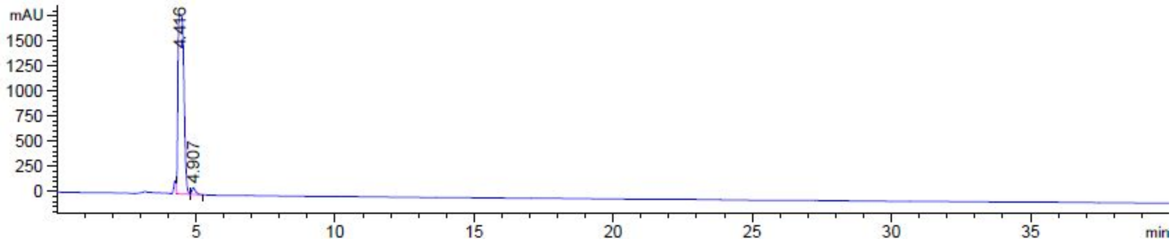

Signal 2: DAD1 B, Sig=210,4 Ref=360,100

Peak #	RetTime [min]	Type	Width [min]	Area [mAU*s]	Height [mAU]	Area %
1	4.416	VV	0.1944	2.62148e4	1774.30798	97.6264
2	4.907	VB	0.1415	637.36047	66.68542	2.3736

Totals : 2.68521e4 1840.99340

2-(2-hydroxypropyl)furan (S21)

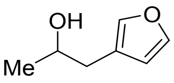

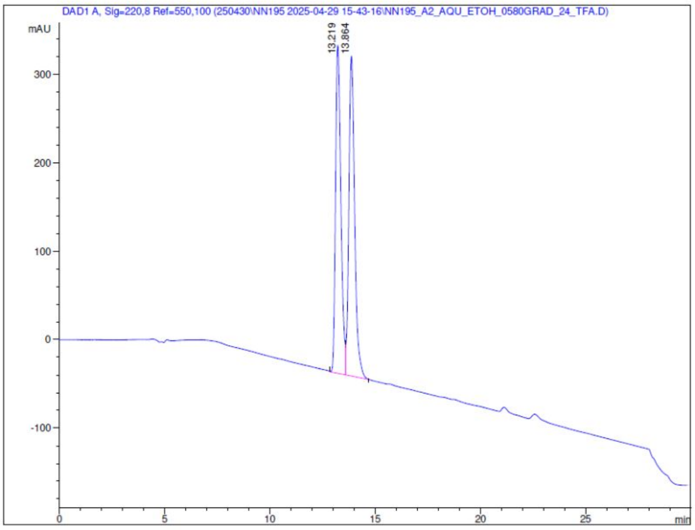

#	Meas. R	Height	Width	Area	Area %	Symmetr
1	13.219	370.869	0.295	6.561e3	48.617	0.713
2	13.864	361.515	0.320	6.934e3	51.383	0.739

(*S*)-2-(2-hydroxypropyl)furan (S10)

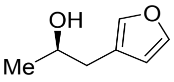

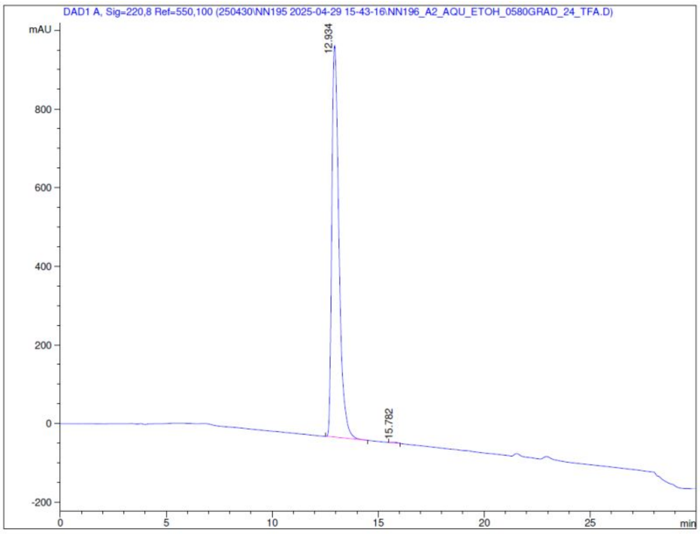

#	Meas. R	Height	Width	Area	Area %	Symmetr
1	12.934	996.833	0.365	2.342e4	99.817	0.597

5-Phenylpent-4-yn-2-ol (S22)

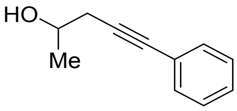

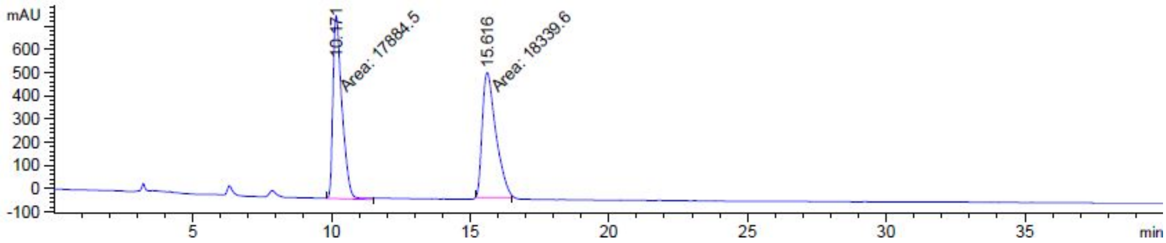

Signal 2: DAD1 B, Sig=210,4 Ref=360,100

Peak #	RetTime [min]	Type	Width [min]	Area [mAU*s]	Height [mAU]	Area %
1	10.171	MM	0.3780	1.78845e4	788.45850	49.3719
2	15.616	MM	0.5679	1.83396e4	538.26154	50.6281
Totals :				3.62241e4	1326.72003	

(*S*)-5-Phenylpent-4-yn-2-ol (S11)

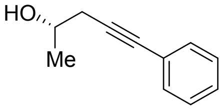

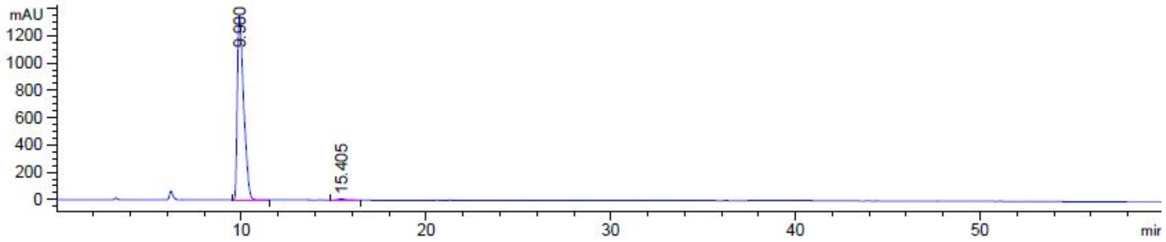

Signal 2: DAD1 B, Sig=210,4 Ref=360,100

Peak #	RetTime [min]	Type	Width [min]	Area [mAU*s]	Height [mAU]	Area %
1	9.900	BB	0.3545	3.22199e4	1349.49316	99.2509
2	15.405	BB	0.4401	243.18327	7.87821	0.7491

Totals : 3.24631e4 1357.37138

Benzyl 3-hydroxy-2-methylpropanoate (S23)

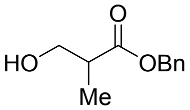

Prepared by combining samples of **S8** and **S9**.

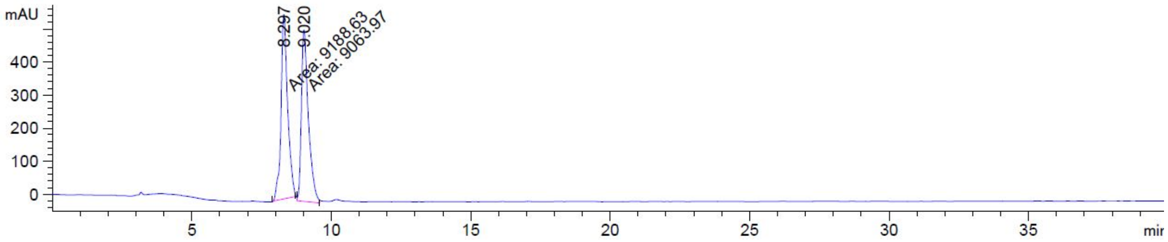

Signal 2: DAD1 B, Sig=210,4 Ref=360,100

Peak #	RetTime [min]	Type	Width [min]	Area [mAU*s]	Height [mAU]	Area %
1	8.297	MM	0.2743	9188.62695	558.40161	50.3415
2	9.020	MM	0.2908	9063.96973	519.46027	49.6585

Totals : 1.82526e4 1077.86188

(*R*)-Benzyl 3-hydroxy-2-methylpropanoate (S8)

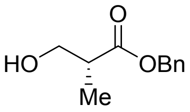

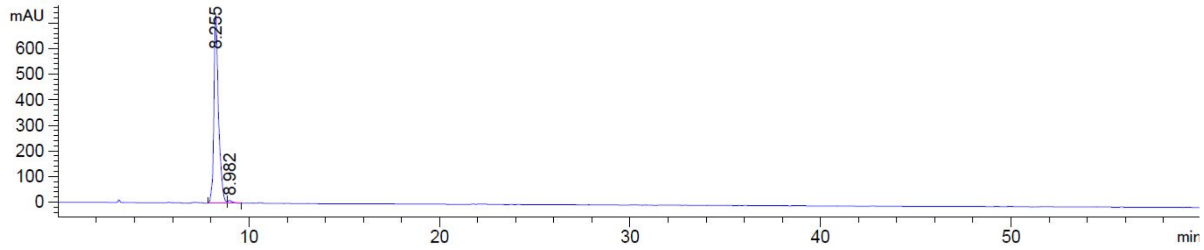

Signal 2: DAD1 B, Sig=210,4 Ref=360,100

Peak #	RetTime [min]	Type	Width [min]	Area [mAU*s]	Height [mAU]	Area %
1	8.255	BV	0.2440	1.23187e4	733.31628	98.6213
2	8.982	VB	0.2460	172.21304	10.04662	1.3787

Totals :				1.24909e4	743.36291	

(*S*)-Benzyl 3-hydroxy-2-methylpropanoate (S9)

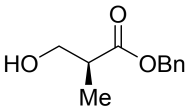

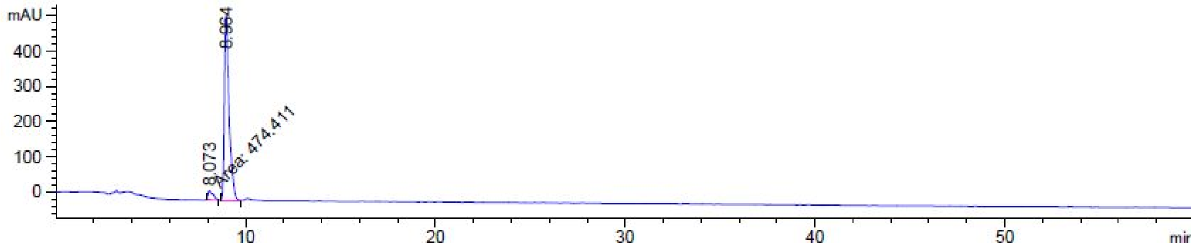

Signal 2: DAD1 B, Sig=210,4 Ref=360,100

Peak #	RetTime [min]	Type	Width [min]	Area [mAU*s]	Height [mAU]	Area %
1	8.073	MM	0.3106	474.41077	25.45915	4.9796
2	8.964	BB	0.2534	9052.73926	524.42560	95.0204

Totals :				9527.15002	549.88475	

Esters

Octan-2-yl 4-nitrobenzoate (S25)

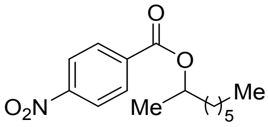

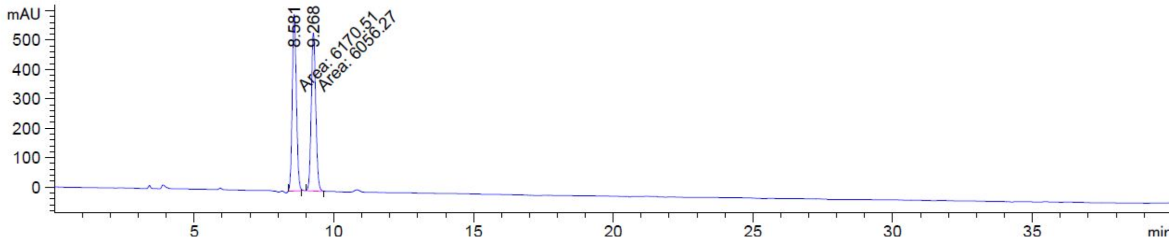

Signal 2: DAD1 B, Sig=210,4 Ref=360,100

Peak #	RetTime [min]	Type	Width [min]	Area [mAU*s]	Height [mAU]	Area %
1	8.581	MM	0.1710	6170.51172	601.37329	50.4672
2	9.268	MM	0.1877	6056.26953	537.63074	49.5328

Totals :				1.22268e4	1139.00403	

(S)-octan-2-yl 4-nitrobenzoate (S24)

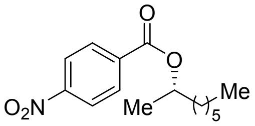

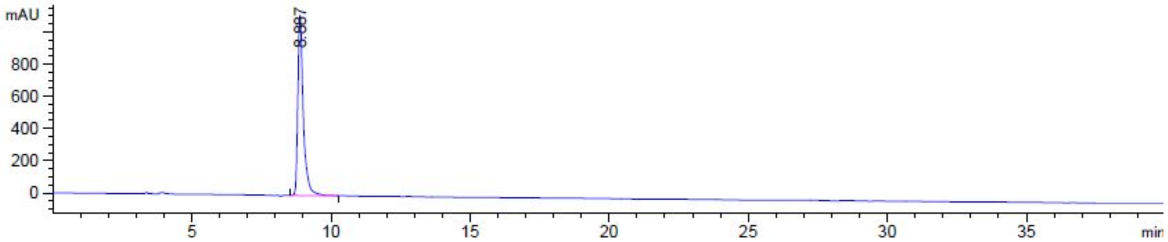

Signal 2: DAD1 B, Sig=210,4 Ref=360,100

Peak #	RetTime [min]	Type	Width [min]	Area [mAU*s]	Height [mAU]	Area %
1	8.887	BB	0.2064	1.53687e4	1117.62720	100.0000

Totals : 1.53687e4 1117.62720

Alkyl chlorides

(*R*)-((2-chloropropoxy)methyl)benzene (2)

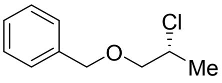

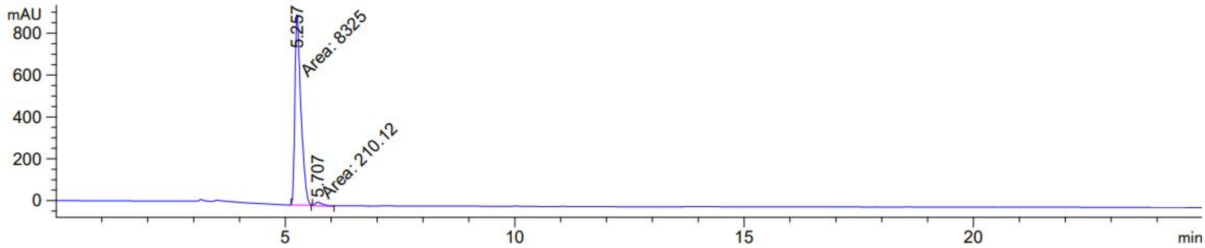

Signal 2: DAD1 B, Sig=210,4 Ref=360,100

Peak #	RetTime [min]	Type	Width [min]	Area [mAU*s]	Height [mAU]	Area %
1	5.257	MM	0.1522	8325.00488	911.57477	97.5382
2	5.707	MM	0.1941	210.12013	18.04121	2.4618

Totals : 8535.12502 929.61598

((2-chloropropoxy)methyl)benzene (S37)

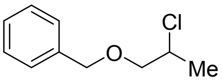

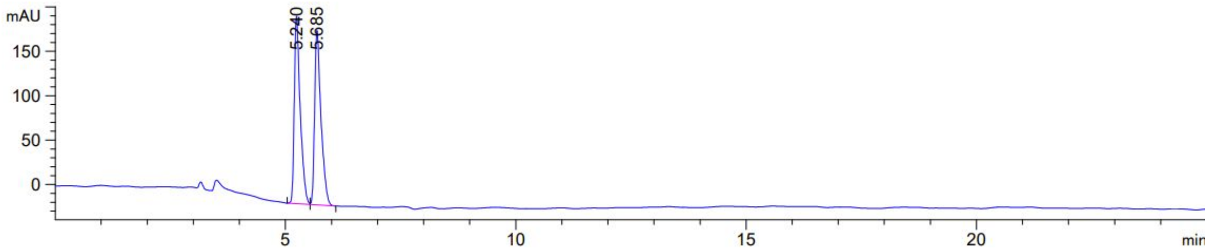

Signal 2: DAD1 B, Sig=210,4 Ref=360,100

Peak #	RetTime [min]	Type	Width [min]	Area [mAU*s]	Height [mAU]	Area %
1	5.240	BV	0.1304	1891.01880	211.27943	49.9821
2	5.685	VB	0.1404	1892.37231	196.48100	50.0179
Totals :				3783.39111	407.76044	

Products

Diethyl 2-(4-phenylbutan-2-yl)malonate (S27)

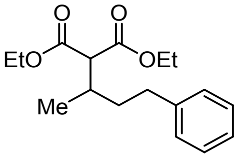

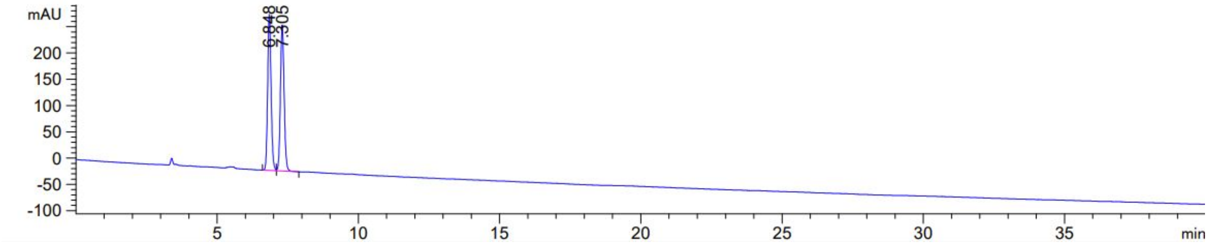

Signal 2: DAD1 B, Sig=210,4 Ref=360,100

Peak #	RetTime [min]	Type	Width [min]	Area [mAU*s]	Height [mAU]	Area %
1	6.848	BV	0.1310	2516.67334	296.67490	49.8348
2	7.305	VB	0.1402	2533.35889	278.20706	50.1652
Totals :				5050.03223	574.88196	

(*S*)-Diethyl 2-(4-phenylbutan-2-yl)malonate (4b)

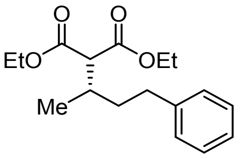

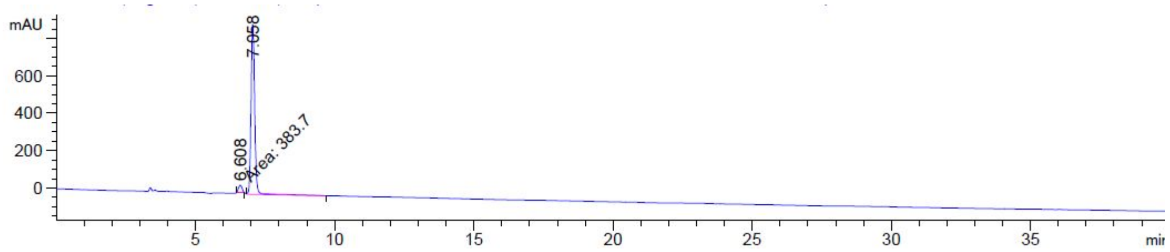

Signal 2: DAD1 B, Sig=210,4 Ref=360,100

Peak #	RetTime [min]	Type	Width [min]	Area [mAU*s]	Height [mAU]	Area %
1	6.608	MM	0.1562	383.70020	40.94417	4.4283
2	7.058	VB	0.1404	8280.93652	908.19604	95.5717

Totals : 8664.63672 949.14022

Diethyl 2-(1-(benzyloxy)propan-2-yl)malonate (S26)

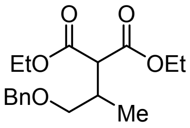

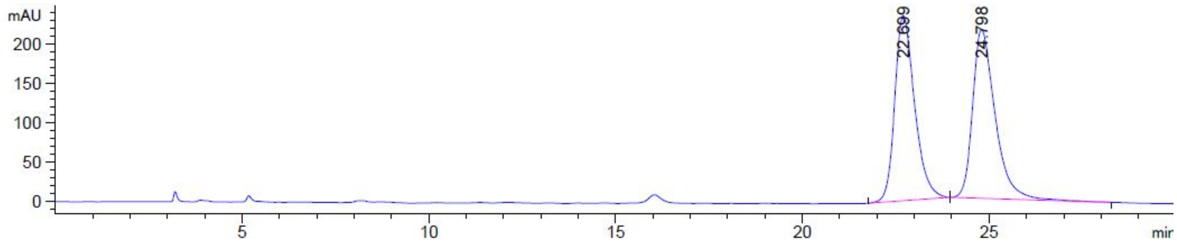

Signal 2: DAD1 B, Sig=210,4 Ref=360,100

Peak #	RetTime [min]	Type	Width [min]	Area [mAU*s]	Height [mAU]	Area %
1	22.699	BB	0.5632	8731.38965	236.21198	49.1976
2	24.798	BBA	0.6290	9016.19629	215.08981	50.8024

Totals : 1.77476e4 451.30179

(*R*)-Diethyl 2-(1-(benzyloxy)propan-2-yl)malonate (4a)

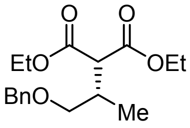

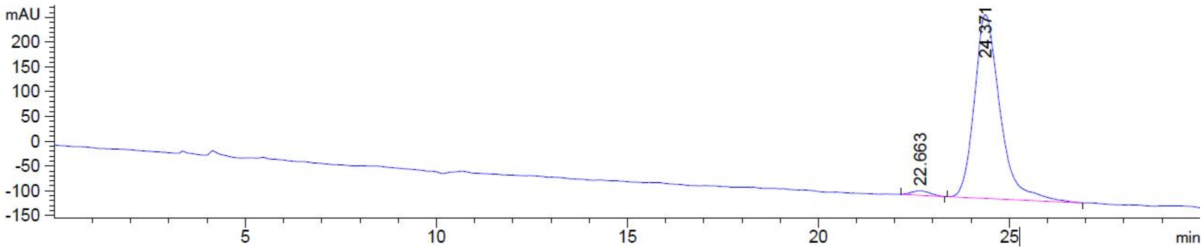

Signal 2: DAD1 B, Sig=210,4 Ref=360,100

Peak #	RetTime [min]	Type	Width [min]	Area [mAU*s]	Height [mAU]	Area %
1	22.663	BB	0.4136	312.09784	9.15871	1.7659
2	24.371	BB	0.6796	1.73612e4	371.21622	98.2341

Totals : 1.76733e4 380.37493

Diethyl 2-(1-phenylpropan-2-yl)malonate (S28)

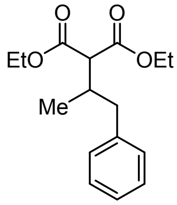

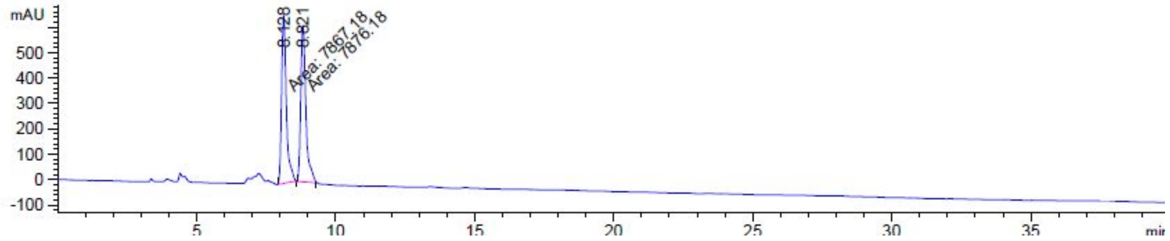

Signal 2: DAD1 B, Sig=210,4 Ref=360,100

Peak #	RetTime [min]	Type	Width [min]	Area [mAU*s]	Height [mAU]	Area %
1	8.128	MM	0.1982	7867.17969	661.63434	49.9714
2	8.821	MM	0.2137	7876.17529	614.26904	50.0286
Totals :				1.57434e4	1275.90338	

(*S*)-Diethyl 2-(1-phenylpropan-2-yl)malonate (4c)

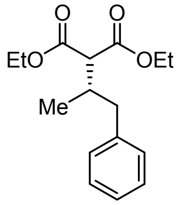

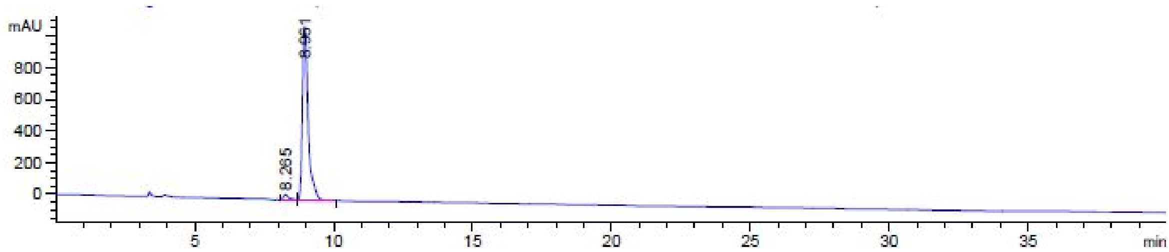

Signal 2: DAD1 B, Sig=210,4 Ref=360,100

Peak #	RetTime [min]	Type	Width [min]	Area [mAU*s]	Height [mAU]	Area %
1	8.265	BV	0.2145	539.19659	36.88721	3.2848
2	8.961	VB	0.2183	1.58755e4	1100.75818	96.7152
Totals :				1.64147e4	1137.64539	

Dibenzyl 2-(octan-2-yl)malonate (S29)

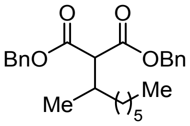

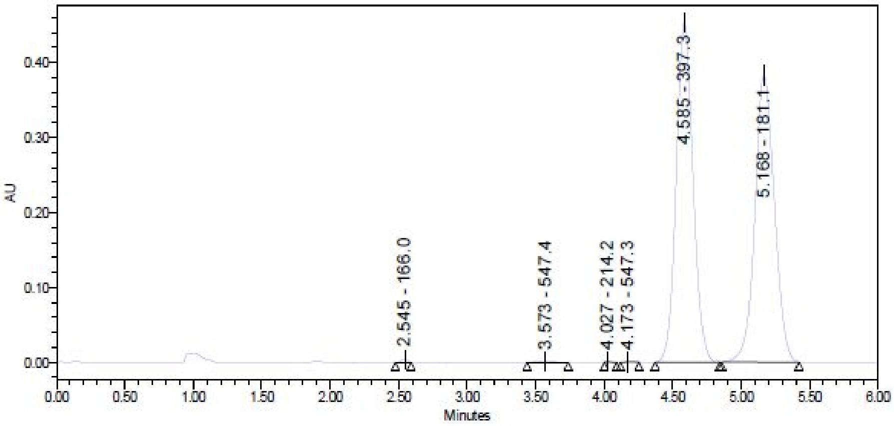

Peak Results

	Retention Time (min)	Area (µV*sec)	% Area	Width @ 50%
1	2.55	11098	0.1	0.04970
2	3.57	9511	0.1	0.13076
3	4.03	1267	0.0	0.04539
4	4.17	2437	0.0	0.08394
5	4.58	3861487	50.3	0.13095
6	5.17	3797314	49.4	0.15126

(*R*)-Dibenzyl 2-(octan-2-yl)malonate (4e)

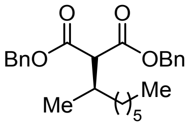

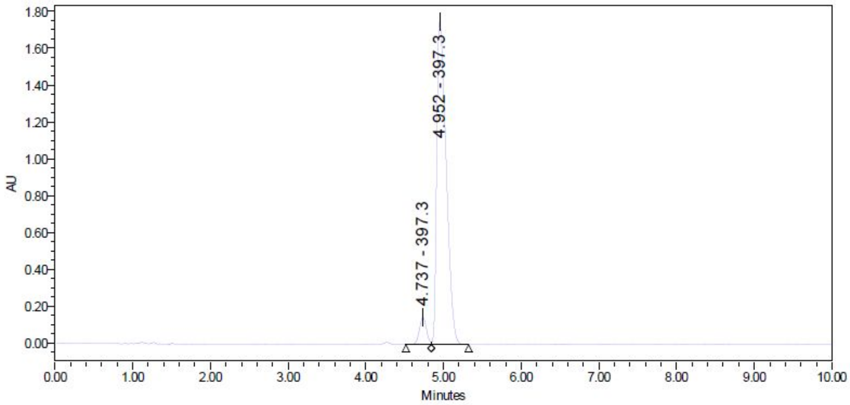

Peak Results

	Retention Time (min)	Area (µV*sec)	% Area	Width @ 50%	Resolution
1	4.74	949168	6.0	0.09885	
2	4.95	14851196	94.0	0.13466	1.1

Diethyl 2-(1-((tert-butyldiphenylsilyl)oxy)propan-2-yl)malonate (S30)

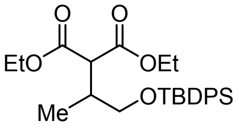

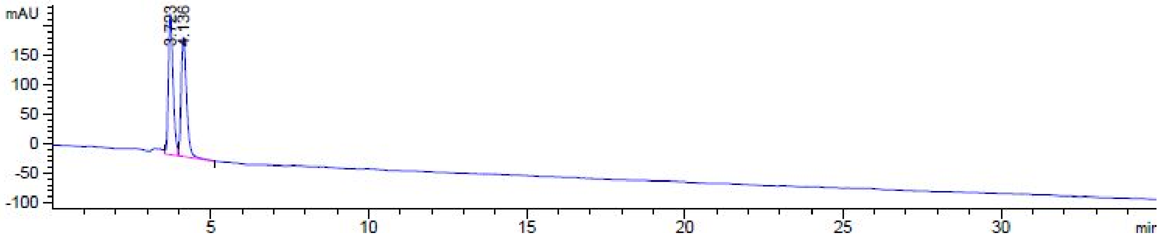

Signal 2: DAD1 B, Sig=210,4 Ref=360,100

Peak #	RetTime [min]	Type	Width [min]	Area [mAU*s]	Height [mAU]	Area %
1	3.723	VV	0.1626	2494.19971	237.35750	50.8035
2	4.136	VB	0.1839	2415.30420	201.40694	49.1965

Totals : 4909.50391 438.76443

(*R*)-Diethyl 2-(1-((tert-butyldiphenylsilyl)oxy)propan-2-yl)malonate (4i)

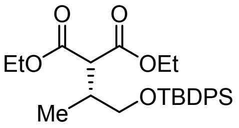

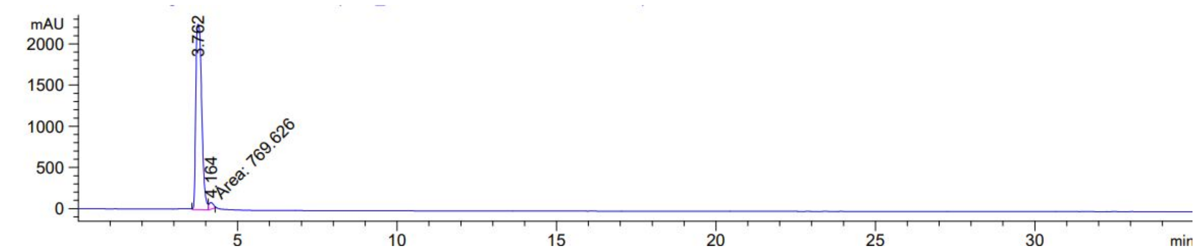

Signal 2: DAD1 B, Sig=210,4 Ref=360,100

Peak #	RetTime [min]	Type	Width [min]	Area [mAU*s]	Height [mAU]	Area %
1	3.762	VV	0.2039	2.88785e4	2247.88818	97.4041
2	4.164	MM	0.1588	769.62555	80.79473	2.5959

Totals : 2.96481e4 2328.68291

Diethyl 2-(4-(phenylsulfonyl)butan-2-yl)malonate (S31)

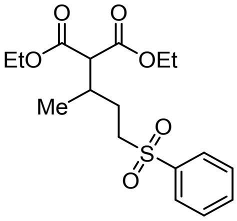

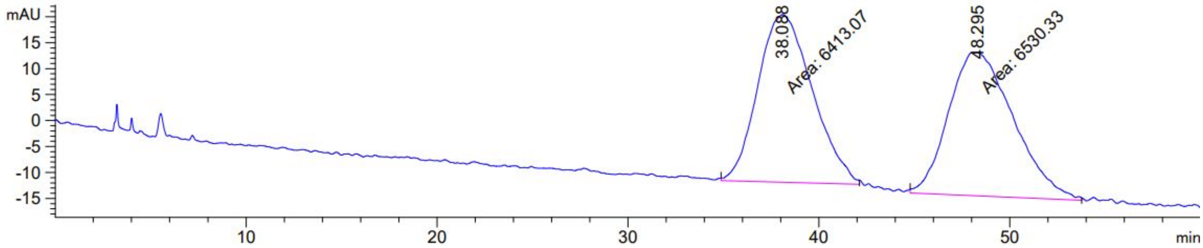

Signal 2: DAD1 B, Sig=210,4 Ref=360,100

Peak #	RetTime [min]	Type	Width [min]	Area [mAU*s]	Height [mAU]	Area %
1	38.088	MM	3.3114	6413.06592	32.27819	49.5470
2	48.295	MM	3.9358	6530.32813	27.65342	50.4530
Totals :				1.29434e4	59.93161	

(*S*)-Diethyl 2-(4-(phenylsulfonyl)butan-2-yl)malonate (4d)

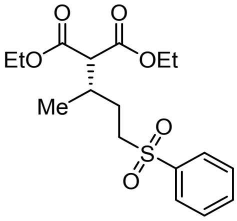

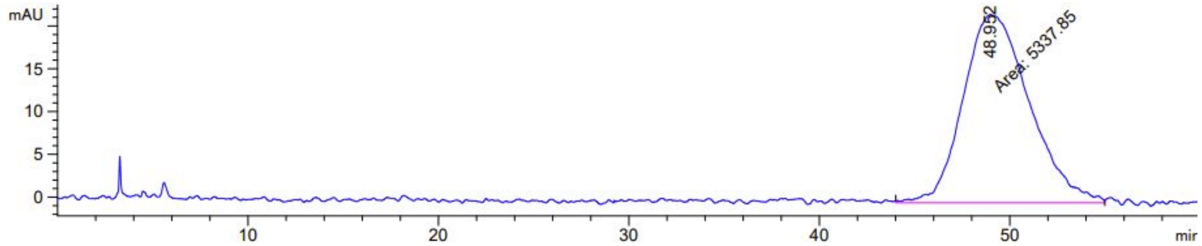

Signal 2: DAD1 B, Sig=210,4 Ref=360,100

Peak #	RetTime [min]	Type	Width [min]	Area [mAU*s]	Height [mAU]	Area %
1	48.952	MM	4.0414	5337.85059	22.01326	100.0000
Totals :				5337.85059	22.01326	

Diethyl 2-(5-phenylpent-4-yn-2-yl)malonate (S33)

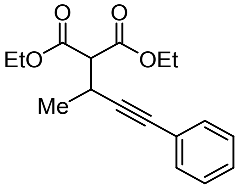

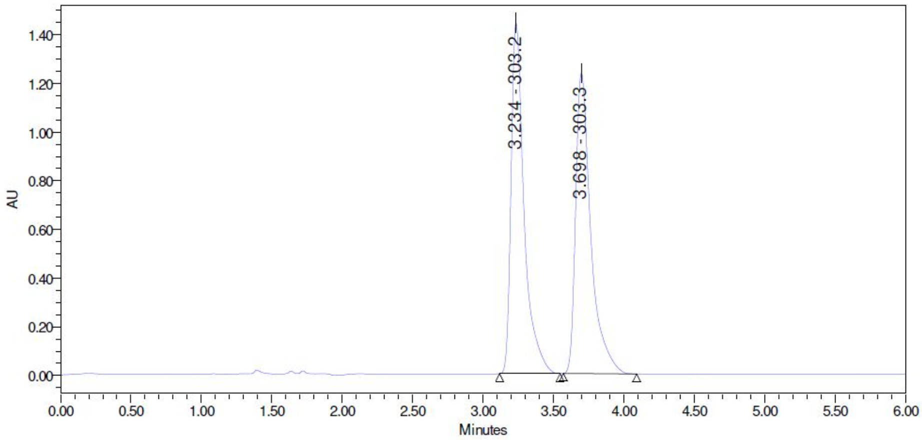

	Retention Time (min)	Area (µV*sec)	% Area	Width (sec)
1	3.23	9233864	50.0	25.800
2	3.70	9234757	50.0	31.200

(*R*)-Diethyl 2-(5-phenylpent-4-yn-2-yl)malonate (4g)

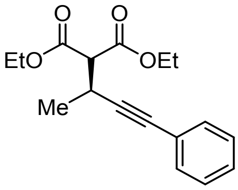

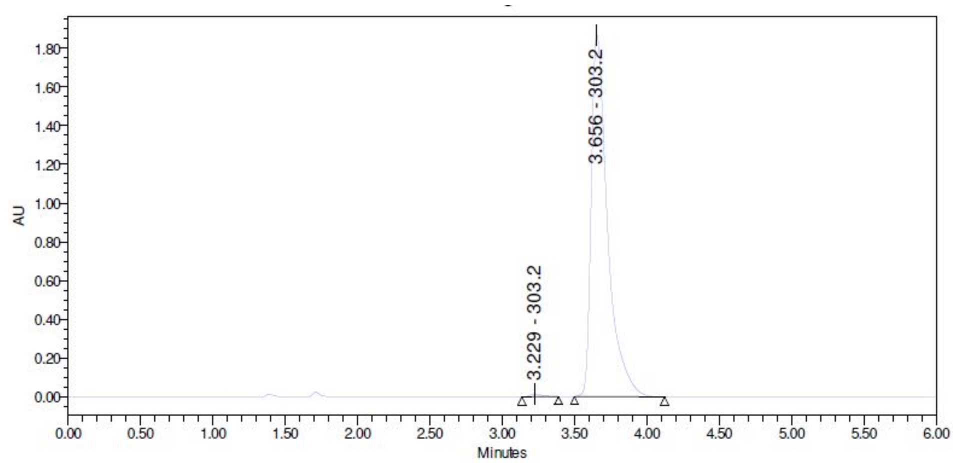

	Retention Time (min)	Area (μV*sec)	% Area	Width (sec)
1	3.23	80553	0.5	15.200
2	3.66	14644051	99.5	37.400

Diethyl 2-(1-(furan-2-yl)propan-2-yl)malonate (S34)

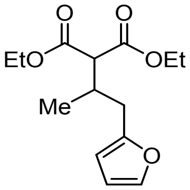

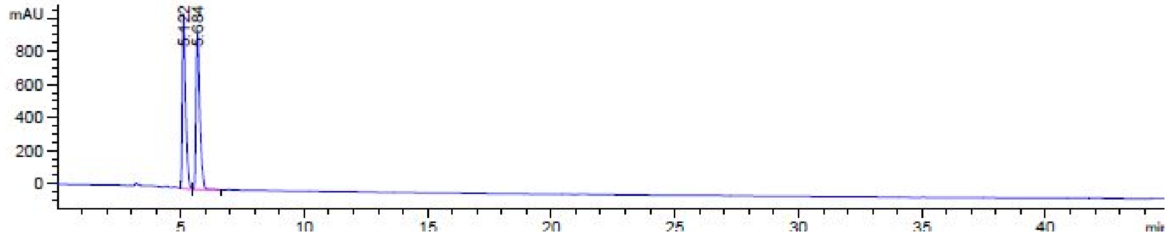

```
Signal 2: DAD1 B, Sig=210,4 Ref=360,100

Peak RetTime Type  Width     Area      Height     Area
  #   [min]        [min]   [mAU*s]     [mAU]        %
----|-------|----|-------|----------|----------|--------|
   1   5.122 VB    0.1351 9694.44922 1056.12061  49.7255
   2   5.684 BB    0.1529 9801.48535  945.68536  50.2745

Totals :                  1.94959e4  2001.80597
```

(*S*)-Diethyl 2-(1-(furan-2-yl)propan-2-yl)malonate (4h)

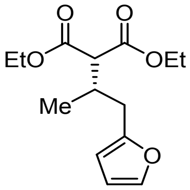

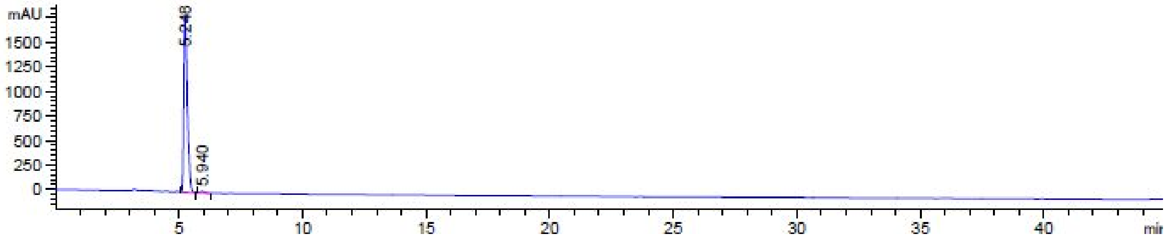

Signal 2: DAD1 B, Sig=210,4 Ref=360,100

Peak #	RetTime [min]	Type	Width [min]	Area [mAU*s]	Height [mAU]	Area %
1	5.248	VB	0.1532	1.85975e4	1819.59216	99.0254
2	5.940	BB	0.1535	183.03903	17.58120	0.9746
Totals :				1.87806e4	1837.17336	

3-benzyl 1,1-diethyl butane-1,1,3-tricarboxylate (S32)

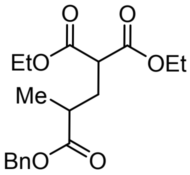

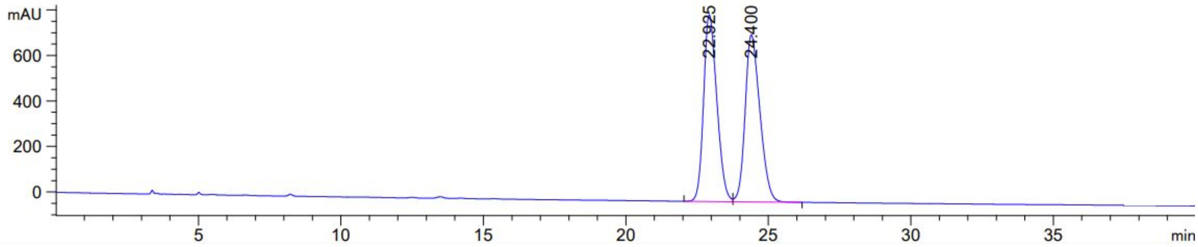

Signal 2: DAD1 B, Sig=210,4 Ref=360,100

Peak #	RetTime [min]	Type	Width [min]	Area [mAU*s]	Height [mAU]	Area %
1	22.925	BV	0.4961	2.64695e4	821.18262	49.9659
2	24.400	VB	0.5533	2.65056e4	733.94116	50.0341
Totals :				5.29751e4	1555.12378	

3-benzyl 1,1-diethyl (*R*)-butane-1,1,3-tricarboxylate (4q)

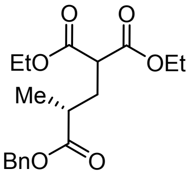

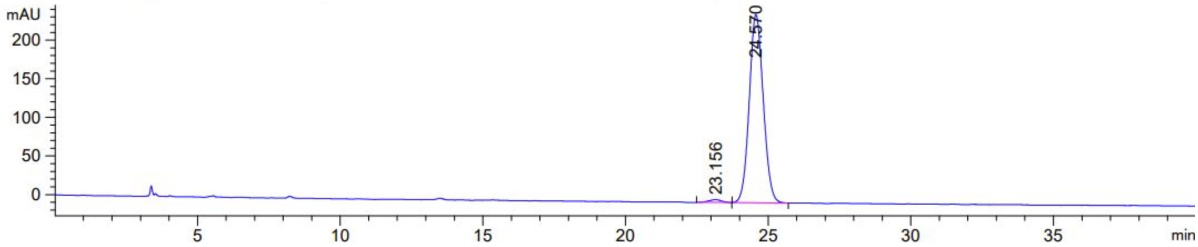

Signal 2: DAD1 B, Sig=210,4 Ref=360,100

Peak #	RetTime [min]	Type	Width [min]	Area [mAU*s]	Height [mAU]	Area %
1	23.156	BB	0.4508	107.93516	3.51002	1.2823
2	24.570	BB	0.5279	8309.17383	243.63776	98.7177
Totals :				8417.10899	247.14777	

2-(4-phenylbutan-2-yl)malononitrile (S35)

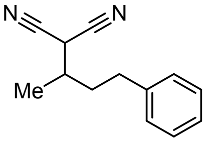

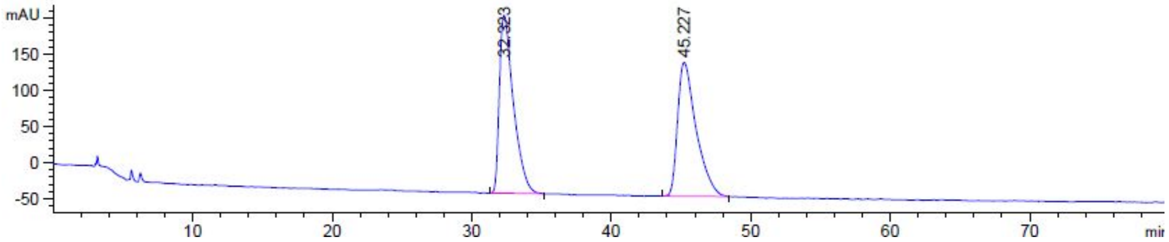

Signal 2: DAD1 B, Sig=210,4 Ref=360,100

Peak #	RetTime [min]	Type	Width [min]	Area [mAU*s]	Height [mAU]	Area %
1	32.323	BB	1.0448	1.71754e4	245.33470	50.1186
2	45.227	BB	1.3559	1.70942e4	184.66794	49.8814
Totals :				3.42696e4	430.00264	

(*S*)-2-(4-phenylbutan-2-yl)malononitrile (4l)

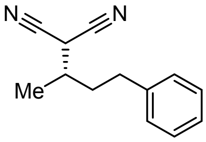

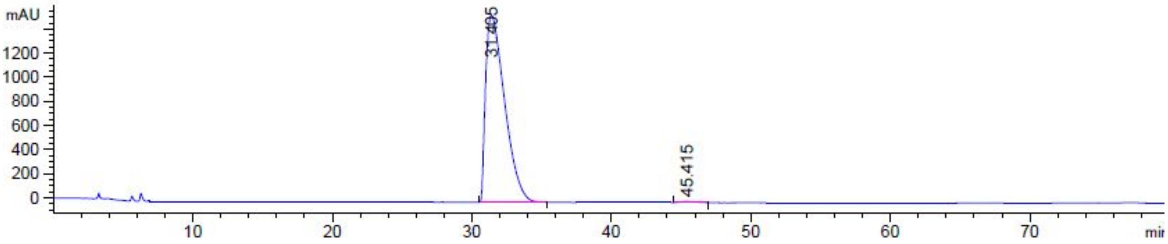

Signal 2: DAD1 B, Sig=210,4 Ref=360,100

Peak #	RetTime [min]	Type	Width [min]	Area [mAU*s]	Height [mAU]	Area %
1	31.405	BB	1.3760	1.46996e5	1550.61853	99.6171
2	45.415	BB	0.9050	565.00330	7.62063	0.3829
Totals :				1.47561e5	1558.23916	

(2-methyl-4-phenylbutane-1,1-diyldisulfonyl)dibenzene (S36)

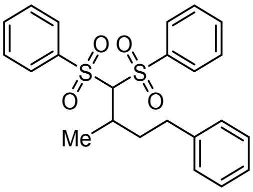

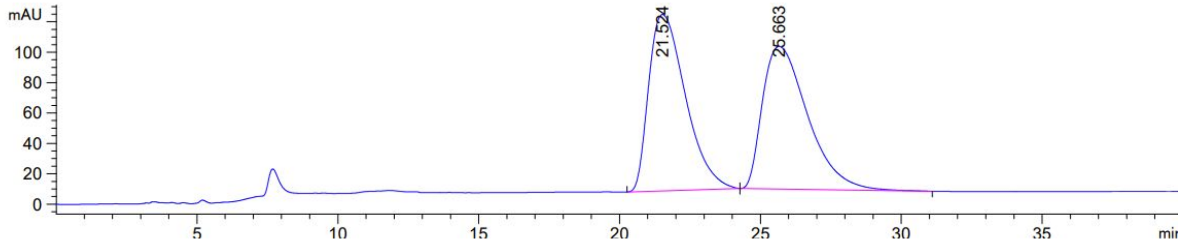

Signal 1: DAD1 A, Sig=254,4 Ref=360,100

Peak #	RetTime [min]	Type	Width [min]	Area [mAU*s]	Height [mAU]	Area %
1	21.524	BB	1.4187	1.05340e4	116.07716	50.0856
2	25.663	BB	1.6936	1.04980e4	93.95023	49.9144
Totals :				2.10320e4	210.02739	

(*S*)-(2-methyl-4-phenylbutane-1,1-diyldisulfonyl)dibenzene (4k)

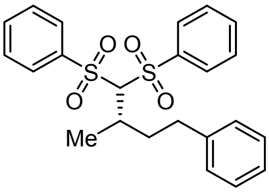

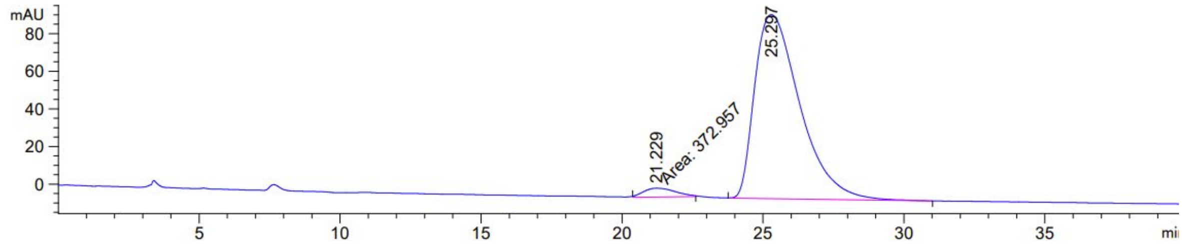

Signal 1: DAD1 A, Sig=254,4 Ref=360,100

Peak #	RetTime [min]	Type	Width [min]	Area [mAU*s]	Height [mAU]	Area %
1	21.229	MM	1.3162	372.95724	4.72270	3.3020
2	25.297	BB	1.6745	1.09219e4	97.82813	96.6980
Totals :				1.12948e4	102.55082	

(*R*)-Dimethyl-2-(1-phenylpropan-2-yl)malonate (4j)

See Absolute Stereochemistry Standards section for the chromatogram of the related racemic sample (**S13**).

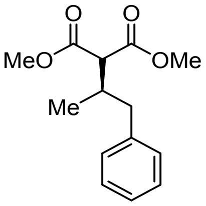

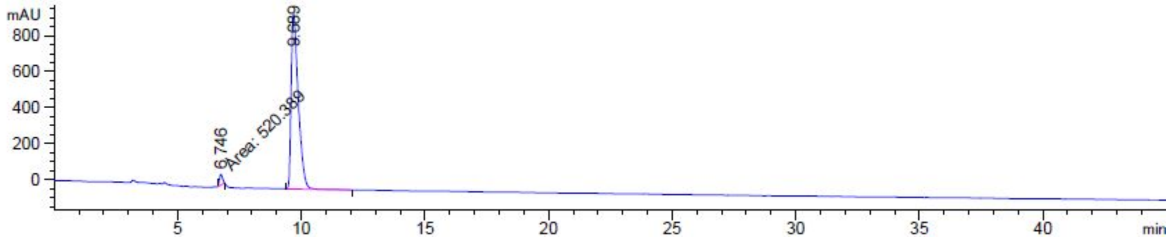

Signal 2: DAD1 B, Sig=210,4 Ref=360,100

Peak #	RetTime [min]	Type	Width [min]	Area [mAU*s]	Height [mAU]	Area %
1	6.746	MM	0.1439	520.38885	60.27648	2.5992
2	9.689	BB	0.3005	1.95006e4	970.05670	97.4008
Totals :				2.00210e4	1030.33318	

Absolute Stereochemistry Standards

Dimethyl-2-(1-phenylpropan-2-yl)malonate (S13)

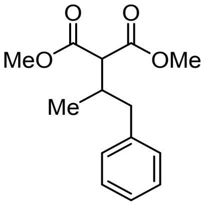

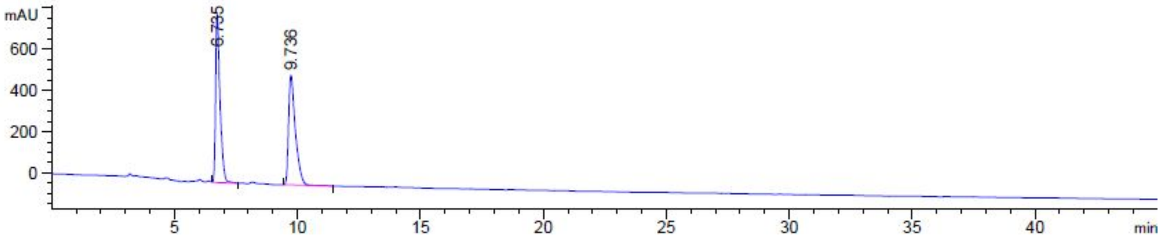

Signal 2: DAD1 B, Sig=210,4 Ref=360,100

Peak #	RetTime [min]	Type	Width [min]	Area [mAU*s]	Height [mAU]	Area %
1	6.735	VB	0.1821	1.01896e4	813.49945	49.9074
2	9.736	BB	0.2850	1.02274e4	530.39813	50.0926
Totals :				2.04170e4	1343.89758	

(*R*)-Dimethyl-2-(1-phenylpropan-2-yl)malonate (S14)

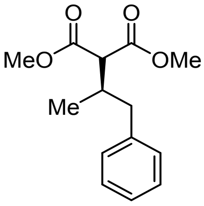

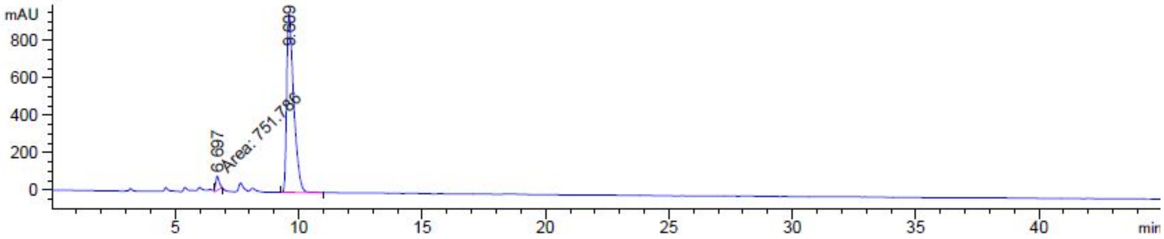

Signal 2: DAD1 B, Sig=210,4 Ref=360,100

Peak #	RetTime [min]	Type	Width [min]	Area [mAU*s]	Height [mAU]	Area %
1	6.697	MM	0.1669	751.78650	75.09280	3.7952
2	9.609	BB	0.2971	1.90569e4	953.84467	96.2048

Totals : 1.98087e4 1028.93746

(*S*)-Dimethyl-2-(1-phenylpropan-2-yl)malonate (S12)

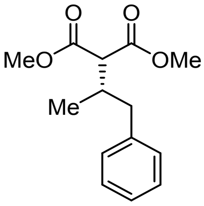

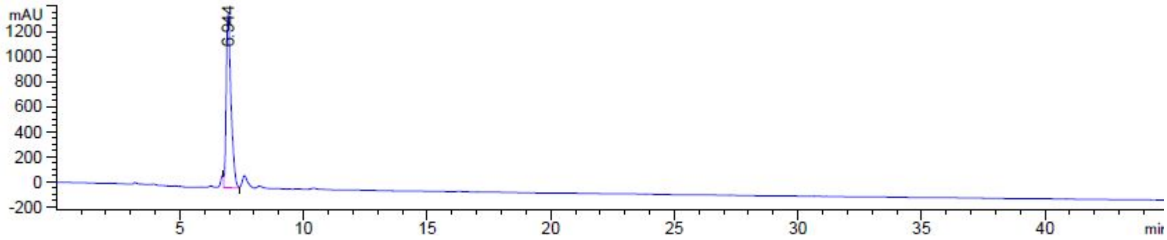

Signal 2: DAD1 B, Sig=210,4 Ref=360,100

Peak #	RetTime [min]	Type	Width [min]	Area [mAU*s]	Height [mAU]	Area %
1	6.944	VV	0.1988	1.85969e4	1383.70471	100.0000

Totals : 1.85969e4 1383.70471

NMR spectra

Alcohol Starting Materials

(*S*)-1-(benzyloxy)propan-2-ol (A1)

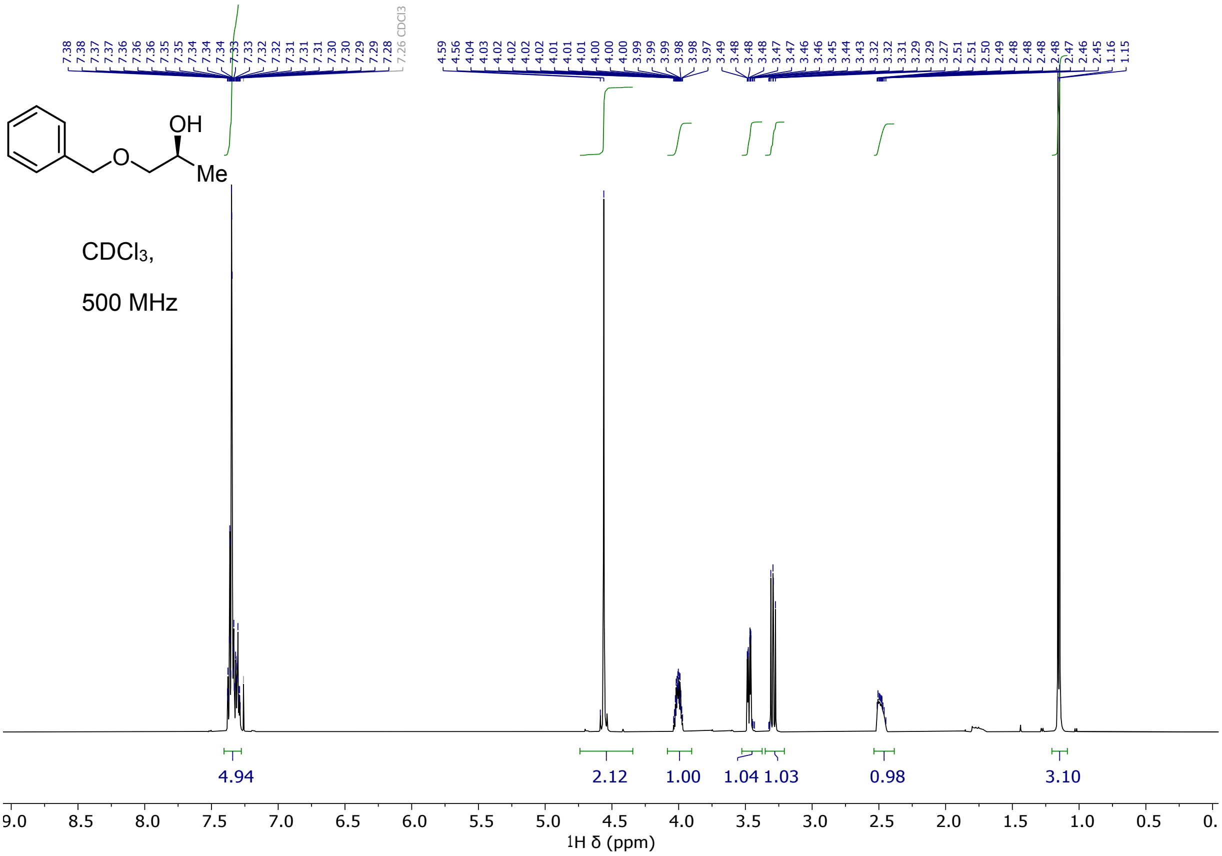

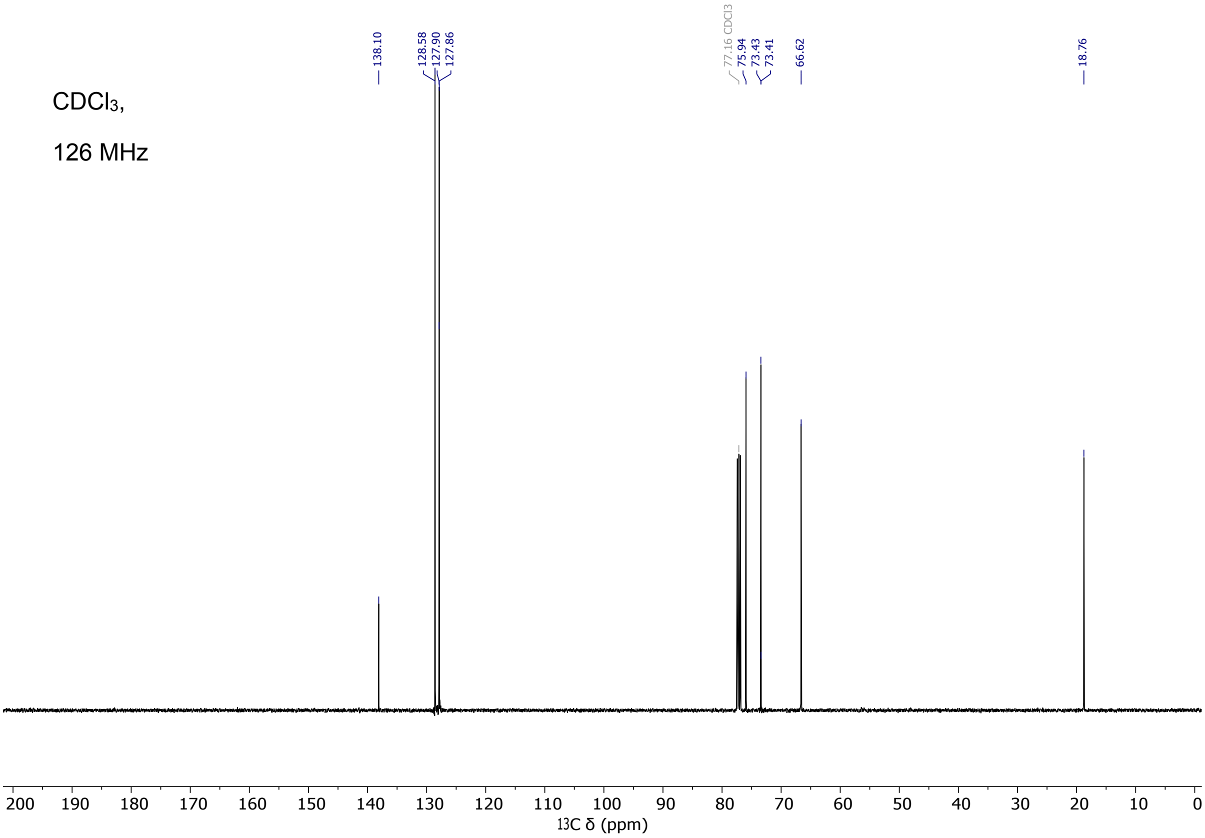

(*R*)-(−)-4-(phenylsulfonyl)-2-butanol (S6)

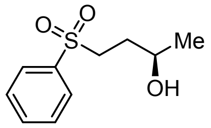

$CDCl_3$,

400 MHz

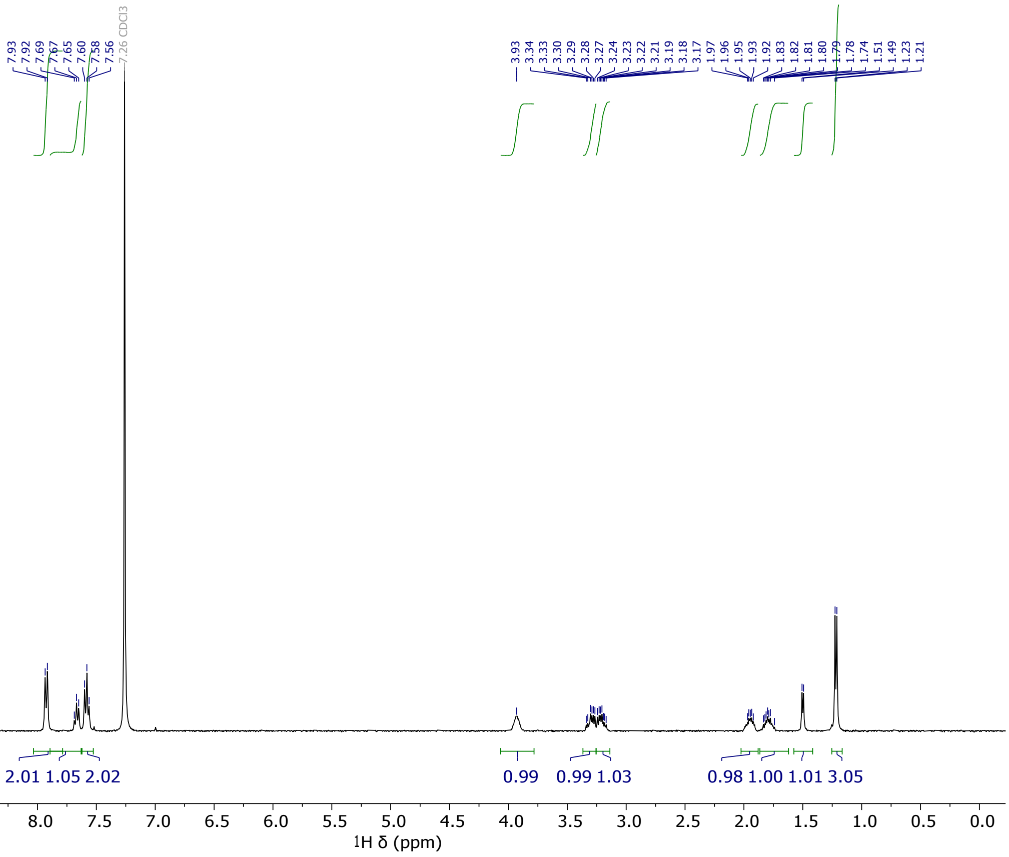

$CDCl_3$,

126 MHz

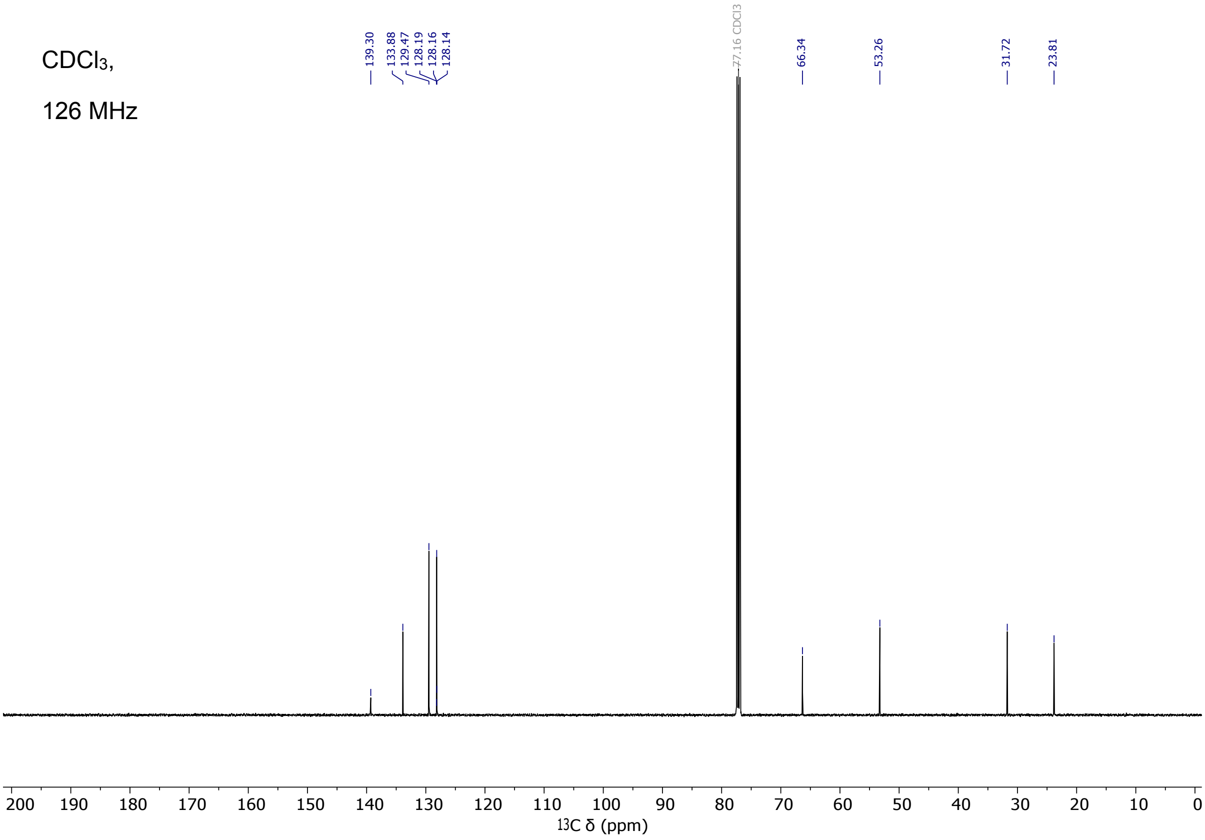

(*R*)-1-((tert-butyldiphenylsilyl)oxy)propan-2-ol (S7)

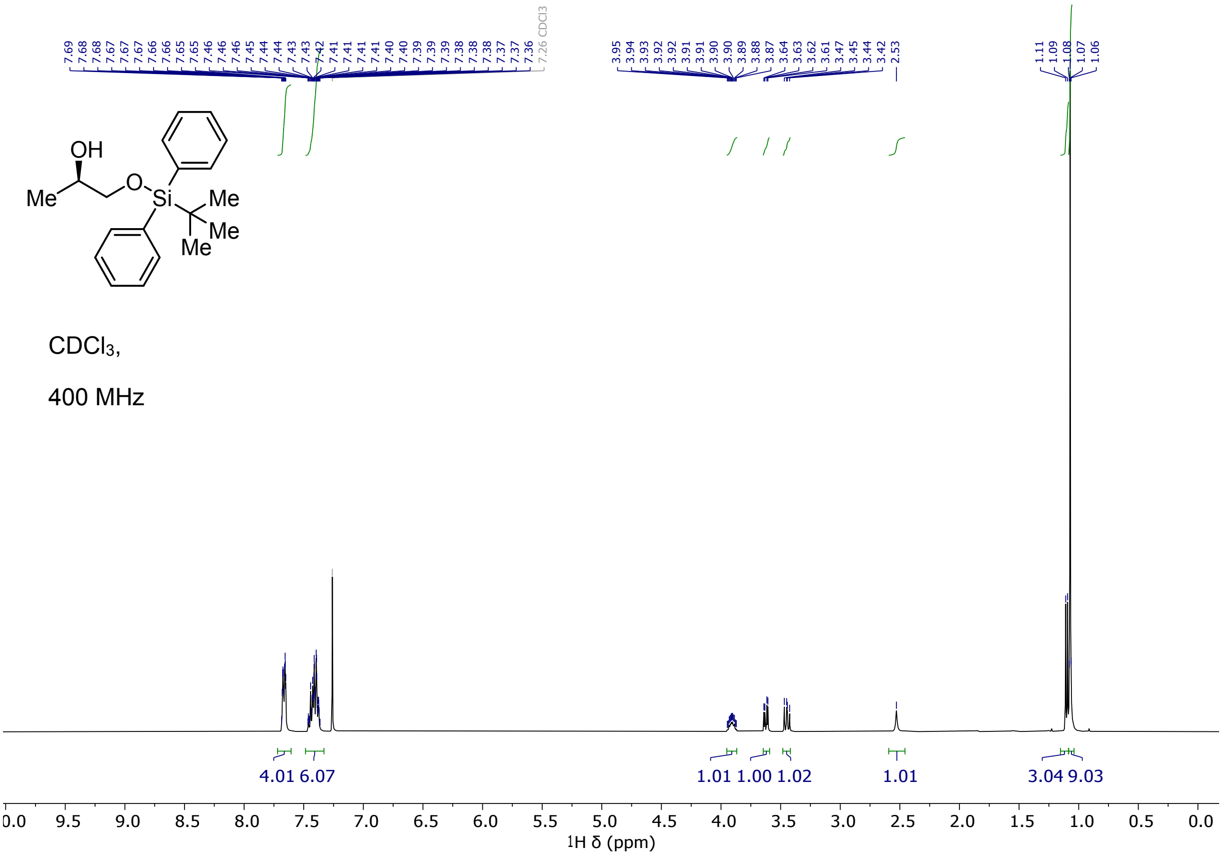

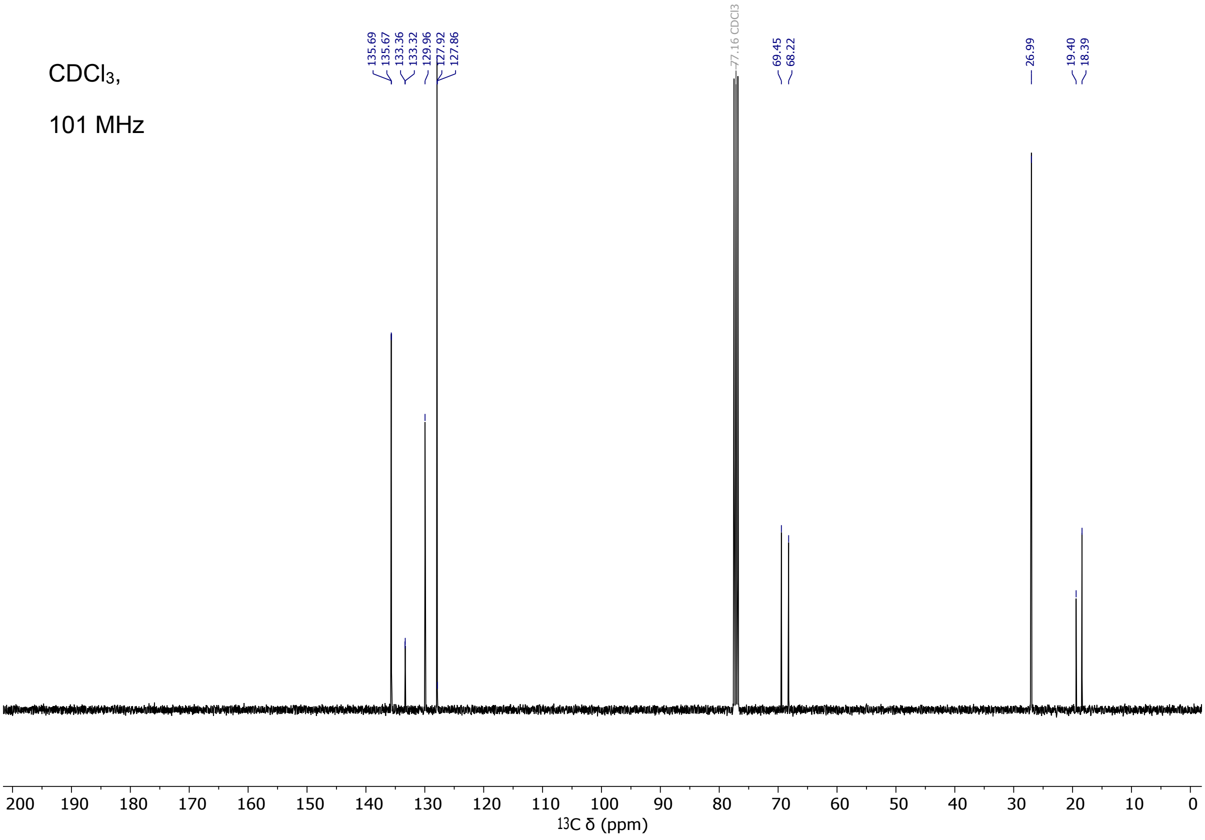

(*S*)-2-(2-hydroxypropyl)furan (S10)

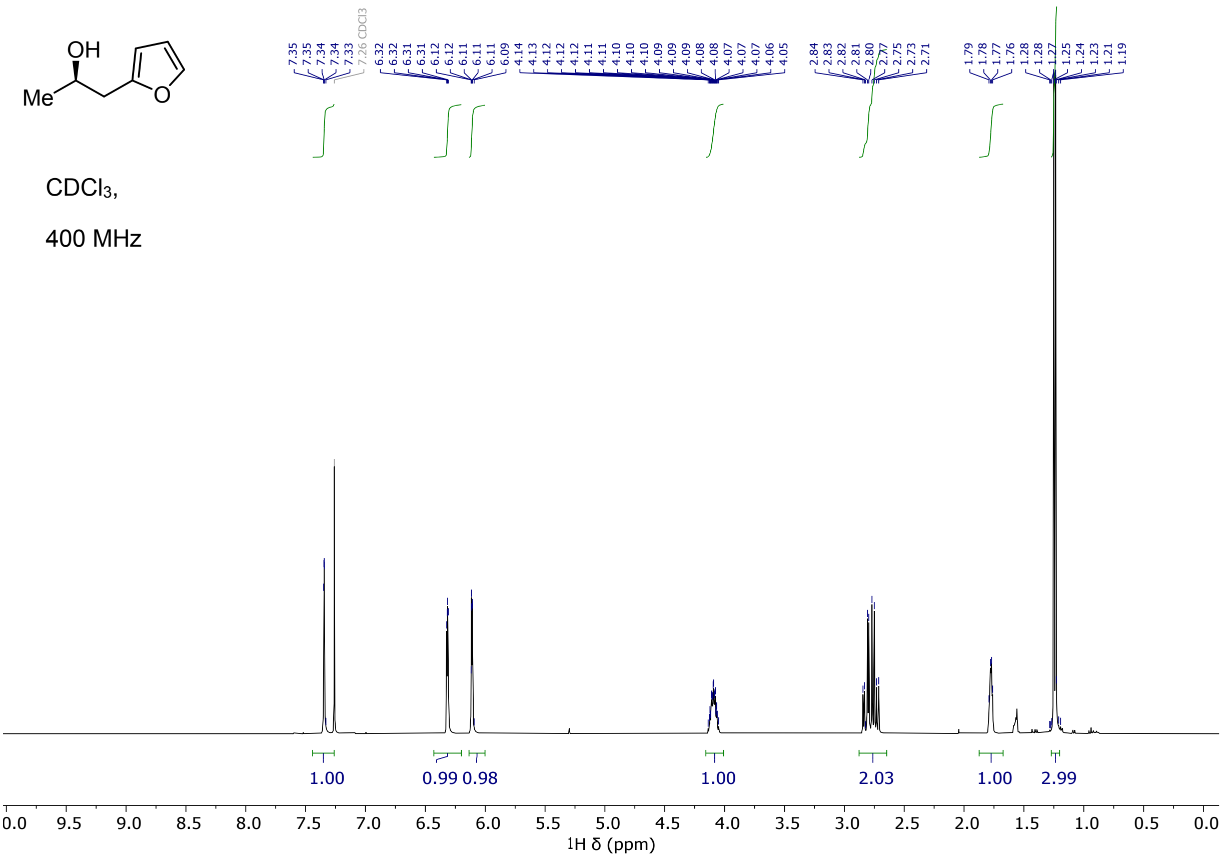

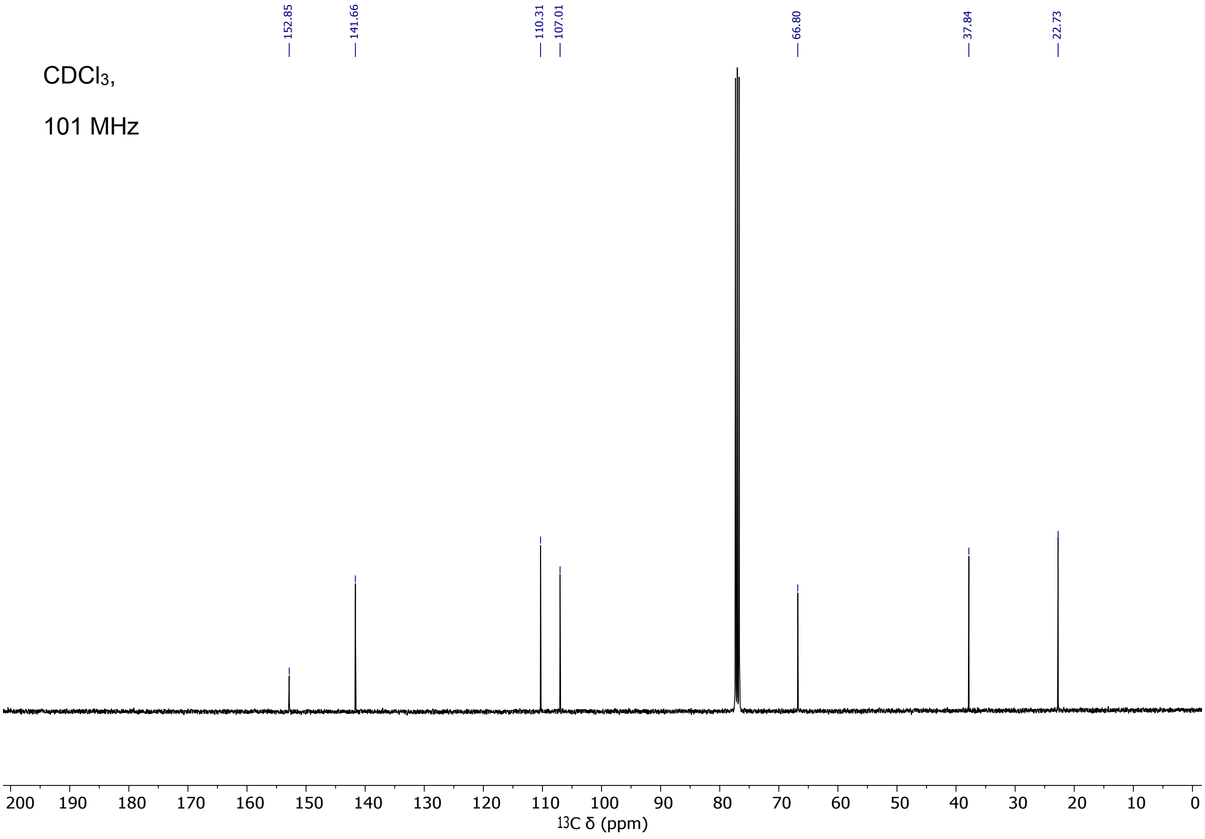

(*S*)-5-Phenylpent-4-yn-2-ol (S11)

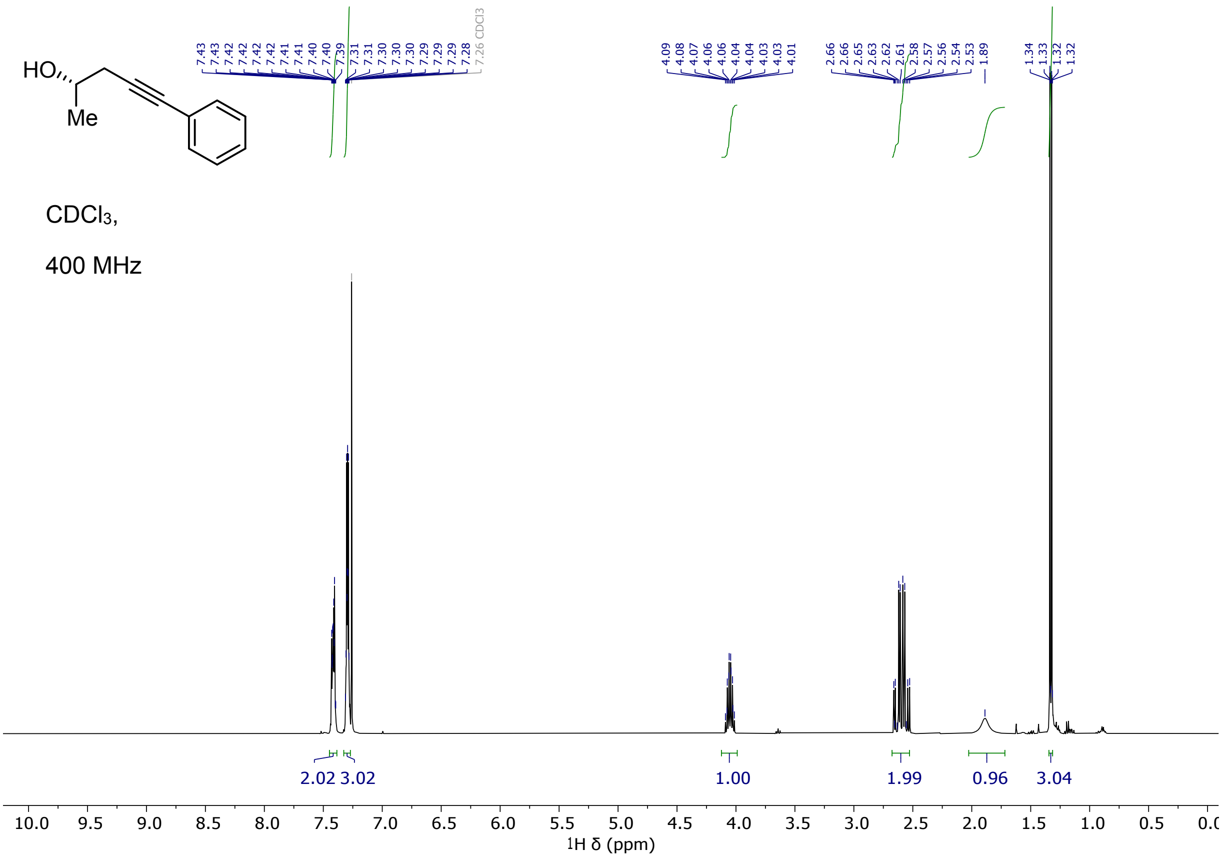

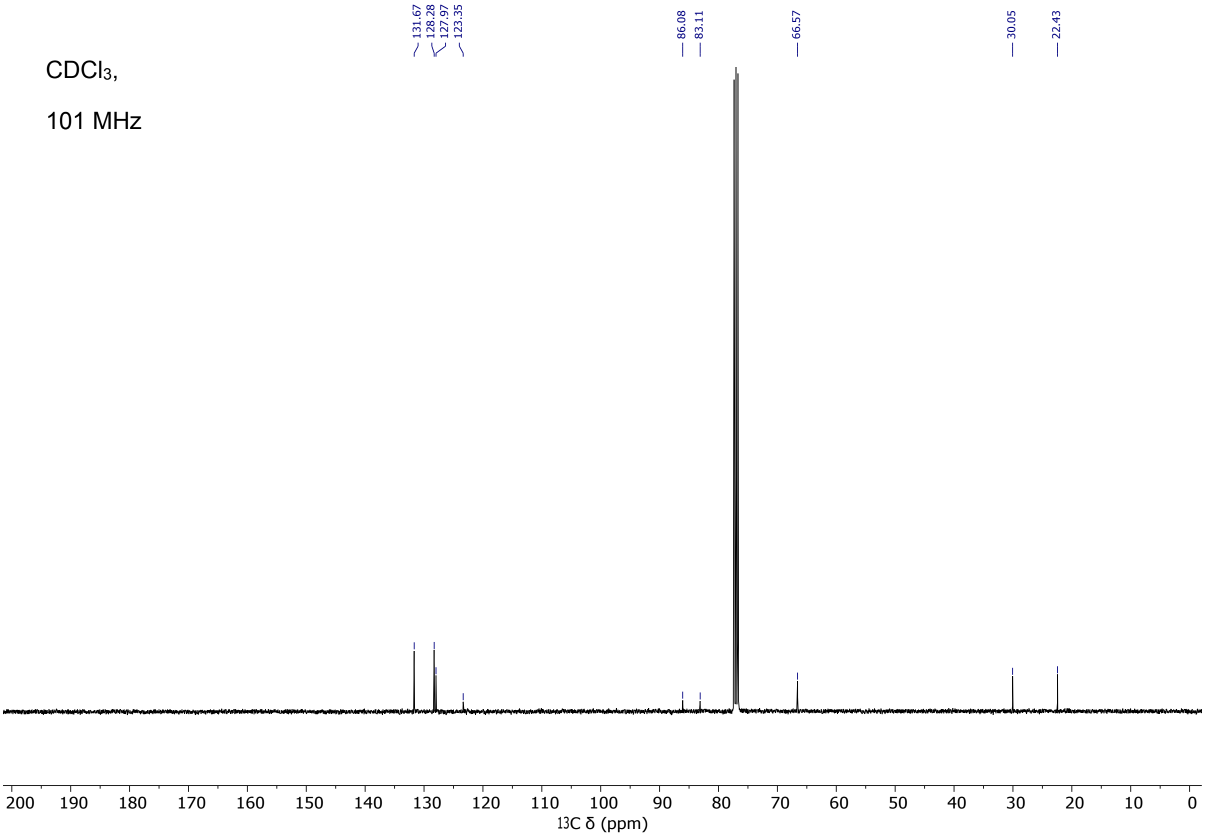

(*R*)-Benzyl 3-hydroxy-2-methylpropanoate (S8)

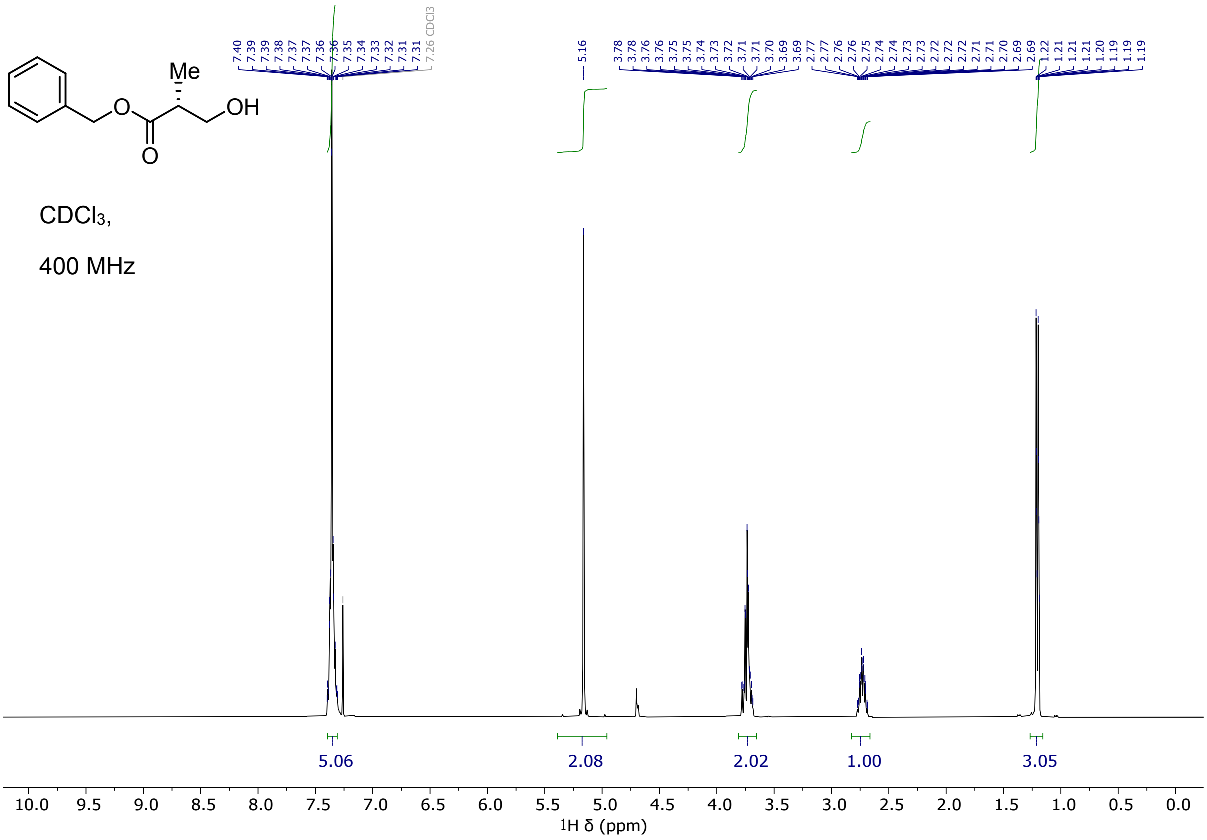

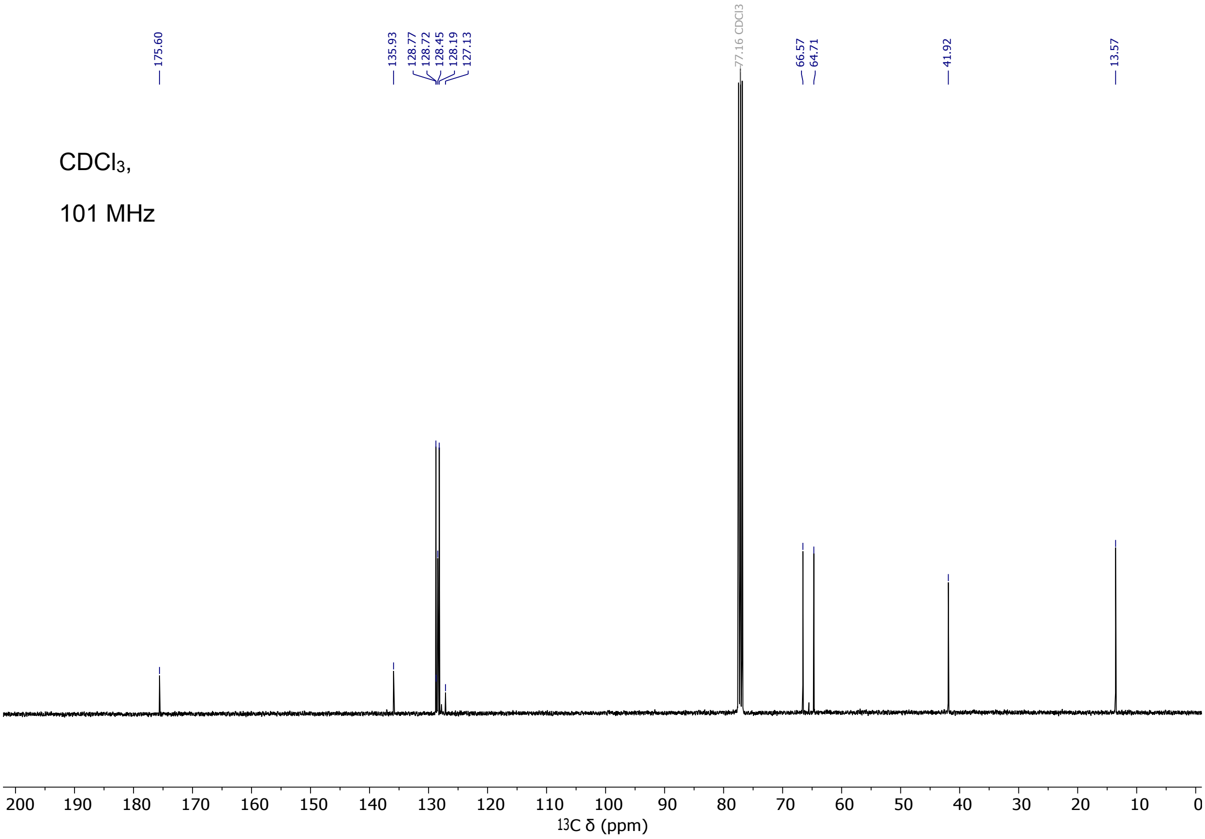

Products (from secondary alcohols)

(*R*)-Diethyl 2-(1-(benzyloxy)propan-2-yl)malonate (4a)

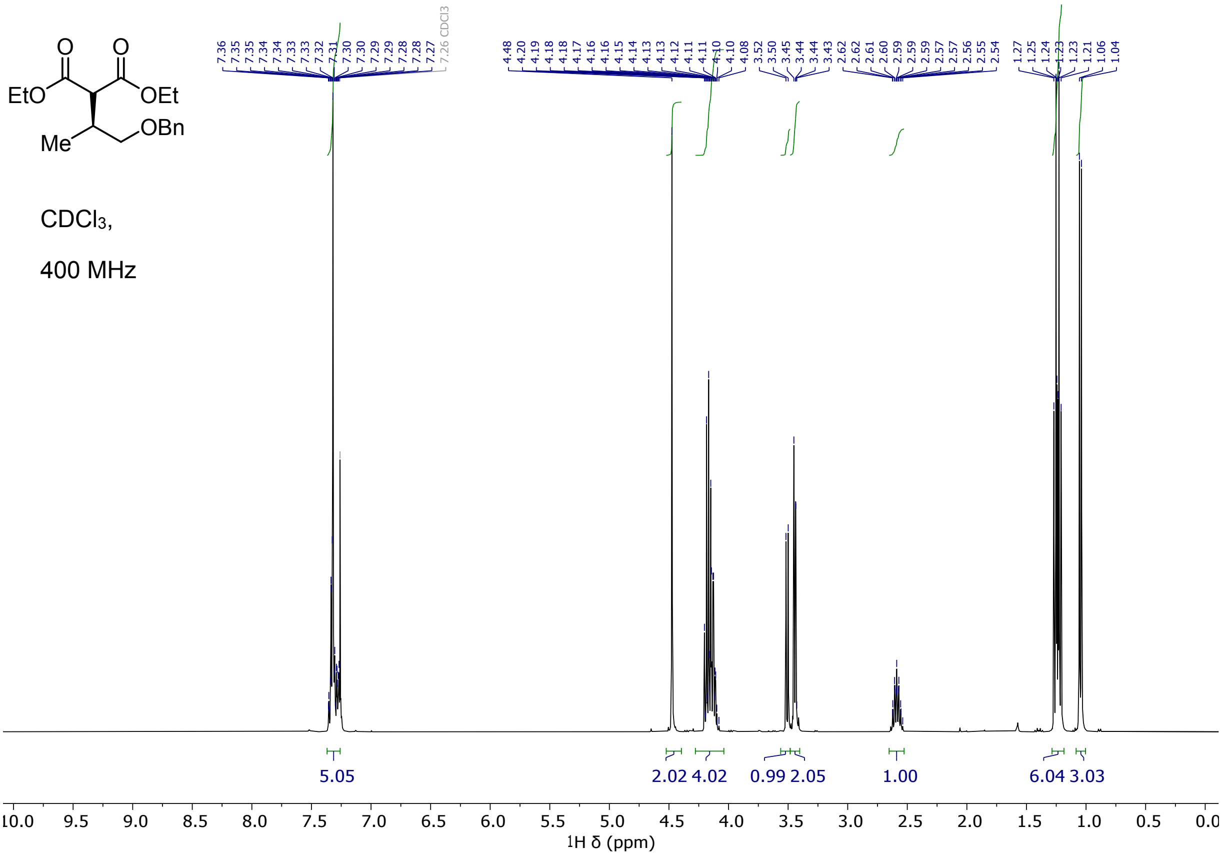

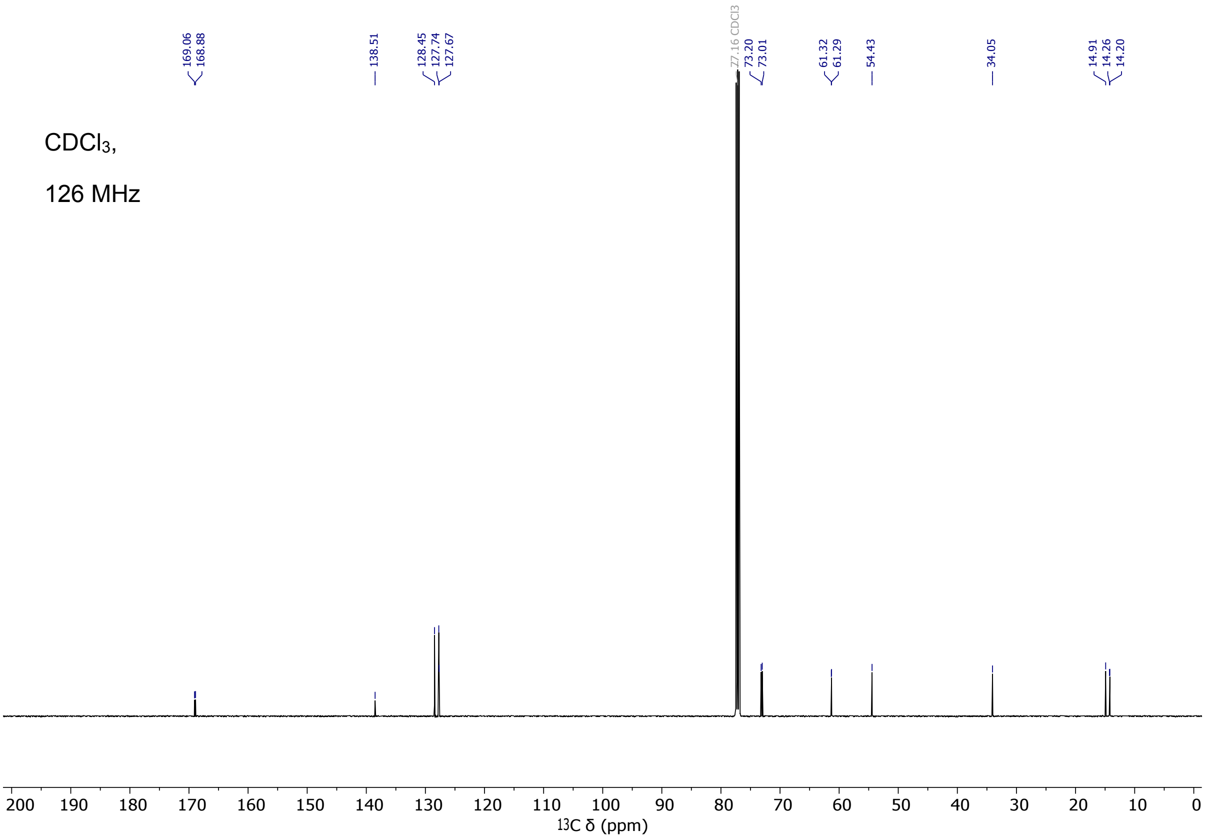

(*S*)-Diethyl 2-(4-phenylbutan-2-yl)malonate (4b)

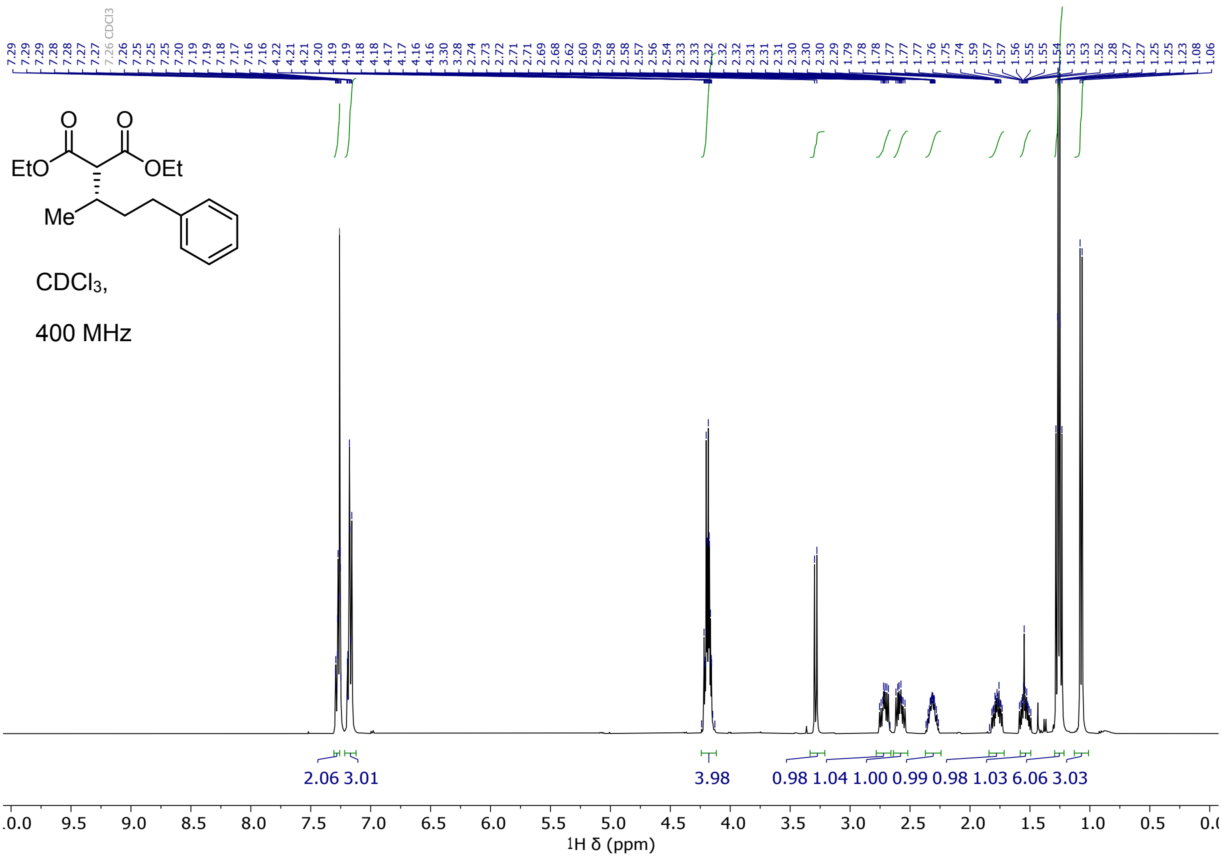

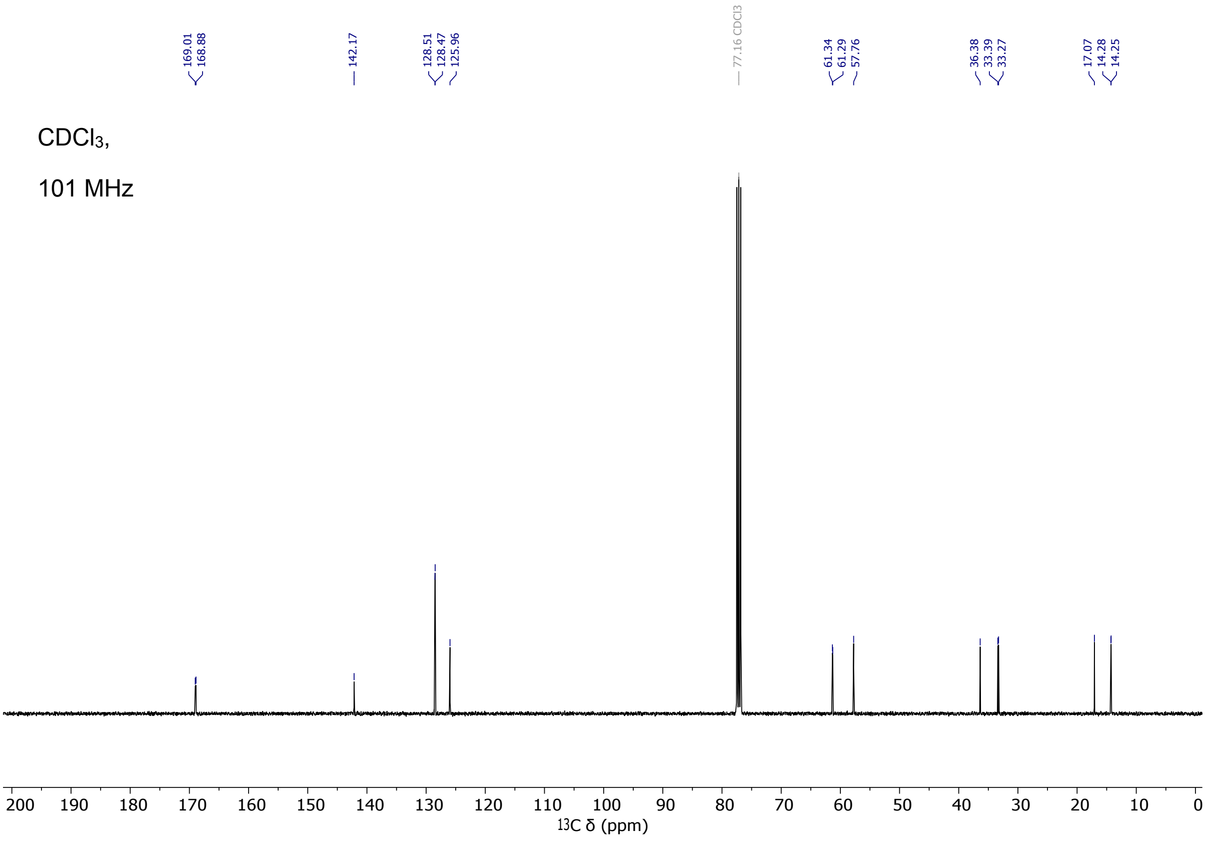

(*S*)-Diethyl 2-(1-phenylpropan-2-yl)malonate (4c)

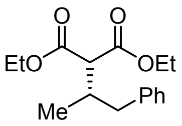

CDCl$_3$,

500 MHz

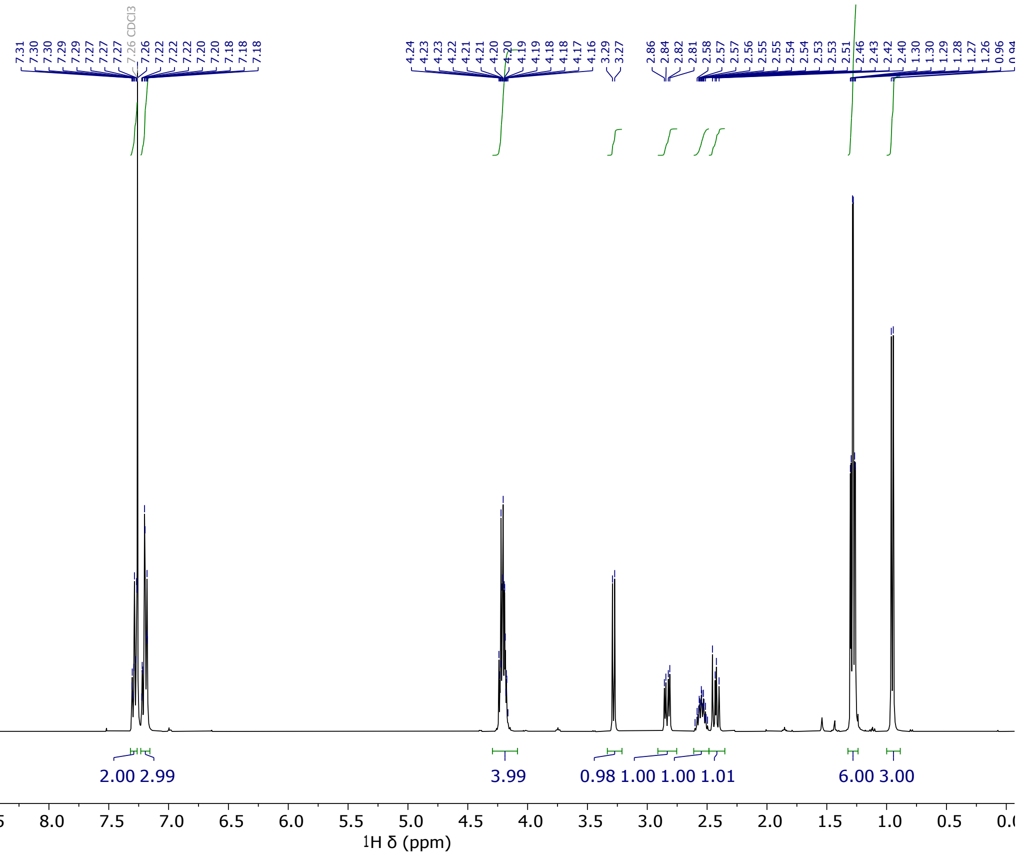

CDCl$_3$,

126 MHz

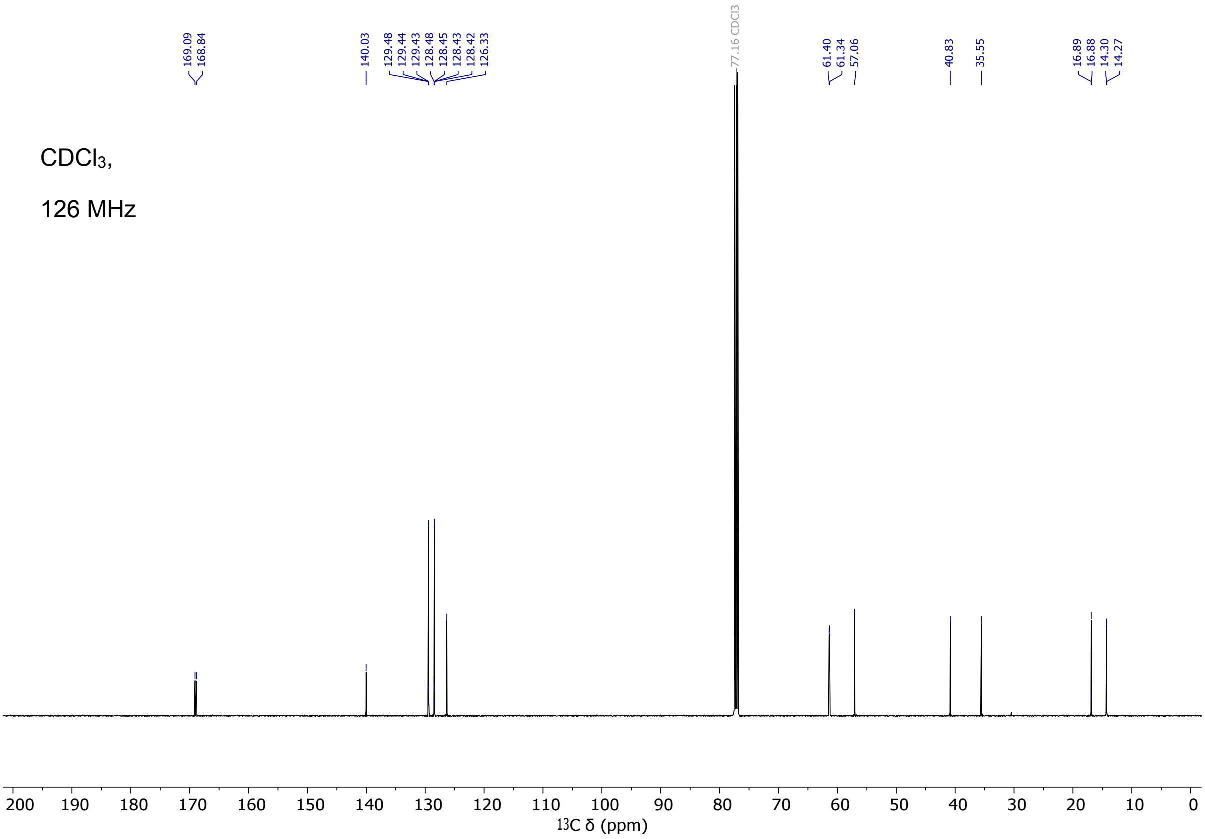

(*R*)-Dibenzyl 2-(octan-2-yl)malonate (4e)

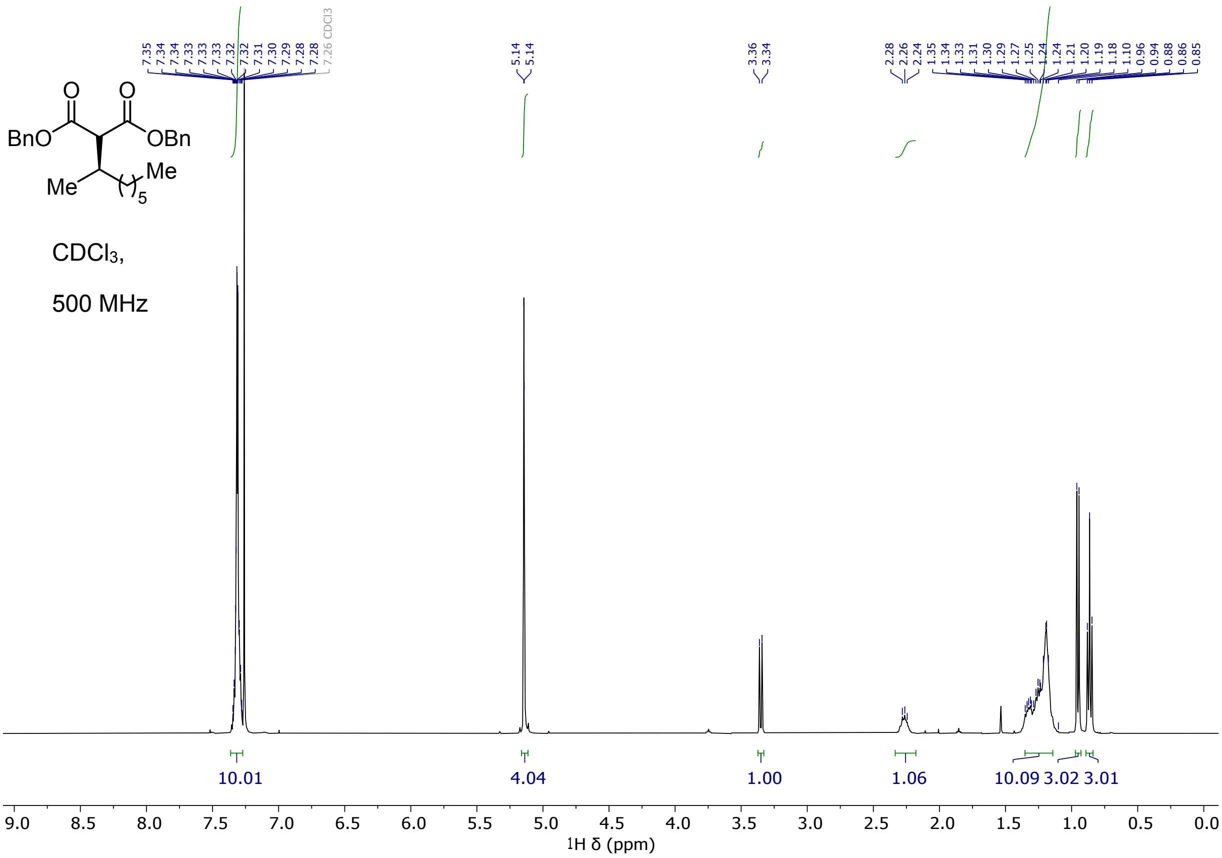

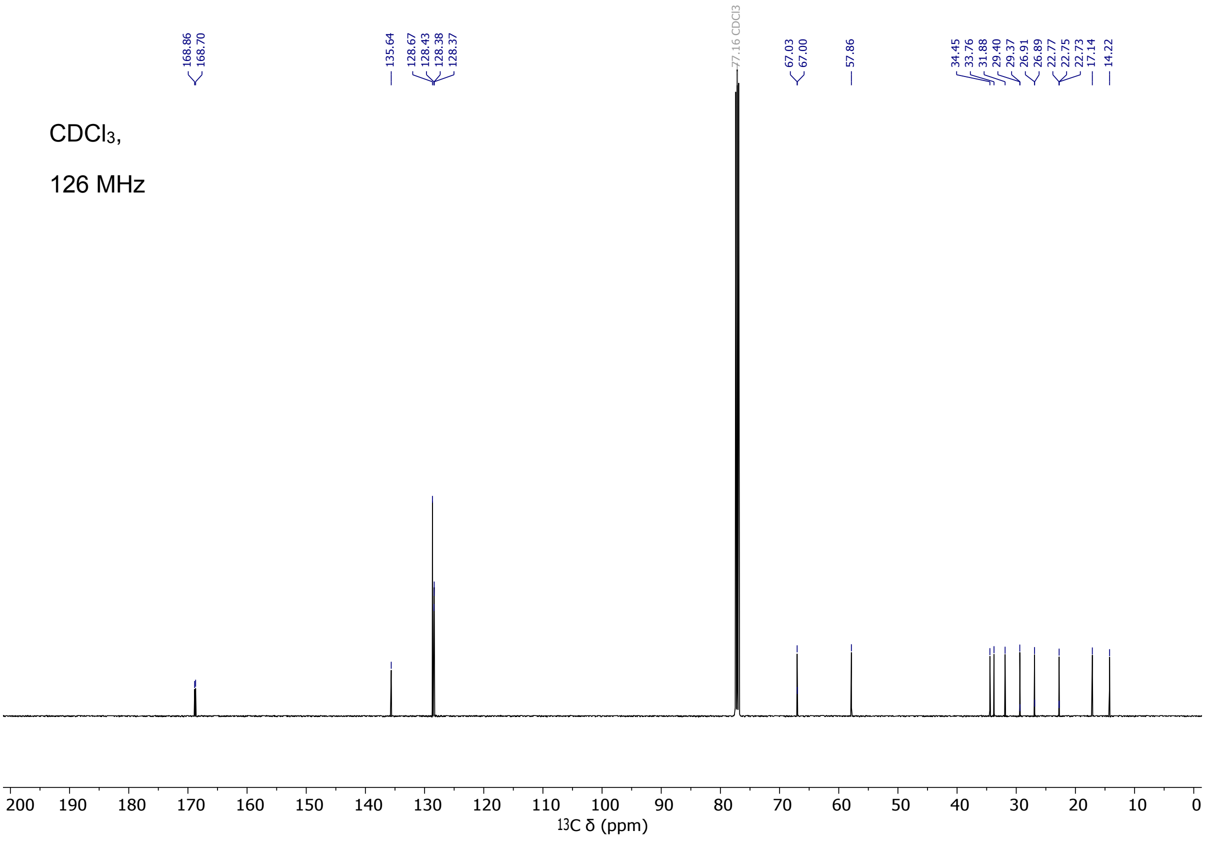

(*R*)-Diethyl 2-(1-((tert-butyldiphenylsilyl)oxy)propan-2-yl)malonate (4i)

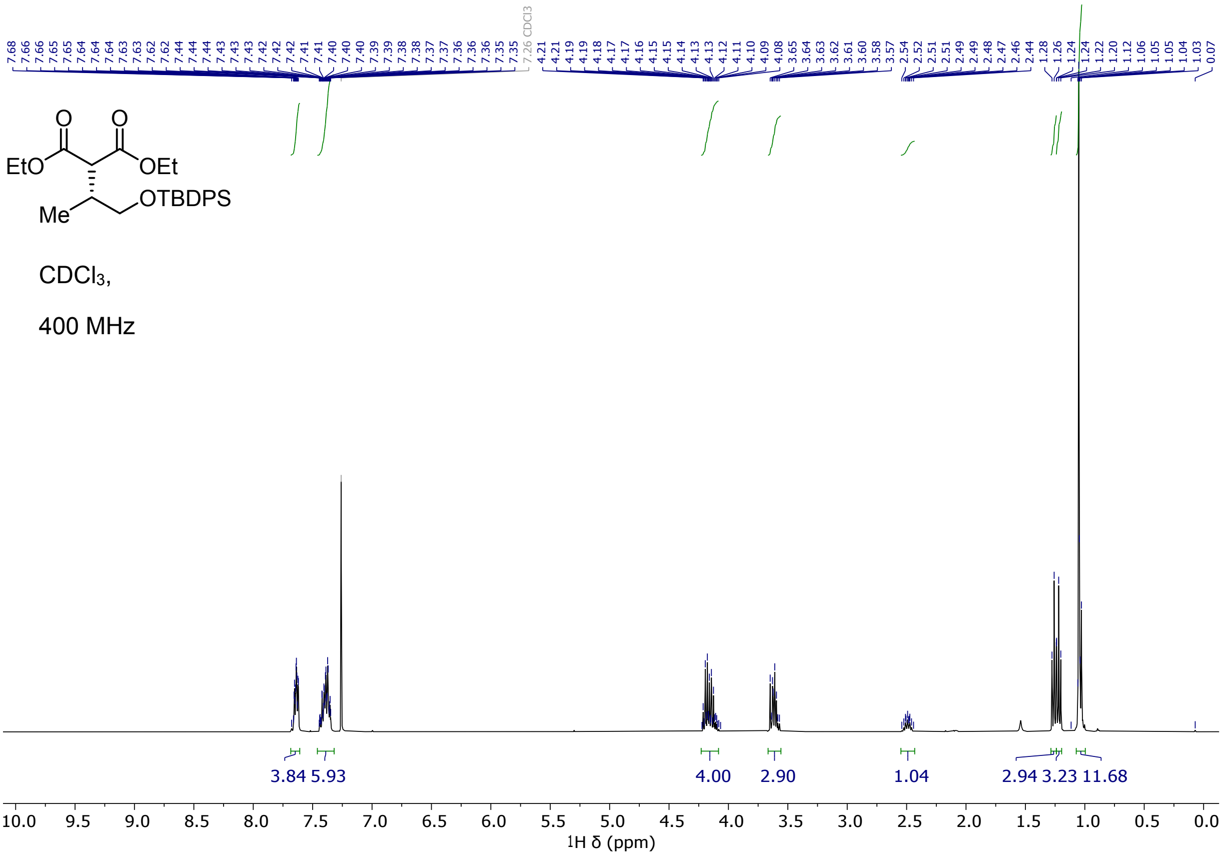

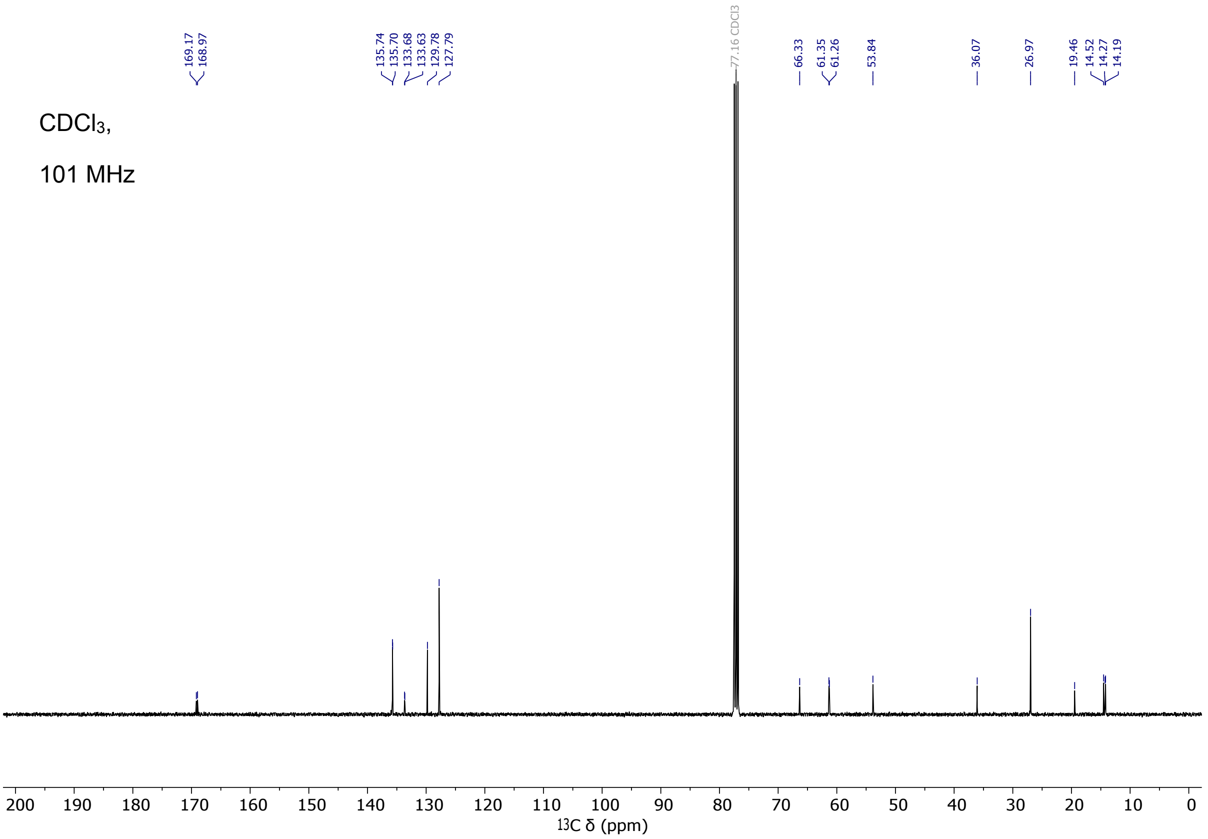

(*S*)-Diethyl 2-(4-(phenylsulfonyl)butan-2-yl)malonate (4d)

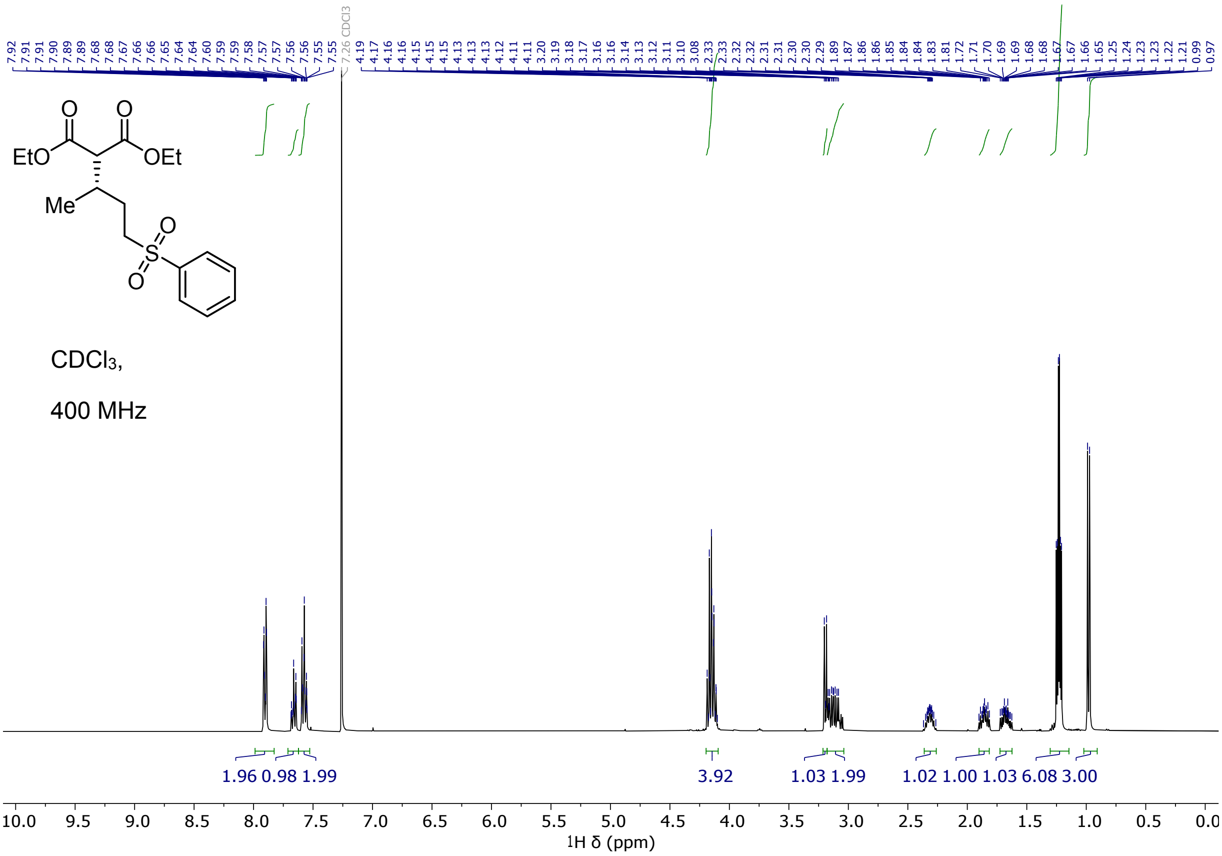

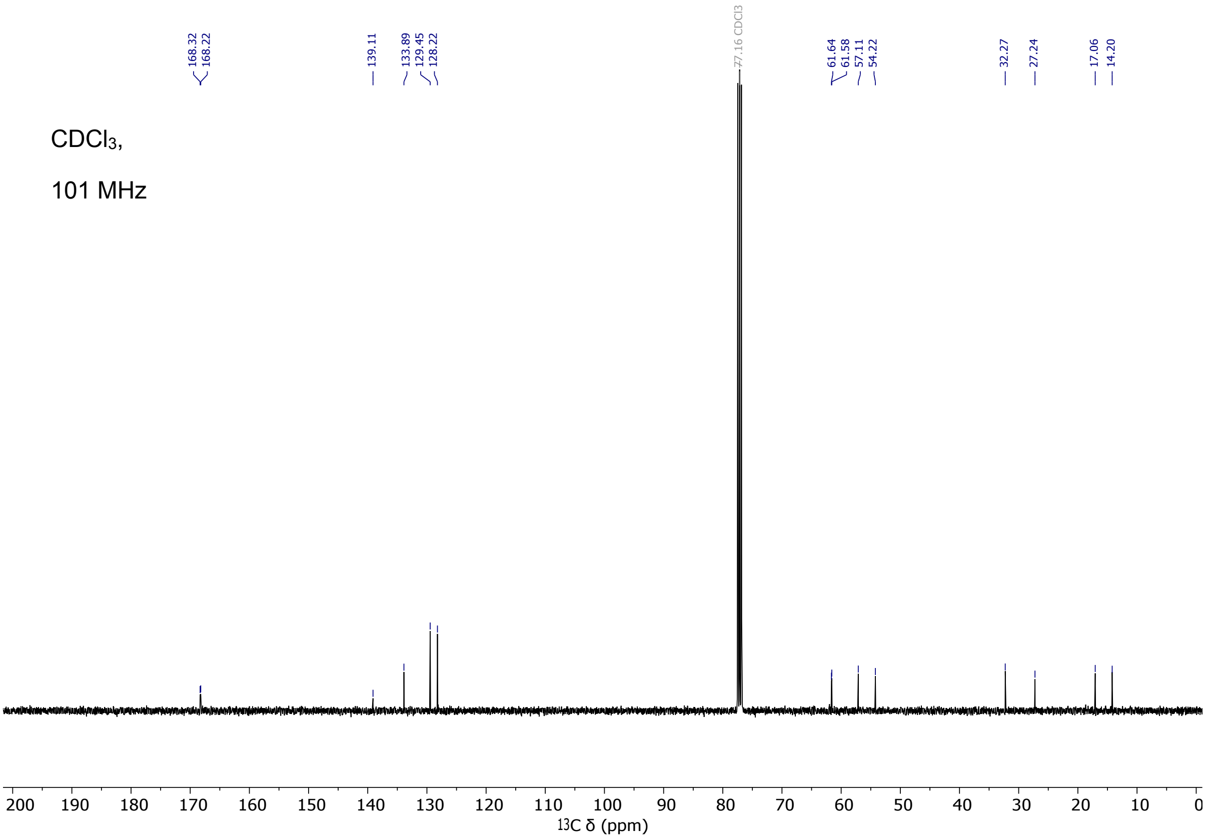

(*R*)-Dimethyl-2-(1-phenylpropan-2-yl)malonate (4j)

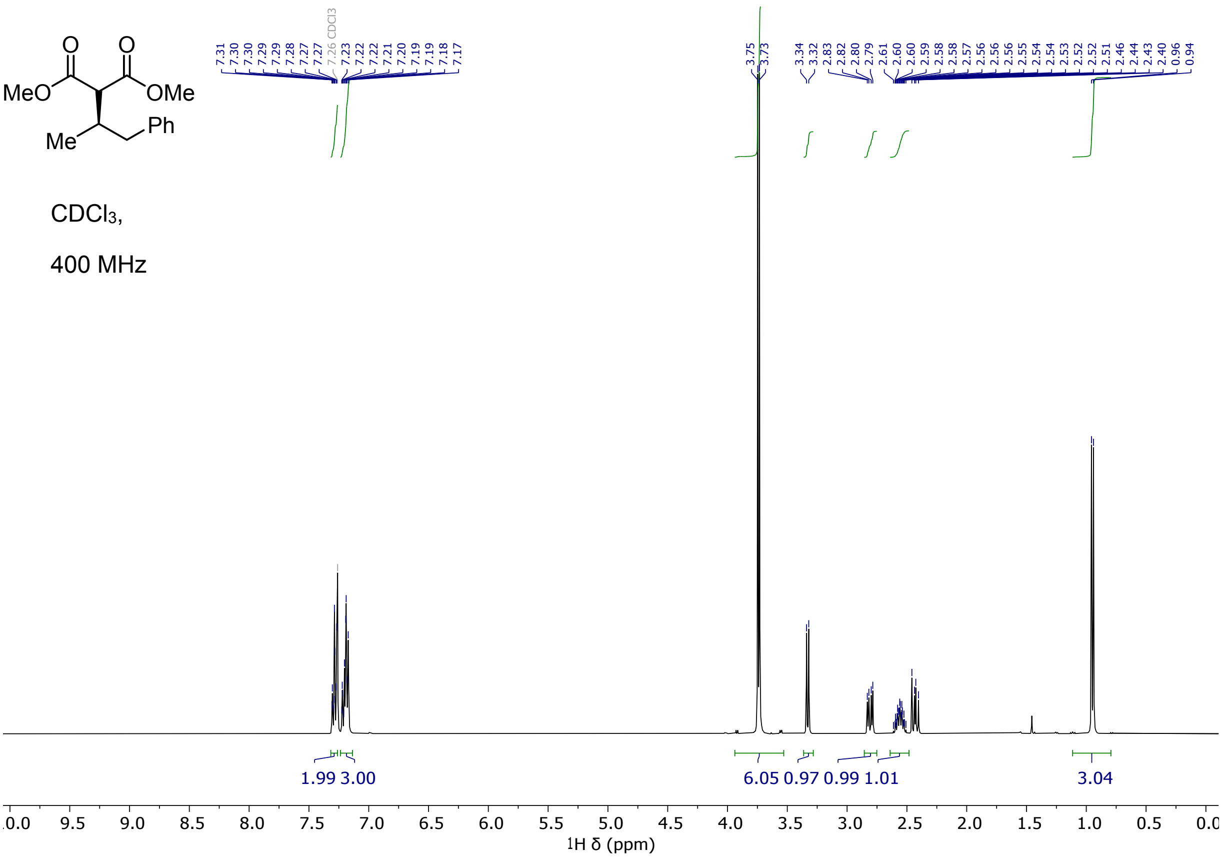

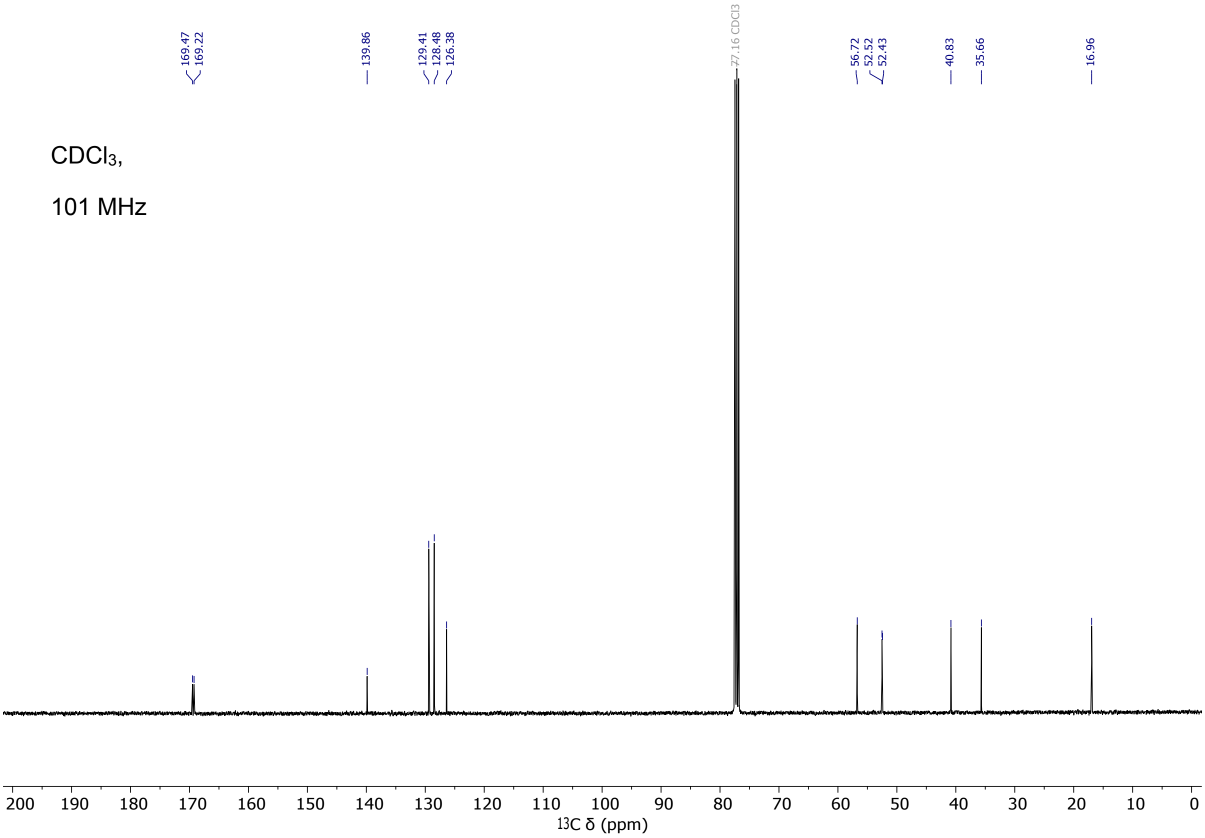

Recovered triphenylphosphine oxide (1) from the synthesis of 4j

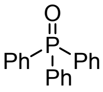

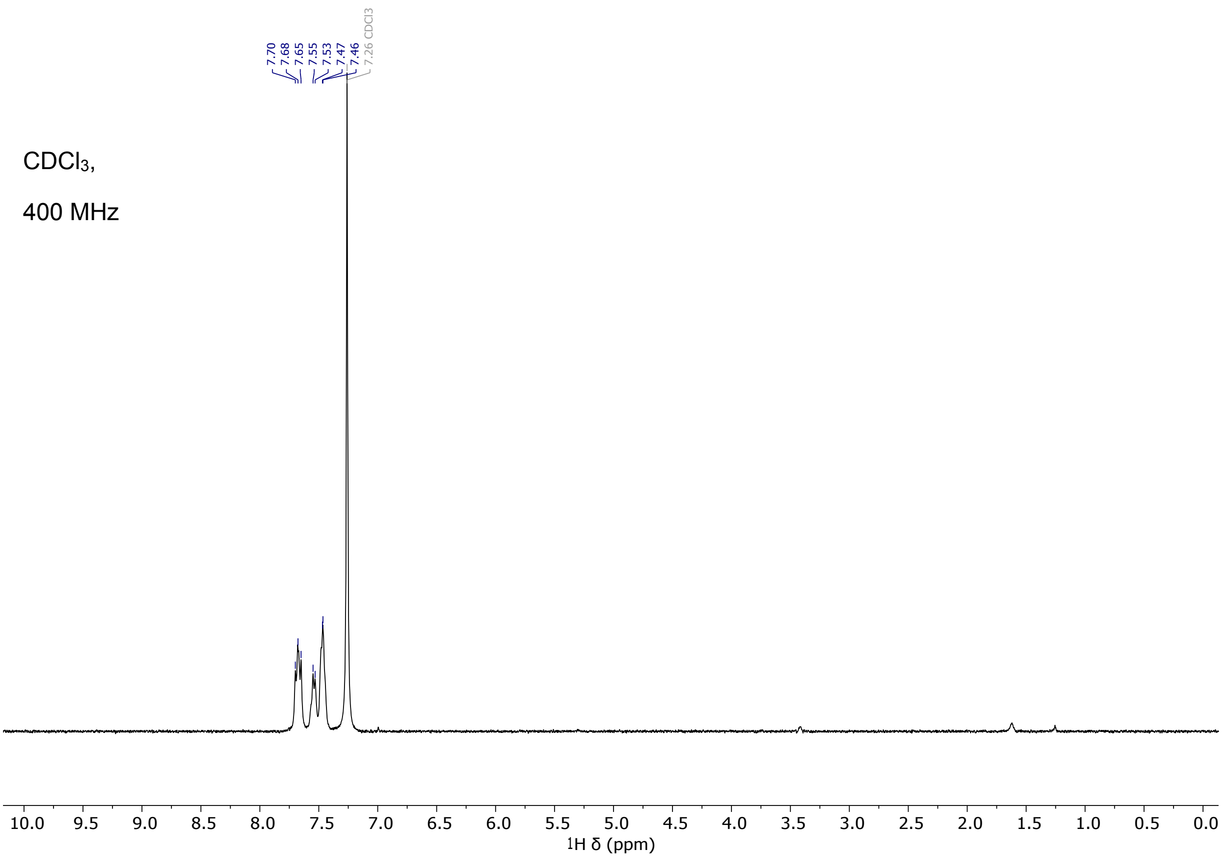

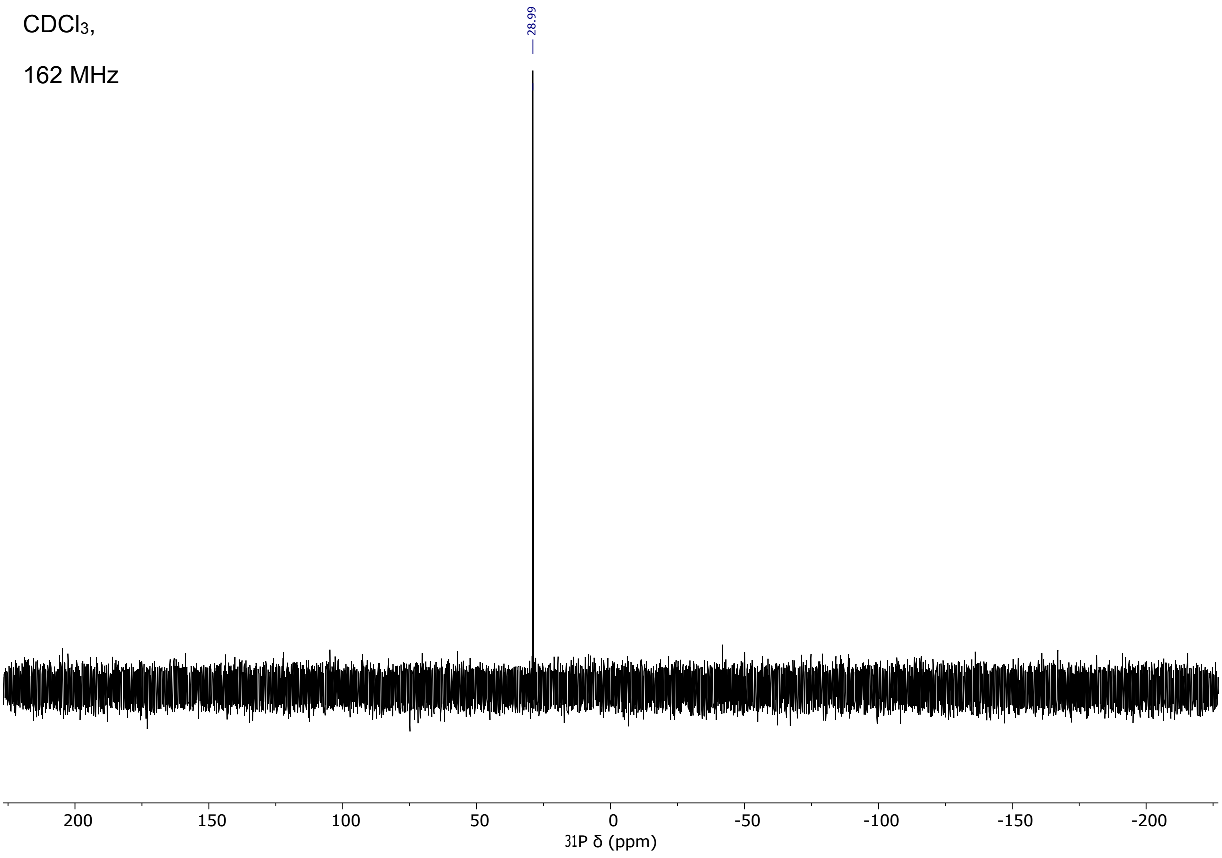

(*R*)-Diethyl 2-(5-phenylpent-4-yn-2-yl)malonate (4g)

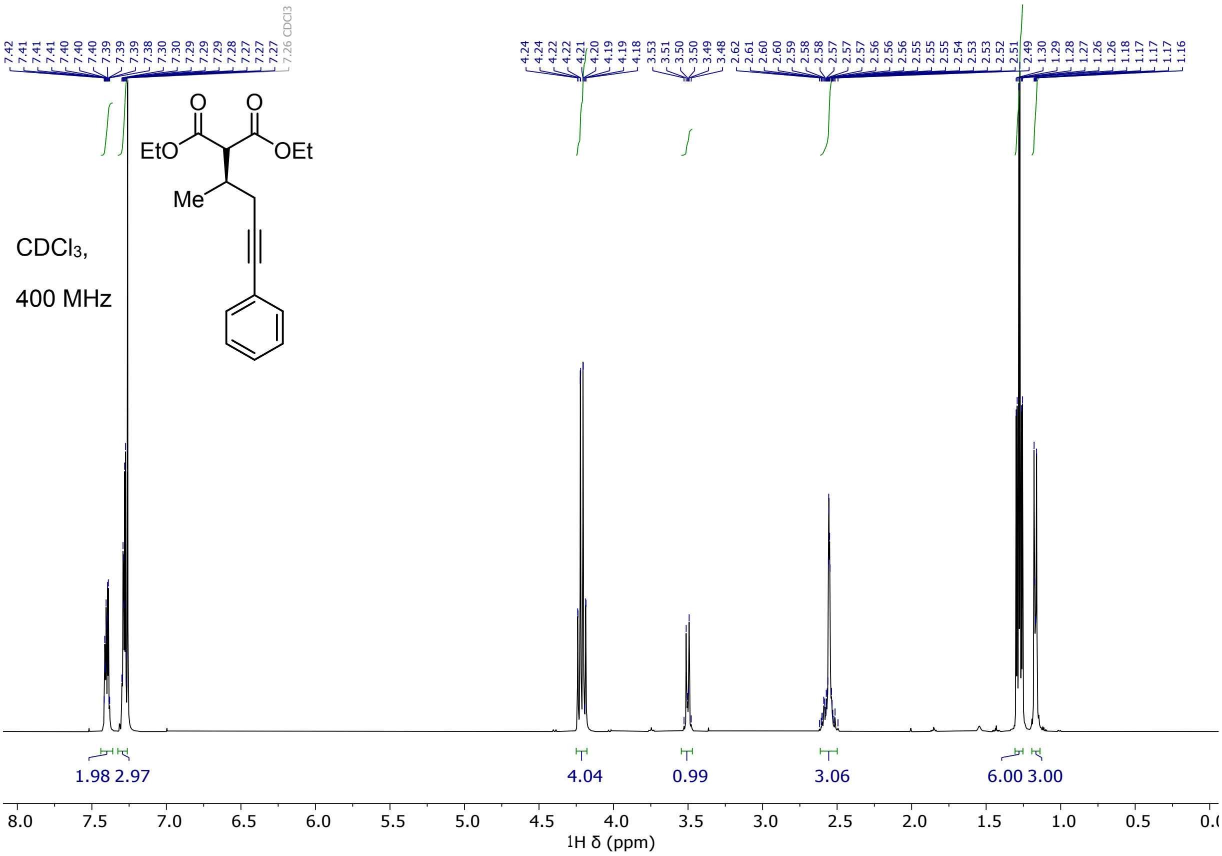

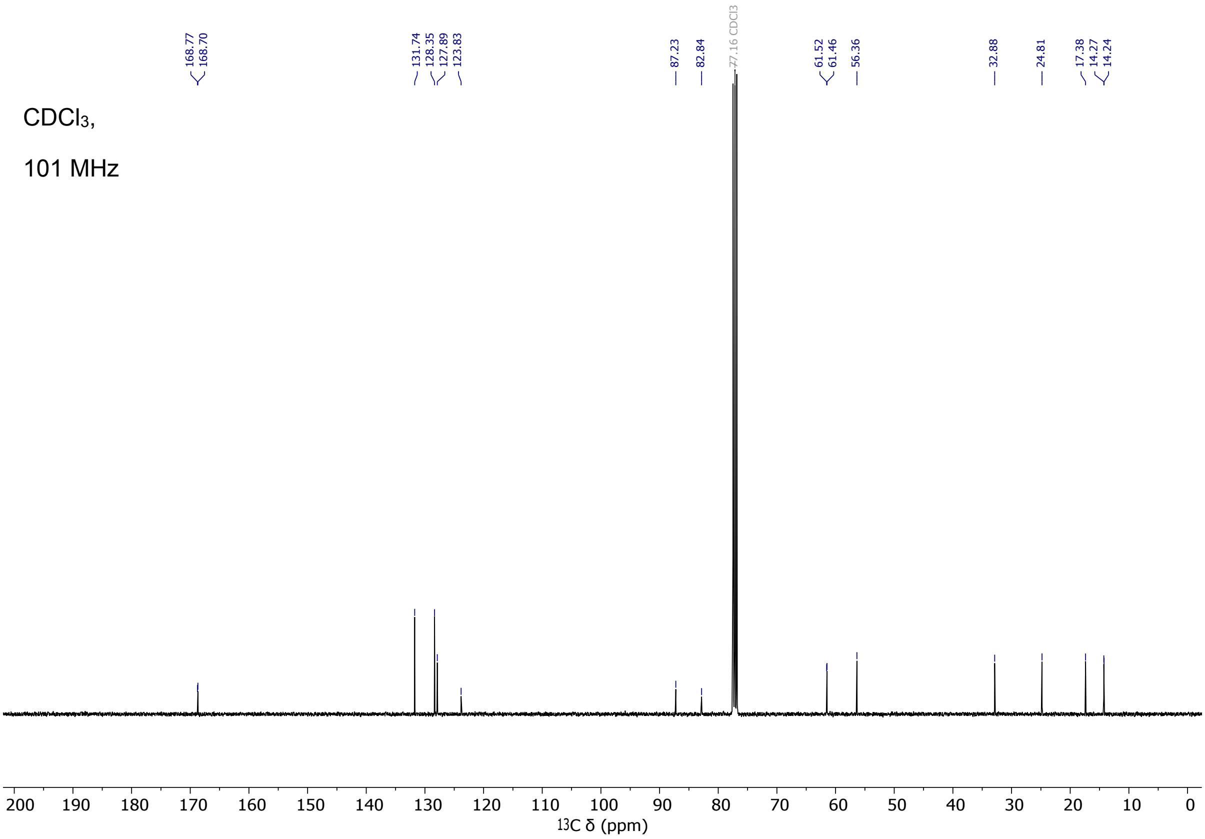

(*S*)-Diethyl 2-(1-(furan-2-yl)propan-2-yl)malonate (4h)

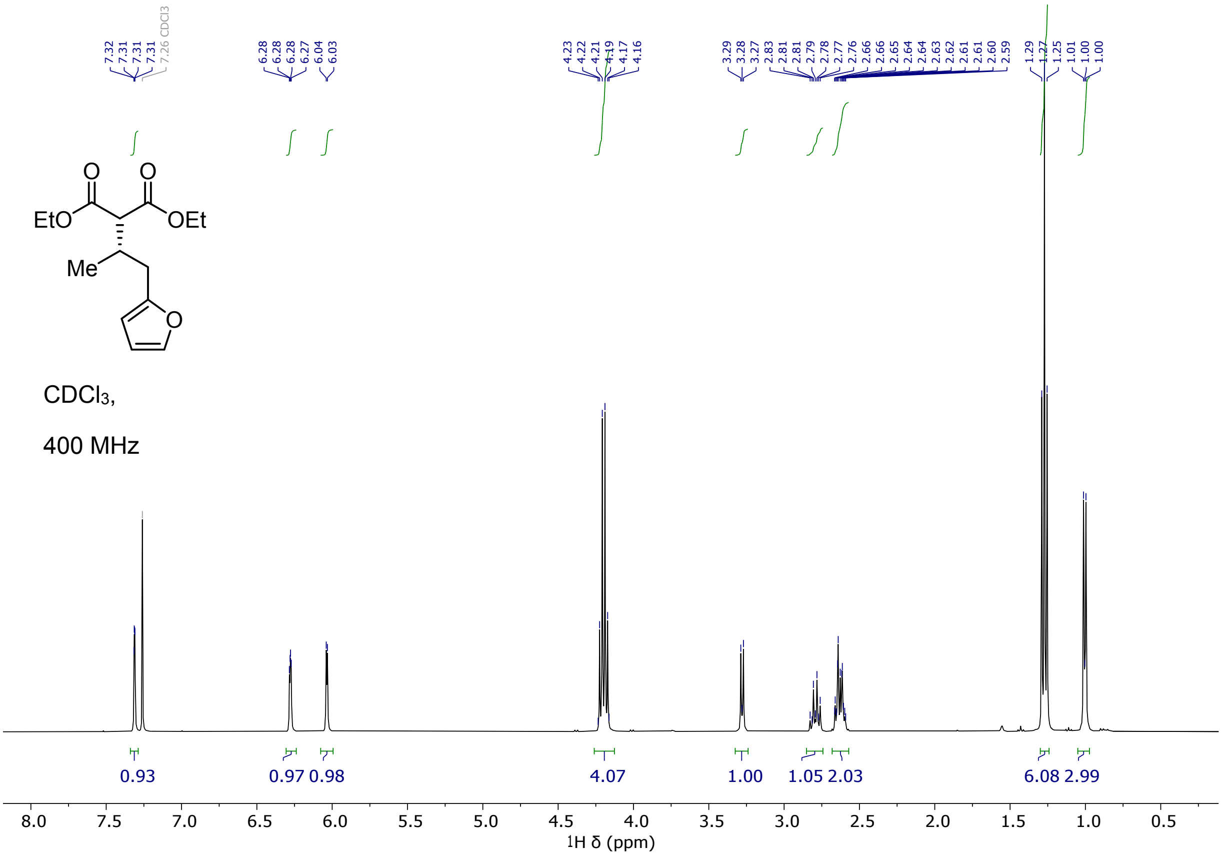

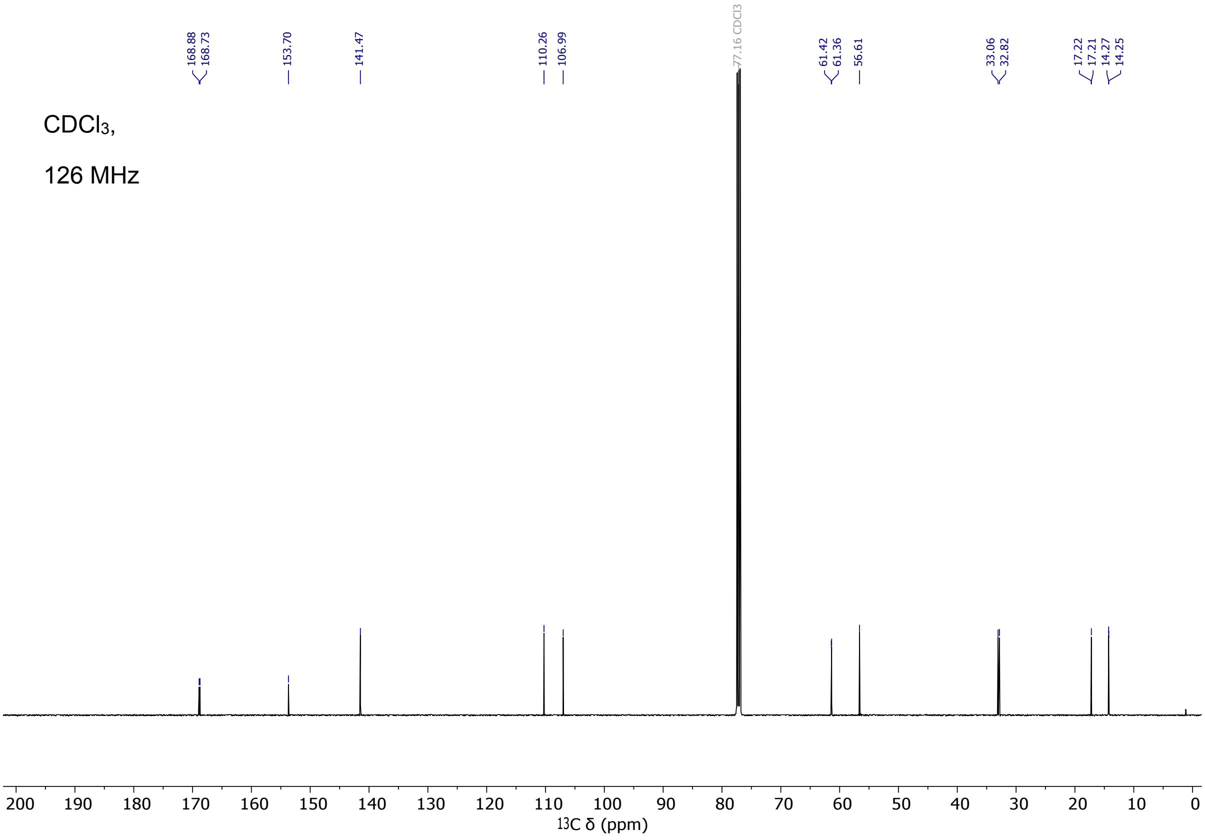

Diethyl 2-cyclohexylmalonate (4f)

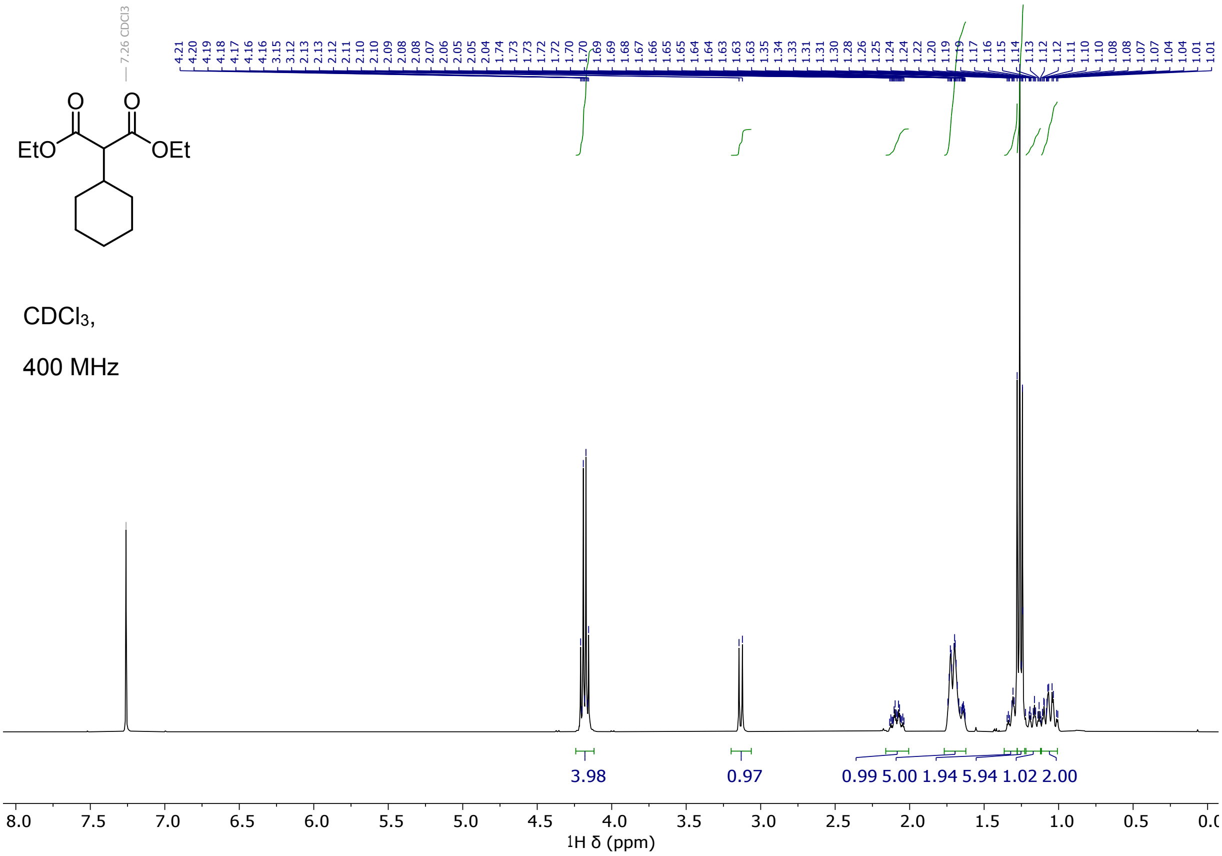

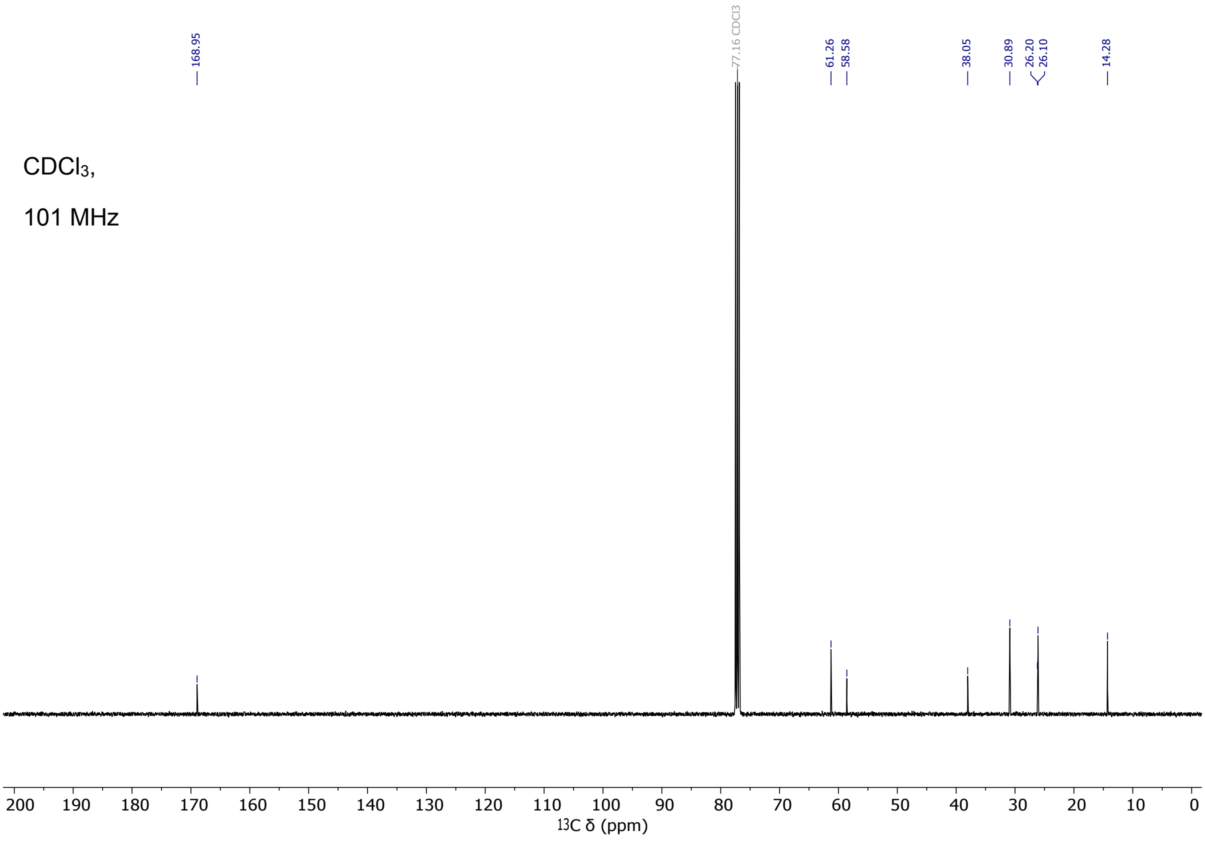

(*S*)-2-(4-phenylbutan-2-yl)malononitrile (4l)

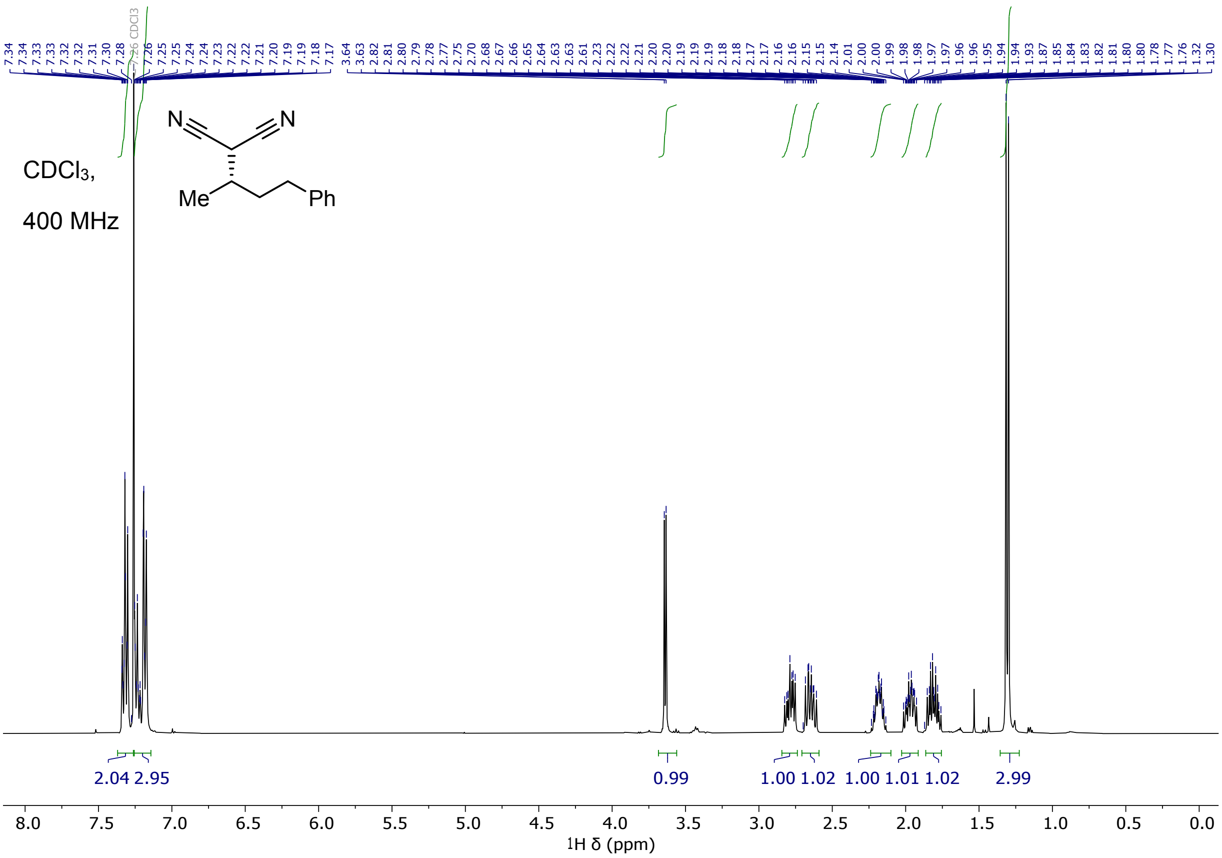

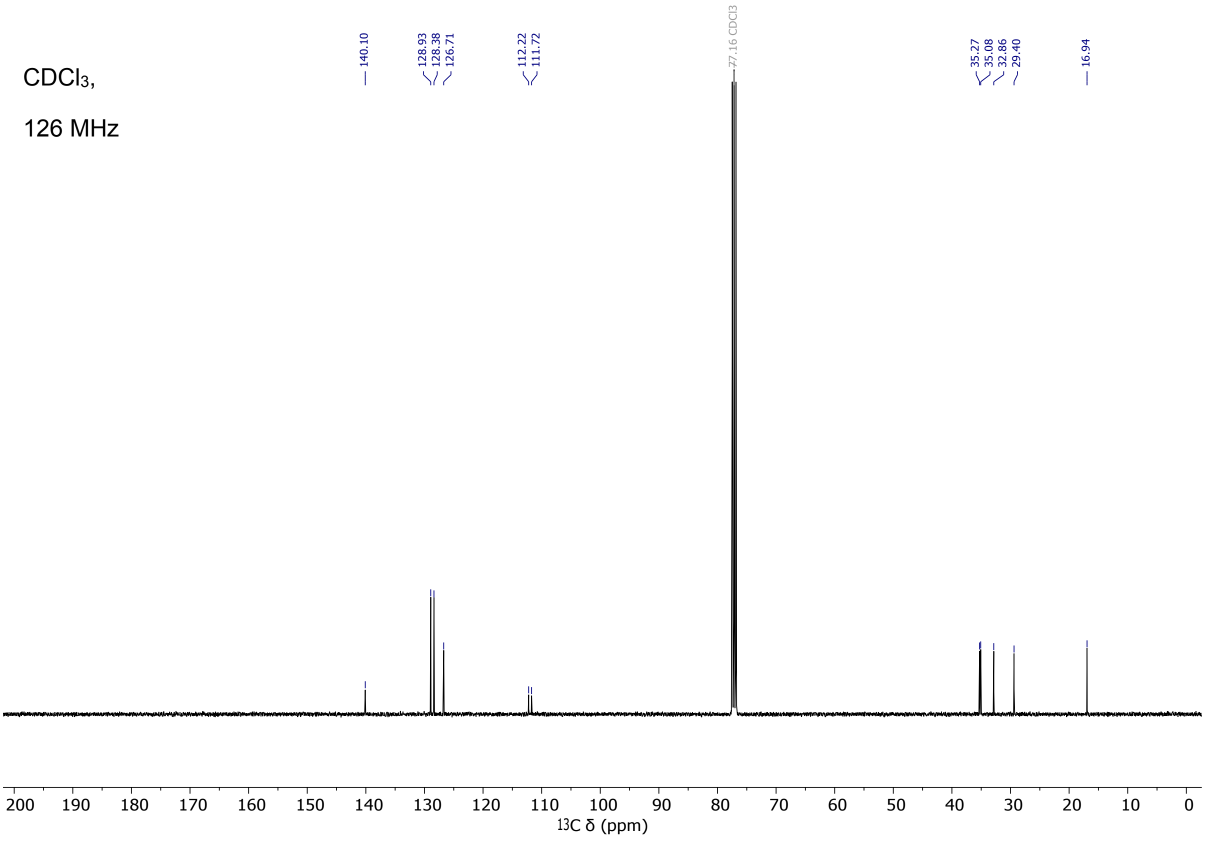

(*S*)-(2-methyl-4-phenylbutane-1,1-diyldisulfonyl)dibenzene (4k)

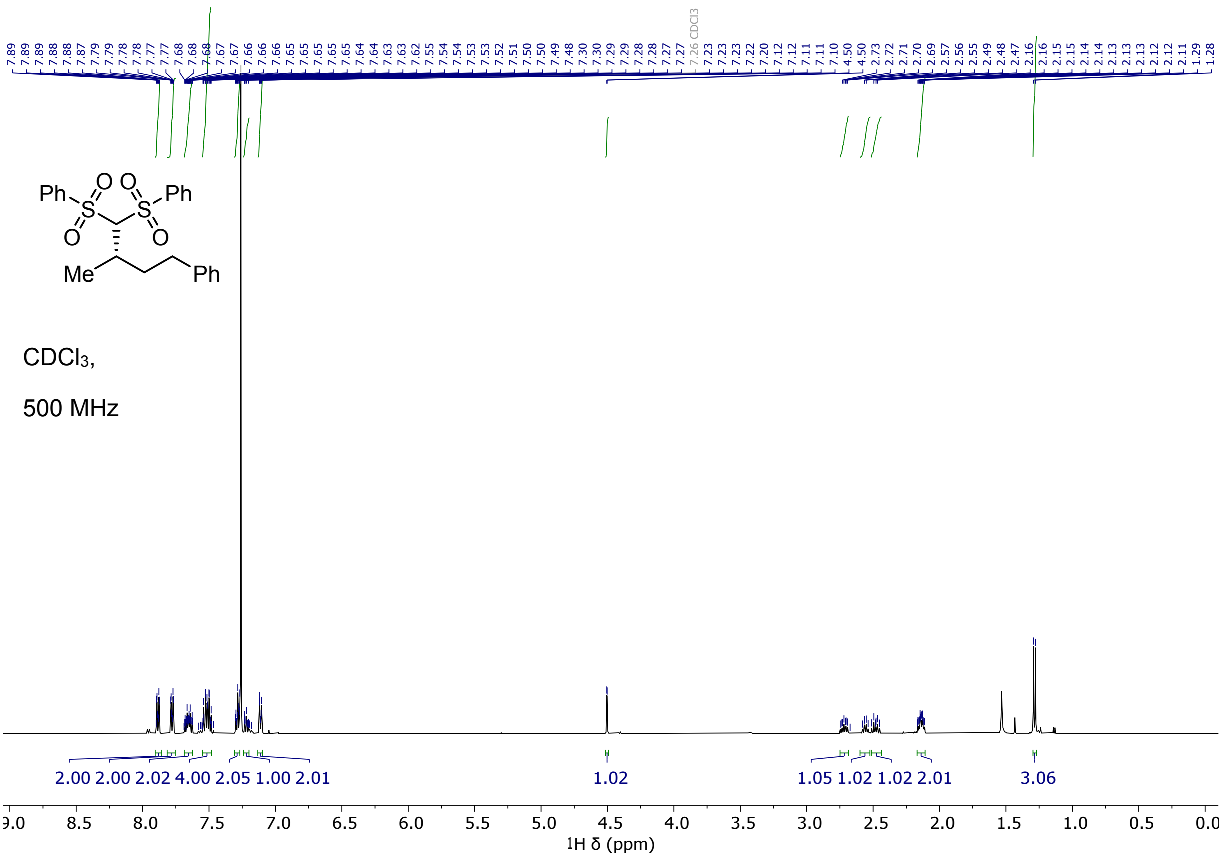

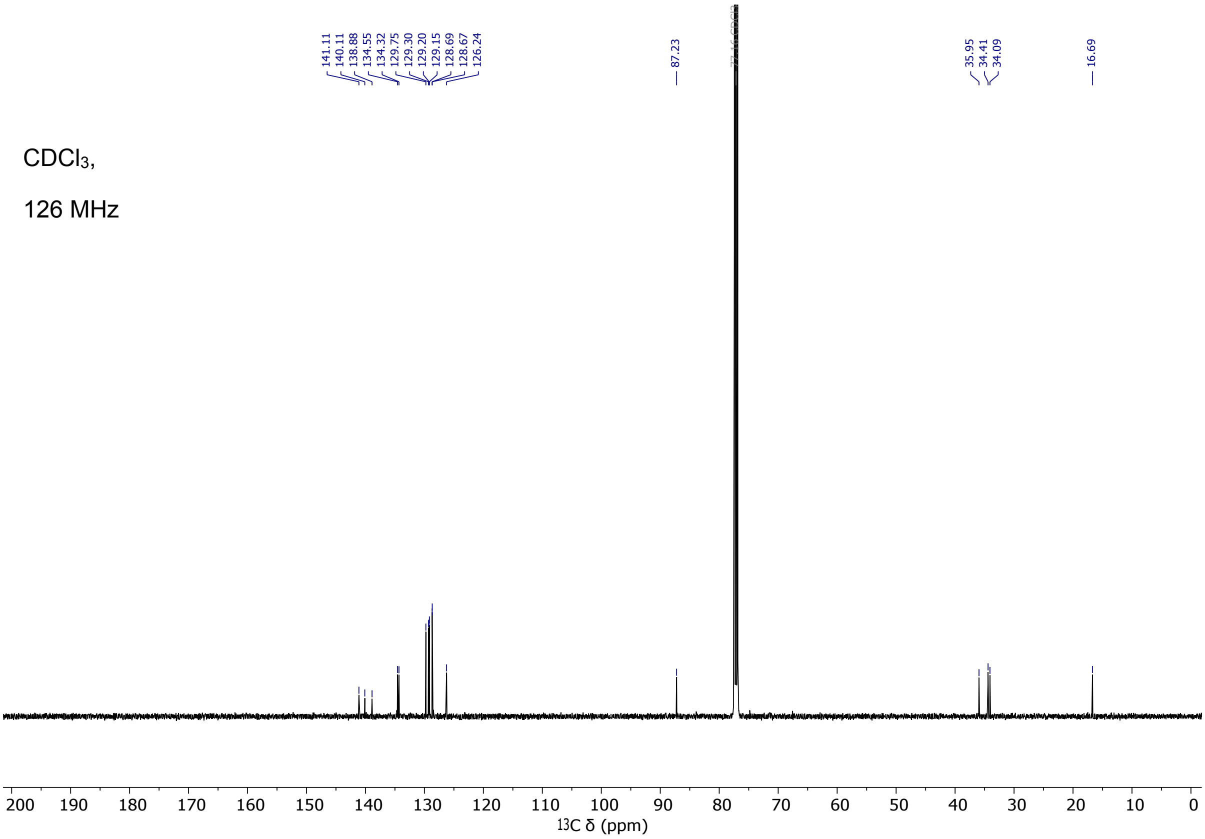

Products (from primary alcohols)

Diethyl 2-(5-chloropentyl)malonate (4m)

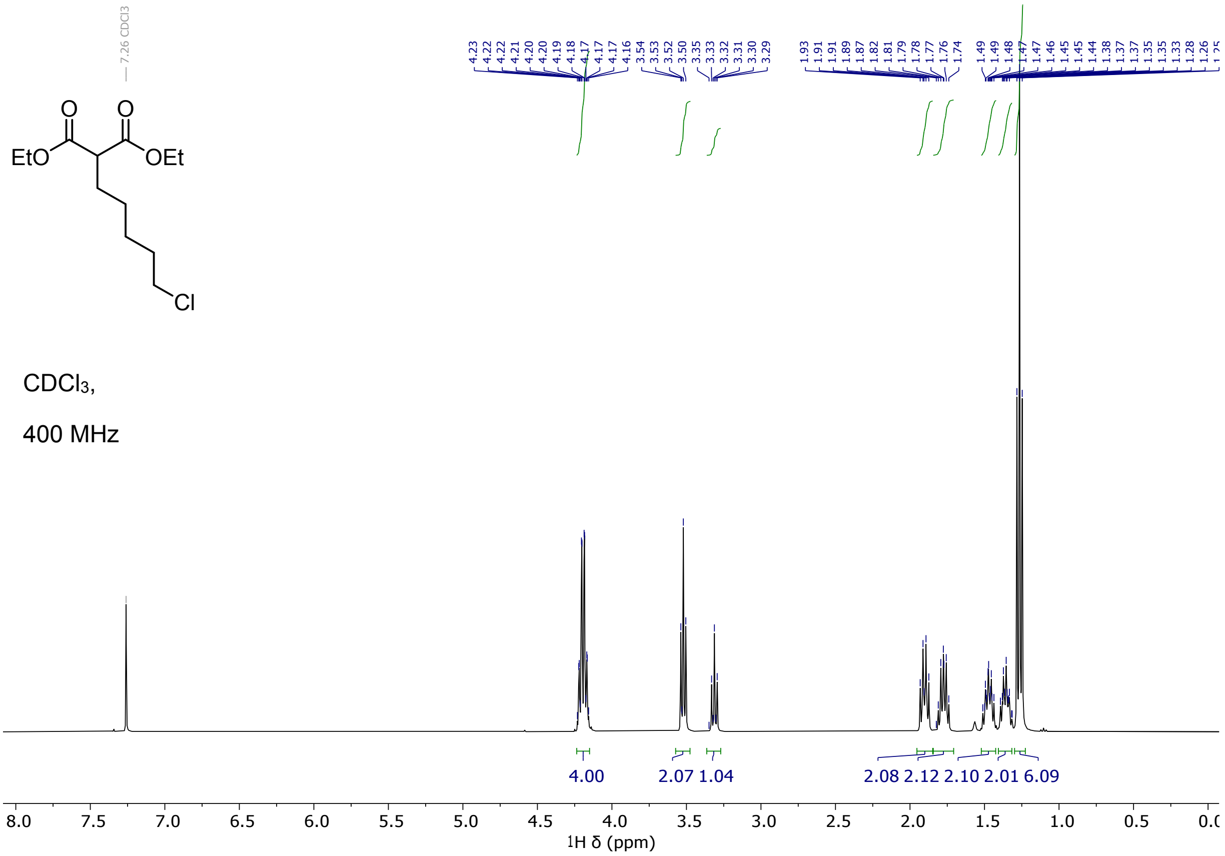

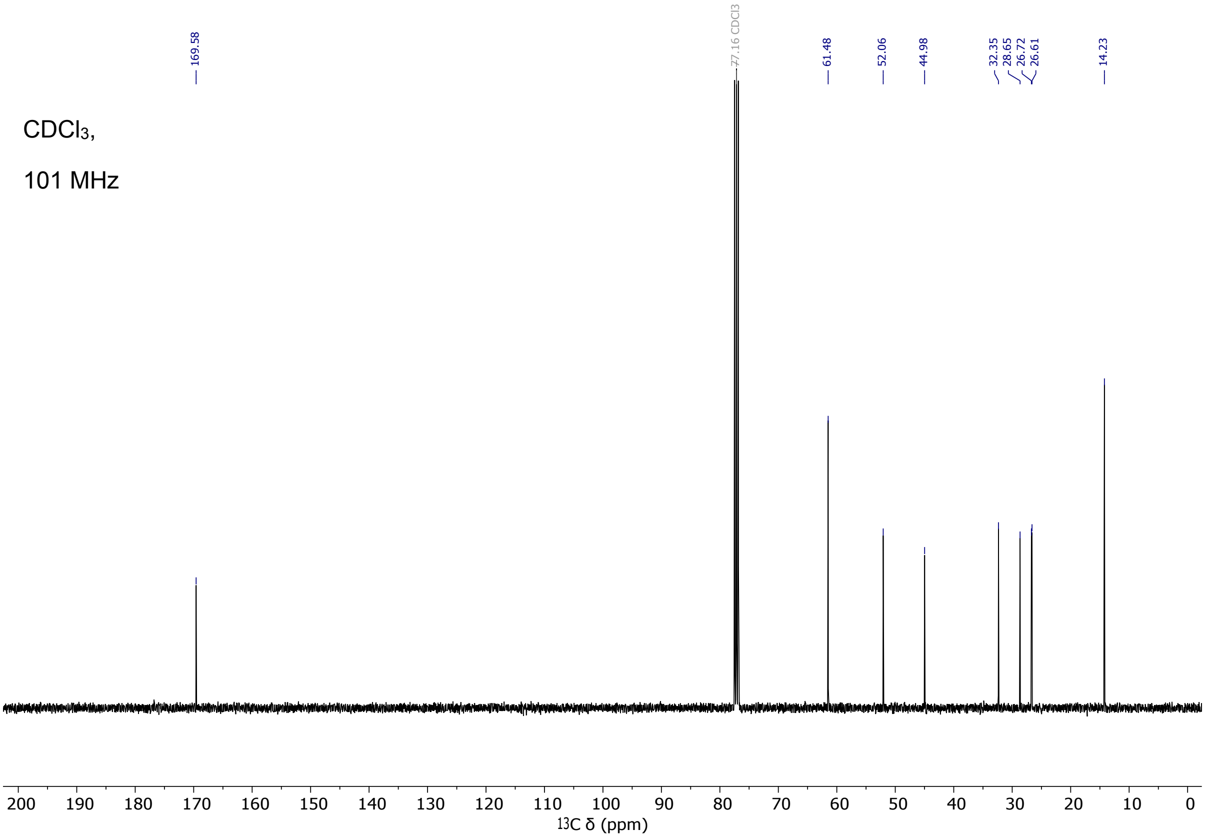

Diethyl 2-(2-fluorophenethyl)malonate (4o)

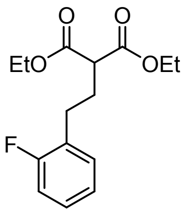

CDCl3,

400 MHz

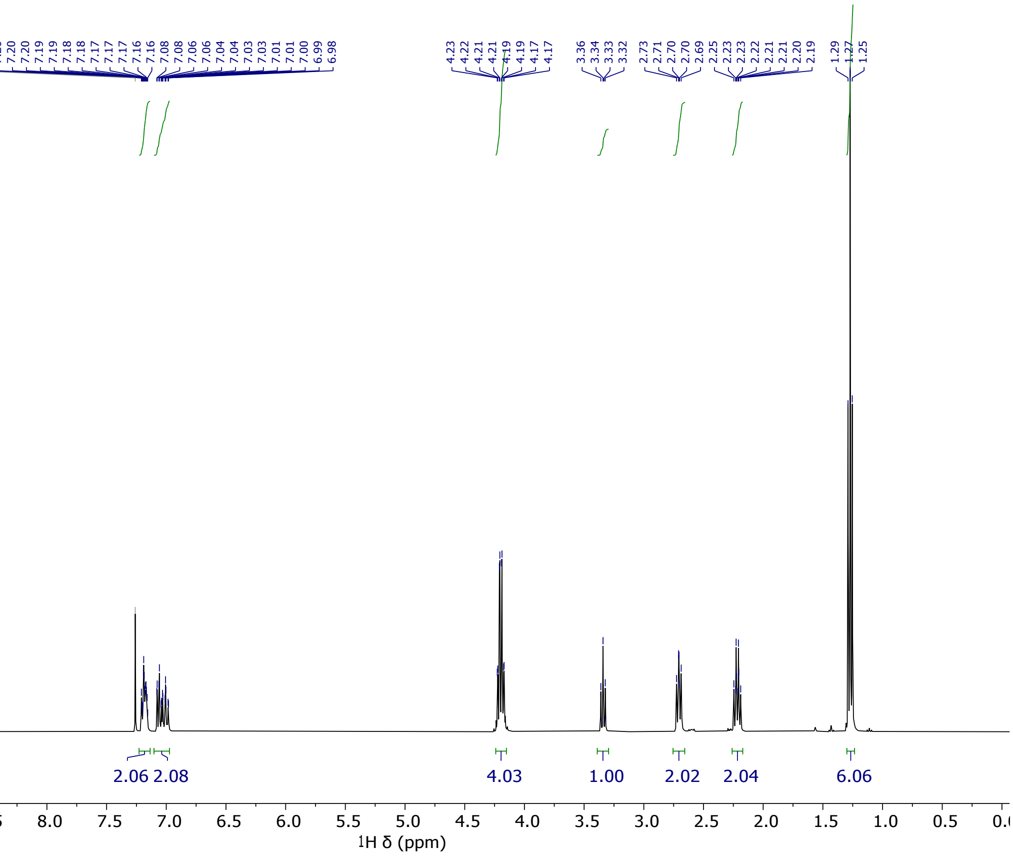

CDCl3,

101 MHz

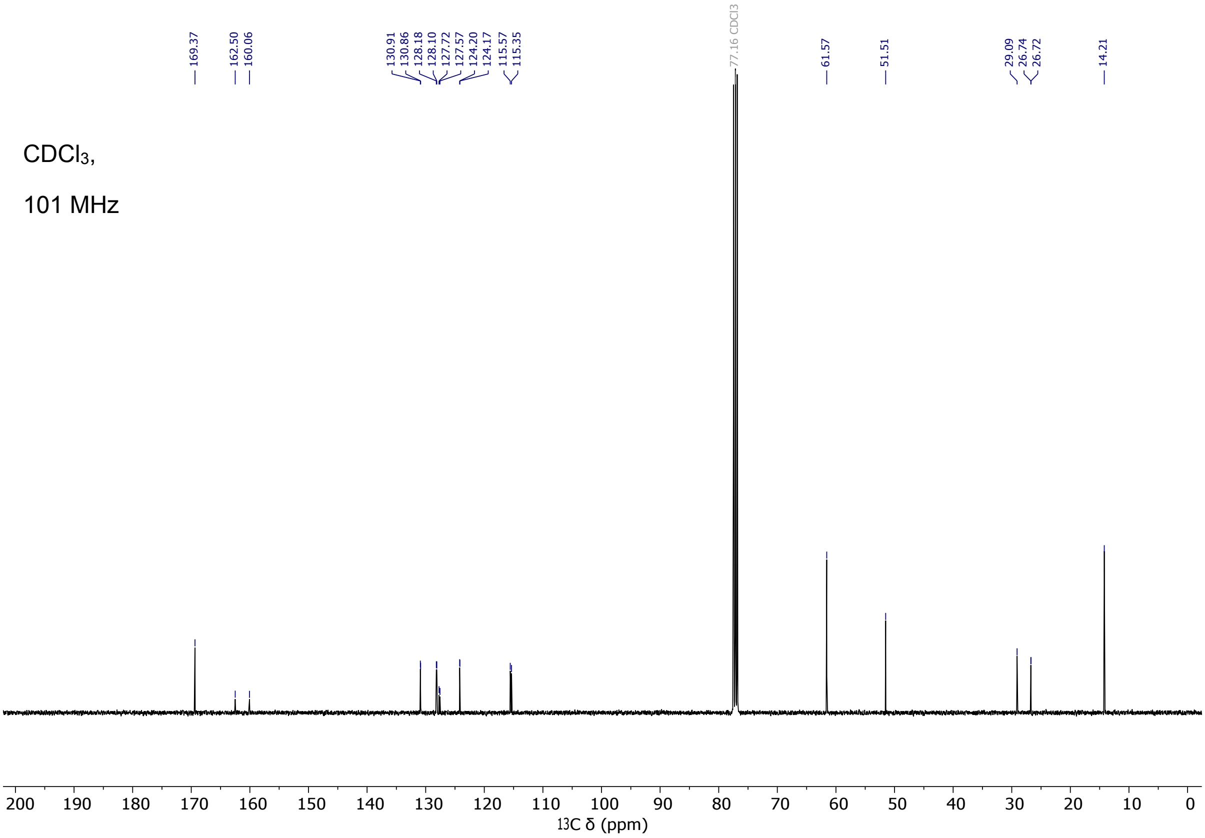

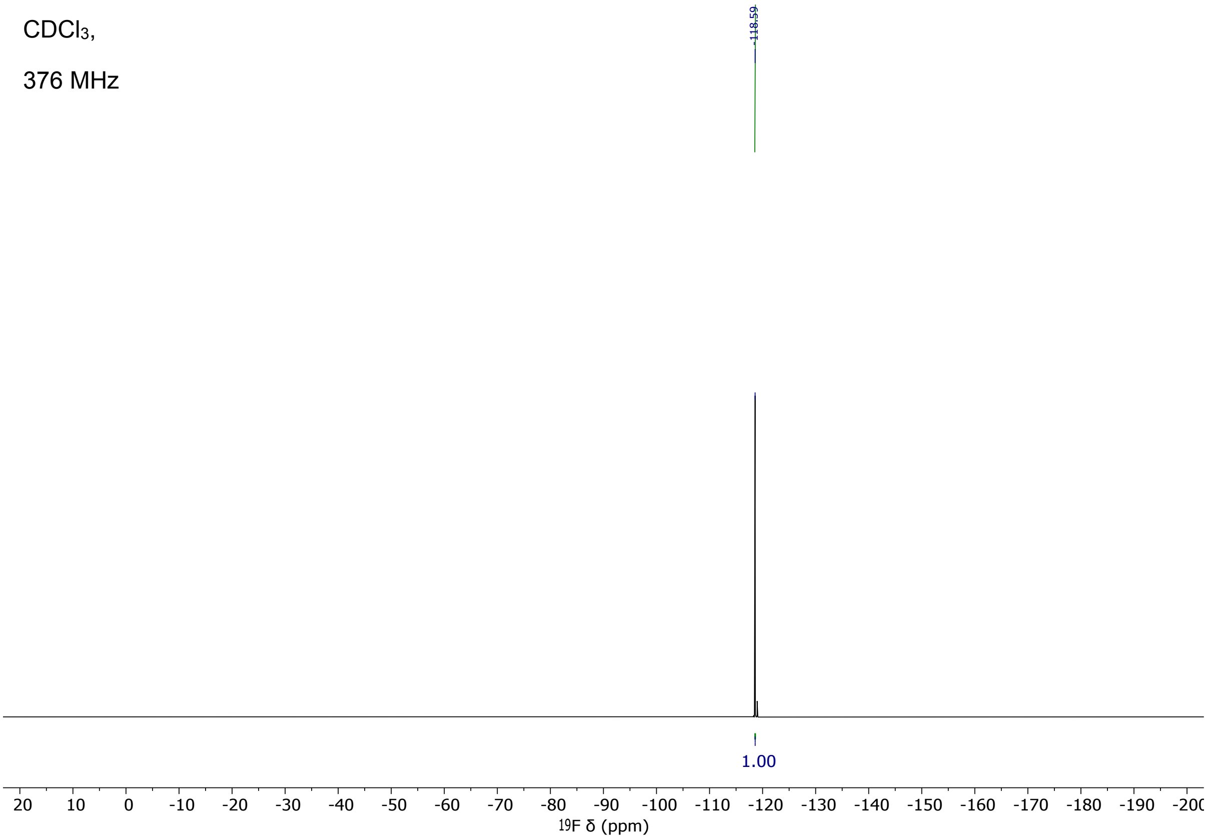
CDCl3,
376 MHz
-118.59
1.00
20 10 0 -10 -20 -30 -40 -50 -60 -70 -80 -90 -100 -110 -120 -130 -140 -150 -160 -170 -180 -190 -200
19F δ (ppm)

3-benzyl 1,1-diethyl (*R*)-butane-1,1,3-tricarboxylate (4q)

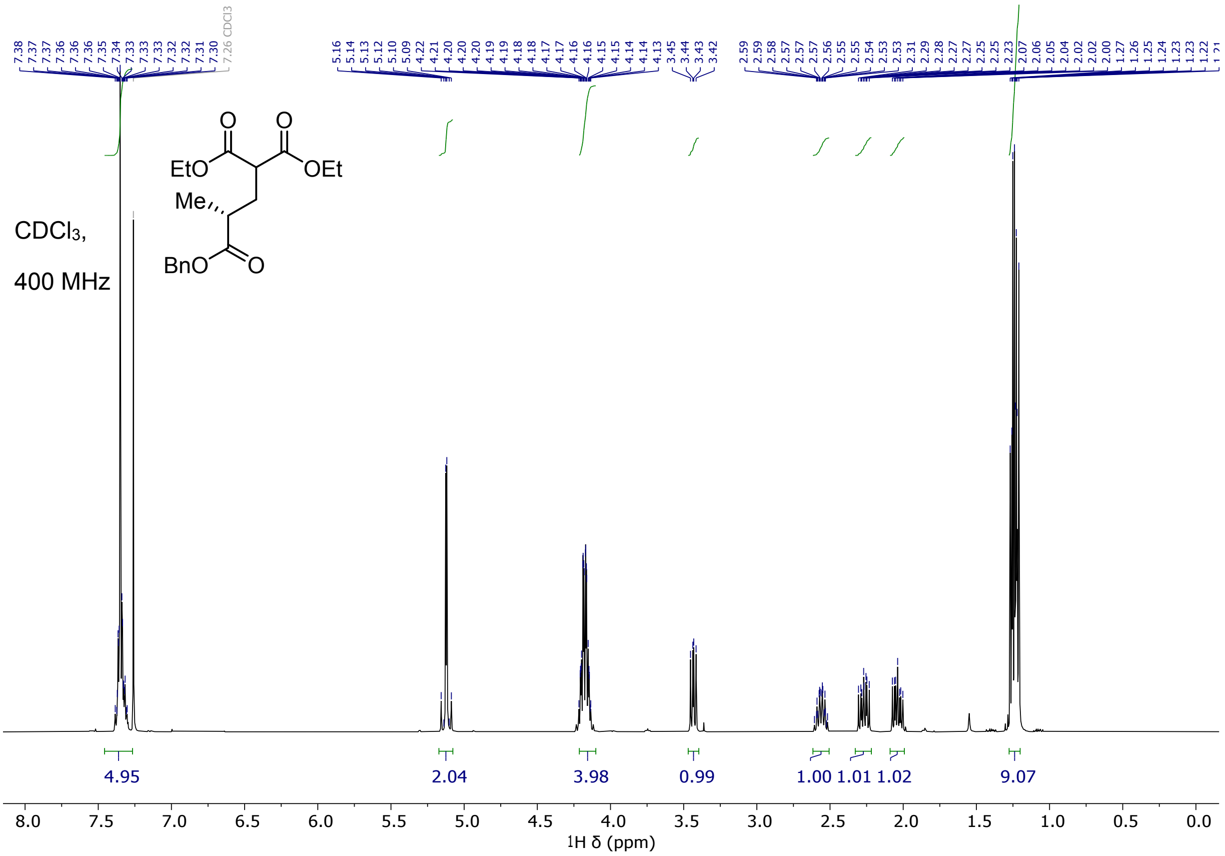

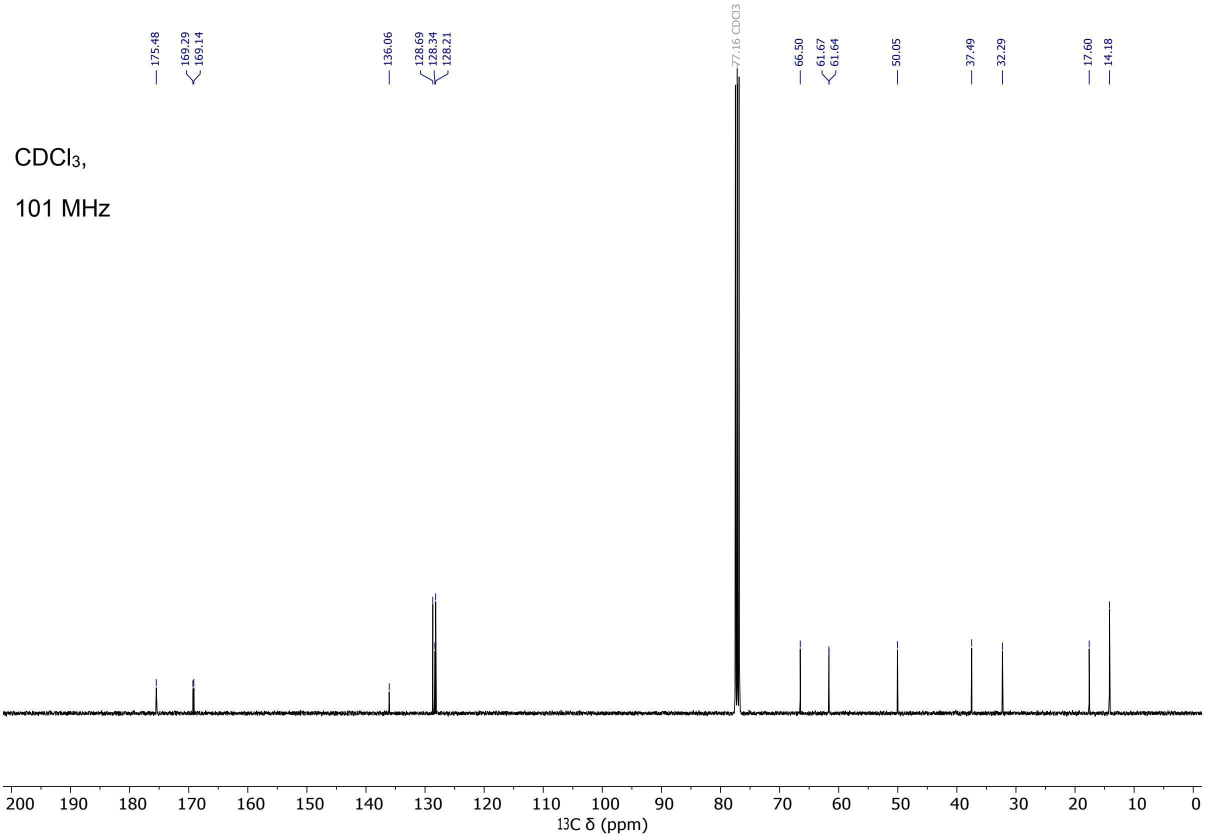

Diethyl 2-(3-(dimethylamino)-3-oxopropyl)malonate (4p)

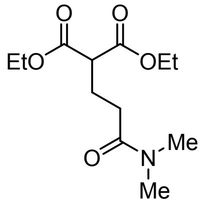

CDCl3,

500 MHz

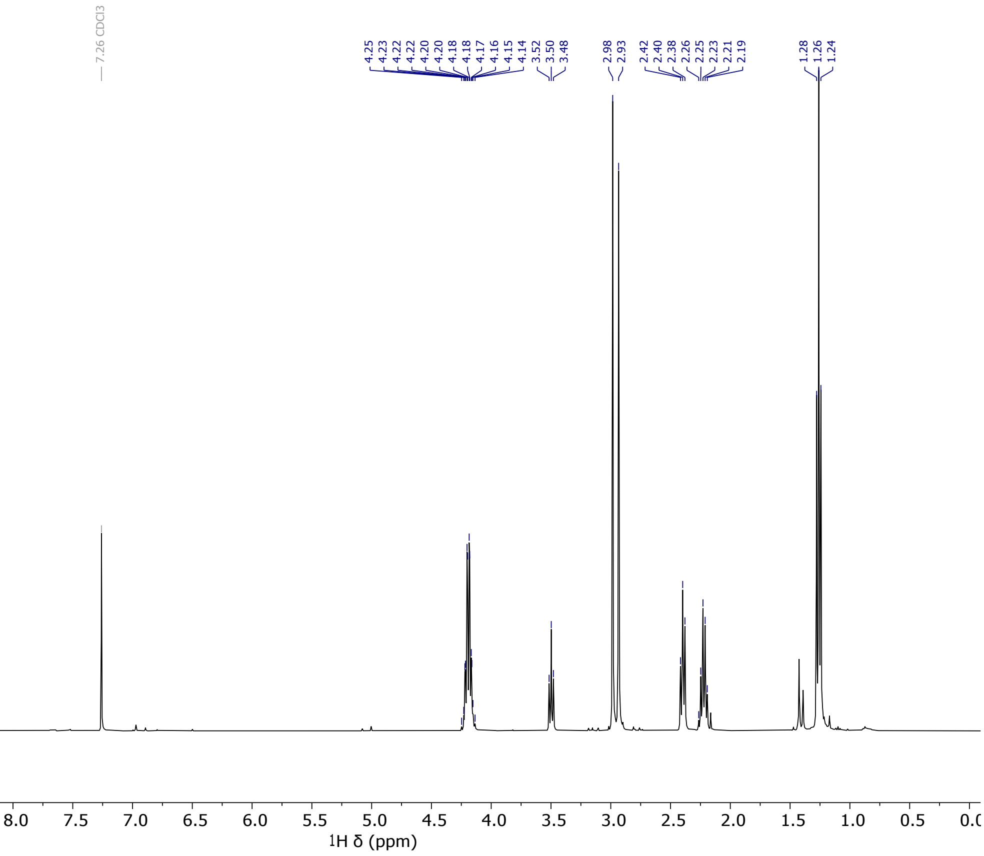

CDCl3,

126 MHz

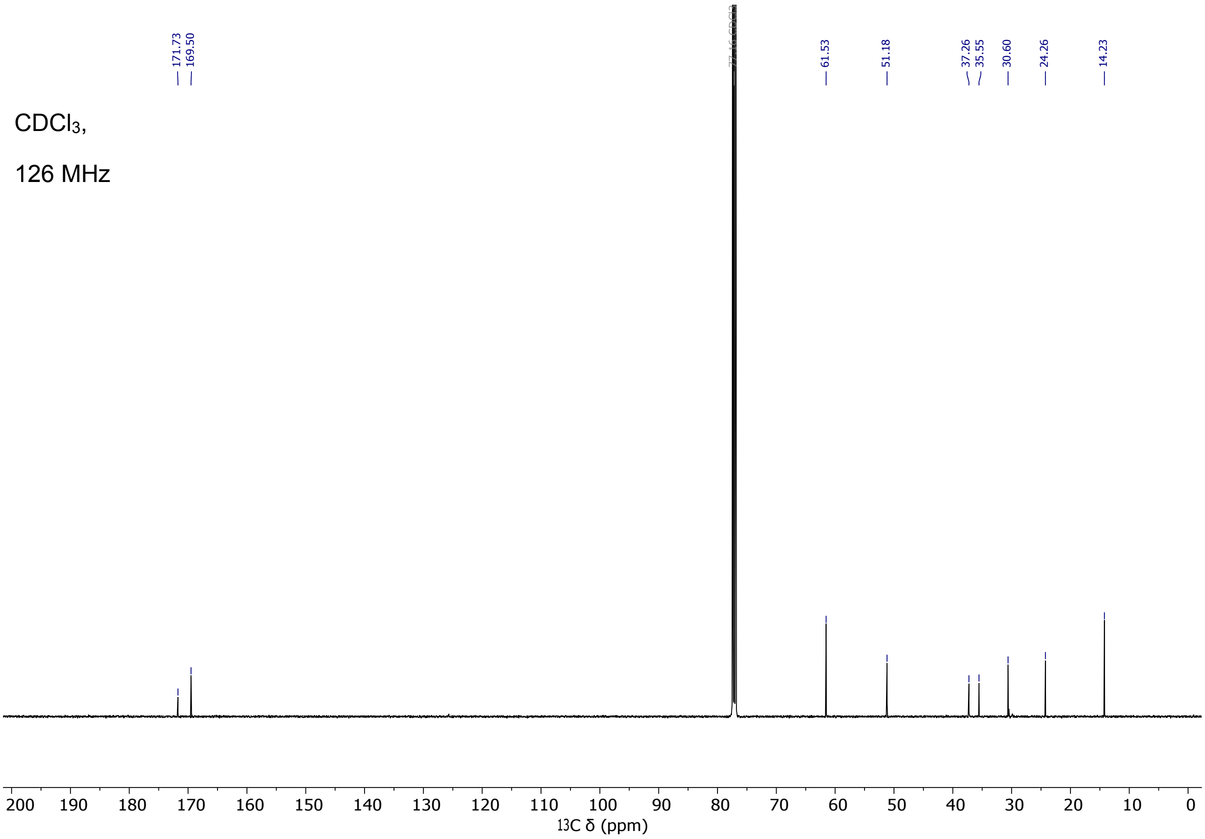

Diethyl 2-(5-bromopentyl)malonate (4n)

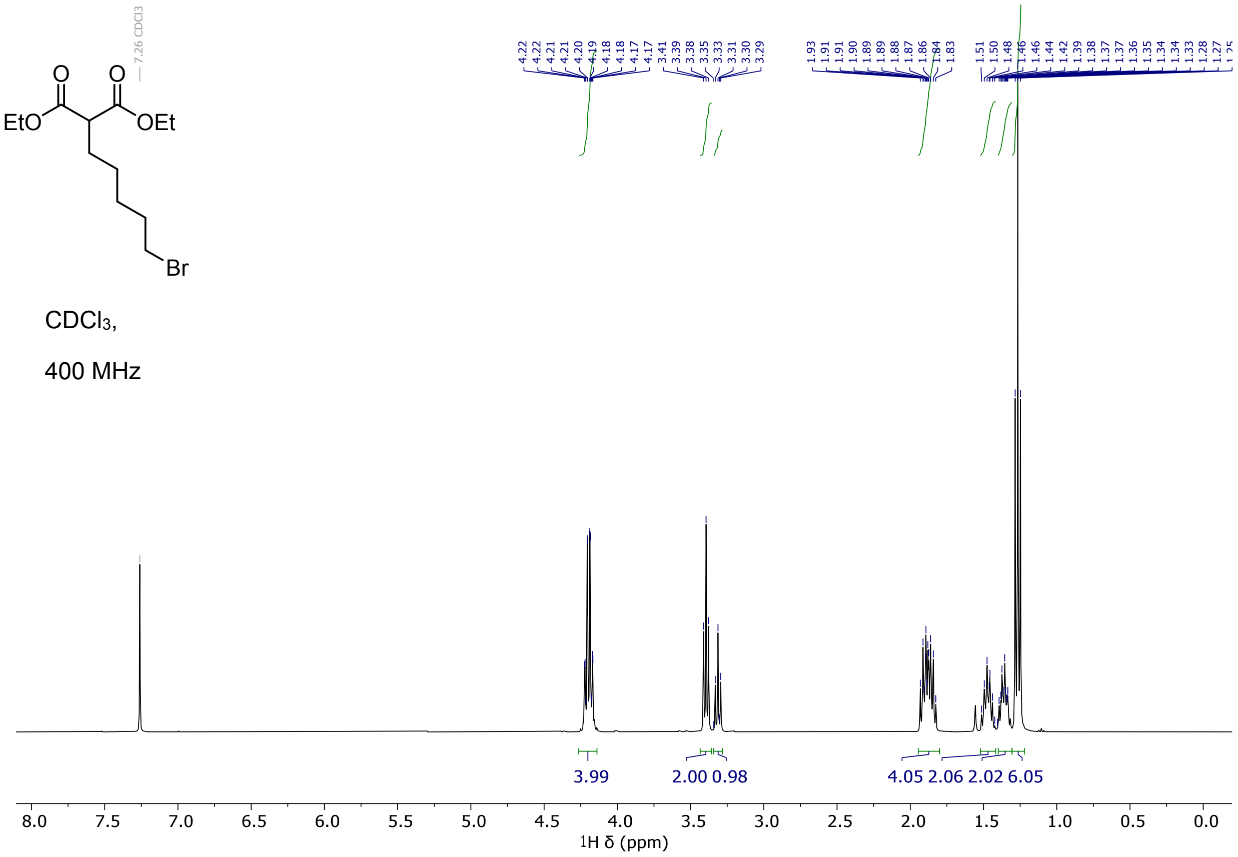

CDCl3,

400 MHz

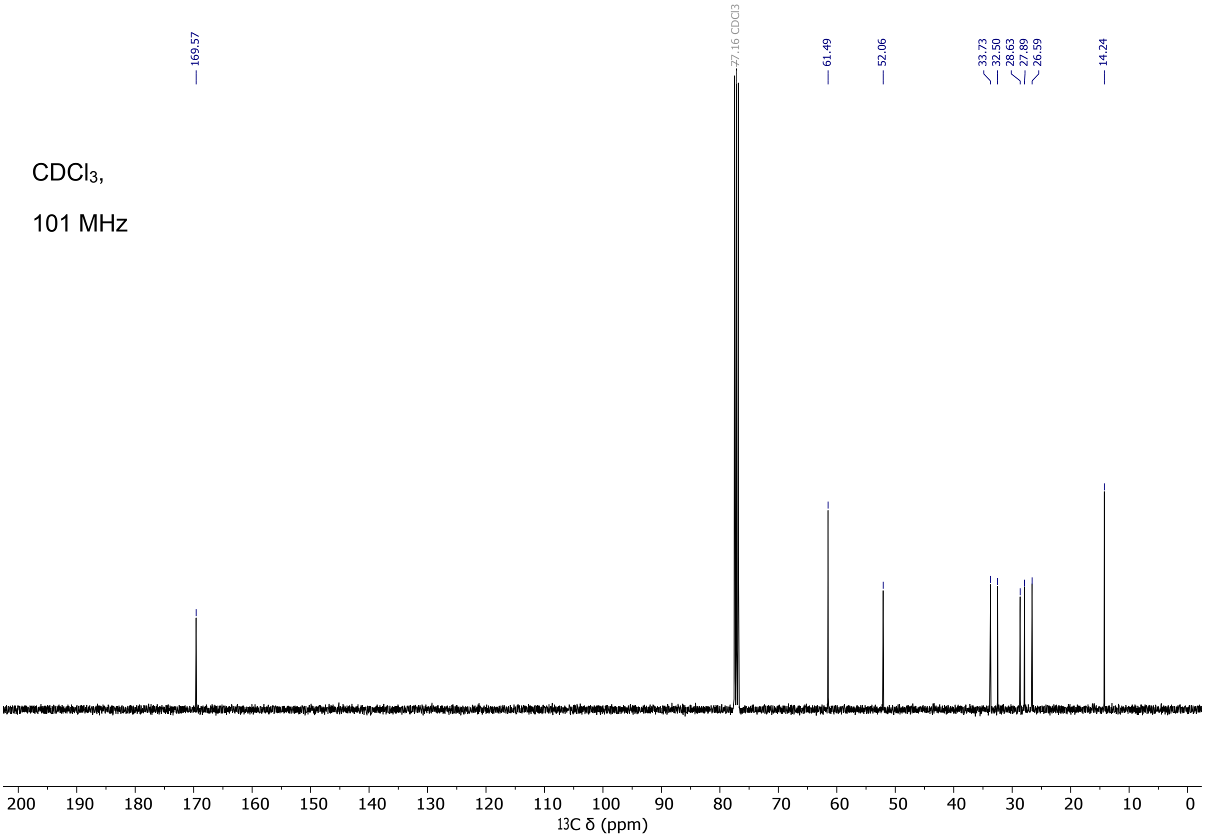

CDCl3,

101 MHz

Diethyl 2-(2-(pyridin-2-yl)ethyl)malonate (4r)

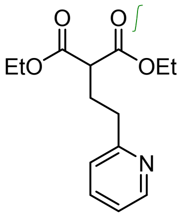

CDCl$_3$,

400 MHz

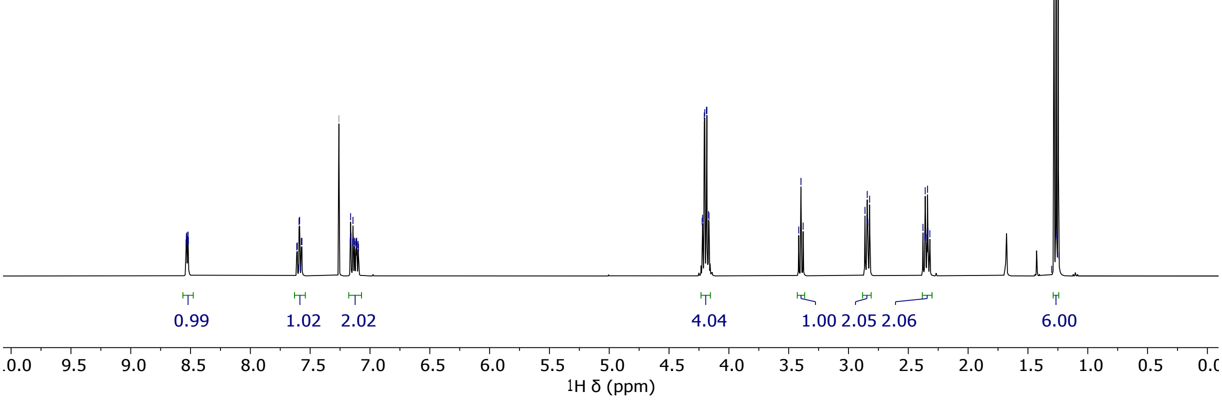

CDCl$_3$,

101 MHz

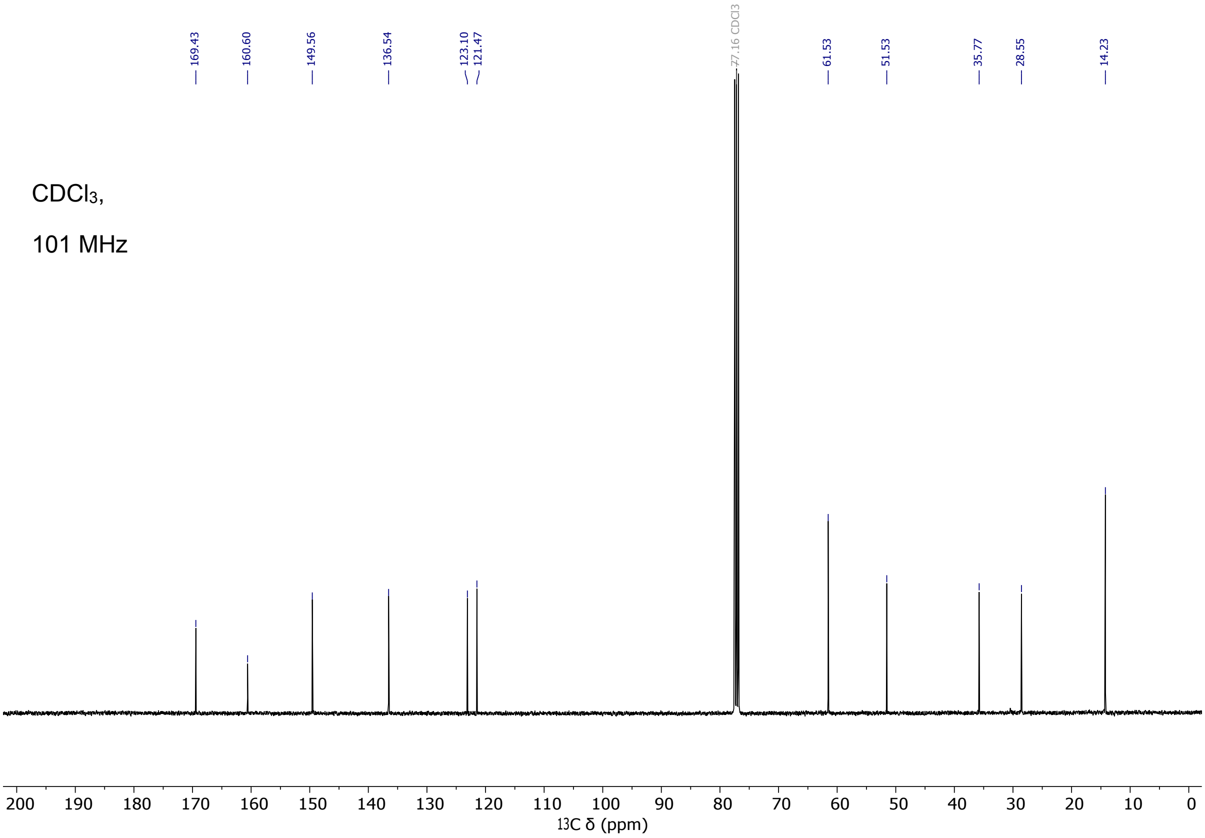

Ethyl 2-acetyl-4-(pyridin-2-yl)butanoate (4s)

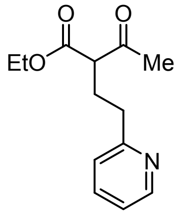

$CDCl_3$,

400 MHz

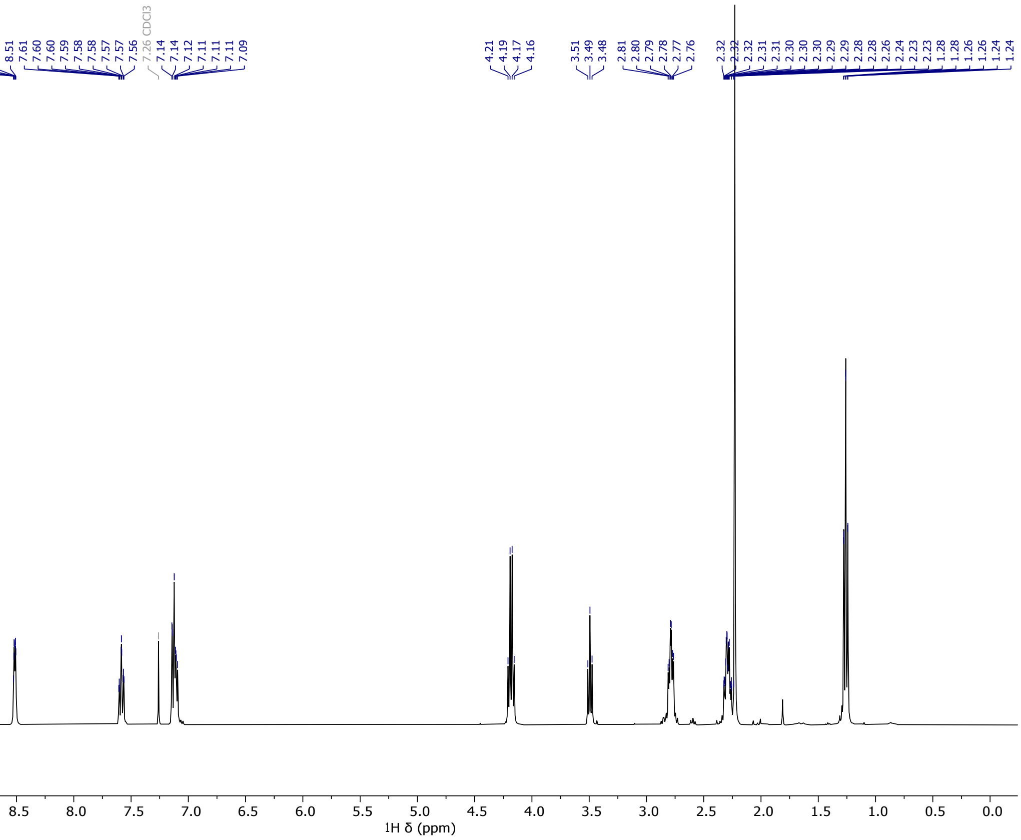

$CDCl_3$,

101 MHz

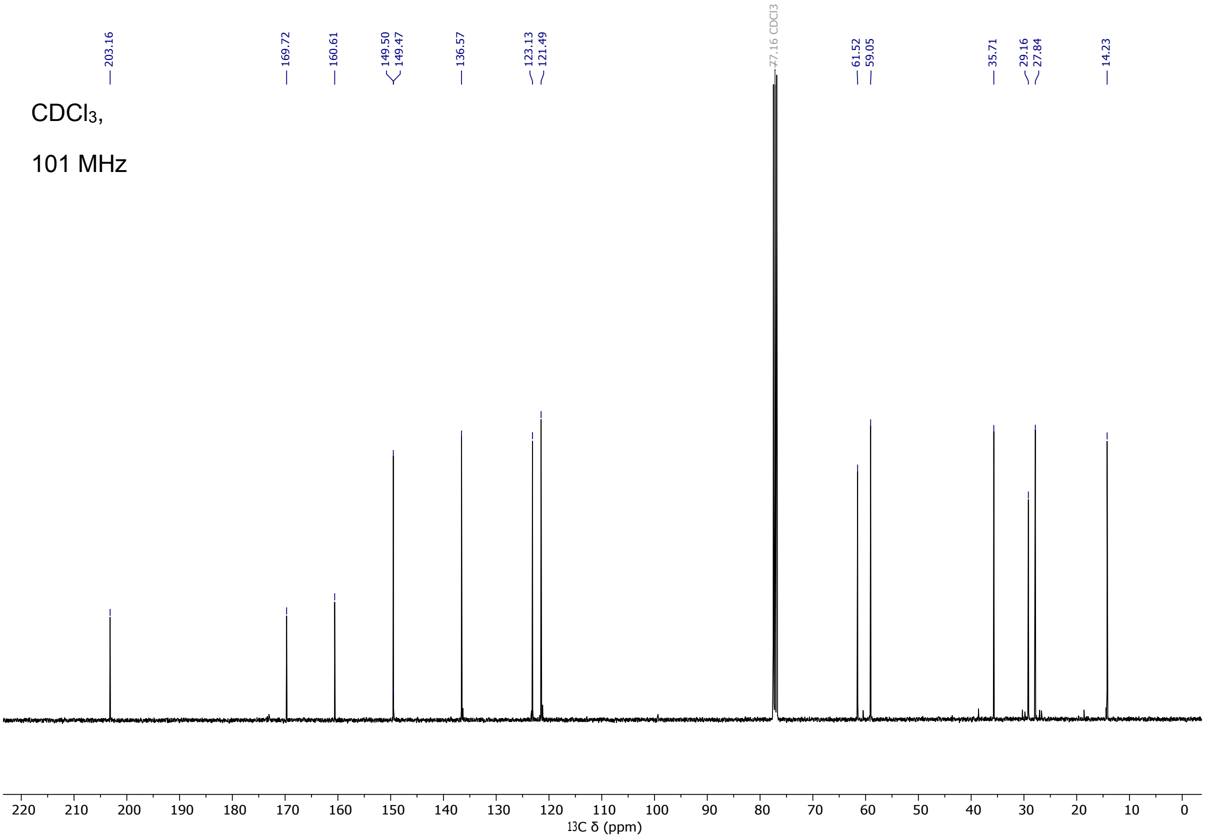

Diethyl 2-methyl-2-(2-(pyridin-2-yl)ethyl)malonate (4t)

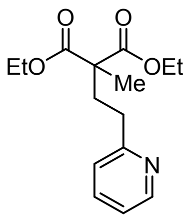

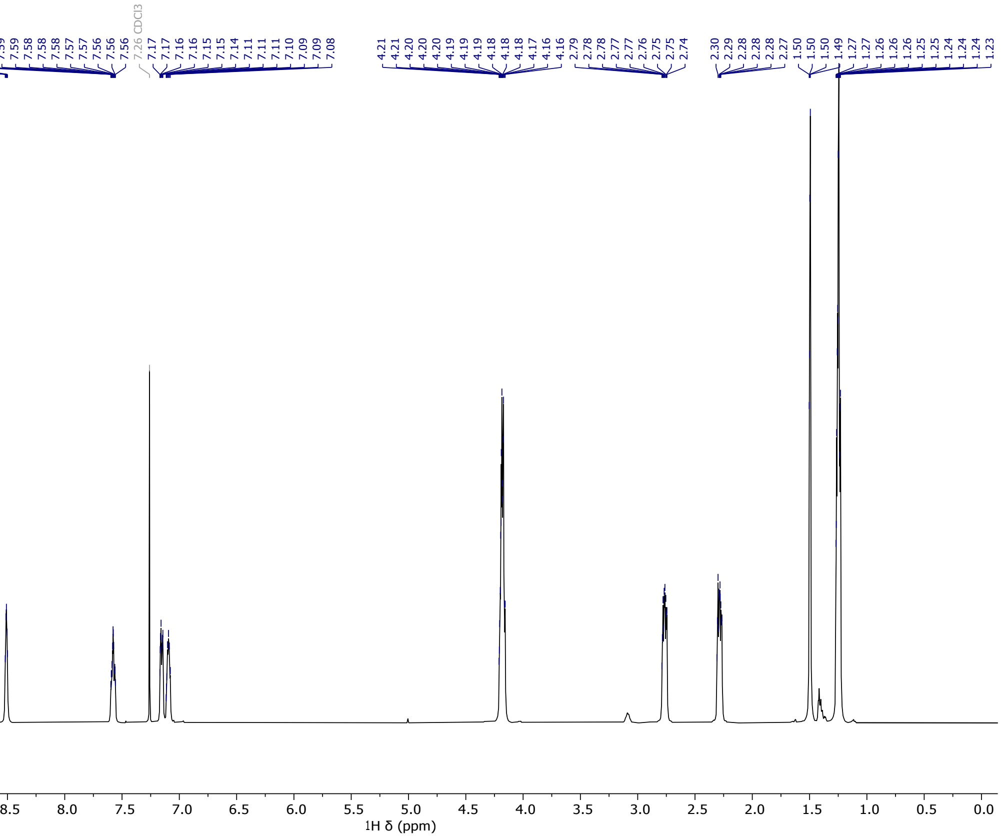

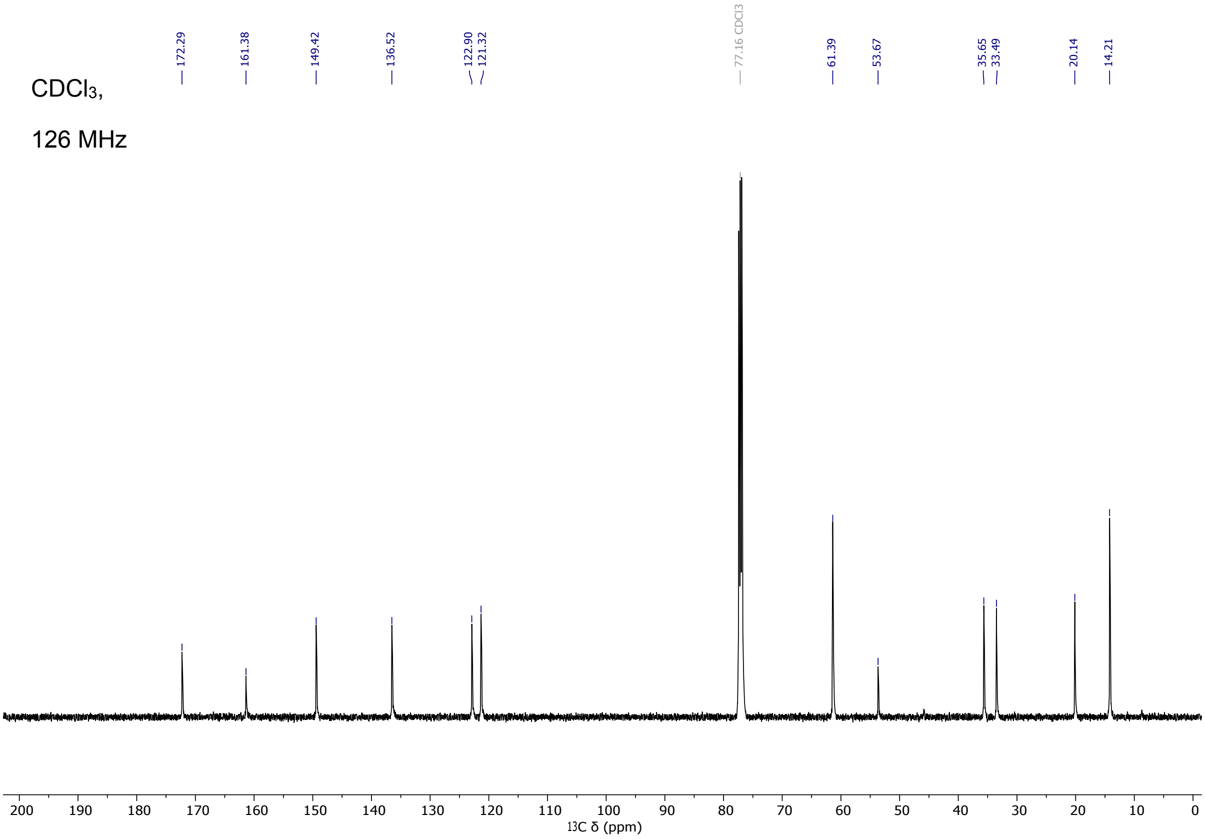

Other

(S)-octan-2-yl 4-nitrobenzoate (S24)

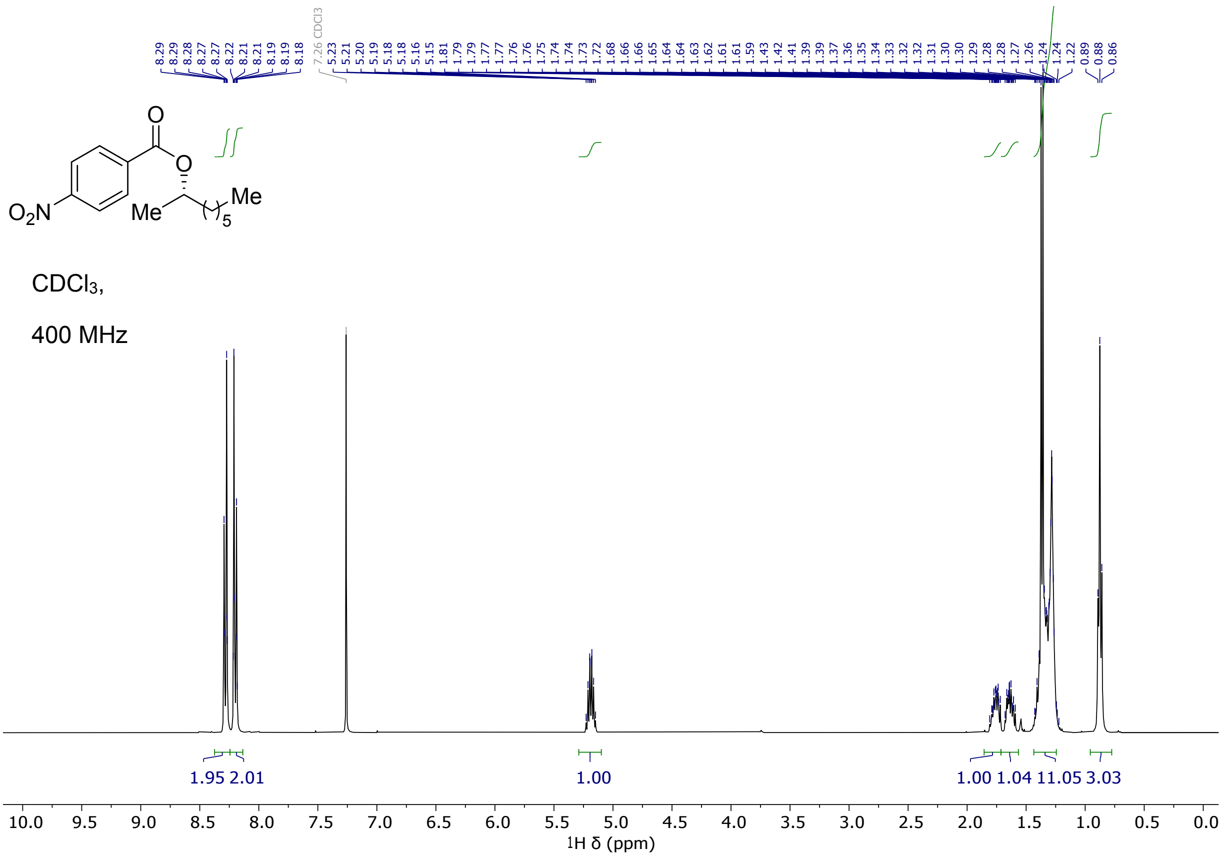

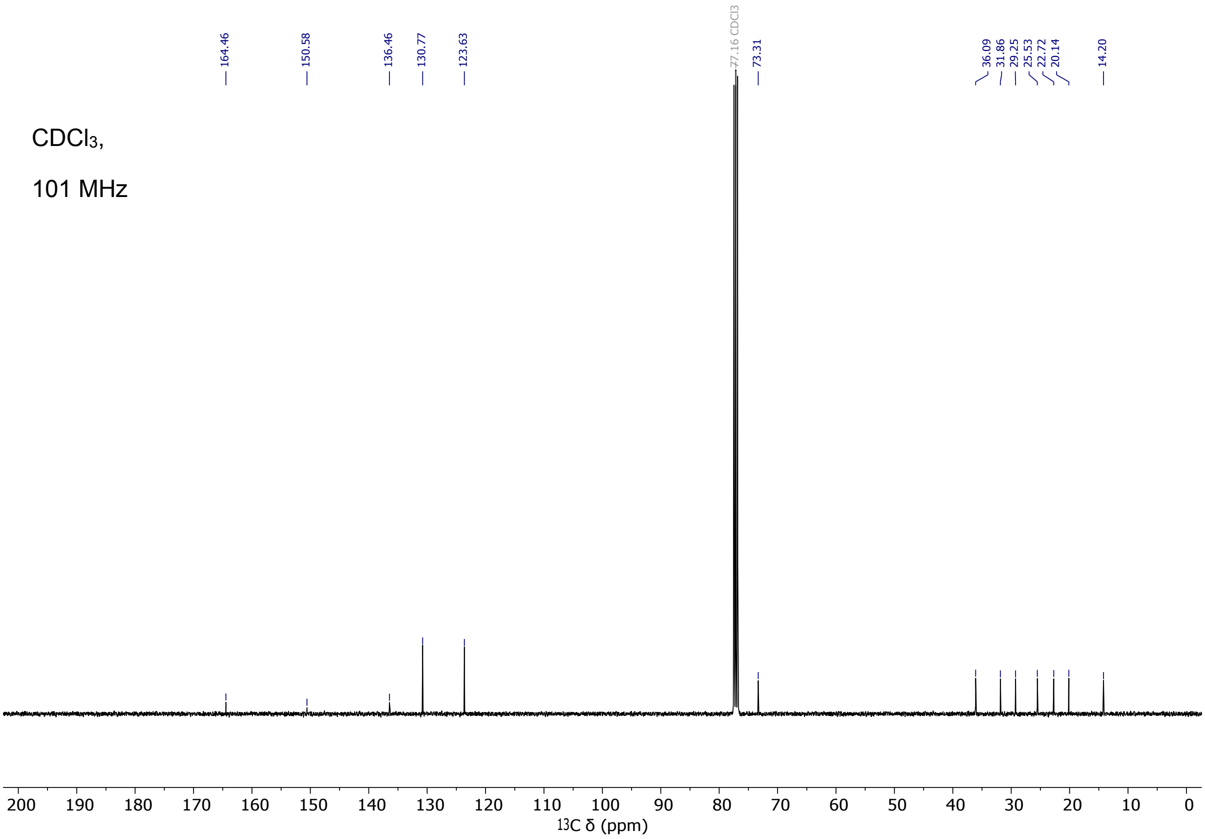

(*R*)-((2-chloropropoxy)methyl)benzene (2)

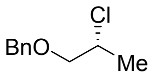

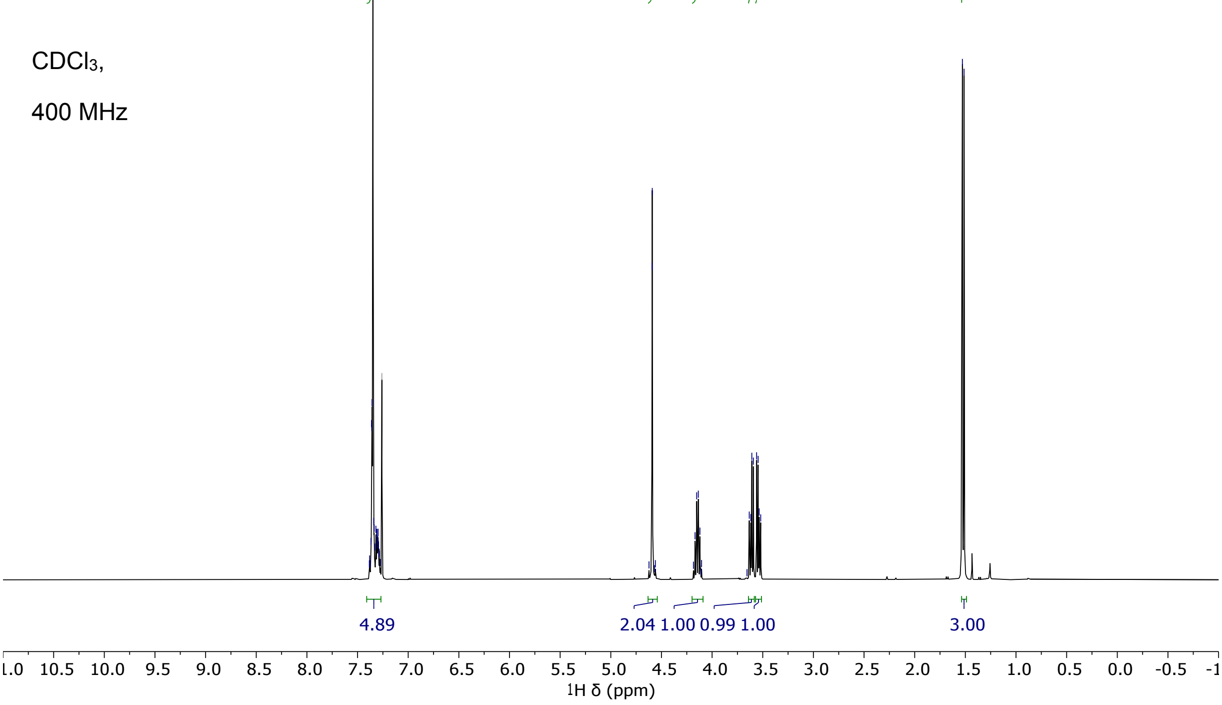

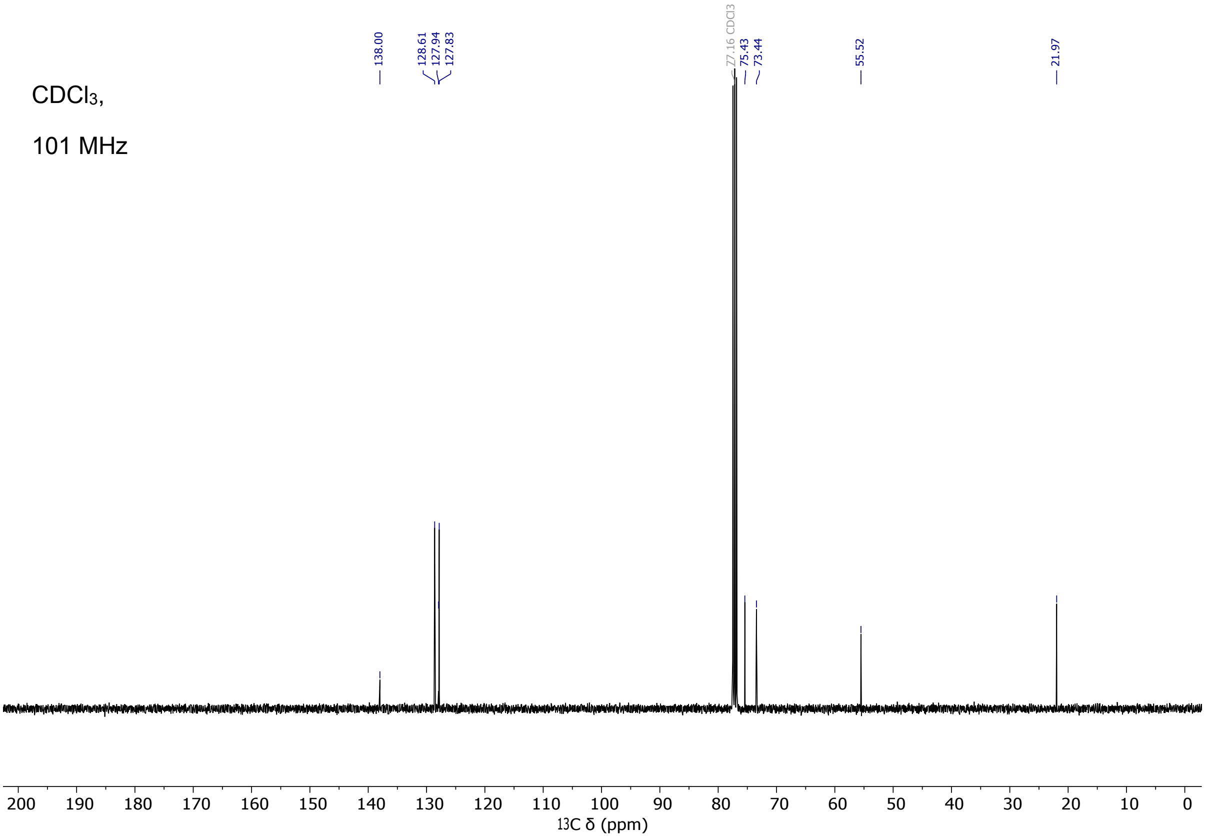

(*S*)-((1-(benzyloxy)propan-2-yl)oxy)triphenylphosphonium triflate (5a)

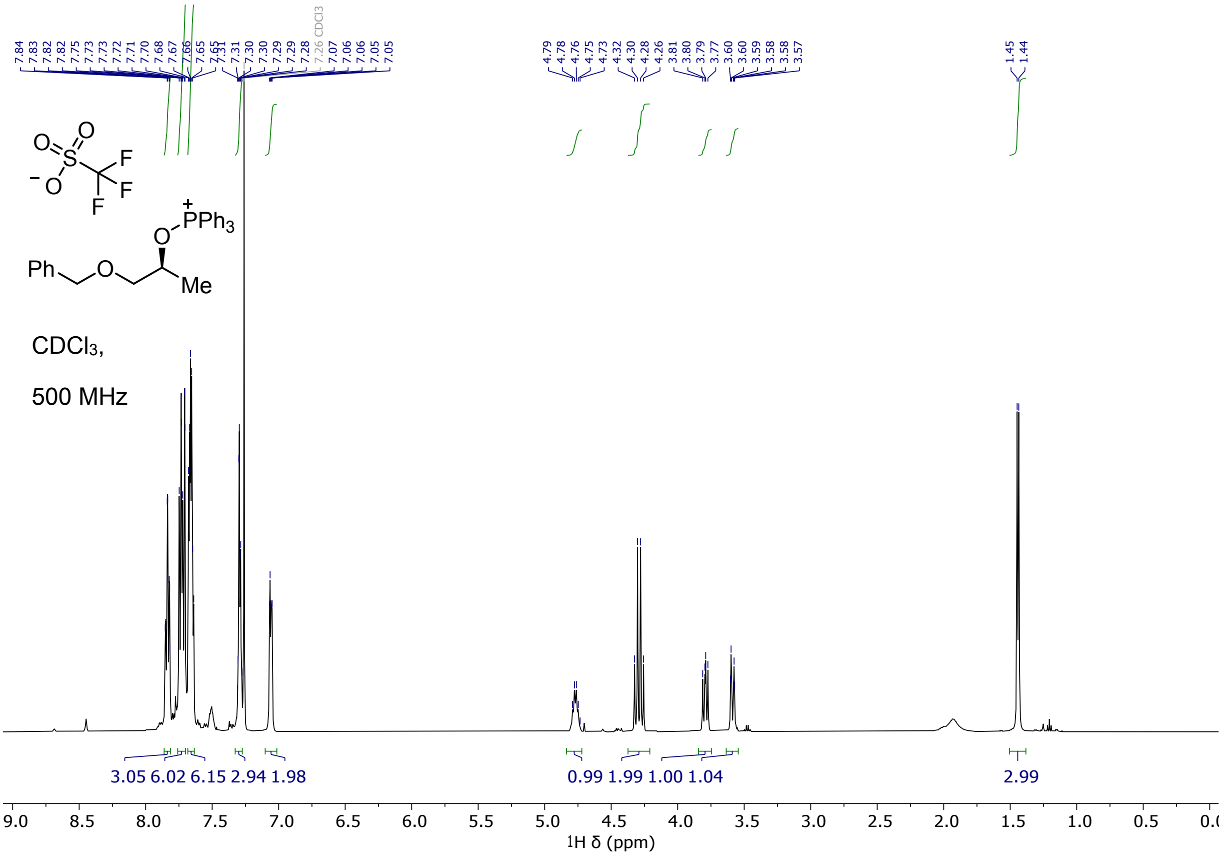

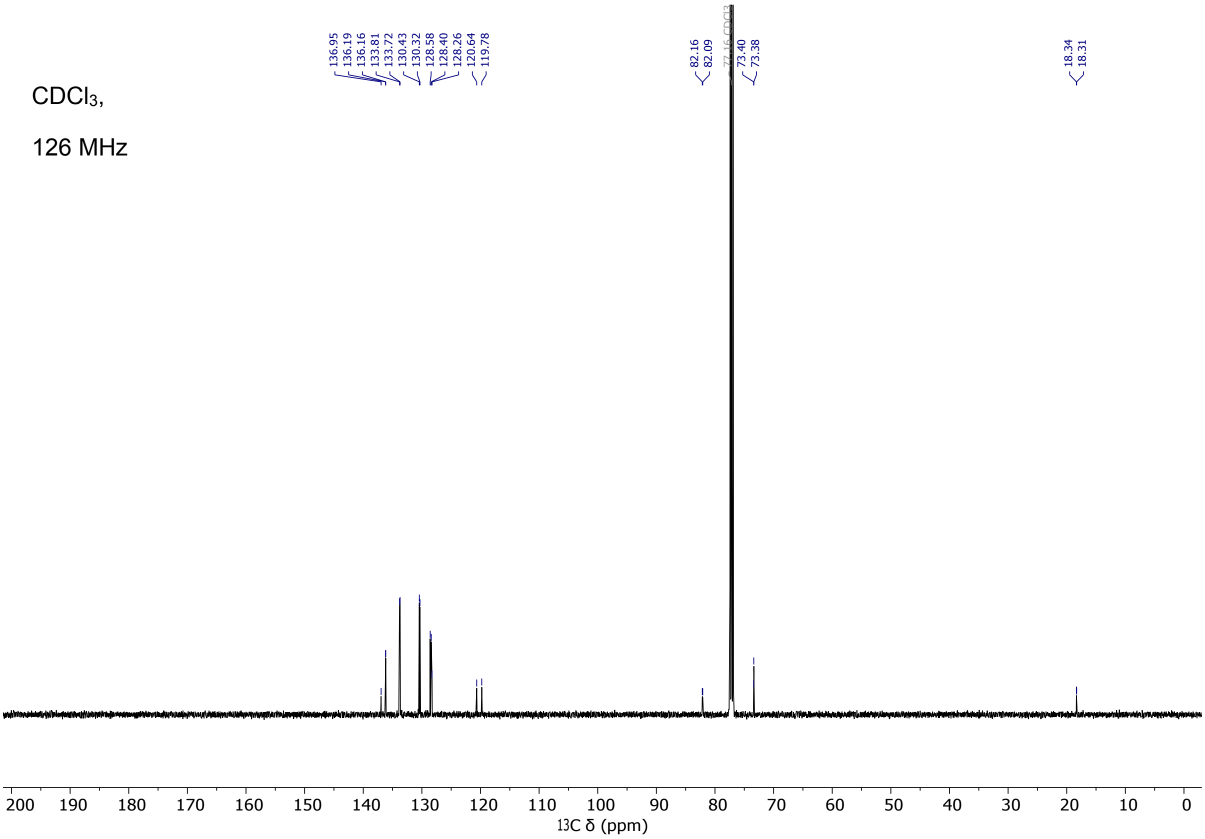

CDCl3,

202 MHz

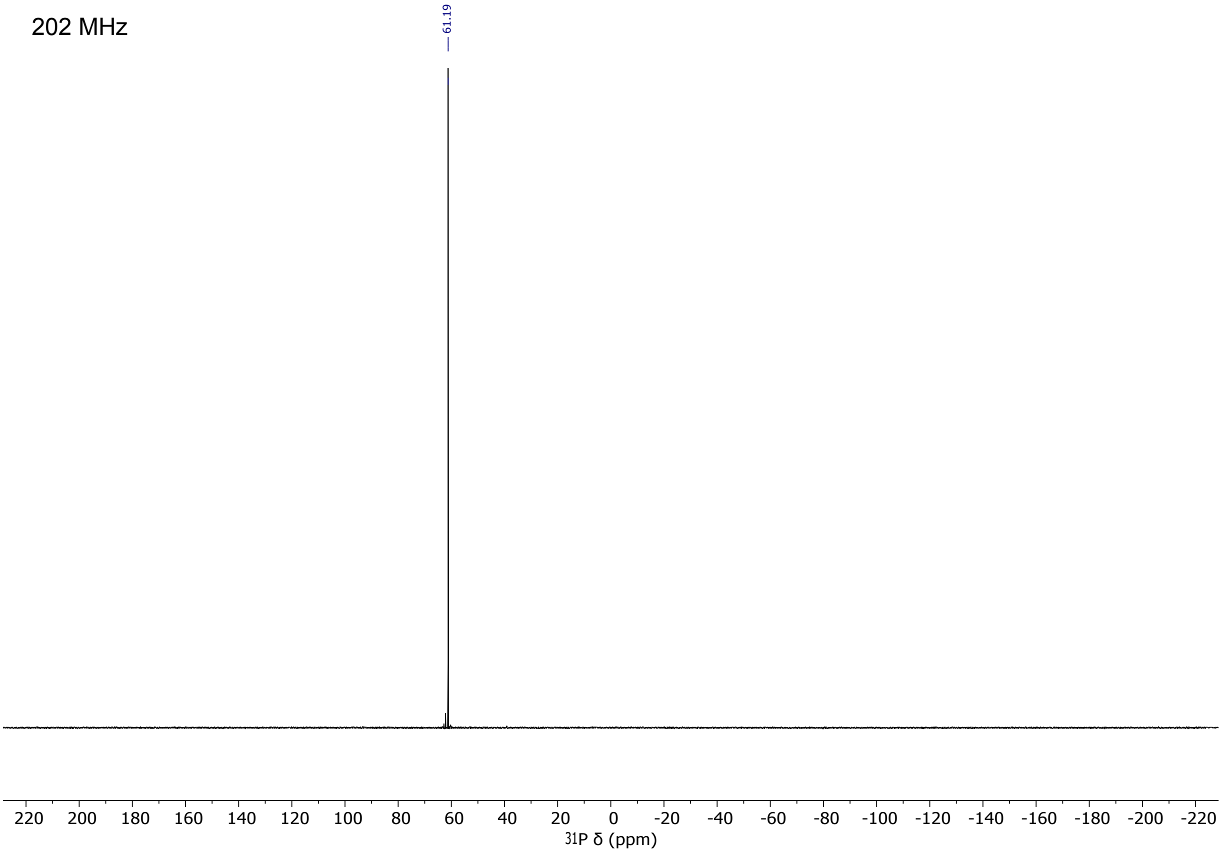

CDCl3,

376 MHz

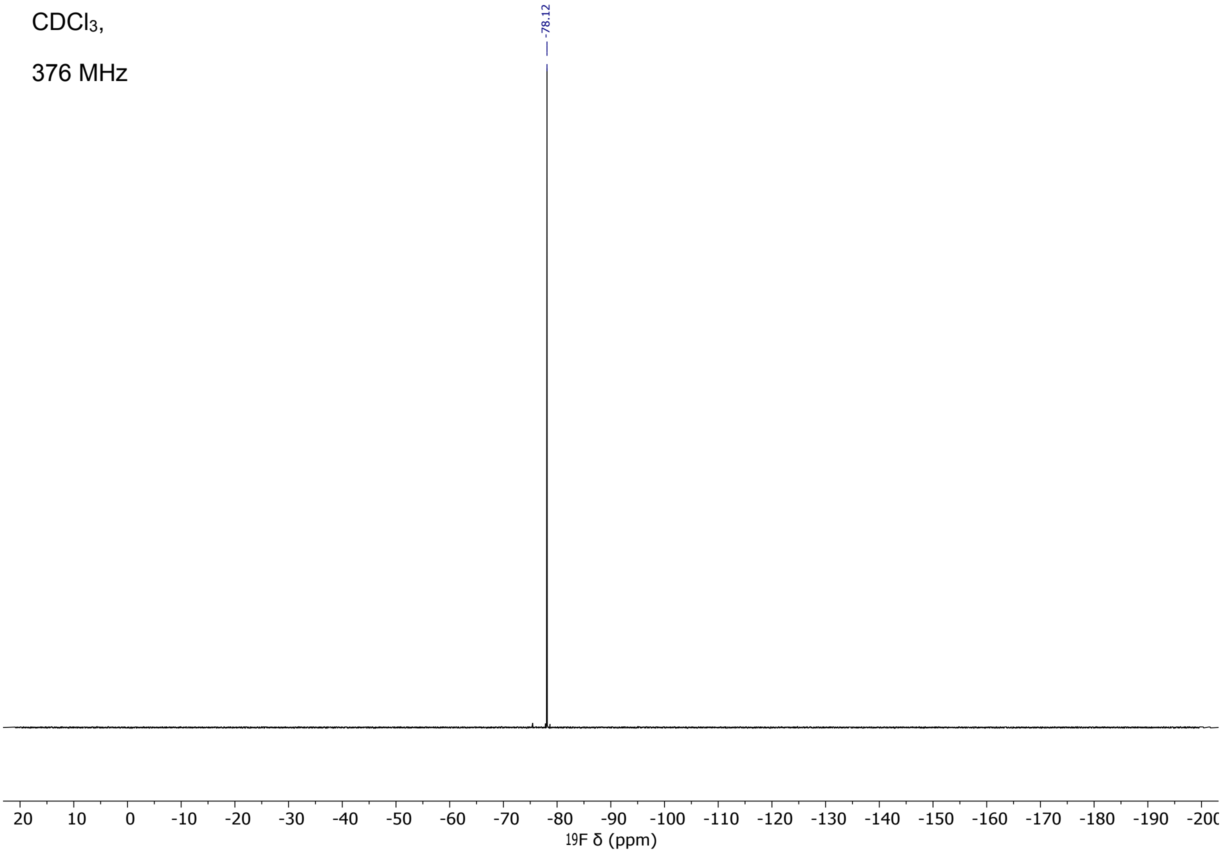

(*S*)-((1-(benzyloxy)propan-2-yl)oxy)triphenylphosphonium tetrakis[3,5-bis(trifluoromethyl) phenyl]borate (5b)

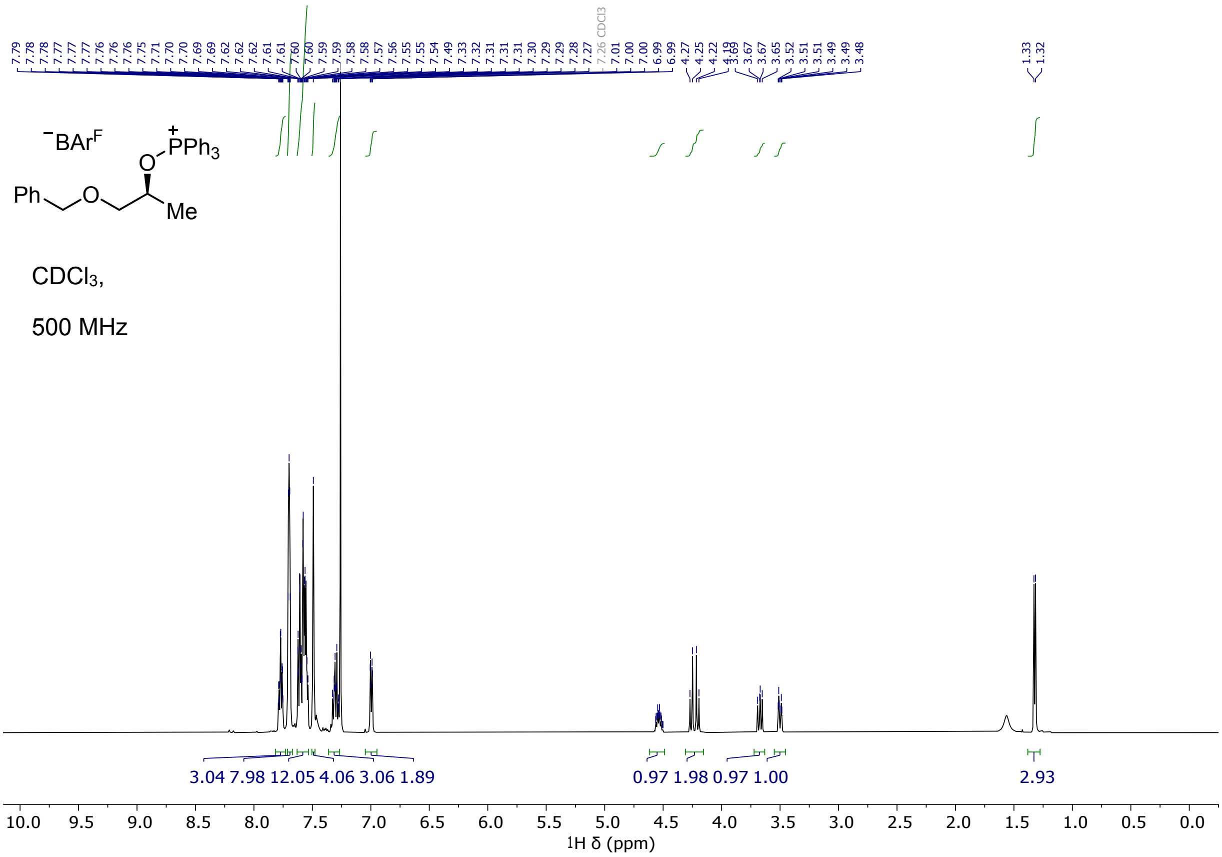

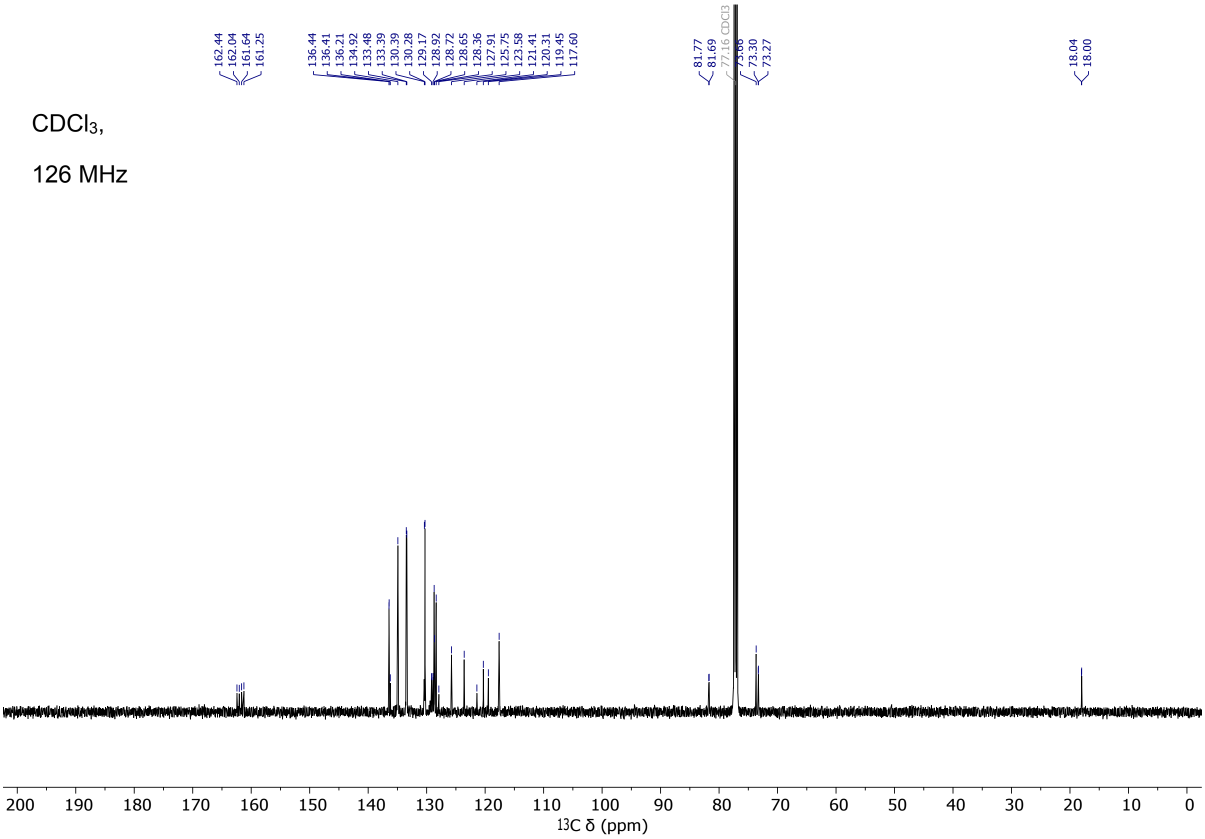

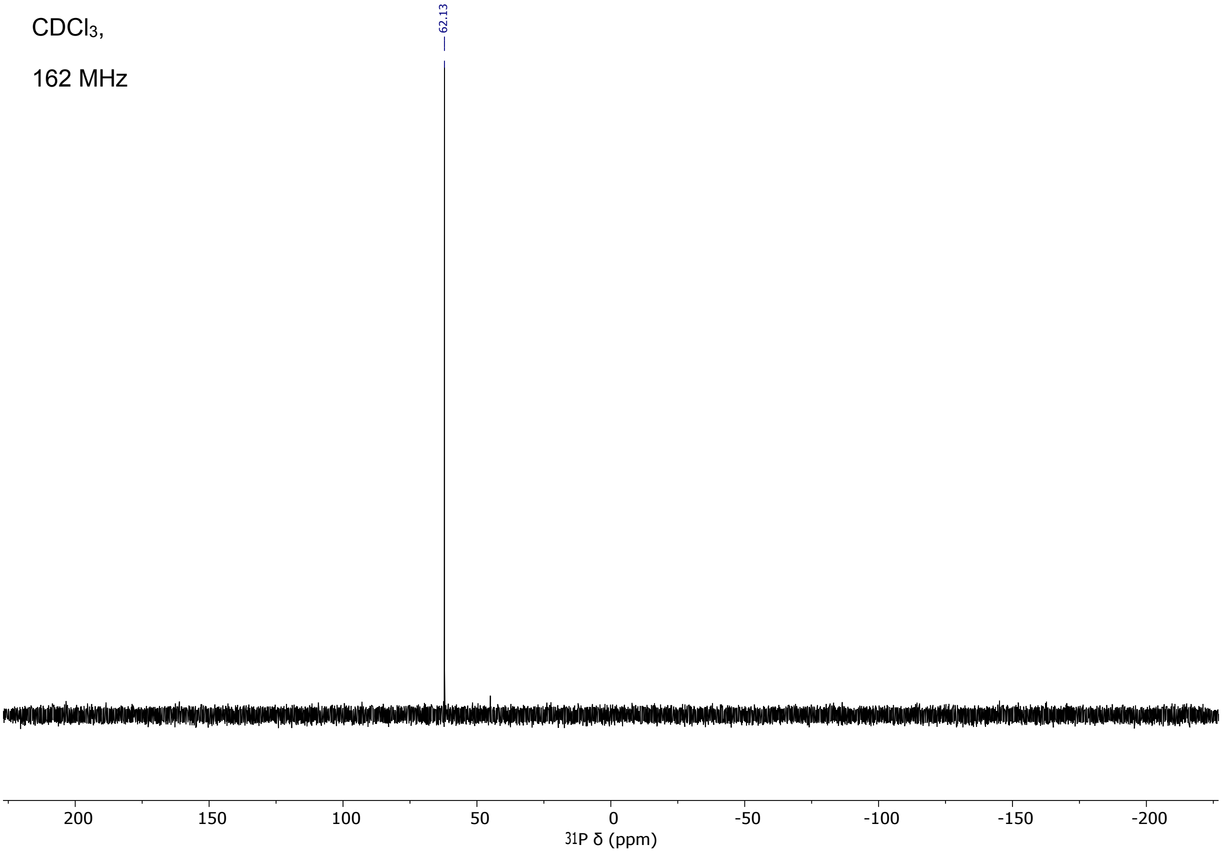
CDCl3,
162 MHz
62.13
200 150 100 50 0 -50 -100 -150 -200
31P δ (ppm)

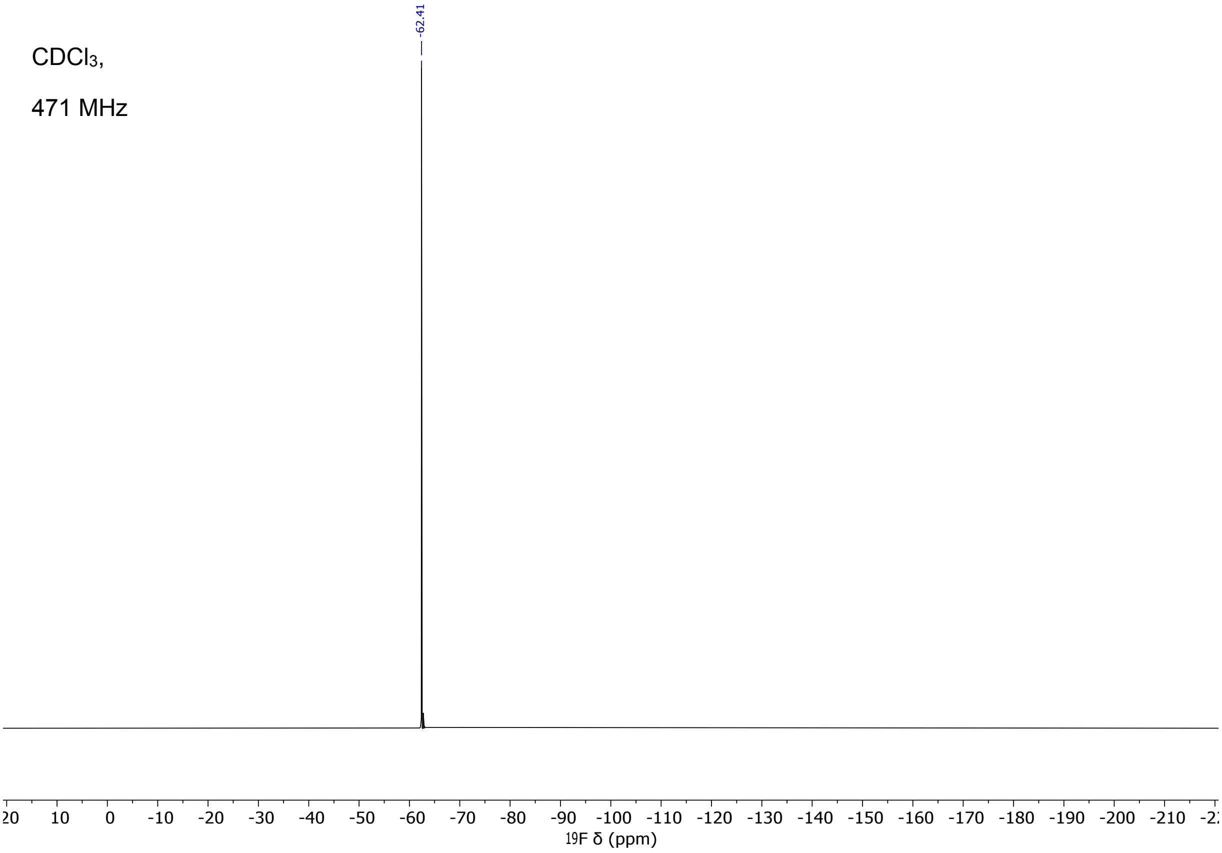
CDCl3,
471 MHz
-62.41
20 10 0 -10 -20 -30 -40 -50 -60 -70 -80 -90 -100 -110 -120 -130 -140 -150 -160 -170 -180 -190 -200 -210
19F δ (ppm)

References

(1) Thiyagarajan, S.; Gunanathan, C. Ruthenium-catalyzed selective hydrogenation of epoxides to secondary alcohols. *Org. Lett.* **2019**, *21* (23), 9774-9778. DOI: 10.1021/acs.orglett.9b03995.

(2) Beddoe, R. H.; Andrews, K. G.; Magné, V.; Cuthbertson, J. D.; Saska, J.; Shannon-Little, A. L.; Shanahan, S. E.; Sneddon, H. F.; Denton, R. M. Redox-neutral organocatalytic Mitsunobu reactions. *Science* **2019**, *365* (6456), 910-914. DOI: 10.1126/science.aax3353.

(3) Hayashi, Y.; Yamaguchi, J.; Sumiya, T.; Hibino, K.; Shoji, M. Direct Proline-Catalyzed Asymmetric α-Aminoxylation of Aldehydes and Ketones. *J. Org. Chem.* **2004**, *69* (18), 5966-5973. DOI: 10.1021/jo049338s.

(4) Xu, Z.; Shen, J.; Li, L.; Chen, W.; Li, S.; Jiang, J.; Zhang, Y. Q. (Salen) Titanium-Catalyzed Asymmetric Hydrogen Atom Transfer for Epoxides Reduction. *Angewandte Chemie International Edition* **2022**, *61* (50), e202214111. DOI: 10.1002/anie.202214111.

(5) Sayama, M.; Uwamizu, A.; Ikubo, M.; Chen, L.; Yan, G.; Otani, Y.; Inoue, A.; Aoki, J.; Ohwada, T. Switching lysophosphatidylserine G protein-coupled receptor agonists to antagonists by acylation of the hydrophilic serine amine. *Journal of Medicinal Chemistry* **2021**, *64* (14), 10059-10101. DOI: 10.1021/acs.jmedchem.1c00347.

(6) Ferreira, D. S.; Ferreira, J. G.; Everaldo Filho, F.; Princival, J. L. Tuning lipase-catalysed kinetic resolution of 2-substituted thiophenes and furans: a scalable chemoenzymatic route to masked γ-bis-oxo-alcohols. *Journal of Molecular Catalysis B: Enzymatic* **2016**, *126*, 37-45. DOI: 10.1016/j.molcatb.2016.01.014.

(7) Chen, R.; Kayrouz, C. S.; McAmis, E.; Clark, D. S.; Hartwig, J. F. Carbonic Anhydrase Variants Catalyze the Reduction of Dialkyl Ketones with High Enantioselectivity. *Angewandte Chemie International Edition* **2024**, *63* (40), e202407111. DOI: 10.1002/anie.202407111.

(8) Zhou, Y.; Porco, J. A.; Snyder, J. K. Synthesis of 5,6,7,8-tetrahydro-1,6-naphthyridines and related heterocycles by cobalt-catalyzed [2+2+2] cyclizations. *Org. Lett.* **2007**, *9* (3), 393-396. DOI: 10.1021/ol0625280.

(9) Horn, A.; Kazmaier, U. Stereoselective modification of N-(α-Hydroxyacyl)-glycinesters via palladium-catalyzed allylic alkylation. *Org. Lett.* **2019**, *21* (12), 4595-4599. DOI: 10.1021/acs.orglett.9b01497.

(10) van Leeuwen, T.; Buzzetti, L.; Perego, L. A.; Melchiorre, P. A Redox-Active Nickel Complex that Acts as an Electron Mediator in Photochemical Giese Reactions. *Angewandte Chemie International Edition* **2019**, *58* (15), 4953-4957. DOI: 10.1002/anie.201814497.

(11) Schuppan, J.; Minnaard, A. J.; Feringa, B. L. A catalytic and iterative route to β-substituted esters via highly enantioselective conjugate addition of dimethylzinc to unsaturated malonates. *Chem. Commun.* **2004**, (7), 792-793. DOI: 10.1039/B315871C.

(12) Zhang, K.; Chang, L.; An, Q.; Wang, X.; Zuo, Z. Dehydroxymethylation of Alcohols Enabled by Cerium Photocatalysis. *J. Am. Chem. Soc.* **2019**, *141* (26), 10556-10564. DOI: 10.1021/jacs.9b05932.

(13) Taguri, T.; Yamamoto, M.; Fujii, T.; Muraki, Y.; Ando, T. Synthesis of Four Stereoisomers of (S)-2-Methylpent-3-yl 3,13-Dimethylpentadecanoate, a Sex Pheromone of the Bagworm Moth Clania variegate, Using Stereospecific Inversion of Secondary Sulfonates as a Key Step. *Eur. J. Org. Chem.* **2013**, *2013* (30), 6924-6933. DOI: 10.1002/ejoc.201300874.

(14) Puente, Á.; He, S.; Corral-Bautista, F.; Ofial, A. R.; Mayr, H. Nucleophilic Reactivities of 2-Substituted Malonates. *Eur. J. Org. Chem.* **2016**, *2016* (10), 1841-1848. DOI: 10.1002/ejoc.201600107.

(15) Li, J.-Z.; Fan, S.-M.; Sun, X.-F.; Liu, S. Nano-K2CO3: preparation, characterization and evaluation of reactive activities. *RSC Advances* **2016**, *6* (3), 1865-1869. DOI: 10.1039/C5RA21597H.
(16) Banerjee, N.; Kumar, R.; Manna, B.; Banerjee, P. Strain-Releasing Hydrogenation of Donor–Acceptor Cyclopropanes and Cyclobutanes via Electrochemical Site Selective Carbonyl Reduction. *J. Org. Chem.* **2025**. DOI: 10.1021/acs.joc.5c00180.
(17) Lu, Y.-S.; Yu, W.-Y. Cp* Rh (III)-Catalyzed Cross-Coupling of Alkyltrifluoroborate with α-Diazomalonates for C(sp3)–C(sp3) Bond Formation. *Org. Lett.* **2016**, *18* (6), 1350-1353. DOI: 10.1021/acs.orglett.6b00283.
(18) Zięba, G.; Rojkiewicz, M.; Kozik, V.; Jarzembek, K.; Jarczyk, A.; Sochanik, A.; Kuś, P. The synthesis of new potential photosensitizers. 1. Mono-carboxylic acid derivatives of tetraphenylporphyrin. *Monatshefte für Chemie-Chemical Monthly* **2012**, *143*, 153-159. DOI: 10.1007/s00706-011-0586-3.
(19) Öhänen, T.; Wallen, E.; Kipeläinen, T.; Pätsi, H.; Jumppanen, M.; Leino, T.; Uhari-Väänänen, J.; Svarcbahs, R.; Eteläinen, T. PREP Binding Ligands. Finland WO2023209191A1, 2023.
(20) Hayashi, Y.; Cocco, E.; Odaira, H.; Matoba, H.; Mori, N. Oxidative Synthesis of α-Nitroketones from α-Substituted Malononitrile and Nitromethane Using Molecular Oxygen without Condensation Reagents. *Eur. J. Org. Chem.* **2024**, *27* (6), e202300964. DOI: 10.1002/ejoc.202300964.
(21) Tönjes, J.; Kell, L.; Werner, T. Organocatalytic stereospecific Appel reaction. *Org. Lett.* **2023**, *25* (51), 9114-9118. DOI: 10.1021/acs.orglett.3c03463.
(22) Saikam, V.; Dara, S.; Yadav, M.; Singh, P. P.; Vishwakarma, R. A. Dimethyltin dichloride catalyzed regioselective alkylation of cis-1,2-diols at room temperature. *J. Org. Chem.* **2015**, *80* (24), 11916-11925. DOI: 10.1021/acs.joc.5b01898.
(23) Sakai, K.; Oisaki, K.; Kanai, M. A bond-weakening borinate catalyst that improves the scope of the photoredox α-C–H alkylation of alcohols. *Synthesis* **2020**, *52* (15), 2171-2189. DOI: 10.1055/s-0040-1707114.
(24) Kallus, C.; Griebenow, N.; Wirtz, S.-N.; Baumann, K.; Gnoth, M. J.; Reinhard, F.; Hendrix, M. Phenylsulfonamide derivatives for treating Alzheimer's disease. WO2004080952A1, 2004.
(25) Hashmi, A. S. K.; Wölfle, M. Gold catalysis: benzanellation versus alkylidenecyclopentenone synthesis. *Tetrahedron* **2009**, *65* (44), 9021-9029. DOI: 10.1016/j.tet.2009.08.074.
(26) Ke, F.; Yu, C.; Li, X.; Sheng, H.; Song, Q. Copper-Catalyzed Radical Addition of Alkynols to Synthesize Difluoroheterocyclic Compounds. *Org. Lett.* **2023**, *25* (15), 2733-2738. DOI: 10.1021/acs.orglett.3c00968.
(27) Tang, X.; Chapman, C.; Whiting, M.; Denton, R. Development of a redox-free Mitsunobu reaction exploiting phosphine oxides as precursors to dioxyphosphoranes. *Chem. Commun.* **2014**, *50* (55), 7340-7343. DOI: 10.1039/C4CC02171A.
(28) Chighine, A.; Crosignani, S.; Arnal, M.-C.; Bradley, M.; Linclau, B. Microwave-assisted ester formation using O-alkylisoureas: a convenient method for the synthesis of esters with inversion of configuration. *J. Org. Chem.* **2009**, *74* (13), 4753-4762. DOI: 10.1021/jo900476y.
(29) ElMarrouni, A.; Ritts, C. B.; Balsells, J. Silyl-mediated photoredox-catalyzed Giese reaction: addition of non-activated alkyl bromides. *Chem. Sci.* **2018**, *9* (32), 6639-6646. DOI: 10.1039/C8SC02253D.
(30) Cosier, J. t.; Glazer, A. A nitrogen-gas-stream cryostat for general X-ray diffraction studies. *Applied Crystallography* **1986**, *19* (2), 105-107. DOI: 10.1107/S0021889886089835.

(31) Rigaku Oxford Diffraction. In *CrysAlisPro Software system, version 1.171.40.45a*, Rigaku Corporation, Oxford, UK, 2018.
(32) Dolomanov, O. V.; Bourhis, L. J.; Gildea, R. J.; Howard, J. A.; Puschmann, H. OLEX2: a complete structure solution, refinement and analysis program. *Applied Crystallography* **2009**, *42* (2), 339-341. DOI: 10.1107/S0021889808042726.
(33) Sheldrick, G. M. Crystal structure solution with ShelXT. *Acta Crystallogr. A* **2015**, *71*, 3-8. DOI: 10.1107/S2053273314026370.
(34) Sheldrick, G. M. Crystal structure solution with ShelXT. *Acta Crystallogr. C* **2015**, *71*, 3-8. DOI: 10.1107/S2053229614024218.
(35) "CheckCIF" can be found under http://checkcif.iucr.org
